# Supplementary material for: Prognostic accuracy of antenatal Doppler ultrasound for adverse perinatal outcomes in low-income and middle-income countries: a systematic review
Source: BMJ Open. 2021 Dec 2;11(12):e049799. doi: 10.1136/bmjopen-2021-049799 (PMC8640672; doi:10.1136/bmjopen-2021-049799)
Supplement: Supplementary data [file bmjopen-2021-049799supp001.pdf]

Appendix S1. Search strings for the databases used to retrieve articles

EMBASE

(‘developing countr*’:ti,ab,kw OR ‘developing nation*’:ti,ab,kw OR ‘developing population*’:ti,ab,kw OR ‘developing econom*’:ti,ab,kw OR ‘undeveloped countr*’:ti,ab,kw OR ‘undeveloped nation*’:ti,ab,kw OR ‘undeveloped economy’:ti,ab,kw OR ‘undeveloped economies’:ti,ab,kw OR ‘least developed countr*’:ti,ab,kw OR ‘least developed nation*’:ti,ab,kw OR ‘least developed economy’:ti,ab,kw OR ‘least developed economies’:ti,ab,kw OR ‘less-developed countr*’:ti,ab,kw OR ‘less-developed nation*’:ti,ab,kw OR ‘less-developed population’:ti,ab,kw OR ‘less-developed populations’:ti,ab,kw OR ‘less-developed econom*’:ti,ab,kw OR ‘lesser developed countr*’:ti,ab,kw OR ‘lesser developed nation*’:ti,ab,kw OR ‘lesser developed population’:ti,ab,kw OR ‘lesser developed populations’:ti,ab,kw OR ‘lesser developed economy’:ti,ab,kw OR ‘lesser developed economies’:ti,ab,kw OR ‘under-developed countr*’:ti,ab,kw OR ‘under-developed nation*’:ti,ab,kw OR ‘underdeveloped countr*’:ti,ab,kw OR ‘underdeveloped nation*’:ti,ab,kw OR ‘underdeveloped population*’:ti,ab,kw OR ‘underdeveloped econom*’:ti,ab,kw OR ‘low income countr*’:ti,ab,kw OR ‘middle income countr*’:ti,ab,kw OR ‘low income nation*’:ti,ab,kw OR ‘middle income nation*’:ti,ab,kw OR ‘low income population*’:ti,ab,kw OR ‘middle income population*’:ti,ab,kw OR ‘low income econom*’:ti,ab,kw OR ‘middle income econom*’:ti,ab,kw OR ‘lower income countr*’:ti,ab,kw OR ‘lower income nation*’:ti,ab,kw OR ‘lower income population*’:ti,ab,kw OR ‘lower income economy’:ti,ab,kw OR ‘lower income economies’:ti,ab,kw OR ‘resource limited’:ti,ab,kw OR ‘low resource countr*’:ti,ab,kw OR ‘lower resource countr*’:ti,ab,kw OR ‘low resource nation*’:ti,ab,kw OR ‘low resource population*’:ti,ab,kw OR ‘low resource economy’:ti,ab,kw OR ‘low resource economies’:ti,ab,kw OR ‘underserved countr*’:ti,ab,kw OR ‘underserved nation*’:ti,ab,kw OR ‘underserved population*’:ti,ab,kw OR ‘underserved economy’:ti,ab,kw OR ‘underserved economies’:ti,ab,kw OR ‘under-served country’:ti,ab,kw OR ‘under-served countries’:ti,ab,kw OR ‘under-served nation’:ti,ab,kw OR ‘under-served nations’:ti,ab,kw OR ‘under-served population’:ti,ab,kw OR ‘under-served populations’:ti,ab,kw OR ‘underserved economy’:ti,ab,kw OR ‘underserved economies’:ti,ab,kw OR ‘derived countr*’:ti,ab,kw OR ‘deprived nation’:ti,ab,kw OR ‘deprived nations’:ti,ab,kw OR ‘derived population*’:ti,ab,kw OR ‘deprived economy’:ti,ab,kw OR ‘deprived economies’:ti,ab,kw OR ‘poor countr*’:ti,ab,kw OR ‘poor nation*’:ti,ab,kw OR ‘poor population*’:ti,ab,kw OR ‘poor econom*’:ti,ab,kw OR ‘poorer countr*’:ti,ab,kw OR ‘poorer nation*’:ti,ab,kw OR ‘poorer population*’:ti,ab,kw OR ‘poorer econom*’:ti,ab,kw OR ‘lmic’:ti,ab,kw OR ‘lmics’:ti,ab,kw OR ‘lami’:ti,ab,kw OR ‘transitional countr*’:ti,ab,kw OR ‘transitional nation’:ti,ab,kw OR ‘transitional nations’:ti,ab,kw OR ‘transitional econom*’:ti,ab,kw OR ‘transition countr*’:ti,ab,kw OR ‘transition nation*’:ti,ab,kw OR ‘transition econom*’:ti,ab,kw OR low ‘resource setting*’:ti,ab,kw OR ‘lower resource setting*’:ti,ab,kw OR ‘middle resource setting*’:ti,ab,kw OR ‘Third World*’:ti,ab,kw OR ‘south east asia*’:ti,ab,kw OR ‘middle east*’:ti,ab,kw OR ‘Afghan*’:ti,ab,kw OR ‘Angola*’:ti,ab,kw OR ‘Angolese*’:ti,ab,kw OR ‘Angolian*’:ti,ab,kw OR ‘Armenia*’:ti,ab,kw OR ‘Bangladesh*’:ti,ab,kw OR ‘Benin*’:ti,ab,kw OR ‘Bhutan*’:ti,ab,kw OR ‘Birma*’:ti,ab,kw OR ‘Burma*’:ti,ab,kw OR ‘Birmese*’:ti,ab,kw OR ‘Burmese*’:ti,ab,kw OR ‘Boliv*’:ti,ab,kw OR ‘Botswan*’:ti,ab,kw OR ‘burkina Faso*’:ti,ab,kw OR ‘Burundi*’:ti,ab,kw OR ‘Cabo Verde*’:ti,ab,kw OR ‘Cambod*’:ti,ab,kw OR ‘Cameroon*’:ti,ab,kw OR ‘Cape Verd*’:ti,ab,kw OR ‘Central Africa*’:ti,ab,kw OR ‘Chad’:ti,ab,kw OR ‘Comoro*’:ti,ab,kw OR ‘Congo*’:ti,ab,kw OR ‘Cote d/Ivoire*’:ti,ab,kw OR ‘Djibouti*’:ti,ab,kw OR ‘East Africa*’:ti,ab,kw OR ‘Eastern Africa*’:ti,ab,kw OR ‘Egypt*’:ti,ab,kw OR ‘El Salvador*’:ti,ab,kw OR ‘Equatorial Guinea*’:ti,ab,kw OR ‘Eritre*’:ti,ab,kw OR ‘Ethiopia*’:ti,ab,kw OR ‘Gabon*’:ti,ab,kw OR ‘Gambia*’:ti,ab,kw OR ‘Gaza*’:ti,ab,kw OR ‘Georgia Republic’/exp OR

'Ghan*':ti,ab,kw OR 'Guatemal*':ti,ab,kw OR 'Guinea':ti,ab,kw OR 'Haiti*':ti,ab,kw OR 'Hondur*':ti,ab,kw OR 'India*':ti,ab,kw OR 'Indones*':ti,ab,kw OR 'Ivory Coast*':ti,ab,kw OR 'Kenya*':ti,ab,kw OR 'Kiribati*':ti,ab,kw OR 'Kosovo*':ti,ab,kw OR 'Kyrgyz*':ti,ab,kw OR 'Lao PDR*':ti,ab,kw OR 'Laos*':ti,ab,kw OR 'Lesotho*':ti,ab,kw OR 'Liberia*':ti,ab,kw OR 'Madagascar*':ti,ab,kw OR 'Malaw*':ti,ab,kw OR 'Mali':ti,ab,kw OR 'Mauritan*':ti,ab,kw OR 'Mauriti*':ti,ab,kw OR 'Micronesi*':ti,ab,kw OR 'Mocambiqu*':ti,ab,kw OR 'Moldov*':ti,ab,kw OR 'Mongolia*':ti,ab,kw OR 'Morocc*':ti,ab,kw OR 'Mozambiqu*':ti,ab,kw OR 'Myanmar*':ti,ab,kw OR 'Namibia*':ti,ab,kw OR 'Nepal*':ti,ab,kw OR 'Nicaragua*':ti,ab,kw OR 'Niger*':ti,ab,kw OR 'North Korea*':ti,ab,kw OR 'Northern Korea*':ti,ab,kw OR 'Democratic People/s Republic of Korea':ti,ab,kw OR 'Pakistan*':ti,ab,kw OR 'Papua New Guinea*':ti,ab,kw OR 'Philippine*':ti,ab,kw OR 'Principe':ti,ab,kw OR 'Rhodesia*':ti,ab,kw OR 'Rwanda*':ti,ab,kw OR 'Samoa*':ti,ab,kw OR 'Sao Tome*':ti,ab,kw OR 'Senegal*':ti,ab,kw OR 'Sierra Leone*':ti,ab,kw OR 'Solomon Islands*':ti,ab,kw OR 'Somalia*':ti,ab,kw OR 'South Africa*':ti,ab,kw OR 'South Sudan*':ti,ab,kw OR 'Southern Africa*':ti,ab,kw OR 'Sri Lanka*':ti,ab,kw OR 'Sub Saharan Africa*':ti,ab,kw OR 'Subsaharan Africa*':ti,ab,kw OR 'Sudan*':ti,ab,kw OR 'Swaziland*':ti,ab,kw OR 'Syria*':ti,ab,kw OR 'Tajikist*':ti,ab,kw OR 'Tanzan*':ti,ab,kw OR 'Timor*':ti,ab,kw OR 'Togo*':ti,ab,kw OR 'Tonga*':ti,ab,kw OR 'Tunis*':ti,ab,kw OR 'Ugand*':ti,ab,kw OR 'Ukrain*':ti,ab,kw OR 'Uzbekistan*':ti,ab,kw OR 'Vanuatu*':ti,ab,kw OR 'Vietnam*':ti,ab,kw OR 'West Africa*':ti,ab,kw OR 'West Bank*':ti,ab,kw OR 'Western Africa*':ti,ab,kw OR 'Yemen*':ti,ab,kw OR 'Zaire*':ti,ab,kw OR 'Zambia*':ti,ab,kw OR 'Zimbabw*':ti,ab,kw)

AND

('Umbilical Arter*'/exp OR 'Uterine Artery'/exp OR 'Middle Cerebral Artery'/exp OR 'Ductus Venosus'/exp OR 'Umbilical Vein*'/exp OR 'Inferior Cava Vein'/exp OR 'Umbilical Arter*':ti,ab,kw OR 'Uterine Arter*':ti,ab,kw OR 'Middle Cerebral Arter*':ti,ab,kw OR 'Patent Ductus Venosus':ti,ab,kw OR 'Umbilical Vein*':ti,ab,kw OR 'Inferior Vena Cava':ti,ab,kw OR 'Cerebroplacental Ratio':ti,ab,kw OR 'CPR':ti,ab,kw OR 'Fetal Descending Aorta':ti,ab,kw OR 'FDA':ti,ab,kw OR 'Doppler Ultrasonography'/exp OR 'Doppler Ultrasound*':ti,ab,kw OR 'Doppler Ultrasonography':ti,ab,kw OR 'Uterine Artery Doppler':ti,ab,kw)

AND

('Stillbirth':ti,ab,kw OR 'Perinatal Death':ti,ab,kw OR 'Cesarean Section*':ti,ab,kw OR 'Caesarean Section*':ti,ab,kw OR 'Acidosis':ti,ab,kw OR 'Premature Birth':ti,ab,kw OR 'Neonatal Intensive Care':ti,ab,kw OR 'Fetal Growth Retard*':ti,ab,kw OR 'Newborn Respiratory Distress Syndrome*':ti,ab,kw OR 'Gestational Age':ti,ab,kw OR 'Birth Weight':ti,ab,kw OR 'Asphyxia Neonatorum':ti,ab,kw OR 'Apgar Score*':ti,ab,kw OR 'Length of Stay':ti,ab,kw OR 'Stillbirth'/exp OR 'Perinatal Death'/exp OR 'Perinatal Mortality'/exp OR 'Cesarean Section'/exp OR 'Acidosis'/exp OR 'Prematurity'/exp OR 'Newborn Intensive Care'/exp OR 'Intrauterine Growth Retardation'/exp OR 'Neonatal Respiratory Distress Syndrome'/exp OR 'Gestational Age'/exp OR 'Birth Weight'/exp OR 'Newborn Hypoxia'/exp OR 'Apgar Score'/exp OR 'Length of Stay'/exp OR 'Pregnancy':ti,ab,kw OR 'Pregnancies':ti,ab,kw OR 'Gestation':ti,ab,kw OR 'Pregnant':ti,ab,kw OR 'Pregnancy'/exp)

PUBMED (MEDLINE)

("Developing Countries"[Mesh] OR developing countr*[tiab] OR developing nation*[tiab] OR developing population*[tiab] OR developing econom*[tiab] OR undeveloped countr*[tiab] OR undeveloped nation*[tiab] OR "undeveloped economy"[tiab] OR "undeveloped economies"[tiab] OR least developed countr*[tiab] OR least developed nation*[tiab] OR "least developed economy"[tiab] OR "least developed economies"[tiab] OR less-developed countr*[tiab] OR less-developed nation*[tiab] OR "less-developed population"[tiab] OR "less-developed populations"[tiab] OR less-developed econom*[tiab] OR lesser developed countr*[tiab] OR lesser developed nation*[tiab] OR "lesser developed population"[tiab] OR "lesser developed populations"[tiab] OR "lesser developed economy"[tiab] OR "lesser developed economies"[tiab] OR under-developed countr*[tiab] OR under-developed nation*[tiab] OR underdeveloped countr*[tiab] OR underdeveloped nation*[tiab] OR underdeveloped population*[tiab] OR underdeveloped econom*[tiab] OR low income countr*[tiab] OR middle income countr*[tiab] OR low income nation*[tiab] OR middle income nation*[tiab] OR low income population*[tiab] OR middle income population*[tiab] OR low income econom*[tiab] OR middle income econom*[tiab] OR lower income countr*[tiab] OR lower income nation*[tiab] OR lower income population*[tiab] OR "lower income economy"[tiab] OR "lower income economies"[tiab] OR resource limited[tiab] OR low resource countr*[tiab] OR lower resource countr*[tiab] OR low resource nation*[tiab] OR low resource population*[tiab] OR "low resource economy"[tiab] OR "low resource economies"[tiab] OR underserved countr*[tiab] OR underserved nation*[tiab] OR underserved population*[tiab] OR "underserved economy"[tiab] OR "underserved economies"[tiab] OR "under-served country"[tiab] OR "under-served countries"[tiab] OR "under-served nation"[tiab] OR "under-served nations"[tiab] OR "under-served population"[tiab] OR "under-served populations"[tiab] OR "underserved economy"[tiab] OR "underserved economies"[tiab] OR derived countr*[tiab] OR "deprived nation"[tiab] OR "deprived nations"[tiab] OR derived population*[tiab] OR "deprived economy"[tiab] OR "deprived economies"[tiab] OR poor countr*[tiab] OR poor nation*[tiab] OR poor population*[tiab] OR poor econom*[tiab] OR poorer countr*[tiab] OR poorer nation*[tiab] OR poorer population*[tiab] OR poorer econom*[tiab] OR lmic[tiab] OR lmics[tiab] OR lami[tiab] OR transitional countr*[tiab] OR "transitional nation"[tiab] OR "transitional nations"[tiab] OR transitional econom*[tiab] OR transition countr*[tiab] OR transition nation*[tiab] OR transition econom*[tiab] OR low resource setting*[tiab] OR lower resource setting*[tiab] OR middle resource setting*[tiab] OR Third World*[tiab] OR south east asia*[tw] OR middle east*[tw] OR Afghan*[tw] OR Angola*[tw] OR Angolese*[tw] OR Angolian*[tw] OR Armenia*[tw] OR Bangladesh*[tw] OR Benin*[tw] OR Bhutan*[tw] OR Birma*[tw] OR Burma*[tw] OR Birmese*[tw] OR Burmese*[tw] OR Boliv*[tw] OR Botswan*[tw] OR burkina Faso*[tw] OR Burundi*[tw] OR Cabo Verde*[tw] OR Cambod*[tw] OR Cameroon*[tw] OR Cape Verd*[tw] OR Central Africa*[tw] OR Chad[tw] OR Comoro*[tw] OR Congo*[tw] OR Cote d'Ivoire*[tw] OR Djibouti*[tw] OR East Africa*[tw] OR Eastern Africa*[tw] OR Egypt*[tw] OR El Salvador*[tw] OR Equatorial Guinea*[tw] OR Eritre*[tw] OR Ethiopia*[tw] OR Gabon*[tw] OR Gambia*[tw] OR Gaza*[tw] OR "Georgia Republic"[Mesh] OR Ghan*[tw] OR Guatemal*[tw] OR Guinea[tw] OR Haiti*[tw] OR Hondur*[tw] OR India*[tw] OR Indones*[tw] OR Ivory Coast*[tw] OR Kenya*[tw] OR Kiribati*[tw] OR Kosovo*[tw] OR Kyrgyz*[tw] OR Lao PDR*[tw] OR Laos*[tw] OR Lesotho*[tw] OR Liberia*[tw] OR Madagascar*[tw] OR Malaw*[tw] OR Mali[tw] OR Mauritan*[tw] OR Mauriti*[tw] OR Micronesi*[tw] OR Mocambiqu*[tw] OR Moldov*[tw] OR Mongolia*[tw] OR Morocc*[tw] OR Mozambiqu*[tw] OR Myanmar*[tw] OR Namibia*[tw] OR Nepal*[tw] OR Nicaragua*[tw] OR Niger*[tw] OR North Korea*[tw] OR Northern Korea*[tw] OR "Democratic People s Republic of Korea"[tiab] OR "Democratic People's Republic of Korea"[Mesh] OR Pakistan*[tw] OR Papua New Guinea*[tw] OR Philippine*[tw] OR Principe[tw] OR Rhodesia*[tw] OR Rwanda*[tw] OR Samoa*[tw] OR Sao Tome*[tw] OR Senegal*[tw] OR Sierra Leone*[tw] OR Solomon Islands*[tw]

OR Somalia*[tw] OR South Africa*[tw] OR South Sudan*[tw] OR Southern Africa*[tw] OR Sri Lanka*[tw] OR Sub Saharan Africa*[tw] OR Subsaharan Africa*[tw] OR Sudan*[tw] OR Swaziland*[tw] OR Syria*[tw] OR Tajikist*[tw] OR Tanzan*[tw] OR Timor*[tw] OR Togo*[tw] OR Tonga*[tw] OR Tunis*[tw] OR Ugand*[tw] OR Ukrain*[tw] OR Uzbekistan*[tw] OR Vanuatu*[tw] OR Vietnam*[tw] OR West Africa*[tw] OR West Bank*[tw] OR Western Africa*[tw] OR Yemen*[tw] OR Zaire*[tw] OR Zambia*[tw] OR Zimbabw*[tw])

AND

("Umbilical Arteries"[Mesh] OR "Uterine Artery"[Mesh] OR "Middle Cerebral Artery"[Mesh] OR "Ductus Venosus" [Supplementary Concept] OR "Umbilical Veins"[Mesh] OR "Vena Cava, Inferior"[Mesh] OR Umbilical Arter*[tiab] OR Uterine Arter*[tiab] OR Middle Cerebral Arter*[tiab] OR Patent Ductus Venosus[tiab] OR Umbilical Vein*[tiab] OR Inferior Vena Cava[tiab] OR Cerebroplacental Ratio[tiab] OR CPR[tiab] OR Fetal Descending Aorta[tiab] OR FDA[tiab] OR "Ultrasonography, Doppler"[Mesh] OR Doppler Ultrasound*[Title/Abstract] OR Doppler Ultrasonography[Title/Abstract] OR Uterine Artery Doppler[Title/Abstract])

AND

("Stillbirth"[tiab] OR "Perinatal Death"[tiab] OR "Cesarean Section*"[tiab] OR "Caesarean Section*"[tiab] OR Acidosis[tiab] OR Premature Birth[tiab] OR Neonatal Intensive Care"[tiab] OR Fetal Growth Retard*[tiab] OR Newborn Respiratory Distress Syndrome*[tiab] OR Gestational Age[tiab] OR Birth Weight[tiab] OR Asphyxia Neonatorum[tiab] OR Apgar Score*[tiab] OR Length of Stay"[tiab] OR "Stillbirth"[Mesh] OR "Perinatal Death"[Mesh] OR "Cesarean Section"[Mesh] OR "Acidosis"[Mesh] OR "Premature Birth"[Mesh] OR "Intensive Care, Neonatal"[Mesh] OR "Fetal Growth Retardation"[Mesh] OR "Respiratory Distress Syndrome, Newborn"[Mesh] OR "Gestational Age"[Mesh] OR "Birth Weight"[Mesh] OR "Asphyxia Neonatorum"[Mesh] OR "Apgar Score"[Mesh] OR "Length of Stay"[Mesh] OR Pregnancy[Title/Abstract] OR Pregnancies[Title/Abstract] OR Gestation[Title/Abstract] OR Pregnant[Title/Abstract] OR "Pregnancy"[Mesh])

COCHRANE

‘developing countr*’ OR ‘developing nation*’ OR ‘developing population*’ OR ‘developing econom*’ OR ‘undeveloped countr*’ OR ‘undeveloped nation*’ OR ‘undeveloped economy’ OR ‘undeveloped economies’ OR ‘least developed countr*’ OR ‘least developed nation*’ OR ‘least developed economy’ OR ‘least developed economies’ OR ‘less-developed countr*’ OR ‘less-developed nation*’ OR ‘less-developed population’ OR ‘less-developed populations’ OR ‘less-developed econom*’ OR ‘lesser developed countr*’ OR ‘lesser developed nation*’ OR ‘lesser developed population’ OR ‘lesser developed populations’ OR ‘lesser developed economy’ OR ‘lesser developed economies’ OR ‘under-developed countr*’ OR ‘under-developed nation*’ OR ‘underdeveloped countr*’OR ‘underdeveloped nation*’ OR ‘underdeveloped population*’ OR ‘underdeveloped econom*’ OR ‘low income countr*’ OR ‘middle income countr*’ OR ‘low income nation*’ OR ‘middle income nation*’ OR ‘low income population*’ OR ‘middle income population*’ OR ‘low income econom*’ OR ‘middle income econom*’ OR ‘lower income countr*’ OR ‘lower income nation*’ OR ‘lower income population*’ OR ‘lower income economy’ OR ‘lower income economies’ OR ‘resource limited’ OR ‘low resource countr*’ OR ‘lower resource countr*’ OR ‘low resource nation*’ OR ‘low resource population*’ OR ‘low resource economy’ OR ‘low resource economies’ OR ‘underserved countr*’ OR ‘underserved nation*’ OR ‘underserved

population*' OR 'underserved economy' OR 'underserved economies' OR 'under-served country' OR 'under-served countries' OR 'under-served nation' OR 'under-served nations' OR 'under-served population' OR 'under-served populations' OR 'underserved economy' OR 'underserved economies' OR 'derived countr*' OR 'deprived nation' OR 'deprived nations' OR 'derived population*' OR 'deprived economy' OR 'deprived economies' OR 'poor countr*' OR 'poor nation*' OR 'poor population*' OR 'poor econom*' OR 'poorer countr*' OR 'poorer nation*' OR 'poorer population*' OR 'poorer econom*' OR 'lmic' OR 'lmics' OR 'lami' OR 'transitional countr*' OR 'transitional nation' OR 'transitional nations' OR 'transitional econom*' OR 'transition countr*' OR 'transition nation*' OR 'transition econom*' OR low 'resource setting*' OR 'lower resource setting*' OR 'middle resource setting*' OR 'Third World*' OR 'south east asia*' OR 'middle east*' OR 'Afghan*' OR 'Angola*' OR 'Angolese*' OR 'Angolian*' OR 'Armenia*' OR 'Bangladesh*' OR 'Benin*' OR 'Bhutan*' OR 'Birma*' OR 'Burma*' OR 'Birmese*' OR 'Burmese*' OR 'Boliv*' OR 'Botswan*' OR 'burkina Faso*' OR 'Burundi*' OR 'Cabo Verde*' OR 'Cambod*' OR 'Cameroon*' OR 'Cape Verd*' OR 'Central Africa*' OR 'Chad' OR 'Comoro*' OR 'Congo*' OR 'Cote d'Ivoire*' OR 'Djibouti*' OR 'East Africa*' OR 'Eastern Africa*' OR 'Egypt*' OR 'El Salvador*' OR 'Equatorial Guinea*' OR 'Eritre*' OR 'Ethiopia*' OR 'Gabon*' OR 'Gambia*' OR 'Gaza*' OR 'Georgia Republic' OR 'Ghan*' OR 'Guatemal*' OR 'Guinea' OR 'Haiti*' OR 'Hondur*' OR 'India*' OR 'Indones*' OR 'Ivory Coast*' OR 'Kenya*' OR 'Kiribati*' OR 'Kosovo*' OR 'Kyrgyz*' OR 'Lao PDR*' OR 'Laos*' OR 'Lesotho*' OR 'Liberia*' OR 'Madagascar*' OR 'Malaw*' OR 'Mali' OR 'Mauritan*' OR 'Mauriti*' OR 'Micronesi*' OR 'Mocambiqu*' OR 'Moldov*' OR 'Mongolia*' OR 'Morocc*' OR 'Mozambiqu*' OR 'Myanmar*' OR 'Namibia*' OR 'Nepal*' OR 'Nicaragua*' OR 'Niger*' OR 'North Korea*' OR 'Northern Korea*' OR 'Democratic People's Republic of Korea' OR 'Pakistan*' OR 'Papua New Guinea*' OR 'Philippine*' OR 'Principe' OR 'Rhodesia*' OR 'Rwanda*' OR 'Samoa*' OR 'Sao Tome*' OR 'Senegal*' OR 'Sierra Leone*' OR 'Solomon Islands*' OR 'Somalia*' OR 'South Africa*' OR 'South Sudan*' OR 'Southern Africa*' OR 'Sri Lanka*' OR 'Sub Saharan Africa*' OR 'Subsaharan Africa*' OR 'Sudan*' OR 'Swaziland*' OR 'Syria*' OR 'Tajikist*' OR 'Tanzan*' OR 'Timor*' OR 'Togo*' OR 'Tonga*' OR 'Tunis*' OR 'Ugand*' OR 'Ukrain*' OR 'Uzbekistan*' OR 'Vanuatu*' OR 'Vietnam*' OR 'West Africa*' OR 'West Bank*' OR 'Western Africa*' OR 'Yemen*' OR 'Zaire*' OR 'Zambia*' OR 'Zimbabw*'

AND
'Umbilical Arter*' OR 'Uterine Artery' OR 'Middle Cerebral Artery' OR 'Ductus Venosus' OR 'Umbilical Vein*' OR 'Inferior Cava Vein' OR 'Uterine Arter*' OR 'Middle Cerebral Arter*' OR 'Patent Ductus Venosus' OR 'Inferior Vena Cava' OR 'Cerebroplacental Ratio' OR 'CPR' OR 'Fetal Descending Aorta' OR 'FDA' OR 'Doppler Ultrasonography' OR 'Doppler Ultrasound*' OR 'Doppler Ultrasonography' OR 'Uterine Artery Doppler'

AND

'Stillbirth' OR 'Perinatal Death' OR 'Cesarean Section*' OR 'Caesarean Section*' OR 'Acidosis' OR 'Premature Birth' OR 'Neonatal Intensive Care' OR 'Fetal Growth Retard*' OR 'Newborn Respiratory Distress Syndrome*' OR 'Gestational Age' OR 'Birth Weight' OR 'Asphyxia Neonatorum' OR 'Apgar Score*' OR 'Perinatal Mortality' OR 'Cesarean Section' OR 'Prematurity' OR 'Newborn Intensive Care' OR 'Intrauterine Growth Retardation' OR 'Neonatal Respiratory Distress Syndrome' OR 'Gestational Age' OR 'Birth Weight' OR 'Newborn Hypoxia' OR 'Length of Stay' OR 'Pregnancy' OR 'Pregnancies' OR 'Gestation' OR 'Pregnant'

SCOPUS

TITLE-ABS-KEY(“developing countr*” OR “developing nation*” OR “developing population*” OR “developing econom*” OR “undeveloped countr*” OR “undeveloped nation*” OR “undeveloped economy” OR “undeveloped economies” OR “least developed countr*” OR “least developed nation*” OR “least developed economy” OR “least developed economies” OR “less-developed countr*” OR “less-developed nation*” OR “less-developed population” OR “less-developed populations” OR “less-developed econom*” OR “lesser developed countr*” OR “lesser developed nation*” OR “lesser developed population” OR “lesser developed populations” OR “lesser developed economy” OR “lesser developed economies” OR “under-developed countr*” OR “under-developed nation*” OR “underdeveloped countr*” OR “underdeveloped nation*” OR “underdeveloped population*” OR “underdeveloped econom*” OR “low income countr*” OR “middle income countr*” OR “low income nation*” OR “middle income nation*” OR “low income population*” OR “middle income population*” OR “low income econom*” OR “middle income econom*” OR “lower income countr*” OR “lower income nation*” OR “lower income population*” OR “lower income economy” OR “lower income economies” OR “resource limited” OR “low resource countr*” OR “lower resource countr*” OR “low resource nation*” OR “low resource population*” OR “low resource economy” OR “low resource economies” OR “underserved countr*” OR “underserved nation*” OR “underserved population*” OR “underserved economy” OR “underserved economies” OR “under-served country” OR “under-served countries” OR “under-served nation” OR “under-served nations” OR “under-served population” OR “under-served populations” OR “underserved economy” OR “underserved economies” OR “derived countr*” OR “deprived nation” OR “deprived nations” OR “derived population*” OR “deprived economy” OR “deprived economies” OR “poor countr*” OR “poor nation*” OR “poor population*” OR “poor econom*” OR “poorer countr*” OR “poorer nation*” OR “poorer population*” OR “poorer econom*” OR “lmic” OR “lmics” OR “lami” OR “transitional countr*” OR “transitional nation” OR “transitional nations” OR “transitional econom*” OR “transition countr*” OR “transition nation*” OR “transition econom*” OR low “resource setting*” OR “lower resource setting*” OR “middle resource setting*” OR “Third World*” OR “south east asia*” OR “middle east*” OR “Afghan*” OR “Angola*” OR “Angolese*” OR “Angolian*” OR “Armenia*” OR “Bangladesh*” OR “Benin*” OR “Bhutan*” OR “Birma*” OR “Burma*” OR “Birmese*” OR “Burmese*” OR “Boliv*” OR “Botswan*” OR “burkina Faso*” OR “Burundi*” OR “Cabo Verde*” OR “Cambod*” OR “Cameroon*” OR “Cape Verd*” OR “Central Africa*” OR “Chad” OR “Comoro*” OR “Congo*” OR “Cote d/Ivoire*” OR “Djibouti*” OR “East Africa*” OR “Eastern Africa*” OR “Egypt*” OR “El Salvador*” OR “Equatorial Guinea*” OR “Eritre*” OR “Ethiopia*” OR “Gabon*” OR “Gambia*” OR “Gaza*” OR “Georgia Republic” OR “Ghan*” OR “Guatemal*” OR “Guinea” OR “Haiti*” OR “Hondur*” OR “India*” OR “Indones*” OR “Ivory Coast*” OR “Kenya*” OR “Kiribati*” OR “Kosovo*” OR “Kyrgyz*” OR “Lao PDR*” OR “Laos*” OR “Lesotho*” OR “Liberia*” OR “Madagascar*” OR “Malaw*” OR “Mali” OR “Mauritan*” OR “Mauriti*” OR “Micronesi*” OR “Mocambiqu*” OR “Moldov*” OR “Mongolia*” OR “Morocc*” OR “Mozambiqu*” OR “Myanmar*” OR “Namibia*” OR “Nepal*” OR “Nicaragua*” OR “Niger*” OR “North Korea*” OR “Northern Korea*” OR “Democratic People/s Republic of Korea” OR “Pakistan*” OR “Papua New Guinea*” OR “Philippine*” OR “Principe” OR “Rhodesia*” OR “Rwanda*” OR “Samoa*” OR “Sao Tome*” OR “Senegal*” OR “Sierra Leone*” OR “Solomon Islands*” OR “Somalia*” OR “South Africa*” OR “South Sudan*” OR “Southern Africa*” OR “Sri Lanka*” OR “Sub Saharan Africa*” OR “Subsaharan Africa*” OR “Sudan*” OR “Swaziland*” OR “Syria*” OR “Tajikist*” OR “Tanzan*” OR “Timor*” OR “Togo*” OR “Tonga*” OR “Tunis*” OR “Ugand*” OR “Ukrain*” OR “Uzbekistan*” OR “Vanuatu*” OR “Vietnam*” OR “West Africa*” OR “West Bank*” OR “Western Africa*” OR “Yemen*” OR “Zaire*” OR “Zambia*” OR “Zimbabw*”)

AND

TITLE-ABS-KEY("Stillbirth" OR "Perinatal Death" OR "Cesarean Section*" OR "Caesarean Section*" OR “Acidosis” OR “Premature Birth” OR “Neonatal Intensive Care" OR “Fetal Growth Retard*” OR “Newborn Respiratory Distress Syndrome*” OR “Gestational Age” OR “Birth Weight” OR “Asphyxia Neonatorum” OR “Apgar Score*” OR “Length of Stay" OR "Stillbirth" OR "Perinatal Death" OR "Cesarean Section" OR "Acidosis" OR "Premature Birth" OR "Intensive Care, Neonatal" OR "Fetal Growth Retardation" OR "Respiratory Distress Syndrome, Newborn" OR "Gestational Age" OR "Birth Weight" OR "Asphyxia Neonatorum" OR "Apgar Score" OR "Length of Stay" OR “Pregnancy” OR “Pregnancies” OR “Gestation” OR “Pregnant” OR "Pregnancy")

AND

TITLE-ABS-KEY("Umbilical Arteries" OR "Uterine Artery" OR "Middle Cerebral Artery" OR "Ductus Venosus" OR "Umbilical Veins" OR "Vena Cava, Inferior" OR “Umbilical Arter*” OR “Uterine Arter*” OR “Middle Cerebral Arter*” OR “Patent Ductus Venosus” OR “Umbilical Vein*” OR “Inferior Vena Cava” OR “Cerebroplacental Ratio” OR “CPR” OR “Fetal Descending Aorta” OR “FDA” OR "Ultrasonography, Doppler" OR “Doppler Ultrasound*”OR” Doppler Ultrasonography” OR “Uterine Artery Doppler”)
