# Supplementary material for: Prognostic accuracy of antenatal Doppler ultrasound for adverse perinatal outcomes in low-income and middle-income countries: a systematic review
Source: BMJ Open. 2021 Dec 2;11(12):e049799. doi: 10.1136/bmjopen-2021-049799 (PMC8640672; doi:10.1136/bmjopen-2021-049799)
Supplement: Supplementary data [file bmjopen-2021-049799supp005.pdf]

Table S1. Statistical measures of prognostic performance of Doppler ultrasound reported in the selected studies

Prognostic determinant	Outcome	Studies	Sn	Sp	PPV	NPV	AUROC	Diagnostic accuracy	OR [95% CI]	RR [95% CI]	Correlation	Normal Doppler n (%)	Abnormal Doppler n (%)
UA flow impedance	FGR	Agbaje et al., 2018	67.00	53.00			0.63						
		Mullick et al., 1993	85.00	89.00	88.50								
		Najam et al., 2016	48.15	80.67	53.06	77.40							
		Rocca et al., 1995	92.30	91.90	77.40	97.60		92.0					
		Khanduri et al., 2013	73.80	75.90	87.70	55.40		75.00					
		Bano et al., 2010	46.70	93.30	87.50	63.60		70.00					
		Nagar et al., 2015	42.86	94.62	37.50	95.65							
	NICU Admission	Anshul et al., 2010										13 (24.07)	36 (78.2)
		Najam et al., 2016	50.00	80.30	48.90	80.95							
	Fetal Distress	Anshul et al., 2010										18 (33)	35 (76)
		Rocca et al., 1995										2 (2.5)	12 (39)
		Najam et al., 2016	66.67	78.04	74.89	89.72							
		Yelikar et al., 2013	42.10	65.90	12.10	91.10							
	Stillbirth	Anshul et al., 2010										0 (0)	4 (9.5)
		Najam et al., 2016										0 (0)	5 (8.2)
	Perinatal death	Rocca et al., 1995										0 (0)	2 (6.5)
		Anshul et al., 2010										0 (0)	9 (60)
	LBW	Anshul et al., 2010										15 (27.0)	35 (77.8)
	Apgar Score	Rocca et al., 1995	80.00	82.40	41.00	96.00		83.00					
		Anshul et al., 2010										2 (3.7)	14 (82.35)
		Najam et al., 2016										3 (60.0)	6 (85.71)
		Agbaje et al., 2018									0.378		
	Fetal Anemia	Kumari et al., 2019									0.21		
	HIE	Najam et al., 2016										1 (1.29)	8 (16.31)
	MAS	Najam et al., 2016										1 (1.29)	16 (32.65)
	CAPO	Bano et al., 2010	79.20	92.40	79.20	92.20		88.90					
		Lakhkar et al 2006	50.00	59.00	66.60	41.90							

		Rani et al., 2016	17.80	95.80	80.70	50.50	0.57						
		Geerts et al., 2007	75.00			95.00			0.6 (0.1, 4.1)				
		Malik et al., 2013	64.40	80.00	96.60	20.00							
		Pattinson et al., 1993	12.50	91.80	22.70	84.50							
		Ebrashy et al., 2005	53.30	36.40	81.10	30.80							
		Waa et al., 2010	8.00	100.00	0.00	26.00							
**UA AREDF**	Perinatal death	Lakshmi et al., 2013							9.8 (2.1, 46.4)				
		Najam et al., 2016										2 (2.59)	4 (33.33)
	RDS	Lakshmi et al., 2013							2.4 (1.1, 5.0)				
	CAPO	Pattinson et al., 1991	75.00	90.00	69.00								
		Lakshmi et al., 2013							8.4 (2.3, 30.5)				
**MCA flow impedance**	FGR	Najam et al., 2016	59.25	88.89	72.72	81.35							
		Bano et al., 2010	8.90	100.0	100.0	52.30		54.40					
		Khanduri et al., 2013	26.20	92.60	89.20	35.00		46.10					
	Fetal Anemia	Pares et al., 2008	100.00	65.00	90.90	100.0		92.20					
		Kumari et al., 2019	68.00	57.00	83.00	33.00	0.70				-0.43		
	NICU Admission	Najam et al., 2016	64.58	88.69	70.45	85.71							
	Neonatal Acidosis	Allam et al., 2013	87.50	64.00	74.00	82.00	0.82						
	Fetal Distress	Najam et al., 2016	72.73	78.05	54.55	91.53							
	Stillbirth	Najam et al., 2016										0 (0)	2 (4.5)
	Apgar Score	Najam et al., 2016										1 (1.29)	17 (38.6)
	HIE	Najam et al., 2016										1 (1.29)	10 (22.72)
	MAS	Najam et al., 2016										1 (1.29)	20 (45.5)
	CAPO	Bano et al., 2010	16.70	100.0	100.0	76.70		77.80					
		Lakhkar et al 2006	41.60	90.90	88.20	48.70							
		Rani et al., 2016	18.60	90.30	68.70	49.40	0.58						
		Dhand et al., 2011	71.00	92.00	94.00	65.00							
		Malik et al., 2013	7.70	90.00	87.50	9.80							
		Ebrashy et al., 2005	41.00	63.60	80.00	23.30							
		Waa et al., 2010	23.0	68.00	76.00	33.00							

**CPR**	FGR	Najam et al., 2016	85.10	89.72	80.70	92.30							
		Bano et al., 2010						72.20					
	NICU Admission	Najam et al., 2016	75.00	82.92	63.15	89.47							
		Alanwar et al., 2018	62.50	71.42	29.40	90.90							
	Foetal Distress	Najam et al., 2016	90.91	78.04	52.63	96.97							
		Masihi et al.2019	80.95	50.00	17.50	95.20							
	Stillbirth	Najam et al., 2016										0 (0)	4 (7.14)
	Apgar Score	Najam et al., 2016										1 (1.29)	19 (33.33)
		Alanwar et al., 2018	50.0	88.10	44.40	90.20							
	Neonatal Acidosis	Ebrashy et al., 2005	64.10	72.70	89.30	36.40				1.4 (1.2, 1.7)			
		Alanwar et al., 2018	43.75	69.05	21.21	86.57							
	HIE	Najam et al., 2016										1 (1.29)	12 (21.05)
	MAS	Najam et al., 2016	96.15			99.20						1 (1.29)	25 (43.85)
	CAPO	Bano et al., 2010	83.30	100.0	100.00	94.30		95.60					
		Lakhkar et al 2006	47.20	86.30	85.00	50.00							
		Rani et al., 2016	7.60	98.00	81.80	48.30	0.60						
		Malik et al., 2013	68.80	100.00	100.0	26.30							
		Geerts et al., 2007			57.0				1.1 (0.1, 14.6)				
**UtA flow impedance**	FGR	Verma et al., 2016	45.0	84.10	28.10	91.70							
		Phupong et al., 2003	67.0	82.90	6.90	99.20				9.1 (1.7, 48.5)			
		Nagar et al., 2015	25.0	94.56	28.57	93.55							
	Perinatal Death	Dorman et al., 2002								2.37 (1.3, 4.3)			
	LBW	Verma et al., 2016	45.40	84.60	31.30	90.90							
		Dorman et al., 2002								2.52 (1.5, 4.2)			
	Preterm Birth	Verma et al., 2016	57.10	63.20	18.50	91.00							
		Dorman et al., 2002								1.53 (0.9, 2.4)			

	CAPO	Verma et al., 2016	48.20	95.40	84.40	78.20							
		Nouh et al., 2011	84.60	96.30	91.70	92.90							
		Malik et al., 2013	37.70	70.00	91.80	11.00							
		Zarean et al., 2018	37.50	73.30	48.40	63.70	0.55						
**FDA flow impedance**	Fetal anemia	Pares et al., 2008	95.70	100.0	100.0	86.90		96.70					
		Kumari et al., 2019	87.00	57.00			0.80				-0.54		
	CAPO	Lakhkar et al 2006	44.40	59.00	64.00	56.50							
**FDA & MCA**	Fetal anemia	Pares et al., 2008	98.40	100.0	100.0	91.70		98.60					
		Kumari et al., 2019	86.00	67.00	86.00	67.00							
**DV flow impedance**	Neonatal Acidosis	Allam et al., 2013	100.0	57.00	72.0	100.0	0.88	80.00					
	CAPO	Geerts et al., 2007		92.0	33.0				0.3 (0.03, 4.6)				

[a]UA: umbilical artery; MCA: middle cerebral artery; CPR: cerebroplacental ratio; UtA: uterine artery; FDA: fetal descending aorta; DV: ductus venosus; RI: resistive index; PI: pulsatility index; S/D ratio: systolic diastolic ratio; PSV: peak systolic velocity; MV: mean velocity; AREDF: absent and/or reversed end diastolic flow; FGR: fetal growth restriction; LBW: low birth weight; HIE: hypoxic ischemic encephalopathy; MAS: meconium aspiration syndrome; RDS: respiratory distress syndrome; NICU: neonatal intensive care unit; CAPO: composite adverse perinatal outcomes; Sn: sensitivity; Sp: specificity; PPV: positive predictive value; NPV: negative predictive value; OR: odds ratio; RR: relative risk; and n (%): frequency (percentage).
