# Supplementary material for: Prognostic accuracy of antenatal Doppler ultrasound for adverse perinatal outcomes in low-income and middle-income countries: a systematic review
Source: BMJ Open. 2021 Dec 2;11(12):e049799. doi: 10.1136/bmjopen-2021-049799 (PMC8640672; doi:10.1136/bmjopen-2021-049799)
Supplement: Supplementary data [file bmjopen-2021-049799supp002.pdf]

Appendix S2. List of full-text articles excluded with reasons

a) Country income level: 3 studies

1. El Shourbagy, S., Elsakhawy, M. (2012). Prediction of fetal anemia by middle cerebral artery Doppler. *Middle East Fertility Society Journal, 17(4), 275-282.*
2. Haley, J., Tuffnell, D. J., Johnson, N. (1997). Randomized controlled trial of cardiotocography versus umbilical artery Doppler in the management of small for gestational age fetuses. *British Journal of Obstetrics and Gynaecology, 104(4), 431-435).*
3. Morales-Rosello, J., Dias, T., Khalil, A., Fornes-Ferrer, V., Ciammella, R., Gimenez-Roca, L., Perales-Marin, A., Thilaganathan, B. (2018). Birth-weight differences at term are explained by placental dysfunction and not by maternal ethnicity. *Ultrasound Obstet Gynecol, 52(4), 488-493.*

b) Design and quality: 9 studies

1. Abidoye, I. A., Ayoola, O. O., Idowu, B., Aderibigbe, A. S., Loto, O. M. (2017). Uterine artery Doppler velocimetry in hypertensive disorder of pregnancy in Nigeria. *J Ultrason, 17(71)) 253-258.*
2. Agarwal, R., Tiwari, A., Wadhwa, N., Radhakrishnan, G., Bhatt, S., Batra, P. (2017). Abnormal umbilical artery Doppler velocimetry and placental histopathological correlation in fetal growth restriction. *South African Journal of Obstetrics and Gynaecology, 23(1), 12-16.*
3. Ali, A., Ara, I., Sultana, R., Akram, F., Zaib, M. J. (2014). Comparison of perinatal outcome of growth restricted fetuses with normal and abnormal umbilical artery Doppler waveforms. *Journal of Ayub Medical College, Abottabad: JAMC, 26(3), 344-348.*
4. Kumar, S., Datta, S., Mittal, S., Roy, K. K. (2002). Doppler flow studies in middle cerebral and umbilical arteries in growth retarded and normal pregnancies. *JK Science, 4(0), 185-189*
5. Mufenda, J., Gebhardt, S., van Rooyen, R., Theron, G. (2015). Introducing a Mobile-Connected Umbilical Doppler Device (UmbiFlow) into a Primary Care Maternity Setting: Does This Reduce Unnecessary Referrals to Specialised Care? Results of a Pilot Study in Kraaifontein, South Africa. *PLoS One, 10(11) e0142743.*
6. Nguku, S. W., Wanyoike-Gichuhi, J., Aywak, A. A. (2006). Biophysical profile scores and resistance indices of the umbilical artery as seen in patients with pregnancy induced hypertension. *East African Medical Journal, 83(3), 96-101*
7. Nkosi, S., Makin, J., Hlongwane, T. M. A. G., & Pattinson, R. C. (2019). Screening and managing a low-risk pregnant population using continuous-wave Doppler ultrasound in a low-income population: A cohort analytical study. *SAMJ: South African Medical Journal*, *109*(5), 347-352.
8. Siddiqui, T. S., Asim, A., Ali, S., Tariq, A. (2014). Comparison of perinatal outcome in growth restricted fetuses retaining normal umbilical artery Doppler flow to those with diminished end-diastolic flow. *Journal of Ayub Medical College, Abbottabad: JAMC, 26(2), 221-224.*
9. Kachewar, S. G., Gandage, S. G., Pawar, H. J. (2012). An Indian study of novel non-invasive method of screening for foetal anaemia. *Journal of Clinical and Diagnostic Research, 6(4), 688-691.*

c) Outcomes: 11 studies

1. Adekanmi, A. J., Roberts, A., Akinmoladun, J. A., & Adeyinka, A. O. (2019). Uterine and umbilical artery doppler in women with pre-eclampsia and their pregnancy outcomes. *Nigerian Postgraduate Medical Journal*, *26*(2), 106.
2. El Behery, M. M., Siam, S., Seksaka, M. A., Mansou, S. M. (2013). Uterine artery Doppler and urinary hyperglycosylated HCG as predictors of threatened abortion outcome. *Middle East Fertility Society Journal, 19(1), 42-46.*
3. El-Mashad, A. I., Mohamed, M. A., Elahadi Farag, M. A., Ahmad, M. K., Ismail, Y. (2011). Role of uterine artery Doppler velocimetry indices and plasma adrenomedullin level in women with unexplained recurrent pregnancy loss. *Journal of Obstetrics and Gynaecology Research, 37(1), 51-57.*
4. Geerts, L., Van der Merwe, E., Theron, A., Rademan, K. (2016). Placental insufficiency among high-risk pregnancies with a normal umbilical artery resistance index after 32 weeks. *Int J Gynaecol Obstet, 135(1), 38-42.*
5. Kumar, B. S., Sarmila, K., Prasad, K. S. (2012). Prediction of preeclampsia by midtrimester uterine artery doppler velocimetry in high-risk and low-risk women. *Journal of Obstetrics and Gynecology of India, 62(3), 297-300.*
6. Maged. A. M., Elnassery, N., Fouad, M., Abdelhafiz, A., Al Mostafa, W. (2015). Third-trimester uterine artery Doppler measurement and maternal postpartum outcome among patients with severe pre-eclampsia. *International Journal of Gynecology and Obstetrics, 131(1), 49-53.*
7. Prajapati, S. R., Maitra, N. (2013). Prediction of pre-eclampsia by a combination of history, uterine artery doppler, and mean arterial pressure (A Prospective Study of 200 Cases). *Journal of Obstetrics and Gynecology of India, 63(1), 32-36.*
8. Sebastian, A., Raj, T. S., Yenuberi, H., Job, V., Varuhghese, S., & Regi, A. (2019). Angiogenic factors and uterine artery Doppler in predicting preeclampsia and associated adverse outcomes in a tertiary hospital in south India. *Pregnancy hypertension*, *16*, 26.
9. Shehata, N. A. A., Ali, H. A. A., Hassan, A., Katta, M. A., Ali, A. S. F. (2018). Doppler and biochemical assessment for the prediction of early pregnancy outcome in patients experiencing threatened spontaneous abortion. *Int J Gynaecol Obstet, 143(2), 150-155.*
10. Yusuf, M., Galadanci, H., Ismail, A., Aliyu, L. D., Danbatta, A. H. (2017). Uterine artery doppler velocimetry for the prediction of preeclampsia among high-risk pregnancies in low-resource setting: Our experience at aminu Kano teaching hospital, Kano, Nigeria. *Donald School Journal of Ultrasound in Obstetrics and Gynecology, 11(3), 197-202*
11. Puri, M. S., Deshpande, H., Kohli, S., Sharma, K., Singhania, S. (2013). A study of uterine artery colour doppler at 20-24 weeks gestation as a predictor of pregnancy induced hypertension and intra uterine growth restriction from industrial town in Western India. *Research Journal of Pharmaceutical, Biological and Chemical Sciences, 4(1), 698-705.*
