# Supplementary material for: Prognostic accuracy of antenatal Doppler ultrasound for adverse perinatal outcomes in low-income and middle-income countries: a systematic review
Source: BMJ Open. 2021 Dec 2;11(12):e049799. doi: 10.1136/bmjopen-2021-049799 (PMC8640672; doi:10.1136/bmjopen-2021-049799)
Supplement: Supplementary data [file bmjopen-2021-049799supp003.pdf]

Appendix S3. The aims of the selected studies and risk profiles of the women recruited

First Author	Aim of study	Dating method	Risk Profile	Participant risk profile details in the article
Abdallah et al., 2019	To study the value of umbilical artery Doppler indices in predicting the risk of intrapartum and neonatal outcomes in pregnancies with and without nuchal cord.	LMP or first trimester ultrasound	Low risk	Primigravida >=37 weeks admitted in labor to the delivery unit. Women with BMI >30 kg/m2, multiple pregnancy, fetal malpresentation, fetal demise, chorioamnionitis, meconium-stained liquor, associated medical disorder (hypertension, diabetes, autoimmune disease, etc.), perinatal complication (e.g. placental abruption), fetal malformation or abnormal fetal growth were excluded from the study.
Agbaje et al., 2018	To assess umbilical artery Doppler findings in women with sickle cell anemia in the local environment at the onset of the third trimester and compare with obstetric outcomes.	LMP and/or early dating sonograms	High-risk	Sickle cell anemia.
Alanwar et al., 2018	To assess the efficacy of fetal middle cerebral artery/umbilical artery pulsatility index ratio (cerebroplacental ratio CPR) in predicting the occurrence of adverse perinatal outcomes in pregnancies complicated with severe pre-eclampsia.	Not specified	High-risk	Pregnancies complicated with severe pre-eclampsia.
Allam et al., 2013	To investigate, in high-risk pregnancies, the prediction of neonatal acidosis using DV, MCA and UA Doppler studies and subsequently to determine the best parameters and cutoff values.	Not specified	High-risk	Suspected IUGR, oligohydramnios, preeclampsia, or placental vascular dysfunction documented by abnormal umbilical artery pulsatility index by local reference ranges.
Anshul et al., 2010	To evaluate the role of umbilical artery Doppler in growth-restricted fetuses.	LMP and first trimester dating scan	High-risk	SGA foetuses, some mothers had hypertensive disorder, anemia, bad obstetric history
Bano et al., 2010	To evaluate the usefulness of the pulsatility index (PI) of the umbilical artery (UA) and that of the middle cerebral artery (MCA), as well as the ratio of the MCA PI to the UA PI (C/U ratio), in the diagnosis of small-for-gestational-age (SGA) fetuses and the prediction of adverse perinatal outcome.	Not specified	High risk	Clinical suspicion of FGR

Dhand et al., 2011	To compare the role of the middle cerebral artery and umbilical artery Doppler pulsatility indices in predicting the fetal outcome in intrauterine growth restriction.	LMP and fetal biometry <22weeks	High risk	SGA fetuses
Dorman et al., 2002	To determine whether impaired uteroplacental blood flow might account for the low infant birth weight associated with maternal falciparum malaria infection.	LMP and fetal biometry	High-risk	Maternal falciparum malaria infection.
Ebrashy et al., 2005	To evaluate the accuracy of middle cerebral/umbilical artery resistance index (C/U RI) ratio in predicting acidemia and low Apgar score at 5 minutes after birth in the infants of women with preeclampsia.	Fetal biometry (BPD, AC and FL)	High-risk	Pre-eclampsia women
Geerts et al., 2007	To assess the prognostic value of ultrasound findings and fetoplacental Doppler indices in severe preterm preeclampsia in identifying fetuses at high risk of death, major morbidity or long-term compromise.	LMP and fetal biometry	High-risk	Women with severe pre-eclampsia
Khanduri et al., 2013	To measure the pulsatility index (PI) and resistive index (RI) of the middle cerebral artery (MCA) and umbilical artery (UA) in predicting fetal growth restriction.	LMP and first or second trimester ultrasound	High-risk	Clinical suspicion of FGR
Kumari et al., 2019	To assess the correlation between fetal blood vessel Doppler measurements and fetal anemia among Rhesus isoimmunized pregnancies after two intrauterine transfusions as a potential guide to therapy.	Not specified	High risk	Rhesus isoimmunized complicated pregnancies
Lakhkar et al., 2006	To determine and compare the diagnostic performance of Doppler sonography of fetal middle cerebral artery (MCA), descending abdominal aorta (DAA), umbilical artery (UA), umbilical vein (UV) and inferior vena cava (IVC) for prediction of adverse perinatal outcome in suspected intrauterine growth retardation (IUGR) and pre-eclampsia (PET).	LMP, clinical gestational age, 1st or 2nd trimester biometry	High risk	Preeclampsia and suspicion of growth-restricted fetuses

Lakshmi et al., 2013	To determine outcomes of preterm infants with history of absent/reversed end-diastolic umbilical artery Doppler flow (AREDF) vs. infants with forward end-diastolic flow (FEDF).	LMP or first trimester ultrasound	High-risk	FGR, pregnancy induced hypertension, h/o previous intrauterine death
Malik et al., 2013	To determine the role of ultrasonography in screening high-risk mothers for detection of IUGR, to find out the impact of fetal parameters on the extent of IUGR, correlation between the sonographic pattern of IUGR and the birth weight, and to find out the sensitivities of various fetal parameters and their evaluation against each other and against the birth weight.	LMP	High-risk	FGR; hypertensive disorder; pre-eclampsia
Masihi et al.2019	To determine the relationship between the fetal middle cerebral artery and the umbilical artery ratio on color Doppler sonography with fetal distress at 38-40 weeks of gestation.	First trimester ultrasound	Low risk	Women that had uncomplicated pregnancies
Mullick et al., 1993	To explore whether measurement of umbilical artery blood velocity waveform between 22 and 26 weeks might predict pregnancies destined to become complicated by pregnancy could induce hypertension (PIH) and/or fetal growth restriction (IUGR).	Not specified	Low and high-risk	Women attending routine antenatal (any risk profile).
Nagar et al., 2015	To evaluate the predictive values of Uterine and Umbilical artery Doppler indices in high-risk pregnancies.	LMP and ultrasound before 21 weeks	High risk	History of preeclampsia or eclampsia in previous pregnancy pre-existing medical disorders like: Diabetes, Renal disease, Epilepsy, Autoimmune disease, Thrombophilia, and Hypertension, History of IUGR or still birth, history of abruptio placentae, preeclampsia or pregnancy-induced hypertension current, Nulliparity, Extremes of age (<20 years and >35 years).

Najam et al., 2016	To assess the predictive value of the cerebroplacental ratio in the detection of perinatal outcome in high-risk pregnancies in comparison to its components.	Not specified	Low and high-risk	Pregnancies undergoing routine antenatal (any risk profile).
Nouh et al., 2011	To assess the value of uterine artery Doppler screening during pregnancy in predicting adverse pregnancy outcomes in women with polycystic ovary syndrome (PCOS).	LMP and first trimester ultrasound	High-risk	Primigravida with ovulatory polycystic ovary syndrome (PCOS)
Pares et al., 2008	To evaluate the accuracy of middle cerebral artery peak systolic velocity (MCA-PSV) associated with descending thoracic aorta mean velocity (DTA-MV) in the prediction of fetal anemia.	Sonographic exam at <= 20 weeks	High-risk	Fetuses at risk for anemia because of maternal alloimmunization to red-cell antigens
Pattinson et al., 1991	To investigate whether abnormalities in Doppler waveform can predict the outcome of pregnancy accurately before other clinical signs develop	LMP and biometry: 16-20 weeks	High risk	SGA, preeclampsia and pregnancy wastage
Pattinson et al., 1993	To describe the prevalence and natural history of absent end-diastolic velocities (AEDV) in the umbilical artery of the fetus between 16 and 24 weeks gestation, and to evaluate its role as a screening test for identifying high-risk pregnancies.	Not specified	Low and high-risk	Pregnancies undergoing routine antenatal (any risk profile).
Phupong et al., 2003	To assess the value of uterine artery notching as a screening test for preeclampsia and fetal growth restriction in a low-risk population of healthy pregnant women.	LMP and first trimester ultrasound	Low-risk	Healthy pregnant women
Rani et al., 2016	To assess the accuracy of the middle cerebral artery (MCA) and umbilical artery (UmA), pulsatility index (PI) and resistance index (RI) in predicting perinatal outcome in pregnancies complicated by preeclampsia with or without intrauterine growth restriction (IUGR).	Not specified	Low and high-risk	Women attending routine antenatal (any risk profile).

Rocca et al., 1995	To test the value of routine Doppler study of the umbilical artery to predict the perinatal outcome in pre-eclamptic patients.	Not specified	High risk	Pre-eclampsia women
Verma et al., 2016	To assess the predictive value of uterine artery Doppler imaging at 22-24 weeks of gestation for adverse pregnancy outcomes.	Not specified	Low-risk	Women with uncomplicated pregnancies
Waa et al., 2010	To assess the value of umbilical and middle cerebral artery doppler ultrasound values in predicting foetal outcome in high and low-risk pregnancies.	Not specified	Low and high-risk	Women undergoing routine antenatal (any risk profile).
Yelikar et al., 2013	To study the efficacy of fetal Doppler and Non-Stress Test (NST) in predicting fetal compromise in preeclampsia and growth-restricted fetuses.	Not specified	High-risk	Preeclampsia and growth-restricted fetuses
Zarean et al., 2018	To assess the diagnostic value of UtA-PI in the prediction of the adverse perinatal outcome at 30–34 week's gestation.	Not specified	Low-risk	Women that had uncomplicated pregnancies

[a]FGR: fetal growth restriction; LBW: low birth weight; NICU: neonatal intensive care unit. High risk: pregnancies with any underlying condition that threatens the health or life of the mother or her foetus.

Any risk profile: unselected pregnancies (pregnancies undergoing routine antenatal). Low risk: Uncomplicated pregnancies or healthy pregnant women
