# Supplementary material for: Prognostic accuracy of antenatal Doppler ultrasound for adverse perinatal outcomes in low-income and middle-income countries: a systematic review
Source: BMJ Open. 2021 Dec 2;11(12):e049799. doi: 10.1136/bmjopen-2021-049799 (PMC8640672; doi:10.1136/bmjopen-2021-049799)
Supplement: Supplementary data [file bmjopen-2021-049799supp004.pdf]

Appendix S4. Risk of bias assessment results of the 30 studies included in the analysis

First Author: Abdallah et al., 2018 **ID:** 68614233

Potential Bias	Items to be considered for assessment of potential opportunity for bias	Yes	Partly	No	Unsure	NA*
Study population /sample selection	Inclusion and exclusion criteria are adequately described [including explicit diagnostic criteria, start/finish date of recruitment]	x				
	Baseline study sample [i.e., individuals entering the study and their key characteristics and sampling frame are adequately described]	x				
	Study sample represents population of interest on key characteristics, sufficient to limit potential bias to results	Low risk of bias				
Study attrition	Response rate (i.e., proportion of study sample completing the study and providing outcome data) is adequate				x	
	Participants lost to follow-up are adequately described for key characteristics				x	
	Statement as to the possible effect on the results from missing data				x	
	Loss to follow-up is not associated with key characteristics	Moderate risk of bias				
Prognostic factor measurement	Clear definition of the prognostic factors measured is provided (e.g imaging modality method, measurement, and timing described).	x				
	Specified instrument and personnel for measurement of predictive factors	x				
	Continuous variables are reported or appropriate (i.e. not data- dependent) cut-off points are used and specified a priori	x				
	Blinding: were estimators of risk factor status and of outcomes blinded?			x		
	The prognostic factor(s) of interest is (are) adequately measured in study participants to sufficiently limit potential bias	Low risk of bias				
Outcome measurement	Is the outcome(s) clearly defined?			x		
	The outcome measure and method used are adequately valid and reliable to limit misclassification bias	Moderate risk of bias				
Study confounding	Do the authors address potential confounders?	x				
	Important potential confounders are appropriately accounted for, limiting potential bias with respect to the prognostic factor of interest.	Low risk of bias				
Analysis and reporting	There is sufficient presentation of data to assess the adequacy of the analysis strategy and there is no selective reporting		x			
	The statistical analysis is appropriate for the study design, limiting potential for the presentation of invalid results	Moderate risk of bias				
NA*: not applicable. Note: The above table was adapted from: Hayden et al., 2013.						
**Overall opinion of study quality= Moderate risk of bias**						

First Author: Agbaje et al., 2018 **ID:** 6377433

Potential Bias	Items to be considered for assessment of potential opportunity for bias	Yes	Partly	No	Unsure	NA*
Study population /sample selection	Inclusion and exclusion criteria are adequately described [including explicit diagnostic criteria, start/finish date of recruitment]	x				
	Baseline study sample [i.e. individuals entering the study and their key characteristics and sampling frame are adequately described]		x			
	Study sample represents population of interest on key characteristics, sufficient to limit potential bias to results	Moderate risk of bias				
Study attrition	Response rate (i.e., proportion of study sample completing the study and providing outcome data) is adequate					x
	Participants lost to follow-up are adequately described for key characteristics					x
	Statement as to the possible effect on the results from missing data					x
	Loss to follow-up is not associated with key characteristics	Low risk of bias				
Prognostic factor measurement	Clear definition of the prognostic factors measured is provided (e.g. imaging modality method, measurement, and timing described).	x				
	Specified instrument and personnel for measurement of predictive factors	x				
	Continuous variables are reported or appropriate (i.e. not data- dependent) cut-off points are used and specified a priori	x				
	Blinding: were estimators of risk factor status and of outcomes blinded?		x			
	The prognostic factor(s) of interest is (are) adequately measured in study participants to sufficiently limit potential bias	Low risk of bias				
Outcome measurement	Is the outcome(s) clearly defined?		x			
	The outcome measure and method used are adequately valid and reliable to limit misclassification bias	Moderate risk of bias				
Study confounding	Do the authors address potential confounders?		x			
	Important potential confounders are appropriately accounted for, limiting potential bias with respect to the prognostic factor of interest.	Moderate risk of bias				
Analysis and reporting	There is sufficient presentation of data to assess the adequacy of the analysis strategy and there is no selective reporting		x			
	The statistical analysis is appropriate for the study design, limiting potential for the presentation of invalid results	Moderate risk of bias				
NA*: not applicable. Note: The above table was adapted from: Hayden et al., 2013.						
**Overall opinion of study quality= Moderate risk of bias**						

First Author: Alanwar et al., 2018 **ID:** 6377464

Potential Bias	Items to be considered for assessment of potential opportunity for bias	Yes	Partly	No	Unsure	NA*
Study population /sample selection	Inclusion and exclusion criteria are adequately described [including explicit diagnostic criteria, start/finish date of recruitment]	x				
	Baseline study sample [i.e. individuals entering the study and their key characteristics and sampling frame are adequately described]	x				
	Study sample represents population of interest on key characteristics, sufficient to limit potential bias to results	Low risk of bias				
Study attrition	Response rate (i.e., proportion of study sample completing the study and providing outcome data) is adequate					x
	Participants lost to follow-up are adequately described for key characteristics					x
	Statement as to the possible effect on the results from missing data					x
	Loss to follow-up is not associated with key characteristics	Low risk of bias				
Prognostic factor measurement	Clear definition of the prognostic factors measured is provided (e.g. imaging modality method, measurement, and timing described).	x				
	Specified instrument and personnel for measurement of predictive factors	x				
	Continuous variables are reported or appropriate (i.e. not data- dependent) cut-off points are used and specified a priori	x				
	Blinding: were estimators of risk factor status and of outcomes blinded?				x	
	The prognostic factor(s) of interest is (are) adequately measured in study participants to sufficiently limit potential bias	Low risk of bias				
Outcome measurement	Is the outcome(s) clearly defined?	x				
	The outcome measure and method used are adequately valid and reliable to limit misclassification bias	Low risk of bias				
Study confounding	Do the authors address potential confounders?	x				
	Important potential confounders are appropriately accounted for, limiting potential bias with respect to the prognostic factor of interest.	Low risk of bias				
Analysis and reporting	There is sufficient presentation of data to assess the adequacy of the analysis strategy and there is no selective reporting	x				
	The statistical analysis is appropriate for the study design, limiting potential for the presentation of invalid results	Low risk of bias				
NA*: not applicable. Note: The above table was adapted from: Hayden et al., 2013.						
**Overall opinion of study quality= Low risk of bias**						

First Author: Allam et al., 2013 **ID:** 6377480

Potential Bias	Items to be considered for assessment of potential opportunity for bias	Yes	Partly	No	Unsure	NA*
Study population /sample selection	Inclusion and exclusion criteria are adequately described [including explicit diagnostic criteria, start/finish date of recruitment]		x			
	Baseline study sample [i.e. individuals entering the study and their key characteristics and sampling frame are adequately described]	x				
	Study sample represents population of interest on key characteristics, sufficient to limit potential bias to results	Low risk of bias				
Study attrition	Response rate (i.e., proportion of study sample completing the study and providing outcome data) is adequate	x				
	Participants lost to follow-up are adequately described for key characteristics				x	
	Statement as to the possible effect on the results from missing data					x
	Loss to follow-up is not associated with key characteristics	Low risk of bias				
Prognostic factor measurement	Clear definition of the prognostic factors measured is provided (e.g. imaging modality method, measurement, and timing described).	x				
	Specified instrument and personnel for measurement of predictive factors	x				
	Continuous variables are reported or appropriate (i.e. not data- dependent) cut-off points are used and specified a priori	x				
	Blinding: were estimators of risk factor status and of outcomes blinded?				x	
	The prognostic factor(s) of interest is (are) adequately measured in study participants to sufficiently limit potential bias	Low risk of bias				
Outcome measurement	Is the outcome(s) clearly defined?	x				
	The outcome measure and method used are adequately valid and reliable to limit misclassification bias	Low risk of bias				
Study confounding	Do the authors address potential confounders?		x			
	Important potential confounders are appropriately accounted for, limiting potential bias with respect to the prognostic factor of interest.	Moderate risk of bias				
Analysis and reporting	There is sufficient presentation of data to assess the adequacy of the analysis strategy and there is no selective reporting		x			
	The statistical analysis is appropriate for the study design, limiting potential for the presentation of invalid results	Moderate risk of bias				
NA*: not applicable. Note: The above table was adapted from: Hayden et al., 2013.						
**Overall opinion of study quality= Low risk of bias**						

First Author: Anshul et al., 2010 **ID:** 6377837

**Potential Bias**	**Items to be considered for assessment of potential opportunity for bias**	**Yes**	**Partly**	**No**	**Unsure**	**NA***
Study population /sample selection	Inclusion and exclusion criteria are adequately described [including explicit diagnostic criteria, start/finish date of recruitment]	x				
	Baseline study sample [i.e. individuals entering the study and their key characteristics and sampling frame are adequately described]	x				
	Study sample represents population of interest on key characteristics, sufficient to limit potential bias to results	Low risk of bias				
Study attrition	Response rate (i.e., proportion of study sample completing the study and providing outcome data) is adequate				x	
	Participants lost to follow-up are adequately described for key characteristics				x	
	Statement as to the possible effect on the results from missing data				x	
	Loss to follow-up is not associated with key characteristics	High risk of bias				
Prognostic factor measurement	Clear definition of the prognostic factors measured is provided (e.g. imaging modality method, measurement, and timing described).		x			
	Specified instrument and personnel for measurement of predictive factors			x		
	Continuous variables are reported or appropriate (i.e. not data- dependent) cut-off points are used and specified a priori	x				
	Blinding: were estimators of risk factor status and of outcomes blinded?					x
	The prognostic factor(s) of interest is (are) adequately measured in study participants to sufficiently limit potential bias	Moderate risk of bias				
Outcome measurement	Is the outcome(s) clearly defined?			x		
	The outcome measure and method used are adequately valid and reliable to limit misclassification bias	High risk of bias				
Study confounding	Do the authors address potential confounders?	x				
	Important potential confounders are appropriately accounted for, limiting potential bias with respect to the prognostic factor of interest.	Low risk of bias				
Analysis and reporting	There is sufficient presentation of data to assess the adequacy of the analysis strategy and there is no selective reporting			x		
	The statistical analysis is appropriate for the study design, limiting potential for the presentation of invalid results	High risk of bias				
NA*: not applicable. Note: The above table was adapted from: Hayden et al., 2013.						
**Overall opinion of study quality= High risk of bias**						

First Author: Bano et al., 2010 **ID:** 74903018

**Potential Bias**	**Items to be considered for assessment of potential opportunity for bias**	**Yes**	**Partly**	**No**	**Unsure**	**NA***
Study population /sample selection	Inclusion and exclusion criteria are adequately described [including explicit diagnostic criteria, start/finish date of recruitment]		x			
	Baseline study sample [i.e. individuals entering the study and their key characteristics and sampling frame are adequately described]			x		
	Study sample represents population of interest on key characteristics, sufficient to limit potential bias to results	Moderate risk of bias				
Study attrition	Response rate (i.e., proportion of study sample completing the study and providing outcome data) is adequate					x
	Participants lost to follow-up are adequately described for key characteristics					x
	Statement as to the possible effect on the results from missing data					x
	Loss to follow-up is not associated with key characteristics	Low risk of bias				
Prognostic factor measurement	Clear definition of the prognostic factors measured is provided (e.g. imaging modality method, measurement, and timing described).	x				
	Specified instrument and personnel for measurement of predictive factors			x		
	Continuous variables are reported or appropriate (i.e. not data- dependent) cut-off points are used and specified a priori	x				
	Blinding: were estimators of risk factor status and of outcomes blinded?			x		
	The prognostic factor(s) of interest is (are) adequately measured in study participants to sufficiently limit potential bias	Moderate risk of bias				
Outcome measurement	Is the outcome(s) clearly defined?			x		
	The outcome measure and method used are adequately valid and reliable to limit misclassification bias	High risk of bias				
Study confounding	Do the authors address potential confounders?			x		
	Important potential confounders are appropriately accounted for, limiting potential bias with respect to the prognostic factor of interest.	High risk of bias				
Analysis and reporting	There is sufficient presentation of data to assess the adequacy of the analysis strategy and there is no selective reporting		x			
	The statistical analysis is appropriate for the study design, limiting potential for the presentation of invalid results	Moderate risk of bias				
NA*: not applicable. Note: The above table was adapted from: Hayden et al., 2013.						
**Overall opinion of study quality= high risk of bias**						

First Author: Dhand et al., 2011 **ID:** 6379383

Potential Bias	Items to be considered for assessment of potential opportunity for bias	Yes	Partly	No	Unsure	NA*
Study population /sample selection	Inclusion and exclusion criteria are adequately described [including explicit diagnostic criteria, start/finish date of recruitment]	x				
	Baseline study sample [i.e. individuals entering the study and their key characteristics and sampling frame are adequately described]	x				
	Study sample represents population of interest on key characteristics, sufficient to limit potential bias to results	Low risk of bias				
Study attrition	Response rate (i.e., proportion of study sample completing the study and providing outcome data) is adequate				x	
	Participants lost to follow-up are adequately described for key characteristics				x	
	Statement as to the possible effect on the results from missing data				x	
	Loss to follow-up is not associated with key characteristics	Moderate risk of bias				
Prognostic factor measurement	Clear definition of the prognostic factors measured is provided (e.g. imaging modality method, measurement, and timing described).		x			
	Specified instrument and personnel for measurement of predictive factors		x			
	Continuous variables are reported or appropriate (i.e. not data- dependent) cut-off points are used and specified a priori		x			
	Blinding: were estimators of risk factor status and of outcomes blinded?				x	
	The prognostic factor(s) of interest is (are) adequately measured in study participants to sufficiently limit potential bias	High risk of bias				
Outcome measurement	Is the outcome(s) clearly defined?			x		
	The outcome measure and method used are adequately valid and reliable to limit misclassification bias	High risk of bias				
Study confounding	Do the authors address potential confounders?	x				
	Important potential confounders are appropriately accounted for, limiting potential bias with respect to the prognostic factor of interest.	Low risk of bias				
Analysis and reporting	There is sufficient presentation of data to assess the adequacy of the analysis strategy and there is no selective reporting			x		
	The statistical analysis is appropriate for the study design, limiting potential for the presentation of invalid results	High risk of bias				

NA*: not applicable. Note: The above table was adapted from: Hayden et al., 2013.

Overall opinion of study quality= High risk of bias

First Author: Dorman et al., 2002 **ID:** 6377862

**Potential Bias**	**Items to be considered for assessment of potential opportunity for bias**	**Yes**	**Partly**	**No**	**Unsure**	**NA***
Study population /sample selection	Inclusion and exclusion criteria are adequately described [including explicit diagnostic criteria, start/finish date of recruitment]	x				
	Baseline study sample [i.e. individuals entering the study and their key characteristics and sampling frame are adequately described]	x				
	Study sample represents population of interest on key characteristics, sufficient to limit potential bias to results	Low risk of bias				
Study attrition	Response rate (i.e., proportion of study sample completing the study and providing outcome data) is adequate	x				
	Participants lost to follow-up are adequately described for key characteristics	x				
	Statement as to the possible effect on the results from missing data	x				
	Loss to follow-up is not associated with key characteristics	Low risk of bias				
Prognostic factor measurement	Clear definition of the prognostic factors measured is provided (e.g. imaging modality method, measurement, and timing described).	x				
	Specified instrument and personnel for measurement of predictive factors	x				
	Continuous variables are reported or appropriate (i.e. not data- dependent) cut-off points are used and specified a priori	x				
	Blinding: were estimators of risk factor status and of outcomes blinded?	x				
	The prognostic factor(s) of interest is (are) adequately measured in study participants to sufficiently limit potential bias	Low risk of bias				
Outcome measurement	Is the outcome(s) clearly defined?	x				
	The outcome measure and method used are adequately valid and reliable to limit misclassification bias	Low risk of bias				
Study confounding	Do the authors address potential confounders?	x				
	Important potential confounders are appropriately accounted for, limiting potential bias with respect to the prognostic factor of interest.	Low risk of bias				
Analysis and reporting	There is sufficient presentation of data to assess the adequacy of the analysis strategy and there is no selective reporting	x				
	The statistical analysis is appropriate for the study design, limiting potential for the presentation of invalid results	Low risk of bias				
NA*: not applicable. Note: The above table was adapted from: Hayden et al., 2013.						
**Overall opinion of study quality= Low risk of bias**						

First Author: Ebrashy et al., 2005 **ID:** 6377887

**Potential Bias**	**Items to be considered for assessment of potential opportunity for bias**	**Yes**	**Partly**	**No**	**Unsure**	**NA***
Study population /sample selection	Inclusion and exclusion criteria are adequately described [including explicit diagnostic criteria, start/finish date of recruitment]	x				
	Baseline study sample [i.e. individuals entering the study and their key characteristics and sampling frame are adequately described]	x				
	Study sample represents population of interest on key characteristics, sufficient to limit potential bias to results	Low risk of bias				
Study attrition	Response rate (i.e., proportion of study sample completing the study and providing outcome data) is adequate	x				
	Participants lost to follow-up are adequately described for key characteristics	x				
	Statement as to the possible effect on the results from missing data					x
	Loss to follow-up is not associated with key characteristics	Low risk of bias				
Prognostic factor measurement	Clear definition of the prognostic factors measured is provided (e.g. imaging modality method, measurement, and timing described).	x				
	Specified instrument and personnel for measurement of predictive factors	x				
	Continuous variables are reported or appropriate (i.e. not data- dependent) cut-off points are used and specified a priori	x				
	Blinding: were estimators of risk factor status and of outcomes blinded?	x				
	The prognostic factor(s) of interest is (are) adequately measured in study participants to sufficiently limit potential bias	Low risk of bias				
Outcome measurement	Is the outcome(s) clearly defined?	x				
	The outcome measure and method used are adequately valid and reliable to limit misclassification bias	Low risk of bias				
Study confounding	Do the authors address potential confounders?	x				
	Important potential confounders are appropriately accounted for, limiting potential bias with respect to the prognostic factor of interest.	Low risk of bias				
Analysis and reporting	There is sufficient presentation of data to assess the adequacy of the analysis strategy and there is no selective reporting		x			
	The statistical analysis is appropriate for the study design, limiting potential for the presentation of invalid results	Moderate risk of bias				
NA*: not applicable. Note: The above table was adapted from: Hayden et al., 2013.						
**Overall opinion of study quality= Low risk of bias**						

First Author: Geerts et al., 2007 **ID:** 6378017

**Potential Bias**	**Items to be considered for assessment of potential opportunity for bias**	**Yes**	**Partly**	**No**	**Unsure**	**NA***
Study population /sample selection	Inclusion and exclusion criteria are adequately described [including explicit diagnostic criteria, start/finish date of recruitment]	x				
	Baseline study sample [i.e. individuals entering the study and their key characteristics and sampling frame are adequately described]	x				
	Study sample represents population of interest on key characteristics, sufficient to limit potential bias to results	Low risk of bias				
Study attrition	Response rate (i.e., proportion of study sample completing the study and providing outcome data) is adequate	x				
	Participants lost to follow-up are adequately described for key characteristics					x
	Statement as to the possible effect on the results from missing data					x
	Loss to follow-up is not associated with key characteristics	Low risk of bias				
Prognostic factor measurement	Clear definition of the prognostic factors measured is provided (e.g. imaging modality method, measurement, and timing described).		x			
	Specified instrument and personnel for measurement of predictive factors	x				
	Continuous variables are reported or appropriate (i.e. not data- dependent) cut-off points are used and specified a priori	x				
	Blinding: were estimators of risk factor status and of outcomes blinded?	x				
	The prognostic factor(s) of interest is (are) adequately measured in study participants to sufficiently limit potential bias	Low risk of bias				
Outcome measurement	Is the outcome(s) clearly defined?		x			
	The outcome measure and method used are adequately valid and reliable to limit misclassification bias	Moderate risk of bias				
Study confounding	Do the authors address potential confounders?		x			
	Important potential confounders are appropriately accounted for, limiting potential bias with respect to the prognostic factor of interest.	Moderate risk of bias				
Analysis and reporting	There is sufficient presentation of data to assess the adequacy of the analysis strategy and there is no selective reporting	x				
	The statistical analysis is appropriate for the study design, limiting potential for the presentation of invalid results	Low risk of bias				

NA*: not applicable. Note: The above table was adapted from: Hayden et al., 2013.

Overall opinion of study quality= Low risk of bias

First Author: Khanduri et al., 2013 **ID:** 6378321

Potential Bias	Items to be considered for assessment of potential opportunity for bias	Yes	Partly	No	Unsure	NA*
Study population /sample selection	Inclusion and exclusion criteria are adequately described [including explicit diagnostic criteria, start/finish date of recruitment]	x				
	Baseline study sample [i.e. individuals entering the study and their key characteristics and sampling frame are adequately described]	x				
	Study sample represents population of interest on key characteristics, sufficient to limit potential bias to results	Low risk of bias				
Study attrition	Response rate (i.e., proportion of study sample completing the study and providing outcome data) is adequate	x				
	Participants lost to follow-up are adequately described for key characteristics	x				
	Statement as to the possible effect on the results from missing data					x
	Loss to follow-up is not associated with key characteristics	Low risk of bias				
Prognostic factor measurement	Clear definition of the prognostic factors measured is provided (e.g. imaging modality method, measurement, and timing described).	x				
	Specified instrument and personnel for measurement of predictive factors	x				
	Continuous variables are reported or appropriate (i.e. not data- dependent) cut-off points are used and specified a priori	x				
	Blinding: were estimators of risk factor status and of outcomes blinded?		x			
	The prognostic factor(s) of interest is (are) adequately measured in study participants to sufficiently limit potential bias	Low risk of bas				
Outcome measurement	Is the outcome(s) clearly defined?		x			
	The outcome measure and method used are adequately valid and reliable to limit misclassification bias	Moderate risk of bas				
Study confounding	Do the authors address potential confounders?	x				
	Important potential confounders are appropriately accounted for, limiting potential bias with respect to the prognostic factor of interest.	Low risk of bias				
Analysis and reporting	There is sufficient presentation of data to assess the adequacy of the analysis strategy and there is no selective reporting		x			
	The statistical analysis is appropriate for the study design, limiting potential for the presentation of invalid results	Moderate risk of bias				
NA*: not applicable. Note: The above table was adapted from: Hayden et al., 2013.						
**Overall opinion of study quality= Low risk of bias**						

First Author: Kumari et al., 2019 **ID:** 68614385

**Potential Bias**	**Items to be considered for assessment of potential opportunity for bias**	**Yes**	**Partly**	**No**	**Unsure**	**NA***
Study population /sample selection	Inclusion and exclusion criteria are adequately described [including explicit diagnostic criteria, start/finish date of recruitment]		x			
	Baseline study sample [i.e. individuals entering the study and their key characteristics and sampling frame are adequately described]		x			
	Study sample represents population of interest on key characteristics, sufficient to limit potential bias to results	Moderate risk of bias				
Study attrition	Response rate (i.e., proportion of study sample completing the study and providing outcome data) is adequate				x	
	Participants lost to follow-up are adequately described for key characteristics				x	
	Statement as to the possible effect on the results from missing data				x	
	Loss to follow-up is not associated with key characteristics	Moderate risk of bias				
Prognostic factor measurement	Clear definition of the prognostic factors measured is provided (e.g. imaging modality method, measurement, and timing described).	x				
	Specified instrument and personnel for measurement of predictive factors	x				
	Continuous variables are reported or appropriate (i.e. not data- dependent) cut-off points are used and specified a priori	x				
	Blinding: were estimators of risk factor status and of outcomes blinded?			x		
	The prognostic factor(s) of interest is (are) adequately measured in study participants to sufficiently limit potential bias	Low risk of bias				
Outcome measurement	Is the outcome(s) clearly defined?		x			
	The outcome measure and method used are adequately valid and reliable to limit misclassification bias	Moderate risk of bias				
Study confounding	Do the authors address potential confounders?	x				
	Important potential confounders are appropriately accounted for, limiting potential bias with respect to the prognostic factor of interest.	Low risk of bias				
Analysis and reporting	There is sufficient presentation of data to assess the adequacy of the analysis strategy and there is no selective reporting	x				
	The statistical analysis is appropriate for the study design, limiting potential for the presentation of invalid results	Low risk of bias				
NA*: not applicable. Note: The above table was adapted from: Hayden et al., 2013.						
**Overall opinion of study quality= Moderate risk of bias**						

First Author: Lakhkar et al., 2006 **ID:** 74903014

Potential Bias	Items to be considered for assessment of potential opportunity for bias	Yes	Partly	No	Unsure	NA*
Study population /sample selection	Inclusion and exclusion criteria are adequately described [including explicit diagnostic criteria, start/finish date of recruitment]		x			
	Baseline study sample [i.e. individuals entering the study and their key characteristics and sampling frame are adequately described]	x				
	Study sample represents population of interest on key characteristics, sufficient to limit potential bias to results	Moderate risk of bias				
Study attrition	Response rate (i.e., proportion of study sample completing the study and providing outcome data) is adequate					x
	Participants lost to follow-up are adequately described for key characteristics					x
	Statement as to the possible effect on the results from missing data					x
	Loss to follow-up is not associated with key characteristics	Low risk of bias				
Prognostic factor measurement	Clear definition of the prognostic factors measured is provided (e.g. imaging modality method, measurement, and timing described).	x				
	Specified instrument and personnel for measurement of predictive factors		x			
	Continuous variables are reported or appropriate (i.e. not data- dependent) cut-off points are used and specified a priori	x				
	Blinding: were estimators of risk factor status and of outcomes blinded?			x		
	The prognostic factor(s) of interest is (are) adequately measured in study participants to sufficiently limit potential bias	Moderate risk of bias				
Outcome measurement	Is the outcome(s) clearly defined?	x				
	The outcome measure and method used are adequately valid and reliable to limit misclassification bias	Low risk of bias				
Study confounding	Do the authors address potential confounders?	x				
	Important potential confounders are appropriately accounted for, limiting potential bias with respect to the prognostic factor of interest.	Low risk of bias				
Analysis and reporting	There is sufficient presentation of data to assess the adequacy of the analysis strategy and there is no selective reporting		x			
	The statistical analysis is appropriate for the study design, limiting potential for the presentation of invalid results	Moderate risk of bias				
NA*: not applicable. Note: The above table was adapted from: Hayden et al., 2013.						
**Overall opinion of study quality= Moderate risk of bias**						

First Author: Lakshmi et al., 2013 **ID:** 6378401

Potential Bias	Items to be considered for assessment of potential opportunity for bias	Yes	Partly	No	Unsure	NA*
Study population /sample selection	Inclusion and exclusion criteria are adequately described [including explicit diagnostic criteria, start/finish date of recruitment]	x				
	Baseline study sample [i.e. individuals entering the study and their key characteristics and sampling frame are adequately described]	x				
	Study sample represents population of interest on key characteristics, sufficient to limit potential bias to results	Low risk of bias				
Study attrition	Response rate (i.e., proportion of study sample completing the study and providing outcome data) is adequate	x				
	Participants lost to follow-up are adequately described for key characteristics	x				
	Statement as to the possible effect on the results from missing data			x		
	Loss to follow-up is not associated with key characteristics	Low risk of bias				
Prognostic factor measurement	Clear definition of the prognostic factors measured is provided (e.g. imaging modality method, measurement, and timing described).	x				
	Specified instrument and personnel for measurement of predictive factors	x				
	Continuous variables are reported or appropriate (i.e. not data- dependent) cut-off points are used and specified a priori	x				
	Blinding: were estimators of risk factor status and of outcomes blinded?	x				
	The prognostic factor(s) of interest is (are) adequately measured in study participants to sufficiently limit potential bias	Low risk of bias				
Outcome measurement	Is the outcome(s) clearly defined?	x				
	The outcome measure and method used are adequately valid and reliable to limit misclassification bias	Low risk of bias				
Study confounding	Do the authors address potential confounders?	x				
	Important potential confounders are appropriately accounted for, limiting potential bias with respect to the prognostic factor of interest.	Low risk of bias				
Analysis and reporting	There is sufficient presentation of data to assess the adequacy of the analysis strategy and there is no selective reporting		x			
	The statistical analysis is appropriate for the study design, limiting potential for the presentation of invalid results	Moderate risk of bias				
NA*: not applicable. Note: The above table was adapted from: Hayden et al., 2013.						
**Overall opinion of study quality= Low risk of bias**						

First Author: Malik et al., 2013 **ID:** 6378519

Potential Bias	Items to be considered for assessment of potential opportunity for bias	Yes	Partly	No	Unsure	NA*
Study population /sample selection	Inclusion and exclusion criteria are adequately described [including explicit diagnostic criteria, start/finish date of recruitment]	x				
	Baseline study sample [i.e. individuals entering the study and their key characteristics and sampling frame are adequately described]	x				
	Study sample represents population of interest on key characteristics, sufficient to limit potential bias to results	Low risk of bias				
Study attrition	Response rate (i.e., proportion of study sample completing the study and providing outcome data) is adequate	x				
	Participants lost to follow-up are adequately described for key characteristics			x		
	Statement as to the possible effect on the results from missing data				x	
	Loss to follow-up is not associated with key characteristics	High risk of bias				
Prognostic factor measurement	Clear definition of the prognostic factors measured is provided (e.g. imaging modality method, measurement, and timing described).			x		
	Specified instrument and personnel for measurement of predictive factors		x			
	Continuous variables are reported or appropriate (i.e. not data- dependent) cut-off points are used and specified a priori				x	
	Blinding: were estimators of risk factor status and of outcomes blinded?				x	
	The prognostic factor(s) of interest is (are) adequately measured in study participants to sufficiently limit potential bias	High risk of bias				
Outcome measurement	Is the outcome(s) clearly defined?		x			
	The outcome measure and method used are adequately valid and reliable to limit misclassification bias	Moderate risk of bias				
Study confounding	Do the authors address potential confounders?		x			
	Important potential confounders are appropriately accounted for, limiting potential bias with respect to the prognostic factor of interest.	Moderate risk of bias				
Analysis and reporting	There is sufficient presentation of data to assess the adequacy of the analysis strategy and there is no selective reporting		x			
	The statistical analysis is appropriate for the study design, limiting potential for the presentation of invalid results	Moderate risk of bias				
NA*: not applicable. Note: The above table was adapted from: Hayden et al., 2013.						
**Overall opinion of study quality= High risk of bias**						

First Author: Masihi et al., 2019 **ID:** 68614415

**Potential Bias**	**Items to be considered for assessment of potential opportunity for bias**	**Yes**	**Partly**	**No**	**Unsure**	**NA***
Study population /sample selection	Inclusion and exclusion criteria are adequately described [including explicit diagnostic criteria, start/finish date of recruitment]	x				
	Baseline study sample [i.e. individuals entering the study and their key characteristics and sampling frame are adequately described]	x				
	Study sample represents population of interest on key characteristics, sufficient to limit potential bias to results	Low risk of bias				
Study attrition	Response rate (i.e., proportion of study sample completing the study and providing outcome data) is adequate			x		
	Participants lost to follow-up are adequately described for key characteristics			x		
	Statement as to the possible effect on the results from missing data			x		
	Loss to follow-up is not associated with key characteristics	Moderate risk of bias				
Prognostic factor measurement	Clear definition of the prognostic factors measured is provided (e.g. imaging modality method, measurement, and timing described).	x				
	Specified instrument and personnel for measurement of predictive factors			x		
	Continuous variables are reported or appropriate (i.e. not data- dependent) cut-off points are used and specified a priori	x				
	Blinding: were estimators of risk factor status and of outcomes blinded?			x		
	The prognostic factor(s) of interest is (are) adequately measured in study participants to sufficiently limit potential bias	Moderate risk of bias				
Outcome measurement	Is the outcome(s) clearly defined?	x				
	The outcome measure and method used are adequately valid and reliable to limit misclassification bias	Low risk of bias				
Study confounding	Do the authors address potential confounders?	x				
	Important potential confounders are appropriately accounted for, limiting potential bias with respect to the prognostic factor of interest.	Low risk of bias				
Analysis and reporting	There is sufficient presentation of data to assess the adequacy of the analysis strategy and there is no selective reporting	x				
	The statistical analysis is appropriate for the study design, limiting potential for the presentation of invalid results	Low risk of bias				
NA*: not applicable. Note: The above table was adapted from: Hayden et al., 2013.						
**Overall opinion of study quality= Low risk of bias**						

First Author: Mullick et al., 1993 **ID:** 6378675

Potential Bias	Items to be considered for assessment of potential opportunity for bias	Yes	Partly	No	Unsure	NA*
Study population /sample selection	Inclusion and exclusion criteria are adequately described [including explicit diagnostic criteria, start/finish date of recruitment]	x				
	Baseline study sample [i.e. individuals entering the study and their key characteristics and sampling frame are adequately described]		x			
	Study sample represents population of interest on key characteristics, sufficient to limit potential bias to results	Low risk of bias				
Study attrition	Response rate (i.e., proportion of study sample completing the study and providing outcome data) is adequate	x				
	Participants lost to follow-up are adequately described for key characteristics	x				
	Statement as to the possible effect on the results from missing data					x
	Loss to follow-up is not associated with key characteristics	Low risk of bias				
Prognostic factor measurement	Clear definition of the prognostic factors measured is provided (e.g. imaging modality method, measurement, and timing described).	x				
	Specified instrument and personnel for measurement of predictive factors		x			
	Continuous variables are reported or appropriate (i.e. not data- dependent) cut-off points are used and specified a priori		x			
	Blinding: were estimators of risk factor status and of outcomes blinded?				x	
	The prognostic factor(s) of interest is (are) adequately measured in study participants to sufficiently limit potential bias	Moderate risk of bias				
Outcome measurement	Is the outcome(s) clearly defined?		x			
	The outcome measure and method used are adequately valid and reliable to limit misclassification bias	Moderate risk of bias				
Study confounding	Do the authors address potential confounders?		x			
	Important potential confounders are appropriately accounted for, limiting potential bias with respect to the prognostic factor of interest.	Moderate risk of bias				
Analysis and reporting	There is sufficient presentation of data to assess the adequacy of the analysis strategy and there is no selective reporting		x			
	The statistical analysis is appropriate for the study design, limiting potential for the presentation of invalid results	Moderate risk of bias				
NA*: not applicable. Note: The above table was adapted from: Hayden et al., 2013.						
**Overall opinion of study quality= Moderate risk of bias**						

First Author: Nagar et al., 2015 **ID:** 6378692

Potential Bias	Items to be considered for assessment of potential opportunity for bias	Yes	Partly	No	Unsure	NA*
Study population /sample selection	Inclusion and exclusion criteria are adequately described [including explicit diagnostic criteria, start/finish date of recruitment]	x				
	Baseline study sample [i.e. individuals entering the study and their key characteristics and sampling frame are adequately described]		x			
	Study sample represents population of interest on key characteristics, sufficient to limit potential bias to results	Low risk of bias				
Study attrition	Response rate (i.e., proportion of study sample completing the study and providing outcome data) is adequate	x				
	Participants lost to follow-up are adequately described for key characteristics				x	
	Statement as to the possible effect on the results from missing data					x
	Loss to follow-up is not associated with key characteristics	Low risk of bias				
Prognostic factor measurement	Clear definition of the prognostic factors measured is provided (e.g. imaging modality method, measurement, and timing described).	x				
	Specified instrument and personnel for measurement of predictive factors			x		
	Continuous variables are reported or appropriate (i.e. not data- dependent) cut-off points are used and specified a priori	x				
	Blinding: were estimators of risk factor status and of outcomes blinded?			x		
	The prognostic factor(s) of interest is (are) adequately measured in study participants to sufficiently limit potential bias	Moderate risk of bias				
Outcome measurement	Is the outcome(s) clearly defined?		x			
	The outcome measure and method used are adequately valid and reliable to limit misclassification bias	Moderate risk of bias				
Study confounding	Do the authors address potential confounders?	x				
	Important potential confounders are appropriately accounted for, limiting potential bias with respect to the prognostic factor of interest.	Low risk of bias				
Analysis and reporting	There is sufficient presentation of data to assess the adequacy of the analysis strategy and there is no selective reporting		x			
	The statistical analysis is appropriate for the study design, limiting potential for the presentation of invalid results	Moderate risk of bias				
NA*: not applicable. Note: The above table was adapted from: Hayden et al., 2013.						
**Overall opinion of study quality= Moderate risk of bias**						

First Author: Najam et al., 2016 **ID:** 6378705

Potential Bias	Items to be considered for assessment of potential opportunity for bias	Yes	Partly	No	Unsure	NA*
Study population /sample selection	Inclusion and exclusion criteria are adequately described [including explicit diagnostic criteria, start/finish date of recruitment]			x		
	Baseline study sample [i.e. individuals entering the study and their key characteristics and sampling frame are adequately described]			x		
	Study sample represents population of interest on key characteristics, sufficient to limit potential bias to results	High risk of bias				
Study attrition	Response rate (i.e., proportion of study sample completing the study and providing outcome data) is adequate		x			
	Participants lost to follow-up are adequately described for key characteristics		x			
	Statement as to the possible effect on the results from missing data			x		
	Loss to follow-up is not associated with key characteristics	High risk of bias				
Prognostic factor measurement	Clear definition of the prognostic factors measured is provided (e.g. imaging modality method, measurement, and timing described).	x				
	Specified instrument and personnel for measurement of predictive factors			x		
	Continuous variables are reported or appropriate (i.e. not data- dependent) cut-off points are used and specified a priori	x				
	Blinding: were estimators of risk factor status and of outcomes blinded?			x		
	The prognostic factor(s) of interest is (are) adequately measured in study participants to sufficiently limit potential bias	Moderate risk of bias				
Outcome measurement	Is the outcome(s) clearly defined?			x		
	The outcome measure and method used are adequately valid and reliable to limit misclassification bias	High risk of bias				
Study confounding	Do the authors address potential confounders?				x	
	Important potential confounders are appropriately accounted for, limiting potential bias with respect to the prognostic factor of interest.	High risk of bias				
Analysis and reporting	There is sufficient presentation of data to assess the adequacy of the analysis strategy and there is no selective reporting			x		
	The statistical analysis is appropriate for the study design, limiting potential for the presentation of invalid results	High risk of bias				
NA*: not applicable. Note: The above table was adapted from: Hayden et al., 2013.						
**Overall opinion of study quality= High risk of bias**						

First Author: Nouh et al., 2011 **ID:** 6378752

Potential Bias	Items to be considered for assessment of potential opportunity for bias	Yes	Partly	No	Unsure	NA*
Study population /sample selection	Inclusion and exclusion criteria are adequately described [including explicit diagnostic criteria, start/finish date of recruitment]	x				
	Baseline study sample [i.e. individuals entering the study and their key characteristics and sampling frame are adequately described]	x				
	Study sample represents population of interest on key characteristics, sufficient to limit potential bias to results	Low risk of bias				
Study attrition	Response rate (i.e., proportion of study sample completing the study and providing outcome data) is adequate	x				
	Participants lost to follow-up are adequately described for key characteristics					x
	Statement as to the possible effect on the results from missing data				x	
	Loss to follow-up is not associated with key characteristics	Low risk of bias				
Prognostic factor measurement	Clear definition of the prognostic factors measured is provided (e.g. imaging modality method, measurement, and timing described).	x				
	Specified instrument and personnel for measurement of predictive factors	x				
	Continuous variables are reported or appropriate (i.e. not data- dependent) cut-off points are used and specified a priori	x				
	Blinding: were estimators of risk factor status and of outcomes blinded?				x	
	The prognostic factor(s) of interest is (are) adequately measured in study participants to sufficiently limit potential bias	Low risk of bias				
Outcome measurement	Is the outcome(s) clearly defined?		x			
	The outcome measure and method used are adequately valid and reliable to limit misclassification bias	Low risk of bias				
Study confounding	Do the authors address potential confounders?	x				
	Important potential confounders are appropriately accounted for, limiting potential bias with respect to the prognostic factor of interest.	Low risk of bias				
Analysis and reporting	There is sufficient presentation of data to assess the adequacy of the analysis strategy and there is no selective reporting		x			
	The statistical analysis is appropriate for the study design, limiting potential for the presentation of invalid results	Moderate risk of bias				

NA*: not applicable. Note: The above table was adapted from: Hayden et al., 2013.

Overall opinion of study quality= Low risk of bias

First Author: Pares et al., 2008 **ID:** 6378809

Potential Bias	Items to be considered for assessment of potential opportunity for bias	Yes	Partly	No	Unsure	NA*
Study population /sample selection	Inclusion and exclusion criteria are adequately described [including explicit diagnostic criteria, start/finish date of recruitment]	x				
	Baseline study sample [i.e. individuals entering the study and their key characteristics and sampling frame are adequately described]	x				
	Study sample represents population of interest on key characteristics, sufficient to limit potential bias to results	Low risk of bias				
Study attrition	Response rate (i.e., proportion of study sample completing the study and providing outcome data) is adequate	x				
	Participants lost to follow-up are adequately described for key characteristics					x
	Statement as to the possible effect on the results from missing data					x
	Loss to follow-up is not associated with key characteristics	Low risk of bias				
Prognostic factor measurement	Clear definition of the prognostic factors measured is provided (e.g. imaging modality method, measurement, and timing described).	x				
	Specified instrument and personnel for measurement of predictive factors	x				
	Continuous variables are reported or appropriate (i.e. not data- dependent) cut-off points are used and specified a priori	x				
	Blinding: were estimators of risk factor status and of outcomes blinded?				x	
	The prognostic factor(s) of interest is (are) adequately measured in study participants to sufficiently limit potential bias	Moderate risk of bias				
Outcome measurement	Is the outcome(s) clearly defined?	x				
	The outcome measure and method used are adequately valid and reliable to limit misclassification bias	Low risk of bias				
Study confounding	Do the authors address potential confounders?	x				
	Important potential confounders are appropriately accounted for, limiting potential bias with respect to the prognostic factor of interest.	Low risk of bias				
Analysis and reporting	There is sufficient presentation of data to assess the adequacy of the analysis strategy and there is no selective reporting	x				
	The statistical analysis is appropriate for the study design, limiting potential for the presentation of invalid results	Low risk of bias				
NA*: not applicable. Note: The above table was adapted from: Hayden et al., 2013.						
**Overall opinion of study quality= Low risk of bias**						

First Author: Pattinson et al., 1991 **ID:** 74903015

Potential Bias	Items to be considered for assessment of potential opportunity for bias	Yes	Partly	No	Unsure	NA*
Study population /sample selection	Inclusion and exclusion criteria are adequately described [including explicit diagnostic criteria, start/finish date of recruitment]	x				
	Baseline study sample [i.e. individuals entering the study and their key characteristics and sampling frame are adequately described]	x				
	Study sample represents population of interest on key characteristics, sufficient to limit potential bias to results	Low risk of bias				
Study attrition	Response rate (i.e., proportion of study sample completing the study and providing outcome data) is adequate					x
	Participants lost to follow-up are adequately described for key characteristics					x
	Statement as to the possible effect on the results from missing data					x
	Loss to follow-up is not associated with key characteristics	Low risk of bias				
Prognostic factor measurement	Clear definition of the prognostic factors measured is provided (e.g. imaging modality method, measurement, and timing described).	x				
	Specified instrument and personnel for measurement of predictive factors		x			
	Continuous variables are reported or appropriate (i.e. not data- dependent) cut-off points are used and specified a priori	x				
	Blinding: were estimators of risk factor status and of outcomes blinded?	x				
	The prognostic factor(s) of interest is (are) adequately measured in study participants to sufficiently limit potential bias	Low risk of bias				
Outcome measurement	Is the outcome(s) clearly defined?		x			
	The outcome measure and method used are adequately valid and reliable to limit misclassification bias	Moderate risk of bias				
Study confounding	Do the authors address potential confounders?	x				
	Important potential confounders are appropriately accounted for, limiting potential bias with respect to the prognostic factor of interest.	Low risk of bias				
Analysis and reporting	There is sufficient presentation of data to assess the adequacy of the analysis strategy and there is no selective reporting		x			
	The statistical analysis is appropriate for the study design, limiting potential for the presentation of invalid results	Moderate risk of bias				
NA*: not applicable. Note: The above table was adapted from: Hayden et al., 2013.						
**Overall opinion of study quality= Low risk of bias**						

First Author: Pattinson et al., 1993 **ID:** 6378815

**Potential Bias**	**Items to be considered for assessment of potential opportunity for bias**	**Yes**	**Partly**	**No**	**Unsure**	**NA***
Study population /sample selection	Inclusion and exclusion criteria are adequately described [including explicit diagnostic criteria, start/finish date of recruitment]	x				
	Baseline study sample [i.e. individuals entering the study and their key characteristics and sampling frame are adequately described]	x				
	Study sample represents population of interest on key characteristics, sufficient to limit potential bias to results	Low risk of bias				
Study attrition	Response rate (i.e., proportion of study sample completing the study and providing outcome data) is adequate	x				
	Participants lost to follow-up are adequately described for key characteristics	x				
	Statement as to the possible effect on the results from missing data				x	
	Loss to follow-up is not associated with key characteristics	Low risk of bias				
Prognostic factor measurement	Clear definition of the prognostic factors measured is provided (e.g. imaging modality method, measurement, and timing described).	x				
	Specified instrument and personnel for measurement of predictive factors		x			
	Continuous variables are reported or appropriate (i.e. not data- dependent) cut-off points are used and specified a priori	x				
	Blinding: were estimators of risk factor status and of outcomes blinded?				x	
	The prognostic factor(s) of interest is (are) adequately measured in study participants to sufficiently limit potential bias	Moderate risk of bias				
Outcome measurement	Is the outcome(s) clearly defined?	x				
	The outcome measure and method used are adequately valid and reliable to limit misclassification bias	Low risk of bias				
Study confounding	Do the authors address potential confounders?	x				
	Important potential confounders are appropriately accounted for, limiting potential bias with respect to the prognostic factor of interest.	Low risk of bias				
Analysis and reporting	There is sufficient presentation of data to assess the adequacy of the analysis strategy and there is no selective reporting	x				
	The statistical analysis is appropriate for the study design, limiting potential for the presentation of invalid results	Low risk of bias				
NA*: not applicable. Note: The above table was adapted from: Hayden et al., 2013.						
**Overall opinion of study quality= Low risk of bias**						

First Author: Phupong et al., 2003 **ID:** 6378830

**Potential Bias**	**Items to be considered for assessment of potential opportunity for bias**	**Yes**	**Partly**	**No**	**Unsure**	**NA***
Study population /sample selection	Inclusion and exclusion criteria are adequately described [including explicit diagnostic criteria, start/finish date of recruitment]	x				
	Baseline study sample [i.e. individuals entering the study and their key characteristics and sampling frame are adequately described]	x				
	Study sample represents population of interest on key characteristics, sufficient to limit potential bias to results	Low risk of bias				
Study attrition	Response rate (i.e., proportion of study sample completing the study and providing outcome data) is adequate	x				
	Participants lost to follow-up are adequately described for key characteristics	x				
	Statement as to the possible effect on the results from missing data					x
	Loss to follow-up is not associated with key characteristics	Low risk of bias				
Prognostic factor measurement	Clear definition of the prognostic factors measured is provided (e.g. imaging modality method, measurement, and timing described).	x				
	Specified instrument and personnel for measurement of predictive factors	x				
	Continuous variables are reported or appropriate (i.e. not data- dependent) cut-off points are used and specified a priori	x				
	Blinding: were estimators of risk factor status and of outcomes blinded?	x				
	The prognostic factor(s) of interest is (are) adequately measured in study participants to sufficiently limit potential bias	Low risk of bias				
Outcome measurement	Is the outcome(s) clearly defined?	x				
	The outcome measure and method used are adequately valid and reliable to limit misclassification bias	Low risk of bias				
Study confounding	Do the authors address potential confounders?	x				
	Important potential confounders are appropriately accounted for, limiting potential bias with respect to the prognostic factor of interest.	Low risk of bias				
Analysis and reporting	There is sufficient presentation of data to assess the adequacy of the analysis strategy and there is no selective reporting	x				
	The statistical analysis is appropriate for the study design, limiting potential for the presentation of invalid results	Low risk of bias				

NA*: not applicable. Note: The above table was adapted from: Hayden et al., 2013.

Overall opinion of study quality= Low risk of bias

First Author: Rani et al., 2016 **ID:** 74903020

Potential Bias	Items to be considered for assessment of potential opportunity for bias	Yes	Partly	No	Unsure	NA*
Study population /sample selection	Inclusion and exclusion criteria are adequately described [including explicit diagnostic criteria, start/finish date of recruitment]	x				
	Baseline study sample [i.e. individuals entering the study and their key characteristics and sampling frame are adequately described]		x			
	Study sample represents population of interest on key characteristics, sufficient to limit potential bias to results	Moderate risk of bias				
Study attrition	Response rate (i.e., proportion of study sample completing the study and providing outcome data) is adequate					x
	Participants lost to follow-up are adequately described for key characteristics					x
	Statement as to the possible effect on the results from missing data					x
	Loss to follow-up is not associated with key characteristics	Low risk of bias				
Prognostic factor measurement	Clear definition of the prognostic factors measured is provided (e.g. imaging modality method, measurement, and timing described).	x				
	Specified instrument and personnel for measurement of predictive factors		x			
	Continuous variables are reported or appropriate (i.e. not data- dependent) cut-off points are used and specified a priori		x			
	Blinding: were estimators of risk factor status and of outcomes blinded?			x		
	The prognostic factor(s) of interest is (are) adequately measured in study participants to sufficiently limit potential bias	Moderate risk of bias				
Outcome measurement	Is the outcome(s) clearly defined?	x				
	The outcome measure and method used are adequately valid and reliable to limit misclassification bias	Low risk of bias				
Study confounding	Do the authors address potential confounders?		x			
	Important potential confounders are appropriately accounted for, limiting potential bias with respect to the prognostic factor of interest.	Moderate risk of bias				
Analysis and reporting	There is sufficient presentation of data to assess the adequacy of the analysis strategy and there is no selective reporting		x			
	The statistical analysis is appropriate for the study design, limiting potential for the presentation of invalid results	Moderate risk of bias				
NA*: not applicable. Note: The above table was adapted from: Hayden et al., 2013.						
**Overall opinion of study quality= Moderate risk of bias**						

First Author: Rocca et al., 1995 **ID:** 74903016

Potential Bias	Items to be considered for assessment of potential opportunity for bias	Yes	Partly	No	Unsure	NA*
Study population /sample selection	Inclusion and exclusion criteria are adequately described [including explicit diagnostic criteria, start/finish date of recruitment]	x				
	Baseline study sample [i.e. individuals entering the study and their key characteristics and sampling frame are adequately described]		x			
	Study sample represents population of interest on key characteristics, sufficient to limit potential bias to results	Moderate risk of bias				
Study attrition	Response rate (i.e., proportion of study sample completing the study and providing outcome data) is adequate					x
	Participants lost to follow-up are adequately described for key characteristics					x
	Statement as to the possible effect on the results from missing data					x
	Loss to follow-up is not associated with key characteristics	Low risk of bias				
Prognostic factor measurement	Clear definition of the prognostic factors measured is provided (e.g. imaging modality method, measurement, and timing described).	x				
	Specified instrument and personnel for measurement of predictive factors		x			
	Continuous variables are reported or appropriate (i.e. not data- dependent) cut-off points are used and specified a priori	x				
	Blinding: were estimators of risk factor status and of outcomes blinded?			x		
	The prognostic factor(s) of interest is (are) adequately measured in study participants to sufficiently limit potential bias	Moderate risk of bias				
Outcome measurement	Is the outcome(s) clearly defined?		x			
	The outcome measure and method used are adequately valid and reliable to limit misclassification bias	Moderate risk of bias				
Study confounding	Do the authors address potential confounders?		x			
	Important potential confounders are appropriately accounted for, limiting potential bias with respect to the prognostic factor of interest.	Moderate risk of bias				
Analysis and reporting	There is sufficient presentation of data to assess the adequacy of the analysis strategy and there is no selective reporting		x			
	The statistical analysis is appropriate for the study design, limiting potential for the presentation of invalid results	Moderate risk of bias				
NA*: not applicable. Note: The above table was adapted from: Hayden et al., 2013.						
**Overall opinion of study quality= Moderate risk of bias**						

First Author: Verma et al., 2016 **ID:** 6379243

Potential Bias	Items to be considered for assessment of potential opportunity for bias	Yes	Partly	No	Unsure	NA*
Study population /sample selection	Inclusion and exclusion criteria are adequately described [including explicit diagnostic criteria, start/finish date of recruitment]	x				
	Baseline study sample [i.e. individuals entering the study and their key characteristics and sampling frame are adequately described]	x				
	Study sample represents population of interest on key characteristics, sufficient to limit potential bias to results	Low risk of bias				
Study attrition	Response rate (i.e., proportion of study sample completing the study and providing outcome data) is adequate	x				
	Participants lost to follow-up are adequately described for key characteristics					x
	Statement as to the possible effect on the results from missing data				x	
	Loss to follow-up is not associated with key characteristics	Low risk of bias				
Prognostic factor measurement	Clear definition of the prognostic factors measured is provided (e.g. imaging modality method, measurement, and timing described).	x				
	Specified instrument and personnel for measurement of predictive factors	x				
	Continuous variables are reported or appropriate (i.e. not data- dependent) cut-off points are used and specified a priori	x				
	Blinding: were estimators of risk factor status and of outcomes blinded?				x	
	The prognostic factor(s) of interest is (are) adequately measured in study participants to sufficiently limit potential bias	Low risk of bias				
Outcome measurement	Is the outcome(s) clearly defined?	x				
	The outcome measure and method used are adequately valid and reliable to limit misclassification bias	Low risk of bias				
Study confounding	Do the authors address potential confounders?	x				
	Important potential confounders are appropriately accounted for, limiting potential bias with respect to the prognostic factor of interest.	Low risk of bias				
Analysis and reporting	There is sufficient presentation of data to assess the adequacy of the analysis strategy and there is no selective reporting	x				
	The statistical analysis is appropriate for the study design, limiting potential for the presentation of invalid results	Low risk of bias				
NA*: not applicable. Note: The above table was adapted from: Hayden et al., 2013.						
**Overall opinion of study quality= Low risk of bias**						

First Author: Waa et al., 2010 **ID:** 6379255

Potential Bias	Items to be considered for assessment of potential opportunity for bias	Yes	Partly	No	Unsure	NA*
Study population /sample selection	Inclusion and exclusion criteria are adequately described [including explicit diagnostic criteria, start/finish date of recruitment]	x				
	Baseline study sample [i.e. individuals entering the study and their key characteristics and sampling frame are adequately described]	x				
	Study sample represents population of interest on key characteristics, sufficient to limit potential bias to results	Low risk of bias				
Study attrition	Response rate (i.e., proportion of study sample completing the study and providing outcome data) is adequate	x				
	Participants lost to follow-up are adequately described for key characteristics	x				
	Statement as to the possible effect on the results from missing data	x				
	Loss to follow-up is not associated with key characteristics	Low risk of bias				
Prognostic factor measurement	Clear definition of the prognostic factors measured is provided (e.g. imaging modality method, measurement, and timing described).	x				
	Specified instrument and personnel for measurement of predictive factors	x				
	Continuous variables are reported or appropriate (i.e. not data- dependent) cut-off points are used and specified a priori	x				
	Blinding: were estimators of risk factor status and of outcomes blinded?			x		
	The prognostic factor(s) of interest is (are) adequately measured in study participants to sufficiently limit potential bias	Low risk of bias				
Outcome measurement	Is the outcome(s) clearly defined?		x			
	The outcome measure and method used are adequately valid and reliable to limit misclassification bias	Moderate risk of bias				
Study confounding	Do the authors address potential confounders?		x			
	Important potential confounders are appropriately accounted for, limiting potential bias with respect to the prognostic factor of interest.	Moderate risk of bias				
Analysis and reporting	There is sufficient presentation of data to assess the adequacy of the analysis strategy and there is no selective reporting		x			
	The statistical analysis is appropriate for the study design, limiting potential for the presentation of invalid results	Moderate risk of bias				
NA*: not applicable. Note: The above table was adapted from: Hayden et al., 2013.						
**Overall opinion of study quality= Moderate risk of bias**						

First Author: Yelikar et al., 2013 **ID:** 6379339

Potential Bias	Items to be considered for assessment of potential opportunity for bias	Yes	Partly	No	Unsure	NA*
Study population /sample selection	Inclusion and exclusion criteria are adequately described [including explicit diagnostic criteria, start/finish date of recruitment]	x				
	Baseline study sample [i.e. individuals entering the study and their key characteristics and sampling frame are adequately described]	x				
	Study sample represents population of interest on key characteristics, sufficient to limit potential bias to results	Low risk of bias				
Study attrition	Response rate (i.e., proportion of study sample completing the study and providing outcome data) is adequate		x			
	Participants lost to follow-up are adequately described for key characteristics				x	
	Statement as to the possible effect on the results from missing data				x	
	Loss to follow-up is not associated with key characteristics	Moderate risk of bias				
Prognostic factor measurement	Clear definition of the prognostic factors measured is provided (e.g. imaging modality method, measurement, and timing described).	x				
	Specified instrument and personnel for measurement of predictive factors		x			
	Continuous variables are reported or appropriate (i.e. not data- dependent) cut-off points are used and specified a priori	x				
	Blinding: were estimators of risk factor status and of outcomes blinded?			x		
	The prognostic factor(s) of interest is (are) adequately measured in study participants to sufficiently limit potential bias	Moderate risk of bias				
Outcome measurement	Is the outcome(s) clearly defined?		x			
	The outcome measure and method used are adequately valid and reliable to limit misclassification bias	Moderate risk of bias				
Study confounding	Do the authors address potential confounders?	x				
	Important potential confounders are appropriately accounted for, limiting potential bias with respect to the prognostic factor of interest.	Low risk of bias				
Analysis and reporting	There is sufficient presentation of data to assess the adequacy of the analysis strategy and there is no selective reporting		x			
	The statistical analysis is appropriate for the study design, limiting potential for the presentation of invalid results	Moderate risk of bias				
NA*: not applicable. Note: The above table was adapted from: Hayden et al., 2013.						
**Overall opinion of study quality= Moderate risk of bias**						

First Author: Zarean et al., 2018 **ID:** 6379369

Potential Bias	Items to be considered for assessment of potential opportunity for bias	Yes	Partly	No	Unsure	NA*
Study population /sample selection	Inclusion and exclusion criteria are adequately described [including explicit diagnostic criteria, start/finish date of recruitment]	x				
	Baseline study sample [i.e. individuals entering the study and their key characteristics and sampling frame are adequately described]		x			
	Study sample represents population of interest on key characteristics, sufficient to limit potential bias to results	Moderate risk of bias				
Study attrition	Response rate (i.e., proportion of study sample completing the study and providing outcome data) is adequate	x				
	Participants lost to follow-up are adequately described for key characteristics					x
	Statement as to the possible effect on the results from missing data					x
	Loss to follow-up is not associated with key characteristics	Low risk of bias				
Prognostic factor measurement	Clear definition of the prognostic factors measured is provided (e.g. imaging modality method, measurement, and timing described).	x				
	Specified instrument and personnel for measurement of predictive factors	x				
	Continuous variables are reported or appropriate (i.e. not data- dependent) cut-off points are used and specified a priori	x				
	Blinding: were estimators of risk factor status and of outcomes blinded?				x	
	The prognostic factor(s) of interest is (are) adequately measured in study participants to sufficiently limit potential bias	Low risk of bias				
Outcome measurement	Is the outcome(s) clearly defined?		x			
	The outcome measure and method used are adequately valid and reliable to limit misclassification bias	Moderate risk of bias				
Study confounding	Do the authors address potential confounders?	x				
	Important potential confounders are appropriately accounted for, limiting potential bias with respect to the prognostic factor of interest.	Low risk of bias				
Analysis and reporting	There is sufficient presentation of data to assess the adequacy of the analysis strategy and there is no selective reporting	x				
	The statistical analysis is appropriate for the study design, limiting potential for the presentation of invalid results	Low risk of bias				
NA*: not applicable. Note: The above table was adapted from: Hayden et al., 2013.						
**Overall opinion of study quality= Low risk of bias**						
