# Supplementary material for: Prognostic accuracy of antenatal Doppler ultrasound for adverse perinatal outcomes in low-income and middle-income countries: a systematic review
Source: BMJ Open. 2021 Dec 2;11(12):e049799. doi: 10.1136/bmjopen-2021-049799 (PMC8640672; doi:10.1136/bmjopen-2021-049799)
Supplement: Supplementary data [file bmjopen-2021-049799supp006.pdf]

Table S2. Definitions of adverse perinatal outcomes reported in the selected studies

First Author	Outcomes	Definition (detailed description in the article)
Abdallah et al., 2019	LBW	Not defined
	NICU admission	Not defined
	Stillbirth	Not defined
	Perinatal mortality	Not defined
	Low APGAR score (1min & 5min)	Not defined
Agbaje et al., 2018	FGR	Abnormal birth weight: defined as estimated foetal weight below the 10th percentile for gestational age and abdominal circumference below the 10th percentile for gestational age.
	Low APGAR score at 5 minutes	APGAR score less than 6
Alanwar et al., 2018	Acidosis	Neonatal academia of pH < 7.2
	NICU admission	New-born was admitted to the neo- natal intensive care unit
	Low APGAR score at 5 minutes	APGAR score < 7 at 5 min
Allam et al., 2013	Neonatal acidosis	Cord blood pH <7.25
Anshul et al., 2010	Stillbirth	Not defined
	Neonatal death	Not defined
	NICU admission	Admission required
	Foetal distress	Delivered by emergency caesarean section for suspected foetal distress
	LBW	Not defined
	Low APGAR score at birth.	APGAR score <7 at birth
Bano et al., 2010	Perinatal death	Not defined
	Foetal distress	Caesarean section for foetal distress (FD not defined)
	NICU admission	Not defined
	Low APGAR score at 5min	APGAR score <7 at 5 min
	FGR	Birth weight less than 10th percentile for gestational age

	Composite adverse perinatal outcome	Not defined
Dhand et al., 2011	Composite adverse perinatal outcome	Abnormal foetal outcome (details not provided)
Dorman et al., 2002	Perinatal death	Not defined
	Preterm delivery	Delivery < 37 weeks
	LBW	Birth weight <2.5kg
Ebrashy et al., 2005	Acidosis	Neonatal acidaemia of pH<7.2 were present
	Composite adverse neonatal outcome	Neonatal morbidity (neonatal academia pH<7.2, 5-minute APGAR score <6, and/or admission to NICU)
Geerts et al., 2007	Composite adverse perinatal outcome	Poor outcome (perinatal demise or clinical/ultrasound signs of neurological compromise in the infant at the time of discharge from the tertiary institution)
Khanduri et al., 2013	FGR	Ponderal index was calculated as birth weight (in gm) per length (in cm^3). Ponderal index of <10 indicates growth restriction.
Kumari et al., 2019	Foetal anaemia	Haematocrit of the umbilical cord blood was used as the reference test to diagnose foetal anaemia (defined as haemoglobin <0.65 times the median for gestational age).
Lakhkar et al., 2006	Composite adverse perinatal outcome	Adverse perinatal outcome (Major and Minor). Major adverse outcomes were perinatal deaths including intrauterine and early neonatal deaths. Major complications like hypoxic ischemic encephalopathy, intraventricular haemorrhage, periventricular leukomalacia, pulmonary haemorrhage and necrotizing enterocolitis. Minor outcomes include-caesarean delivery for foetal distress, APGAR score below 7 at 5 minutes, admission to NICU (neonatal intensive care unit) for treatment.
Lakshmi et al., 2013	Neonatal death	Not defined
	Respiratory distress syndrome	Not defined
	Composite adverse perinatal outcome	Composite outcome of death or major neuro-morbidity at 12-18 months of corrected age, defined as presence of cerebral palsy or visual or hearing impairment.
Malik et al., 2013	Composite adverse perinatal outcome	Abnormal foetal outcome (IUGR, IUFD and perinatal mortality)
Masihi et al.2019	Intrapartum foetal distress	Emergency caesarean section for foetal distress
Mullick et al., 1993	FGR	Not defined
Nagar et al., 2015	FGR	Not defined
Najam et al., 2016	FGR	Not defined

	NICU admission	Not defined
	Foetal distress	Not defined
	Stillbirth	Not defined
	Neonatal death	Not defined
	Low APGAR score	Not defined
	Hypoxic ischemic encephalopathy	Not defined
	Meconium aspiration syndrome	Not defined.
Nouh et al., 2011	Composite adverse perinatal outcome	The presence of one or more of the following; miscarriage, gestational DM, PIH, PE, antepartum haemorrhage, intrauterine growth retardation, instrumental, caesarean delivery and preterm labour.
Pares et al., 2008	Foetal anaemia	Anaemia was considered moderate to severe when foetal haemoglobin concentrations were < or =0.64 multiples of the median for gestational age.
Pattinson et al., 1991	Composite adverse perinatal outcome	Poor foetal outcome (details not provided).
Pattinson et al., 1993	Composite adverse perinatal outcome	Complications of pregnancy, namely intra-uterine growth retardation and proteinuric hypertension.
Phupong et al., 2003	FGR	Birth weight less than 10 percentile for gestational age.
Rani et al., 2016	Composite adverse perinatal outcome	Adverse perinatal outcome was defined as any of these: small for gestational age, still birth, APGAR score <5 at 5 minutes, need of bag and mask ventilation for >10 minutes or hypoxic ischemic encephalopathy, admission to neonatal intensive care unit (NICU), and caesarean section due to non-reassuring foetal heart rate.
Rocca et al., 1995	IUGR	Not defined.
	Low APGAR score 5mins	APGAR score <7 at 5 minutes.
	Perinatal death	Not defined.
	Foetal distress	Emergency operative delivery for foetal distress.
Verma et al., 2016	FGR	Not defined.
	LBW	Birth weight <2500 gm.
	Preterm delivery	Spontaneous delivery <37 weeks.

	Composite adverse perinatal outcome	At least one adverse outcome (preeclampsia, FGR, low birth weight, spontaneous preterm delivery, oligohydramnios, foetal loss).
Waa et al., 2010	Composite adverse perinatal outcome	Poor outcome was defined by foetal mortality or appearance, pulse rate, grimace, activity, respiration (APGAR) score less than eight at five minutes or weight less than 10th percentile for gestation 20 or head circumference and length below 10th percentile for gestation.
Yelikar et al., 2013	Intrapartum foetal distress	Delivered by emergency caesarean section for suspected foetal distress.
Zarean et al., 2018	Composite adverse perinatal outcome	Adverse perinatal outcome, including preterm labour, intrauterine foetal death, PE, low 5-min APGAR score (<7), low umbilical arterial cord blood pH, admitted to Intensive Care Unit in the first 3 days of birth, low birth weight, infant with low weight, death of new-borns, caesarean section for respiratory distress, and meconial amniotic fluid.

[a]FGR: fetal growth restriction; FGR: intrauterine growth restriction; LBW: low birth weight; NICU: neonatal intensive care unit.
