# Supplementary material for: Prognostic accuracy of antenatal Doppler ultrasound for adverse perinatal outcomes in low-income and middle-income countries: a systematic review
Source: BMJ Open. 2021 Dec 2;11(12):e049799. doi: 10.1136/bmjopen-2021-049799 (PMC8640672; doi:10.1136/bmjopen-2021-049799)
Supplement: Author's manuscript [file bmjopen-2021-049799.draft_revisions.pdf]

BMJ Open

BMJ Open is committed to open peer review. As part of this commitment we make the peer review history of every article we publish publicly available.

When an article is published we post the peer reviewers' comments and the authors' responses online. We also post the versions of the paper that were used during peer review. These are the versions that the peer review comments apply to.

The versions of the paper that follow are the versions that were submitted during the peer review process. They are not the versions of record or the final published versions. They should not be cited or distributed as the published version of this manuscript.

BMJ Open is an open access journal and the full, final, typeset and author-corrected version of record of the manuscript is available on our site with no access controls, subscription charges or pay-per-view fees (http://bmjopen.bmj.com).

If you have any questions on BMJ Open's open peer review process please email info.bmjopen@bmj.com

BMJ Open

Prognostic accuracy of antenatal Doppler ultrasound for adverse perinatal outcomes in low- and middle-income countries: a systematic review

Journal:	*BMJ Open*
Manuscript ID	bmjopen-2021-049799
Article Type:	Original research
Date Submitted by the Author:	02-Feb-2021
Complete List of Authors:	ALI, SAM; Utrecht University, Julius Global Health, Julius Center for Health Sciences and Primary Care, University Medical Center Utrecht; Ernest Cook Ultrasound Research and Education Institute<br>Heuving, Simelina; Utrecht University, Julius Global Health, Julius Center for Health Sciences and Primary Care, University Medical Center Utrecht<br>Kawooya, Michael; Ernest Cook Ultrasound Research and Education Institute<br>Byamugisha , Josaphat; Makerere University, Department of Obstetrics and Gynecology<br>Grobbee, Diederick; Utrecht University, Julius Global Health, Julius Center for Health Sciences and Primary Care, University Medical Center Utrecht<br>Papageorghiou, Aris; University of Oxford, Nuffield Department of Women’s & Reproductive Health<br>Klipstein-Grobusch, Kerstin; Utrecht University, Julius Global Health, Julius Center for Health Sciences and Primary Care, University Medical Center Utrecht; University of the Witwatersrand, Division of Epidemiology and Biostatistics, School of Public Health, Faculty of Health Sciences<br>Rijken, Marcus; Utrecht University, Julius Global Health, Julius Center for Health Sciences and Primary Care, University Medical Center Utrecht; Universitair Medisch Centrum Utrecht, Department of Obstetrics and Gynecology
Keywords:	Ultrasound < RADIOLOGY & IMAGING, Prenatal diagnosis < OBSTETRICS, Ultrasonography < OBSTETRICS, Fetal medicine < OBSTETRICS

SCHOLARONE™
Manuscripts

I, the Submitting Author has the right to grant and does grant on behalf of all authors of the Work (as defined in the below author licence), an exclusive licence and/or a non-exclusive licence for contributions from authors who are: i) UK Crown employees; ii) where BMJ has agreed a CC-BY licence shall apply, and/or iii) in accordance with the terms applicable for US Federal Government officers or employees acting as part of their official duties; on a worldwide, perpetual, irrevocable, royalty-free basis to BMJ Publishing Group Ltd ("BMJ") its licensees and where the relevant Journal is co-owned by BMJ to the co-owners of the Journal, to publish the Work in this journal and any other BMJ products and to exploit all rights, as set out in our licence.

The Submitting Author accepts and understands that any supply made under these terms is made by BMJ to the Submitting Author unless you are acting as an employee on behalf of your employer or a postgraduate student of an affiliated institution which is paying any applicable article publishing charge ("APC") for Open Access articles. Where the Submitting Author wishes to make the Work available on an Open Access basis (and intends to pay the relevant APC), the terms of reuse of such Open Access shall be governed by a Creative Commons licence – details of these licences and which Creative Commons licence will apply to this Work are set out in our licence referred to above.

Other than as permitted in any relevant BMJ Author's Self Archiving Policies, I confirm this Work has not been accepted for publication elsewhere, is not being considered for publication elsewhere and does not duplicate material already published. I confirm all authors consent to publication of this Work and authorise the granting of this licence.

For peer review only

Prognostic accuracy of antenatal Doppler ultrasound for adverse perinatal outcomes in low- and middle-income countries: a systematic review

Sam Ali[1,2*], Simelina Heuving[1], Michael G. Kawooya[2], Josaphat Byamugisha[3], Diederick E. Grobbee[1], Aris T. Papageorghiou[4], Kerstin Klipstein-Grobusch[1,5], Marcus J. Rijken[1,6]

[1]Julius Global Health, Julius Center for Health Sciences and Primary Care, University Medical Center Utrecht, Utrecht University, Utrecht, The Netherlands.

[2]Ernest Cook Ultrasound Research and Education Institute (ECUREI), Mengo Hospital, Kampala, Uganda.

[3]Department of Obstetrics and Gynecology, Makerere University College of Health Sciences, Kampala, Uganda.

[4]Nuffield Department of Women's and Reproductive Health, John Radcliffe Hospital, University of Oxford, Oxford, United Kingdom.

[5]Division of Epidemiology and Biostatistics, School of Public Health, Faculty of Health Sciences, University of the Witwatersrand, Johannesburg, South Africa.

[6]Department of Obstetrics and Gynecology, University Medical Center Utrecht, Utrecht University, Utrecht, Netherlands.

Corresponding author:

Sam Ali

Department of research, Ernest Cook Ultrasound Research and Education Institute (ECUREI), Mengo Hospital. Sir Albert Cook Road. P.O. Box 7161, Kampala, Uganda.

Email: S.Ali-2@umcutrecht.nl or alisambecker@gmail.com

Word count: 2540

For peer review only - http://bmjopen.bmj.com/site/about/guidelines.xhtml

ABSTRACT

Objectives This systematic review examined available literature on the prognostic accuracy of Doppler ultrasound for adverse perinatal outcomes in LMIC.

Design We searched PubMed, Embase, Cochrane Library and Scopus from inception to April 2020.

Setting Observational or interventional studies from low- and middle-income countries

Participants Singleton pregnancies of any risk profile.

Interventions Umbilical artery (UA), middle cerebral artery (MCA), cerebroplacental ratio (CPR), uterine artery (UtA), fetal descending aorta (FDA), ductus venosus, umbilical vein, and inferior vena cava.

Primary and secondary outcome measures. Perinatal death, stillbirth, neonatal death, expedited delivery for fetal distress, meconium-stained liquor, low birth weight, fetal growth restriction (FGR), admission to neonatal intensive care unit, neonatal acidosis, Apgar scores, preterm birth, fetal anemia, respiratory distress syndrome, length of hospital stay, birth asphyxia and composite adverse perinatal outcomes.

Results We identified 2825 records, and 30 (including 4977 women) from Africa (40.0%, n= 12), Asia (56.7%, n= 17) and South America (3.3%, n= 01) were included. UA Doppler had good predictive values for perinatal death (Odds ratio 9.8, 95% confidence interval 2.1- 46.4) and FGR (positive predictive value (PPV) of 77.40 to 88.5). UA, MCA, CPR and UtA Dopplers had moderate to high predictive values for

For peer review only

composite adverse perinatal outcomes. MCA and FDA were potent predictors of fetal anemia (sensitivity: 86.0% - 98.4% and PPV: 86.0 - 100%). No randomized clinical trial was found. Most studies were of sub-optimal quality, poorly powered and characterized by wide variations in outcome classifications, timing for the Doppler tests and study populations.

Conclusion Local evidence to guide how antenatal Doppler ultrasound should be used in LMIC is lacking. Well-designed studies, preferably randomized clinical trials, are required. Standardization of practice and classification of perinatal outcomes across countries, in accordance with international standards, is imperative.

Keywords Pregnancy, ultrasound, prenatal diagnosis, prenatal care, developing countries, and systematic review.

Article Summary

Strengths and limitations of this study

- This systematic review used the most optimal database combinations and snowballing technique with no time restrictions to identify the records.
- We comprehensively examined available literature on the prognostic accuracy of antenatal Doppler ultrasound in low and middle-income countries.
- Although only English language articles were included, it is unlikely that high impact papers were not identified.
- Pooling and interpreting the data for wider clinical application was difficult due to the large heterogeneity across studies.

INTRODUCTION

For peer review only - http://bmjopen.bmj.com/site/about/guidelines.xhtml

Stillbirths remain a major global challenge,[1] with nearly three million cases reported annually.[2] The vast majority of the cases (98%) are contributed by low- and middle-income countries (LMIC).[3] These deaths have profound effects on the families and communities involved, and strategies for reduction are of high societal importance. The risk of adverse perinatal outcomes is higher in compromised fetuses than in normally growing babies, and is distinguishable using antenatal Doppler ultrasound.[4,5] Prenatal diagnosis of fetuses at risk provides a window for close monitoring and/or expedited delivery of well-developed babies with the prospect of improving survival and long-term wellbeing.[4]

The predictive performance of Doppler ultrasound for adverse perinatal outcomes has been demonstrated in primary studies, systematic reviews and meta-analysis from high-income countries (HIC), guiding the development HIC practice guidelines.[6] We believe the use of HIC guidelines for clinical guidance in LMIC is inappropriate given the differences in the prevalence of adverse pregnancy outcomes in the two settings. For instance, the stillbirth rates per 1000 total births (95% confidence interval) in HIC is 3.4 (3.4-3.5), Southern Asia 25.5 (22.5-29.1) and 28.7 (25.1-34.2) in sub-Saharan Africa.[2] Since the prevalence and severity of disease influences the diagnostic or prognostic test performance, context specific guidance is necessary.[7] However, there are still knowledge gaps about the predictive ability of antenatal Doppler for adverse pregnancy outcomes in LMIC.

This systematic review examined existing literature on the prognostic accuracy of Doppler ultrasound for adverse perinatal outcomes in LMIC. The implications for clinical

For peer review only

utility of the available local evidence to guide practice in LMIC are highlighted.

MATERIAL AND METHODS

Protocol and registration

This systematic review protocol was registered in the PROSPERO database: CRD42019128546, and reported in accordance with the Preferred Reporting Items for a Systematic Review and Meta-analysis of Diagnostic Test Accuracy Studies: The PRISMA-DTA Statement.[8]

Eligibility criteria

We included observational (cohort or case control) studies and randomized clinical trials (RCTs) from LMIC (as per the World Bank country classifications in the year 2020) reporting the prognostic value of Doppler ultrasound for adverse perinatal outcomes in singleton pregnancies of any risk profile. Doppler measurements of interest included umbilical artery (UA), middle cerebral artery (MCA), cerebroplacental ratio (CPR), uterine artery (UtA), fetal descending aorta (FDA), ductus venosus (DV), umbilical vein (UV) and inferior vena cava (IVC). Adverse perinatal outcomes (as defined in the included studies) were perinatal death, stillbirth, neonatal death, expedited delivery for fetal distress, meconium-stained liquor, low birth weight, fetal growth restriction (FGR), admission to neonatal intensive care unit (NICU), neonatal acidosis, Apgar scores, preterm birth, fetal anemia, respiratory distress syndrome (RDS), length of hospital stay, birth asphyxia, and composite adverse perinatal outcomes (CAPO). Conference proceedings/posters that did not appear as full text papers, case reports and review articles without original data were excluded.

Information sources and search

For peer review only - http://bmjopen.bmj.com/site/about/guidelines.xhtml

We conducted comprehensive literature search in PubMed (Medline), Embase, Cochrane Library and Scopus for articles published from inception to April 07, 2020. The search strategies (online supplementary appendix S1) were developed with the support of a librarian at University Medical Center Utrecht. When applicable, pre-defined search (Title/Abstract) and MeSH/Emtree terms were used. No limits were applied to the searches.

Study selection

The records retrieved from the databases were exported to Endnote to eliminate duplicates and then transferred to Rayyan for review and selection. Two reviewers (SA and SH) independently assessed all studies for inclusion based on title and abstract. Studies reporting any Doppler parameter and adverse pregnancy outcome of interest in the title or abstract were further retrieved in full text and assessed by the same two reviewers against full eligibility criteria. Disagreements were resolved by discussion or, if required, we consulted the third review author (MJR).

Data extraction

Using a pre-piloted data extraction sheet, two reviewers (SA and SH) independently extracted data on authors, study title, year of publication, study period, number of women recruited, gestational age at Doppler ultrasound exam, method of pregnancy dating, pregnancy risk profile, blood vessels studied, pregnancy outcomes, and key results. If any relevant information was missing, the corresponding authors were contacted once by e-mail.

Risk of bias assessment

Two raters (SA and SH) independently evaluated the risk of bias for each study using the quality in prognostic studies (QUIPS) tool.[9] The risk of bias domains included study population, attrition, prognostic factor measurement,

For peer review only

outcome measurement, confounding and statistical analysis. All the domains were separately judged by two raters as having low, moderate or high risk of bias. Any disagreement during this process was resolved by contacting the third rater (MJR).

Prognostic test accuracy measures

Doppler test prognostic performance measures, as reported in the selected studies, are presented in table S1. These included diagnostic test accuracy measures such as sensitivity, specificity, positive predictive values (PPV) and negative predictive values (NPV); measures of association; proportions; and correlations.

Data synthesis and analysis

The results were narratively summarized. The large heterogeneity in the study populations, timing for Doppler tests, outcome definitions and prognostic performance measures in the included studies did not allow for a meta-analysis. If a study reported multiple Doppler indices, the most commonly used (pulsatility index) was selected.

RESULTS

Study selection

The 2825 records we identified through electronic searches reduced to 2210 after removal of duplicates, and 2162 were further excluded based on title and abstract screening, retaining 48 records. After full-text assessment for eligibility, 23 studies were excluded with reasons, and 25 remained (online supplementary appendix S2). Five additional records were identified through snowballing (Figure 1). Thirty studies, involving a total count of 4977 women and median (interquartile range) sample size of 100 (30, 181) were included in the analysis (table 1).

Study characteristics

For peer review only - http://bmjopen.bmj.com/site/about/guidelines.xhtml

The selected studies were from Africa (40.0%, n = 12), Asia 17 (56.7%, n = 17) and South America (3.3%, n = 01). Twenty studies (67%) recruited high-risk pregnancies, six (16.7%) both high and low-risk populations, while five (16.7%) studied the low-risk group (online supplementary appendix S3). Thirteen (43.3%) studies did not specify a method of pregnancy dating, 13 (43.3%) assessed gestational age using last menstrual period (LMP) combined with ultrasound, three (10.0%) used ultrasound alone, and one (3.3%) study used LMP. No RCTs was identified, and no study provided data on the UV and IVC Doppler (table 1).

Methodological quality of included studies

The results of the QUIPS assessment are provided in Figure 2 and online supplementary appendix S4. Overall, the risk of bias was low in 15 (50%), moderate in 10 (33.3%), and high in five (16.7%) studies. In the study population domain, the risk of bias was low in 73.3%, moderate in 23.3%, and high in 3.3% of the studies. Selective reporting remarkably resulted in moderate to high-risk of bias for analysis and reporting in 20 (66.7%) studies. We found moderate to high-risk of bias for outcome measurement in 17 (56.7%) studies, mostly due to inconsistencies in outcome classifications (online supplementary table S2).

Prognostic accuracy of antenatal Doppler ultrasound for adverse perinatal outcomes

Twenty studies evaluated the umbilical artery,[10,11,20-29,12-19] and seven reported its predictive values for FGR. The positive predictive values for FGR were between 77.40 and 88.5,[11,16,21,24] while area under the receiver operating characteristic (AU ROC) curve was 0.63,[17] mostly in high-risk pregnancies. The NPV ranged from 55.4 - 95.65.[11,16,21,24] FGR was defined as birth weight or abdominal circumference below the 10th

For peer review only

percentile in two studies,[11,17] ponderal index less than 10 in one study,[21] and was not defined in the remaining studies.[16,24,26] Increased flow impedance in the UA had positive predictive values for composite adverse outcome between 66.60 and 96.6 in high-risk pregnancies.[11,13,19,23] All studies provided individual components of the CAPO except only one.[11] Absent or reversed end-diastolic flow (AREDF) in the UA was associated with poor pregnancy outcomes (perinatal death: odds ratio (OR) 9.8, 95% confidence interval (CI) 2.1 to 46.4; CAPO: OR 2.4, 95% CI 1.1 to 5.0; and RDS: OR 8.4, 95% CI 2.3 to 30.5).[14,22,26]

The MCA was reported in 12 studies.[11,12,31,32,13,15,19,21,23,26,28,30] The positive predictive values for fetal anemia in Rhesus (Rh) isoimmunized pregnancies requiring transfusion were between 83.0 - 90.9 and the AU ROC curve was 0.7.[12,32] Fetal anemia was consistently defined as hemoglobin (Hb)=< 0.64 g/dl in the two studies, though they recruited low numbers of women.[12,32] MCA Doppler had a sensitivity of 87.5%, PPV of 74.0% and AU ROC curve of 0.82 for neonatal acidosis.[30] The positive predictive values for CAPO ranged from 80.0-100% in high-risk pregnancies,[11,13,19,23,31] but two studies did not provide details of the individual components of the CAPO.[11,31]

Nine studies reported the prognostic value of CPR.[11,13,15,19,20,23,26,33,34] CPR showed promising predictive value for adverse perinatal outcomes in unselected pregnancies in the third trimester. One study reported sensitivity 85.10, specificity 89.72, PPV 80.70 and NPV 92.30 for FGR.[26] Two studies found sensitivity between 80.90 and 90.91%, and specificity between 50.0 and

For peer review only - http://bmjopen.bmj.com/site/about/guidelines.xhtml

78.04% for emergency caesarean section for fetal distress though the tests had poor positive predictive values.[26,34] Abnormal CPR had positive predictive values for CAPO between 81.80 and 100% in high-risk pregnancies.[11,13,15,23]

Eight studies reported the prognostic value of UtA Doppler,[14,23,25,35–39] and two showed positive predictive values of over 91.8% for CAPO in high-risk pregnancies.[23,36] The remaining studies had poor predictive values for adverse perinatal outcomes.

Three studies evaluated the prognostic accuracy of FDA Doppler.[12,13,32] The FDA sensitivity for fetal anemia in Rh isoimmunized pregnancies ranged from 87.0% to 95.7% when used in isolation.[12,32] The sensitivity varied between 86.0% and 98.4% and positive predictive values ranged from 86.0- 100% when combined with the MCA.[12,32]

The DV was sampled in two studies undertaken in high-risk pregnancies.[20,30] Abnormal DV had a sensitivity of 100, PPV of 72.0 and AU ROC curve of 0.88 for the prediction of neonatal acidosis, though this study included only 30 women between 36-41 weeks of gestation.[30] It had borderline significance (OR 0.379, 95% CI 0.03 to 4.63), and a positive predictive value of 92.0% for the prediction of composite adverse perinatal outcomes at 24-34 weeks of gestation.[20]

DISCUSSION

Our results indicate that abnormal UA Doppler is associated with poor perinatal outcomes, mostly in high-risk pregnancies. Abnormal UA, MCA, CPR and UtA Dopplers had moderate to high predictive values for composite adverse perinatal outcomes. Abnormal MCA Doppler had high individual predictive value for fetal anemia, but performed

For peer review only - http://bmjopen.bmj.com/site/about/guidelines.xhtml

better when combined with the FDA. However, the majority of the available evidence were of sub-optimal quality, based on a few poorly powered studies and had no RCTs. Further, wide variations in the populations studied, definitions of adverse perinatal outcomes and prognostic accuracy measures across studies was present. Thus, pooling and interpreting the evidence for wider clinical application was difficult.

A strength of this systematic review is that it was conducted according to a registered protocol, using the most optimal database combinations and snowballing with no time restrictions. Although we only included English language articles, it is unlikely that high impact papers were not identified. About half of the studies included in the analysis were of poor quality, and 20 (66.7%) studies selectively reported results potentially raising the risk of reporting bias. A meta-analysis was not possible due to large heterogeneity in the study populations, definition of adverse perinatal outcomes and prognostic accuracy measures across studies.

Evidence from HIC suggest that adding Doppler studies into clinical diagnostic or prognostic rules improves pregnancy risk assessment,[6] and are increasingly becoming integrated into their pregnancy management guidelines.[4,6] The use of guidance based entirely on HIC data in daily practice in LMIC could be misleading considering the differences in the adverse outcome rates in the two settings. The stillbirth rates in LMIC is approximately 10 times that of HIC,[2] a large variation likely to influence the predictive performance of diagnostic or prognostic tests.[7] Thus, a proper understanding of existing literature from LMIC is important. This paper reports the findings of a systematic review of primary evidence on the

For peer review only - http://bmjopen.bmj.com/site/about/guidelines.xhtml

prognostic value of antenatal Doppler ultrasound for adverse perinatal outcomes in LMIC.

Abnormal blood flow patterns in the UA had moderate to high predictive values for FGR and was associated with poor outcomes in high-risk pregnancies. Similarly, a recent Cochrane review of RCTs from HIC suggests that using UA Doppler in high-risk pregnancies could reduce perinatal deaths by 30% (risk ratio 0.71, 95% CI 0.52 to 0.98), and lead to fewer obstetric interventions.[40] Despite some similarities with our findings, the definitions of adverse outcomes, including FGR were inconsistent (across studies included in this review) with agreed international standards,[4,41] with no clear distinction between early and late FGR. Scanty data from this review indicate that abnormal CPR, UA, MCA and UtA Doppler could be predictive of CAPO. However, in a previous systematic review from HIC, CPR had low predictive accuracy (pooled sensitivity: 57%, specificity: 77%, and summary positive likelihood ratio (LR): 2.5, and negative LR: 0.60) for CAPO in pregnancies with suspected FGR antenatally.[42] In another review, CPR was significantly better than UA and MCA Doppler in predicting CAPO ($P < 0.001$) and emergency delivery for fetal distress in singleton pregnancies of all risk profiles,[43] but the primary studies reviewed had numerous methodological limitations.[43] Further, first trimester UtA Doppler had very low sensitivity 25.8% (95% CI 15.5 to 39.7) for CAPO in a systematic review of 18 studies (involving 55974 women).[44] More data from HIC indicate that MCA-PSV reliably predicts fetal anemia in un-transfused fetuses.[45] The area under the hierarchical summary ROC curve for moderate-severe anemia in untransfused fetuses was 87%, pooled sensitivity 86% (95% CI 75 to 93%) and specificity 71% (95% CI 49 to 87%).[45]

For peer review only - http://bmjopen.bmj.com/site/about/guidelines.xhtml

Similarly, in our study, MCA alone or when combined with FDA had high predictive values for fetal anemia in Rh isoimmunized pregnancies, but this was based on only three studies. Over all, this review found that high quality studies on the predictive accuracy of Doppler ultrasound for adverse perinatal outcomes in LMIC are scare. The large heterogeneity across studies precluded a meta-analysis and between study comparisons.

Future studies need to specify the methods and timing for pregnancy dating. Accurate dating is crucial for timing the Doppler tests and interventions to expedite delivery in compromised fetuses. The interpretation and comparison of Doppler studies could be improved by using standard outcome definitions and completeness in reporting.[46] Most primary studies in this review studied the predictive ability of a single variable (Doppler test) for the outcome(s) of interest, without considering existing characteristics of clinical importance to estimate pregnancy risk. The predictive accuracies of new determinants need to be assessed individually and by multivariable analysis to facilitate the clinical applicability of the findings. The clinical applicability of Doppler ultrasound also depends on the clinical judgement of the Doppler measurements and the feasibilities of local healthcare systems to interpret and respond to the results of the Doppler scan. Along the same line, our recently concluded prospective cohort study in a sub-Saharan African setting will soon highlight the prognostic value of Doppler ultrasound in the late third trimester, and the feasibilities of integrating such advanced technologies into routine antenatal care in LMIC.

This review demonstrates that a scientific basis to provide evidence for how antenatal Doppler should be used in LMIC is lacking. Well-designed studies, preferably

For peer review only - http://bmjopen.bmj.com/site/about/guidelines.xhtml

randomized clinical trials, testing application models of antenatal Doppler while respecting the local conditions are needed. Moreover, local practice and classification of perinatal outcomes needs to be standardized, utilizing approaches consistent with international consensus.

Acknowledgements

We thank the University Medical Center Utrecht librarians Paulien Wiersma and Felix. We thank the team from Makerere University College of Health Sciences and ECUREI that supported us to undertake this review.

Author contributions

SA, SH, KKG, and MJR drafted the protocol and conducted the review. MGK, JB, DEG, and ATP critically reviewed the work for important intellectual content. All the authors approved the final manuscript.

Funding

This research was supported by the UMC Utrecht Global Health Support Program (Ref: FM/ADB/D-18-015006), University Medical Center Utrecht, Utrecht, The Netherlands, and Grand Challenges Canada (Grant Number: R-ST-POC-1808-17038).

Competing interests

None

Data sharing statement

No additional data are available.

REFERENCES

For peer review only

1 de Bernis L, Kinney M V, Stones W, *et al.* Stillbirths: ending preventable deaths by 2030. *Lancet* 2016;**387**:703–16. doi:10.1016/S0140-6736(15)00954-X
2 Lawn JE, Blencowe H, Waiswa P, *et al.* Stillbirths: Rates, risk factors, and acceleration towards 2030. *Lancet* 2016;**387**:587–603. doi:10.1016/S0140-6736(15)00837-5
3 Reinebrant HE, Leisher SH, Coory M, *et al.* Making stillbirths visible: a systematic review of globally reported causes of stillbirth. *BJOG An Int J Obstet Gynaecol* 2018;**125**:212–24. doi:10.1111/1471-0528.14971
4 Lees CC, Stampalija T, Baschat A, *et al.* ISUOG Practice Guidelines: diagnosis and management of small-for-gestational-age fetus and fetal growth restriction. *Ultrasound Obstet Gynecol* 2020;**56**:298–312. doi:10.1002/uog.22134
5 Figueras F, Caradeux J, Crispi F, *et al.* Diagnosis and surveillance of late-onset fetal growth restriction. *Am J Obstet Gynecol* 2018;**218**:S790-S802.e1. doi:10.1016/j.ajog.2017.12.003
6 McCowan LM, Figueras F, Anderson NH. Evidence-based national guidelines for the management of suspected fetal growth restriction: comparison, consensus, and controversy. *Am J Obstet Gynecol* 2018;**218**:S855–68. doi:10.1016/j.ajog.2017.12.004
7 Grobbee DE, Hoes AW. *Clinical Epidemiology: Principles, Methods, and Applications for Clinical Research*. Jones & Bartlett Publishers 2014.
8 McInnes MDF, Moher D, Thombs BD, *et al.* Preferred Reporting Items for a Systematic Review and Meta-analysis of Diagnostic Test Accuracy Studies: The PRISMA-DTA Statement. *JAMA* 2018;**319**:388–96. doi:10.1001/jama.2017.19163
9 Hayden JA, van der Windt DA, Cartwright JL, *et al.* Assessing Bias in Studies of Prognostic Factors. *Ann Intern Med* 2013;**158**:280–6. doi:10.7326/0003-4819-158-4-201302190-00009
10 Abdallah A, Eldorf A, Sallam S, *et al.* Nuchal cord: impact of umbilical artery Doppler indices on intrapartum and neonatal outcomes: a prospective cohort study. *J Matern Neonatal Med* 2019;**32**:3367–78. doi:10.1080/14767058.2018.1463984
11 Bano S, Chaudhary V, Pande S, *et al.* Color doppler evaluation of cerebral-umbilical pulsatility ratio and its usefulness in the diagnosis of intrauterine growth retardation and prediction of adverse perinatal outcome. *Indian J Radiol Imaging* 2010;**20**:20–5. doi:10.4103/0971-3026.59747
12 Kumari S, Deka D, Dadhwal V, *et al.* Correlation of

For peer review only - http://bmjopen.bmj.com/site/about/guidelines.xhtml

fetal blood vessel Doppler measurements with fetal anemia among Rhesus isoimmunized pregnancies after two intrauterine transfusions. *Int J Gynecol Obstet* 2019;**146**:218–22. doi:10.1002/ijgo.12825

13 Lakhkar BN, Rajagopal K V, Gourisankar PT. Doppler prediction of adverse perinatal outcome in PIH and IUGR. *Indian J Radiol Imaging* 2006;**16**:109.

14 Pattinson RC, Brink AL, De Wet PE, *et al.* Early detection of poor fetal prognosis by serial Doppler velocimetry in high-risk pregnancies. *South African Med J* 1991;**80**:428–31.

15 Rani S, Huria A, Kaur R. Prediction of perinatal outcome in preeclampsia using middle cerebral artery and umbilical artery pulsatility and resistance indices. *Hypertens Pregnancy* 2016;**35**:210–6. doi:10.3109/10641955.2015.1137585

16 Rocca MM, Said MS, Khamis MY, *et al.* The Value of Doppler Study of the Umbilical Artery in Predicting Perinatal Outcome in Pre-Eclamptic Patients. *J Obstet Gynaecol (Lahore)* 1995;**21**:427–31. doi:10.1111/j.1447-0756.1995.tb01032.x

17 Agbaje OA, Adeyomoye AAO, Omidiji OAT, *et al.* Evaluation of Umbilical Artery Doppler Indices in Pregnant Women With Sickle Cell Anemia Disease at a Nigerian Tertiary Hospital. *J Diagnostic Med Sonogr* 2018;**34**:466–78. doi:10.1177/8756479318791157

18 Anshul D, Neelu S, Suneeta G. Significance of umbilical artery Doppler velocimetry in the perinatal outcome of the growth restricted fetuses. *J Obstet Gynecol India* 2010;**60**:38–43. doi:10.1007/s13224-010-0007-2

19 Ebrashy A, Azmy O, Ibrahim M, *et al.* Middle cerebral/umbilical artery resistance index ratio as sensitive parameter for fetal well-being and neonatal outcome in patients with preeclampsia: case-control study. *Croat Med J* 2005;**46**.

20 Geerts L, Odendaal HJ. Severe early onset pre-eclampsia: prognostic value of ultrasound and Doppler assessment. *J Perinatol* 2007;**27**:335–42. doi:10.1038/sj.jp.7211747

21 Khanduri S, Parashari UC, Bashir S, *et al.* Comparison of diagnostic efficacy of umbilical artery and middle cerebral artery waveform with color Doppler study for detection of intrauterine growth restriction. *J Obstet Gynaecol India* 2013;**63**:249–55. doi:10.1007/s13224-012-0326-6

22 Lakshmi CVS, Pramod G, Geeta K, *et al.* Outcome of very low birth weight infants with abnormal antenatal doppler flow patterns: A prospective cohort study.

Indian Pediatr 2013;**50**:847–52. doi:10.1007/s13312-013-0235-5

23 Malik R, Saxena A. Role of Colour Doppler Indices in the Diagnosis of Intrauterine Growth Retardation in High-Risk Pregnancies. *J Obstet Gynecol India* 2013;**63**:37–44. doi:10.1007/s13224-012-0210-4

24 Mullick S, Kushtagi P, Swain UK, *et al.* Doppler waveform patterns of the umbilical artery — screening test for high risk pregnancies. *Int J Gynecol Obstet* 1993;**40**:115–8. doi:10.1016/0020-7292(93)90369-8

25 Nagar T, Sharma D, Choudhary M, *et al.* The Role of Uterine and Umbilical Arterial Doppler in High-risk Pregnancy: A Prospective Observational Study from India. *Clin Med Insights Reprod Heal* 2015;**9**:CMRH.S24048. doi:10.4137/CMRH.S24048

26 Najam R, Gupta S, Shalini. Predictive Value of Cerebroplacental Ratio in Detection of Perinatal Outcome in High-Risk Pregnancies. *J Obstet Gynecol India* 2016;**66**:244–7. doi:10.1007/s13224-015-0671-3

27 Pattinson RC, Norman K, Odendaal HJ. The use of Doppler velocimetry of the umbilical artery before 24 weeks' gestation to screen for high-risk pregnancies. *South African Med J* 1993;**83**:734–6.

28 Waa S, Vinayak S. Comparision of doppler studies in obstetrics with foetal outcome. *East Afr Med J* 2010;**87**:502–8.

29 Yelikar KA, Prabhu A, Thakre GG. Role of Fetal Doppler and Non-Stress Test in Preeclampsia and Intrauterine Growth Restriction. *J Obstet Gynecol India* 2013;**63**:168–72. doi:10.1007/s13224-012-0322-x

30 Allam IS, Abuelghar W, Fathy H, *et al.* Prediction of neonatal acidosis by ductus venosus Doppler pattern in high risk pregnancies. *Middle East Fertil Soc J* 2013;**18**:47–52. doi:https://doi.org/10.1016/j.mefs.2012.10.003

31 Dhand H, Kansal HK, Dave A. Middle cerebral artery Doppler indices better predictor for fetal outcome in IUGR. *J Obstet Gynecol India* 2011;**61**:166–71. doi:10.1007/s13224-011-0018-7

32 Pares D, Chinen PA, Camano L, *et al.* Prediction of fetal anemia by Doppler of the middle cerebral artery and descending thoracic aorta. *Arch Gynecol Obstet* 2008;**278**:27–31. doi:10.1007/s00404-007-0522-2

33 Alanwar A, El Nour AA, El Mandooh M, *et al.* Prognostic accuracy of cerebroplacental ratio for adverse perinatal outcomes in pregnancies complicated with severe pre-eclampsia; a prospective cohort study. *Pregnancy Hypertens* 2018;**14**:86–9. doi:https://doi.org/10.1016/j.preghy.2018.08.446

34 Masihi S, Nikbakht R, Barati M, *et al.* Association Between Fetal Middle Cerebral Artery and Umbilical Artery Doppler Ratio with Fetal Distress in 38–40 Weeks of Gestation. *J Obstet Gynecol India* 2019;**69**:509–13. doi:10.1007/s13224-019-01250-2
35 Dorman EK, Shulman CE, Kingdom J, *et al.* Impaired uteroplacental blood flow in pregnancies complicated by falciparum malaria. *Ultrasound Obstet Gynecol* 2002;**19**:165–70. doi:10.1046/j.0960-7692.2001.00545.x
36 Nouh AA, Shalaby SM. The predictive value of uterine blood flow in detecting the risk of adverse pregnancy outcome in patients with polycystic ovary syndrome. *Middle East Fertil Soc J* 2011;**16**:284–90. doi:https://doi.org/10.1016/j.mefs.2011.09.001
37 Phupong V, Dejthevaporn T, Tanawattanacharoen S, *et al.* Predicting the risk of preeclampsia and small for gestational age infants by uterine artery Doppler in low-risk women. *Arch Gynecol Obstet* 2003;**268**:158–61. doi:10.1007/s00404-002-0361-0
38 Verma D, Gupta S. Prediction of adverse pregnancy outcomes using uterine artery Doppler imaging at 22-24 weeks of pregnancy: A North Indian experience. *Turkish J Obstet Gynecol* 2016;**13**:80–4. doi:10.4274/tjod.55632
39 Zarean E, Shabaninia S. The Assessment of Association between Uterine Artery Pulsatility Index at 30-34 Week's Gestation and Adverse Perinatal Outcome. *Adv Biomed Res* 2018;**7**:111. doi:10.4103/abr.abr_112_17
40 Alfirevic Z, Stampalija T, Dowswell T. Fetal and umbilical Doppler ultrasound in high-risk pregnancies. *Cochrane Database Syst Rev* 2017;**2017**:CD007529. doi:10.1002/14651858.CD007529.pub4
41 Gordijn SJ, Beune IM, Thilaganathan B, *et al.* Consensus definition of fetal growth restriction: a Delphi procedure. *Ultrasound Obstet Gynecol* 2016;**48**:333–9. doi:10.1002/uog.15884
42 Conde-Agudelo A, Villar J, Kennedy SH, *et al.* Predictive accuracy of cerebroplacental ratio for adverse perinatal and neurodevelopmental outcomes in suspected fetal growth restriction: systematic review and meta-analysis. *Ultrasound Obstet Gynecol* 2018;**52**:430–41. doi:10.1002/uog.19117
43 Vollgraff Heidweiller-Schreurs CA, De Boer MA, Heymans MW, *et al.* Prognostic accuracy of cerebroplacental ratio and middle cerebral artery Doppler for adverse perinatal outcome: systematic review and meta-analysis. *Ultrasound Obstet Gynecol* 2018;**51**:313–22. doi:10.1002/uog.18809
44 Velauthar L, Plana MN, Kalidindi M, *et al.* First-trimester uterine artery Doppler and adverse pregnancy

For peer review only - http://bmjopen.bmj.com/site/about/guidelines.xhtml

outcome: a meta-analysis involving 55 974 women. *Ultrasound Obstet Gynecol* 2014;**43**:500–7. doi:https://doi.org/10.1002/uog.13275

45 Martinez-Portilla RJ, Lopez-Felix J, Hawkins-Villareal A, *et al.* Performance of fetal middle cerebral artery peak systolic velocity for prediction of anemia in untransfused and transfused fetuses: systematic review and meta-analysis. *Ultrasound Obstet Gynecol* 2019;**54**:722–31. doi:10.1002/uog.20273

46 Duffy JMN, Rolph R, Gale C, *et al.* Core outcome sets in women's and newborn health: a systematic review. *BJOG An Int J Obstet Gynaecol* 2017;**124**:1481–9. doi:https://doi.org/10.1111/1471-0528.14694

For peer review only

LEGENDS

Online supplementary data legends

Appendix S1. Search strings for the databases used to retrieve articles.

Appendix S2. List of full-text articles excluded with reasons.

Appendix S3. Pregnancy risk profiles in the selected studies

Appendix S4. Risk of bias assessment results of the 30 studies included in the analysis.

Table S1. Statistical measures of prognostic performance of Doppler ultrasound reported in the selected studies.

Table S2. Definitions of adverse perinatal outcomes reported in the selected studies

For peer review only - http://bmjopen.bmj.com/site/about/guidelines.xhtml

Figures legends

Figure 1. PRIMA flow diagram

Figure 2. Risk of bias assessment results of the 30 included studies

Figure 2 key

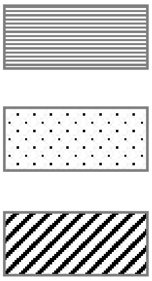

Low-risk of bias

Moderate-risk of bias

High-risk of bias

Table legends

Table 1 Summary of studies included in the systematic review of current evidence on the prognostic value of Doppler ultrasound for predicting adverse pregnancy outcomes in LMIC.

For peer review only

Table 1

Author, Year	Country	Study Period	Women	Weeks	Study Design	Dating method	Vessels	Abnormal Doppler Thresholds
Abdallah, 2019.[10]	Egypt	2015-2017	92	>= 37	Cohort	LMP or first trimester ultrasound	UA	UA (RI, PI and S/D ratio) > 95th centile
Agbaje, 2018.[17]	Nigeria	2014-2015	120	26	Cohort	LMP and/or early dating sonograms	UA	S/D ratio > 95th percentile, RI > 95th percentile, and AREDF.
Alanwar, 2018.[33]	Egypt	2017	100	30 - 40	Cohort	Not specified	CPR	CPR PI < 1 or CPR PI < 5th percentile.
Allam, 2013.[30]	Egypt	2007-2010	30	36 - 41	Cohort	Not specified	MCA, DV	MCA S/D ratio <4.37, DV RI > 0.29, or Decrease in a-, v- and d- waves, or reversed flow in both a- and v-waves.
Anshul, 2010.[18]	India	2005-2007	100	>= 28	Cohort	LMP and first trimester dating scan	UA	S/D ratio >= 3 or AREDF.
Bano, 2010.[11]	India	Not stated	90	30 - 41	Cohort	Not specified	UA, MCA, CPR	MCA < 2SD; UA > 2SD or CPR PI < 1.08
Dhand, 2011.[31]	India	2005-2006	121	28 - 41	Cohort	LMP and fetal biometry <22weeks	MCA	Not specified
Dorman, 2002.[35]	Kenya	1996-1997	854	24 - 31	Cohort	LMP and fetal biometry	UtA	Early diastolic notch or mean/ipsilateral UtA RI >= 0.58
Ebrashy, 2005.[19]	Egypt	2002-2003	80	>= 28	Case-control	Fetal biometry (BPD, AC and FL)	UA, MCA, CPR	UA RI > 0.72, MCA RI < 0.69, CPR RI < 1.0

Geerts, 2007.[20]	South Africa	Not stated	113	24 - 34	Cohort	LMP and fetal biometry	UA, CPR, DV	UA PI >95th centile; UA/MCA >1; DV PI > 95th centile.
Khanduri, 2013.[21]	India	2009-2011	60	23 - 37	Cohort	LMP and first or second trimester ultrasound	UA, MCA	UA PI > 1.42 or UA RI > 0.72, MCA PI <1.5, MCA RI < 0.59
Kumari, 2019.[12]	India	2015-2016	30		Cohort	Not specified	UA, MCA, FDA	MCA PSV > 1.50 MoM, FDA PSV delta > 70.50. Not specified for UA
Lakhkar, 2006.[13]	India	2001-2002	58	> 30	Cohort	LMP, clinical gestational age, 1st or 2nd trimester biometry	UA, MCA, CPR, FDA	S/D ratio, RI or PI of UA > 2SD; MCA < 5th centile; FDA > 2SD; CPR PI or S/D ratio < 1.0
Lakshmi, 2013.[22]	India	2007-2008	238	< 35	Cohort	LMP or first trimester ultrasound	UA	Absent and/or reversed end-diastolic flow (AREDF)
Malik, 2013.[23]	India	2010-2011	100	31 - 41	Cohort	LMP	UA, MCA, CPR, UtA	Not specified
Masihi, 2019.[34]	Iran	2016-2017	181	38 - 40	Cohort	First trimester ultrasound	CPR	CPR PI <1.94
Mullick, 1993.[24]	India	Not stated	73	22 - 26, 30 - 32, > 37	Cohort	Not specified	UA	S/D ratio >= 4 (26 weeks), 3.5 (30-32 weeks) and 3 (37-40 weeks)
Nagar, 2015.[25]	India	2009 - 2011	500	26 - 30	Cohort	LMP and ultrasound before 21 weeks	UA, UtA	UA (S/D ratio or RI) > 95th centile or AREDF. UtA S/D ratio > 95th centile
Najam, 2016.[26]	India	Not stated	150	28 - 40	Cohort	Not specified	UA, MCA, CPR	UA S/D ratio > 2SD, or AREDF, MCA SD ratio < 5th percentile,

For peer review only

								MCA/UA SD ratio of < 1.0
Nouh, 2011.[36]	Egypt	2009-2011	80	8 - 12, 26	Case-control	LMP and first trimester ultrasound	UtA	UtA PI> 95th percentile, and/or Unilateral or bilateral notch
Pares, 2008.[32]	Brasil	1997-2005	46	20 - 34	Cohort	Sonographic exam at <= 20 weeks	MCA, FDA	FDA-MV >= 2SD MCA-PSV >= 1.5 MoM
Pattinson, 1991.[14]	South Africa	1987-1989	53	16 - 28	Cohort	LMP and biometry: 16-20 weeks	UA, UtA	UA RI > 95th centile UtA RI > 0.58
Pattinson, 1993.[27]	South Africa	1990	496	16 - 24	Cohort	Not specified	UA	UA RI > 95th centile
Phupong, 2003.[37]	Thailand	2000-2001	322	22 - 28	Cohort	LMP and first trimester ultrasound	UtA	Unilateral or bilateral early diastolic notch
Rani, 2016.[15]	India	2012-2014	223	30 - 36	Cohort	Not specified	UA, MCA, CPR	UA PI > 1.03, UA RI >0.695; MCA PI < 1.2, MCA RI < 0.75; CPR PI < 1.08 or CPR RI < 1.05.
Rocca, 1995.[16]	Egypt	Not stated	113	>= 28	Cohort	Not specified	UA	UA S/D ratio >= 3
Verma, 2016.[38]	India	Not stated	165	22 - 24	Cohort	Not specified	UtA	Bilateral diastolic notches or mean UtA PI > 1.45 (UtA PI > 95th centile).
Waa, 2010.[28]	Kenya	2007	100	>= 28	Cohort	Not specified	MCA, UA	MCA RI < 0.71, and UA > 0.71.
Yelikar, 2013.[29]	India	Not stated	189	> 32	Cohort	Not specified	UA	UA S/D ratio > 90th centile or AREDF
Zarean, 2018.[39]	Iran	2015-2016	100	30 - 34	Cohort	Not specified	UtA	UtA PI > 95th centile

[a]LMP: last menstrual period; UA: umbilical artery; MCA: middle cerebral artery; CPR: cerebroplacental ratio; UtA: uterine artery; FDA: fetal descending aorta; DV: ductus venosus; RI: resistive index; PI: pulsatility index; S/D ratio: systolic diastolic ratio; PSV: peak systolic velocity; MV: mean velocity; AREDF: absent and/or reversed end diastolic flow.

For peer review only

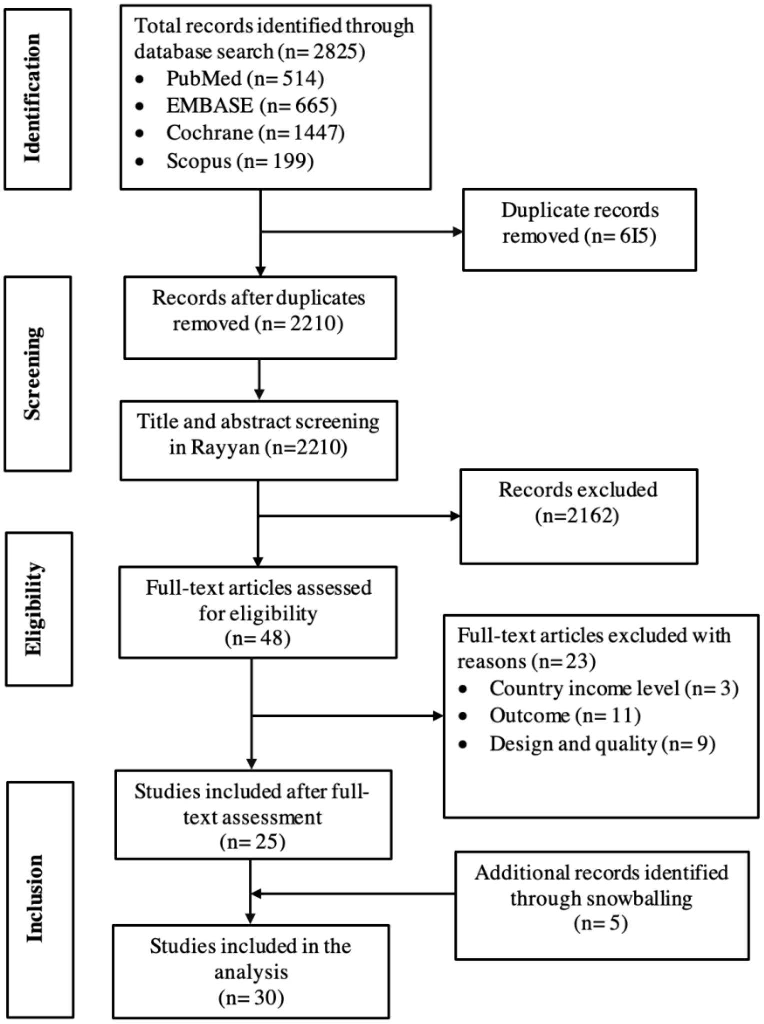

PRISMA flow diagram

273x283mm (144 x 144 DPI)

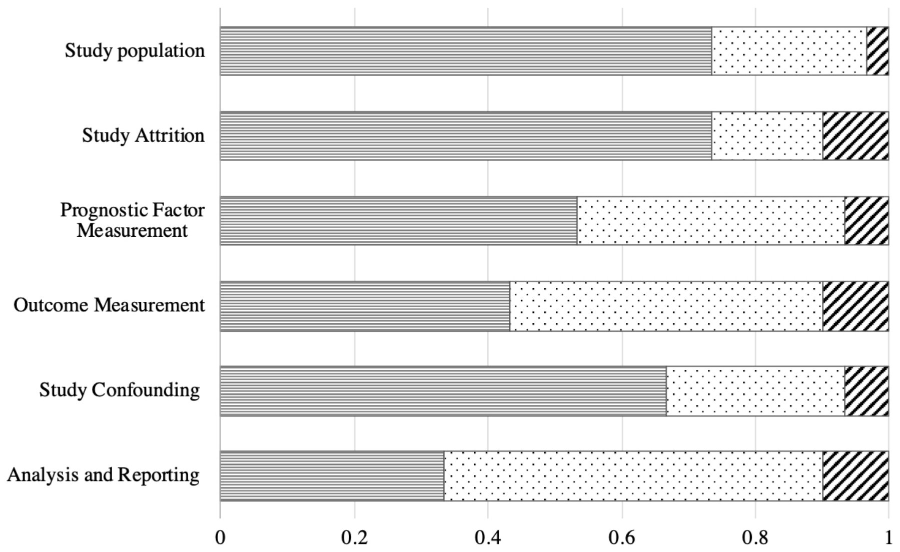

Risk of bias assessment results of the 30 included studies

482x350mm (72 x 72 DPI)

Appendix S1. Search strings for the databases used to retrieve articles

EMBASE

(‘developing countr*’:ti,ab,kw OR ‘developing nation*’:ti,ab,kw OR ‘developing population*’:ti,ab,kw OR ‘developing econom*’:ti,ab,kw OR ‘undeveloped countr*’:ti,ab,kw OR ‘undeveloped nation*’:ti,ab,kw OR ‘undeveloped economy’:ti,ab,kw OR ‘undeveloped economies’:ti,ab,kw OR ‘least developed countr*’:ti,ab,kw OR ‘least developed nation*’:ti,ab,kw OR ‘least developed economy’:ti,ab,kw OR ‘least developed economies’:ti,ab,kw OR ‘less-developed countr*’:ti,ab,kw OR ‘less-developed nation*’:ti,ab,kw OR ‘less-developed population’:ti,ab,kw OR ‘less-developed populations’:ti,ab,kw OR ‘less-developed econom*’:ti,ab,kw OR ‘lesser developed countr*’:ti,ab,kw OR ‘lesser developed nation*’:ti,ab,kw OR ‘lesser developed population’:ti,ab,kw OR ‘lesser developed populations’:ti,ab,kw OR ‘lesser developed economy’:ti,ab,kw OR ‘lesser developed economies’:ti,ab,kw OR ‘under-developed countr*’:ti,ab,kw OR ‘under-developed nation*’:ti,ab,kw OR ‘underdeveloped countr*’:ti,ab,kw OR ‘underdeveloped nation*’:ti,ab,kw OR ‘underdeveloped population*’:ti,ab,kw OR ‘underdeveloped econom*’:ti,ab,kw OR ‘low income countr*’:ti,ab,kw OR ‘middle income countr*’:ti,ab,kw OR ‘low income nation*’:ti,ab,kw OR ‘middle income nation*’:ti,ab,kw OR ‘low income population*’:ti,ab,kw OR ‘middle income population*’:ti,ab,kw OR ‘low income econom*’:ti,ab,kw OR ‘middle income econom*’:ti,ab,kw OR ‘lower income countr*’:ti,ab,kw OR ‘lower income nation*’:ti,ab,kw OR ‘lower income population*’:ti,ab,kw OR ‘lower income economy’:ti,ab,kw OR ‘lower income economies’:ti,ab,kw OR ‘resource limited’:ti,ab,kw OR ‘low resource countr*’:ti,ab,kw OR ‘lower resource countr*’:ti,ab,kw OR ‘low resource nation*’:ti,ab,kw OR ‘low resource population*’:ti,ab,kw OR ‘low resource economy’:ti,ab,kw OR ‘low resource economies’:ti,ab,kw OR ‘underserved countr*’:ti,ab,kw OR ‘underserved nation*’:ti,ab,kw OR ‘underserved population*’:ti,ab,kw OR ‘underserved economy’:ti,ab,kw OR ‘underserved economies’:ti,ab,kw OR ‘under-served country’:ti,ab,kw OR ‘under-served countries’:ti,ab,kw OR ‘under-served nation’:ti,ab,kw OR ‘under-served nations’:ti,ab,kw OR ‘under-served population’:ti,ab,kw OR ‘under-served populations’:ti,ab,kw OR ‘underserved economy’:ti,ab,kw OR ‘underserved economies’:ti,ab,kw OR ‘derived countr*’:ti,ab,kw OR ‘deprived nation’:ti,ab,kw OR ‘deprived nations’:ti,ab,kw OR ‘derived population*’:ti,ab,kw OR ‘deprived economy’:ti,ab,kw OR ‘deprived economies’:ti,ab,kw OR ‘poor countr*’:ti,ab,kw OR ‘poor nation*’:ti,ab,kw OR ‘poor population*’:ti,ab,kw OR ‘poor econom*’:ti,ab,kw OR ‘poorer countr*’:ti,ab,kw OR ‘poorer nation*’:ti,ab,kw OR ‘poorer population*’:ti,ab,kw OR ‘poorer econom*’:ti,ab,kw OR ‘lmic’:ti,ab,kw OR ‘lmics’:ti,ab,kw OR ‘lami’:ti,ab,kw OR ‘transitional countr*’:ti,ab,kw OR ‘transitional nation’:ti,ab,kw OR ‘transitional nations’:ti,ab,kw OR ‘transitional econom*’:ti,ab,kw OR ‘transition countr*’:ti,ab,kw OR ‘transition nation*’:ti,ab,kw OR ‘transition econom*’:ti,ab,kw OR low ‘resource setting*’:ti,ab,kw OR ‘lower resource setting*’:ti,ab,kw OR ‘middle resource setting*’:ti,ab,kw OR ‘Third World*’:ti,ab,kw OR ‘south east asia*’:ti,ab,kw OR ‘middle east*’:ti,ab,kw OR ‘Afghan*’:ti,ab,kw OR ‘Angola*’:ti,ab,kw OR ‘Angolese*’:ti,ab,kw OR ‘Angolian*’:ti,ab,kw OR ‘Armenia*’:ti,ab,kw OR ‘Bangladesh*’:ti,ab,kw OR ‘Benin*’:ti,ab,kw OR ‘Bhutan*’:ti,ab,kw OR ‘Birma*’:ti,ab,kw OR ‘Burma*’:ti,ab,kw OR ‘Birmese*’:ti,ab,kw OR ‘Burmese*’:ti,ab,kw OR ‘Boliv*’:ti,ab,kw OR ‘Botswan*’:ti,ab,kw OR ‘burkina Faso*’:ti,ab,kw OR ‘Burundi*’:ti,ab,kw OR ‘Cabo Verde*’:ti,ab,kw OR ‘Cambod*’:ti,ab,kw OR ‘Cameroon*’:ti,ab,kw OR ‘Cape Verd*’:ti,ab,kw OR ‘Central Africa*’:ti,ab,kw OR ‘Chad’:ti,ab,kw OR ‘Comoro*’:ti,ab,kw OR ‘Congo*’:ti,ab,kw OR ‘Cote d/Ivoire*’:ti,ab,kw OR ‘Djibouti*’:ti,ab,kw OR ‘East Africa*’:ti,ab,kw OR ‘Eastern Africa*’:ti,ab,kw OR ‘Egypt*’:ti,ab,kw OR ‘El Salvador*’:ti,ab,kw OR ‘Equatorial Guinea*’:ti,ab,kw OR ‘Eritre*’:ti,ab,kw OR ‘Ethiopia*’:ti,ab,kw OR ‘Gabon*’:ti,ab,kw OR ‘Gambia*’:ti,ab,kw OR ‘Gaza*’:ti,ab,kw OR ‘Georgia Republic’/exp OR

For peer review only

‘Ghan*’:ti,ab,kw OR ‘Guatemal*’:ti,ab,kw OR ‘Guinea’:ti,ab,kw OR ‘Haiti*’:ti,ab,kw OR ‘Hondur*’:ti,ab,kw OR ‘India*’:ti,ab,kw OR ‘Indones*’:ti,ab,kw OR ‘Ivory Coast*’:ti,ab,kw OR ‘Kenya*’:ti,ab,kw OR ‘Kiribati*’:ti,ab,kw OR ‘Kosovo*’:ti,ab,kw OR ‘Kyrgyz*’:ti,ab,kw OR ‘Lao PDR*’:ti,ab,kw OR ‘Laos*’:ti,ab,kw OR ‘Lesotho*’:ti,ab,kw OR ‘Liberia*’:ti,ab,kw OR ‘Madagascar*’:ti,ab,kw OR ‘Malaw*’:ti,ab,kw OR ‘Mali’:ti,ab,kw OR ‘Mauritan*’:ti,ab,kw OR ‘Mauriti*’:ti,ab,kw OR ‘Micronesi*’:ti,ab,kw OR ‘Mocambiqu*’:ti,ab,kw OR ‘Moldov*’:ti,ab,kw OR ‘Mongolia*’:ti,ab,kw OR ‘Morocc*’:ti,ab,kw OR ‘Mozambiqu*’:ti,ab,kw OR ‘Myanmar*’:ti,ab,kw OR ‘Namibia*’:ti,ab,kw OR ‘Nepal*’:ti,ab,kw OR ‘Nicaragua*’:ti,ab,kw OR ‘Niger*’:ti,ab,kw OR ‘North Korea*’:ti,ab,kw OR ‘Northern Korea*’:ti,ab,kw OR ‘Democratic People/s Republic of Korea’:ti,ab,kw OR ‘Pakistan*’:ti,ab,kw OR ‘Papua New Guinea*’:ti,ab,kw OR ‘Philippine*’:ti,ab,kw OR ‘Principe’:ti,ab,kw OR ‘Rhodesia*’:ti,ab,kw OR ‘Rwanda*’:ti,ab,kw OR ‘Samoa*’:ti,ab,kw OR ‘Sao Tome*’:ti,ab,kw OR ‘Senegal*’:ti,ab,kw OR ‘Sierra Leone*’:ti,ab,kw OR ‘Solomon Islands*’:ti,ab,kw OR ‘Somalia*’:ti,ab,kw OR ‘South Africa*’:ti,ab,kw OR ‘South Sudan*’:ti,ab,kw OR ‘Southern Africa*’:ti,ab,kw OR ‘Sri Lanka*’:ti,ab,kw OR ‘Sub Saharan Africa*’:ti,ab,kw OR ‘Subsaharan Africa*’:ti,ab,kw OR ‘Sudan*’:ti,ab,kw OR ‘Swaziland*’:ti,ab,kw OR ‘Syria*’:ti,ab,kw OR ‘Tajikist*’:ti,ab,kw OR ‘Tanzan*’:ti,ab,kw OR ‘Timor*’:ti,ab,kw OR ‘Togo*’:ti,ab,kw OR ‘Tonga*’:ti,ab,kw OR ‘Tunis*’:ti,ab,kw OR ‘Ugand*’:ti,ab,kw OR ‘Ukrain*’:ti,ab,kw OR ‘Uzbekistan*’:ti,ab,kw OR ‘Vanuatu*’:ti,ab,kw OR ‘Vietnam*’:ti,ab,kw OR ‘West Africa*’:ti,ab,kw OR ‘West Bank*’:ti,ab,kw OR ‘Western Africa*’:ti,ab,kw OR ‘Yemen*’:ti,ab,kw OR ‘Zaire*’:ti,ab,kw OR ‘Zambia*’:ti,ab,kw OR ‘Zimbabw*’:ti,ab,kw)

AND

(‘Umbilical Arter*’/exp OR ‘Uterine Artery’/exp OR ‘Middle Cerebral Artery’/exp OR ‘Ductus Venosus’/exp OR ‘Umbilical Vein*’/exp OR ‘Inferior Cava Vein’/exp OR ‘Umbilical Arter*’:ti,ab,kw OR ‘Uterine Arter*’:ti,ab,kw OR ‘Middle Cerebral Arter*’:ti,ab,kw OR ‘Patent Ductus Venosus’:ti,ab,kw OR ‘Umbilical Vein*’:ti,ab,kw OR ‘Inferior Vena Cava’:ti,ab,kw OR ‘Cerebroplacental Ratio’:ti,ab,kw OR ‘CPR’:ti,ab,kw OR ‘Fetal Descending Aorta’:ti,ab,kw OR ‘FDA’:ti,ab,kw OR ‘Doppler Ultrasonography’/exp OR ‘Doppler Ultrasound*’:ti,ab,kw OR ‘Doppler Ultrasonography’:ti,ab,kw OR ‘Uterine Artery Doppler’:ti,ab,kw)

AND

(‘Stillbirth’:ti,ab,kw OR ‘Perinatal Death’:ti,ab,kw OR ‘Cesarean Section*’:ti,ab,kw OR ‘Caesarean Section*’:ti,ab,kw OR ‘Acidosis’:ti,ab,kw OR ‘Premature Birth’:ti,ab,kw OR ‘Neonatal Intensive Care’:ti,ab,kw OR ‘Fetal Growth Retard*’:ti,ab,kw OR ‘Newborn Respiratory Distress Syndrome*’:ti,ab,kw OR ‘Gestational Age’:ti,ab,kw OR ‘Birth Weight’:ti,ab,kw OR ‘Asphyxia Neonatorum’:ti,ab,kw OR ‘Apgar Score*’:ti,ab,kw OR ‘Length of Stay’:ti,ab,kw OR ‘Stillbirth’/exp OR ‘Perinatal Death’/exp OR ‘Perinatal Mortality’/exp OR ‘Cesarean Section’/exp OR ‘Acidosis’/exp OR ‘Prematurity’/exp OR ‘Newborn Intensive Care’/exp OR ‘Intrauterine Growth Retardation’/exp OR ‘Neonatal Respiratory Distress Syndrome’/exp OR ‘Gestational Age’/exp OR ‘Birth Weight’/exp OR ‘Newborn Hypoxia’/exp OR ‘Apgar Score’/exp OR ‘Length of Stay’/exp OR ‘Pregnancy’:ti,ab,kw OR ‘Pregnancies’:ti,ab,kw OR ‘Gestation’:ti,ab,kw OR ‘Pregnant’:ti,ab,kw OR ‘Pregnancy’/exp)

For peer review only

PUBMED (MEDLINE)

("Developing Countries"[Mesh] OR developing countr*[tiab] OR developing nation*[tiab] OR developing population*[tiab] OR developing econom*[tiab] OR undeveloped countr*[tiab] OR undeveloped nation*[tiab] OR "undeveloped economy"[tiab] OR "undeveloped economies"[tiab] OR least developed countr*[tiab] OR least developed nation*[tiab] OR "least developed economy"[tiab] OR "least developed economies"[tiab] OR less-developed countr*[tiab] OR less-developed nation*[tiab] OR "less-developed population"[tiab] OR "less-developed populations"[tiab] OR less-developed econom*[tiab] OR lesser developed countr*[tiab] OR lesser developed nation*[tiab] OR "lesser developed population"[tiab] OR "lesser developed populations"[tiab] OR "lesser developed economy"[tiab] OR "lesser developed economies"[tiab] OR under-developed countr*[tiab] OR under-developed nation*[tiab] OR underdeveloped countr*[tiab] OR underdeveloped nation*[tiab] OR underdeveloped population*[tiab] OR underdeveloped econom*[tiab] OR low income countr*[tiab] OR middle income countr*[tiab] OR low income nation*[tiab] OR middle income nation*[tiab] OR low income population*[tiab] OR middle income population*[tiab] OR low income econom*[tiab] OR middle income econom*[tiab] OR lower income countr*[tiab] OR lower income nation*[tiab] OR lower income population*[tiab] OR "lower income economy"[tiab] OR "lower income economies"[tiab] OR resource limited[tiab] OR low resource countr*[tiab] OR lower resource countr*[tiab] OR low resource nation*[tiab] OR low resource population*[tiab] OR "low resource economy"[tiab] OR "low resource economies"[tiab] OR underserved countr*[tiab] OR underserved nation*[tiab] OR underserved population*[tiab] OR "underserved economy"[tiab] OR "underserved economies"[tiab] OR "under-served country"[tiab] OR "under-served countries"[tiab] OR "under-served nation"[tiab] OR "under-served nations"[tiab] OR "under-served population"[tiab] OR "under-served populations"[tiab] OR "underserved economy"[tiab] OR "underserved economies"[tiab] OR derived countr*[tiab] OR "deprived nation"[tiab] OR "deprived nations"[tiab] OR derived population*[tiab] OR "deprived economy"[tiab] OR "deprived economies"[tiab] OR poor countr*[tiab] OR poor nation*[tiab] OR poor population*[tiab] OR poor econom*[tiab] OR poorer countr*[tiab] OR poorer nation*[tiab] OR poorer population*[tiab] OR poorer econom*[tiab] OR lmic[tiab] OR lmics[tiab] OR lami[tiab] OR transitional countr*[tiab] OR "transitional nation"[tiab] OR "transitional nations"[tiab] OR transitional econom*[tiab] OR transition countr*[tiab] OR transition nation*[tiab] OR transition econom*[tiab] OR low resource setting*[tiab] OR lower resource setting*[tiab] OR middle resource setting*[tiab] OR Third World*[tiab] OR south east asia*[tw] OR middle east*[tw] OR Afghan*[tw] OR Angola*[tw] OR Angolese*[tw] OR Angolian*[tw] OR Armenia*[tw] OR Bangladesh*[tw] OR Benin*[tw] OR Bhutan*[tw] OR Birma*[tw] OR Burma*[tw] OR Birmese*[tw] OR Burmese*[tw] OR Boliv*[tw] OR Botswan*[tw] OR burkina Faso*[tw] OR Burundi*[tw] OR Cabo Verde*[tw] OR Cambod*[tw] OR Cameroon*[tw] OR Cape Verd*[tw] OR Central Africa*[tw] OR Chad[tw] OR Comoro*[tw] OR Congo*[tw] OR Cote d'Ivoire*[tw] OR Djibouti*[tw] OR East Africa*[tw] OR Eastern Africa*[tw] OR Egypt*[tw] OR El Salvador*[tw] OR Equatorial Guinea*[tw] OR Eritre*[tw] OR Ethiopia*[tw] OR Gabon*[tw] OR Gambia*[tw] OR Gaza*[tw] OR "Georgia Republic"[Mesh] OR Ghan*[tw] OR Guatemal*[tw] OR Guinea[tw] OR Haiti*[tw] OR Hondur*[tw] OR India*[tw] OR Indones*[tw] OR Ivory Coast*[tw] OR Kenya*[tw] OR Kiribati*[tw] OR Kosovo*[tw] OR Kyrgyz*[tw] OR Lao PDR*[tw] OR Laos*[tw] OR Lesotho*[tw] OR Liberia*[tw] OR Madagascar*[tw] OR Malaw*[tw] OR Mali[tw] OR Mauritan*[tw] OR Mauriti*[tw] OR Micronesi*[tw] OR Mocambiqu*[tw] OR Moldov*[tw] OR Mongolia*[tw] OR Morocc*[tw] OR Mozambiqu*[tw] OR Myanmar*[tw] OR Namibia*[tw] OR Nepal*[tw] OR Nicaragua*[tw] OR Niger*[tw] OR North Korea*[tw] OR Northern Korea*[tw] OR "Democratic People s Republic of Korea"[tiab] OR "Democratic People's Republic of Korea"[Mesh] OR Pakistan*[tw] OR Papua New Guinea*[tw] OR Philippine*[tw] OR Principe[tw] OR Rhodesia*[tw] OR Rwanda*[tw] OR Samoa*[tw] OR Sao Tome*[tw] OR Senegal*[tw] OR Sierra Leone*[tw] OR Solomon Islands*[tw]

OR Somalia*[tw] OR South Africa*[tw] OR South Sudan*[tw] OR Southern Africa*[tw] OR Sri Lanka*[tw] OR Sub Saharan Africa*[tw] OR Subsaharan Africa*[tw] OR Sudan*[tw] OR Swaziland*[tw] OR Syria*[tw] OR Tajikist*[tw] OR Tanzan*[tw] OR Timor*[tw] OR Togo*[tw] OR Tonga*[tw] OR Tunis*[tw] OR Ugand*[tw] OR Ukrain*[tw] OR Uzbekistan*[tw] OR Vanuatu*[tw] OR Vietnam*[tw] OR West Africa*[tw] OR West Bank*[tw] OR Western Africa*[tw] OR Yemen*[tw] OR Zaire*[tw] OR Zambia*[tw] OR Zimbabw*[tw])

AND

("Umbilical Arteries"[Mesh] OR "Uterine Artery"[Mesh] OR "Middle Cerebral Artery"[Mesh] OR "Ductus Venosus" [Supplementary Concept] OR "Umbilical Veins"[Mesh] OR "Vena Cava, Inferior"[Mesh] OR Umbilical Arter*[tiab] OR Uterine Arter*[tiab] OR Middle Cerebral Arter*[tiab] OR Patent Ductus Venosus[tiab] OR Umbilical Vein*[tiab] OR Inferior Vena Cava[tiab] OR Cerebroplacental Ratio[tiab] OR CPR[tiab] OR Fetal Descending Aorta[tiab] OR FDA[tiab] OR "Ultrasonography, Doppler"[Mesh] OR Doppler Ultrasound*[Title/Abstract] OR Doppler Ultrasonography[Title/Abstract] OR Uterine Artery Doppler[Title/Abstract])

AND

("Stillbirth"[tiab] OR "Perinatal Death"[tiab] OR "Cesarean Section*"[tiab] OR "Caesarean Section*"[tiab] OR Acidosis[tiab] OR Premature Birth[tiab] OR Neonatal Intensive Care"[tiab] OR Fetal Growth Retard*[tiab] OR Newborn Respiratory Distress Syndrome*[tiab] OR Gestational Age[tiab] OR Birth Weight[tiab] OR Asphyxia Neonatorum[tiab] OR Apgar Score*[tiab] OR Length of Stay"[tiab] OR "Stillbirth"[Mesh] OR "Perinatal Death"[Mesh] OR "Cesarean Section"[Mesh] OR "Acidosis"[Mesh] OR "Premature Birth"[Mesh] OR "Intensive Care, Neonatal"[Mesh] OR "Fetal Growth Retardation"[Mesh] OR "Respiratory Distress Syndrome, Newborn"[Mesh] OR "Gestational Age"[Mesh] OR "Birth Weight"[Mesh] OR "Asphyxia Neonatorum"[Mesh] OR "Apgar Score"[Mesh] OR "Length of Stay"[Mesh] OR Pregnancy[Title/Abstract] OR Pregnancies[Title/Abstract] OR Gestation[Title/Abstract] OR Pregnant[Title/Abstract] OR "Pregnancy"[Mesh])

COCHRANE

'developing countr*' OR 'developing nation*' OR 'developing population*' OR 'developing econom*' OR 'undeveloped countr*' OR 'undeveloped nation*' OR 'undeveloped economy' OR 'undeveloped economies' OR 'least developed countr*' OR 'least developed nation*' OR 'least developed economy' OR 'least developed economies' OR 'less-developed countr*' OR 'less-developed nation*' OR 'less-developed population' OR 'less-developed populations' OR 'less-developed econom*' OR 'lesser developed countr*' OR 'lesser developed nation*' OR 'lesser developed population' OR 'lesser developed populations' OR 'lesser developed economy' OR 'lesser developed economies' OR 'under-developed countr*' OR 'under-developed nation*' OR 'underdeveloped countr*'OR 'underdeveloped nation*' OR 'underdeveloped population*' OR 'underdeveloped econom*' OR 'low income countr*' OR 'middle income countr*' OR 'low income nation*' OR 'middle income nation*' OR 'low income population*' OR 'middle income population*' OR 'low income econom*' OR 'middle income econom*' OR 'lower income countr*' OR 'lower income nation*' OR 'lower income population*' OR 'lower income economy' OR 'lower income economies' OR 'resource limited' OR 'low resource countr*' OR 'lower resource countr*' OR 'low resource nation*' OR 'low resource population*' OR 'low resource economy' OR 'low resource economies' OR 'underserved countr*' OR 'underserved nation*' OR 'underserved

For peer review only

population*' OR 'underserved economy' OR 'underserved economies' OR 'under-served country' OR 'under-served countries' OR 'under-served nation' OR 'under-served nations' OR 'under-served population' OR 'under-served populations' OR 'underserved economy' OR 'underserved economies' OR 'derived countr*' OR 'deprived nation' OR 'deprived nations' OR 'derived population*' OR 'deprived economy' OR 'deprived economies' OR 'poor countr*' OR 'poor nation*' OR 'poor population*' OR 'poor econom*' OR 'poorer countr*' OR 'poorer nation*' OR 'poorer population*' OR 'poorer econom*' OR 'lmic' OR 'lmics' OR 'lami' OR 'transitional countr*' OR 'transitional nation' OR 'transitional nations' OR 'transitional econom*' OR 'transition countr*' OR 'transition nation*' OR 'transition econom*' OR low 'resource setting*' OR 'lower resource setting*' OR 'middle resource setting*' OR 'Third World*' OR 'south east asia*' OR 'middle east*' OR 'Afghan*' OR 'Angola*' OR 'Angolese*' OR 'Angolian*' OR 'Armenia*' OR 'Bangladesh*' OR 'Benin*' OR 'Bhutan*' OR 'Birma*' OR 'Burma*' OR 'Birmese*' OR 'Burmese*' OR 'Boliv*' OR 'Botswan*' OR 'burkina Faso*' OR 'Burundi*' OR 'Cabo Verde*' OR 'Cambod*' OR 'Cameroon*' OR 'Cape Verd*' OR 'Central Africa*' OR 'Chad' OR 'Comoro*' OR 'Congo*' OR 'Cote d'Ivoire*' OR 'Djibouti*' OR 'East Africa*' OR 'Eastern Africa*' OR 'Egypt*' OR 'El Salvador*' OR 'Equatorial Guinea*' OR 'Eritre*' OR 'Ethiopia*' OR 'Gabon*' OR 'Gambia*' OR 'Gaza*' OR 'Georgia Republic' OR 'Ghan*' OR 'Guatemal*' OR 'Guinea' OR 'Haiti*' OR 'Hondur*' OR 'India*' OR 'Indones*' OR 'Ivory Coast*' OR 'Kenya*' OR 'Kiribati*' OR 'Kosovo*' OR 'Kyrgyz*' OR 'Lao PDR*' OR 'Laos*' OR 'Lesotho*' OR 'Liberia*' OR 'Madagascar*' OR 'Malaw*' OR 'Mali' OR 'Mauritan*' OR 'Mauriti*' OR 'Micronesi*' OR 'Mocambiqu*' OR 'Moldov*' OR 'Mongolia*' OR 'Morocc*' OR 'Mozambiqu*' OR 'Myanmar*' OR 'Namibia*' OR 'Nepal*' OR 'Nicaragua*' OR 'Niger*' OR 'North Korea*' OR 'Northern Korea*' OR 'Democratic People's Republic of Korea' OR 'Pakistan*' OR 'Papua New Guinea*' OR 'Philippine*' OR 'Principe' OR 'Rhodesia*' OR 'Rwanda*' OR 'Samoa*' OR 'Sao Tome*' OR 'Senegal*' OR 'Sierra Leone*' OR 'Solomon Islands*' OR 'Somalia*' OR 'South Africa*' OR 'South Sudan*' OR 'Southern Africa*' OR 'Sri Lanka*' OR 'Sub Saharan Africa*' OR 'Subsaharan Africa*' OR 'Sudan*' OR 'Swaziland*' OR 'Syria*' OR 'Tajikist*' OR 'Tanzan*' OR 'Timor*' OR 'Togo*' OR 'Tonga*' OR 'Tunis*' OR 'Ugand*' OR 'Ukrain*' OR 'Uzbekistan*' OR 'Vanuatu*' OR 'Vietnam*' OR 'West Africa*' OR 'West Bank*' OR 'Western Africa*' OR 'Yemen*' OR 'Zaire*' OR 'Zambia*' OR 'Zimbabw*'

AND
'Umbilical Arter*' OR 'Uterine Artery' OR 'Middle Cerebral Artery' OR 'Ductus Venosus' OR 'Umbilical Vein*' OR 'Inferior Cava Vein' OR 'Uterine Arter*' OR 'Middle Cerebral Arter*' OR 'Patent Ductus Venosus' OR 'Inferior Vena Cava' OR 'Cerebroplacental Ratio' OR 'CPR' OR 'Fetal Descending Aorta' OR 'FDA' OR 'Doppler Ultrasonography' OR 'Doppler Ultrasound*' OR 'Doppler Ultrasonography' OR 'Uterine Artery Doppler'

AND

'Stillbirth' OR 'Perinatal Death' OR 'Cesarean Section*' OR 'Caesarean Section*' OR 'Acidosis' OR 'Premature Birth' OR 'Neonatal Intensive Care' OR 'Fetal Growth Retard*' OR 'Newborn Respiratory Distress Syndrome*' OR 'Gestational Age' OR 'Birth Weight' OR 'Asphyxia Neonatorum' OR 'Apgar Score*' OR 'Perinatal Mortality' OR 'Cesarean Section' OR 'Prematurity' OR 'Newborn Intensive Care' OR 'Intrauterine Growth Retardation' OR 'Neonatal Respiratory Distress Syndrome' OR 'Gestational Age' OR 'Birth Weight' OR 'Newborn Hypoxia' OR 'Length of Stay' OR 'Pregnancy' OR 'Pregnancies' OR 'Gestation' OR 'Pregnant'

For peer review only

SCOPUS

TITLE-ABS-KEY(“developing countr*” OR “developing nation*” OR “developing population*” OR “developing econom*” OR “undeveloped countr*” OR “undeveloped nation*” OR “undeveloped economy” OR “undeveloped economies” OR “least developed countr*” OR “least developed nation*” OR “least developed economy” OR “least developed economies” OR “less-developed countr*” OR “less-developed nation*” OR “less-developed population” OR “less-developed populations” OR “less-developed econom*” OR “lesser developed countr*” OR “lesser developed nation*” OR “lesser developed population” OR “lesser developed populations” OR “lesser developed economy” OR “lesser developed economies” OR “under-developed countr*” OR “under-developed nation*” OR “underdeveloped countr*” OR “underdeveloped nation*” OR “underdeveloped population*” OR “underdeveloped econom*” OR “low income countr*” OR “middle income countr*” OR “low income nation*” OR “middle income nation*” OR “low income population*” OR “middle income population*” OR “low income econom*” OR “middle income econom*” OR “lower income countr*” OR “lower income nation*” OR “lower income population*” OR “lower income economy” OR “lower income economies” OR “resource limited” OR “low resource countr*” OR “lower resource countr*” OR “low resource nation*” OR “low resource population*” OR “low resource economy” OR “low resource economies” OR “underserved countr*” OR “underserved nation*” OR “underserved population*” OR “underserved economy” OR “underserved economies” OR “under-served country” OR “under-served countries” OR “under-served nation” OR “under-served nations” OR “under-served population” OR “under-served populations” OR “underserved economy” OR “underserved economies” OR “derived countr*” OR “deprived nation” OR “deprived nations” OR “derived population*” OR “deprived economy” OR “deprived economies” OR “poor countr*” OR “poor nation*” OR “poor population*” OR “poor econom*” OR “poorer countr*” OR “poorer nation*” OR “poorer population*” OR “poorer econom*” OR “lmic” OR “lmics” OR “lami” OR “transitional countr*” OR “transitional nation” OR “transitional nations” OR “transitional econom*” OR “transition countr*” OR “transition nation*” OR “transition econom*” OR low “resource setting*” OR “lower resource setting*” OR “middle resource setting*” OR “Third World*” OR “south east asia*” OR “middle east*” OR “Afghan*” OR “Angola*” OR “Angolese*” OR “Angolian*” OR “Armenia*” OR “Bangladesh*” OR “Benin*” OR “Bhutan*” OR “Birma*” OR “Burma*” OR “Birmese*” OR “Burmese*” OR “Boliv*” OR “Botswan*” OR “burkina Faso*” OR “Burundi*” OR “Cabo Verde*” OR “Cambod*” OR “Cameroon*” OR “Cape Verd*” OR “Central Africa*” OR “Chad” OR “Comoro*” OR “Congo*” OR “Cote d/Ivoire*” OR “Djibouti*” OR “East Africa*” OR “Eastern Africa*” OR “Egypt*” OR “El Salvador*” OR “Equatorial Guinea*” OR “Eritre*” OR “Ethiopia*” OR “Gabon*” OR “Gambia*” OR “Gaza*” OR “Georgia Republic” OR “Ghan*” OR “Guatemal*” OR “Guinea” OR “Haiti*” OR “Hondur*” OR “India*” OR “Indones*” OR “Ivory Coast*” OR “Kenya*” OR “Kiribati*” OR “Kosovo*” OR “Kyrgyz*” OR “Lao PDR*” OR “Laos*” OR “Lesotho*” OR “Liberia*” OR “Madagascar*” OR “Malaw*” OR “Mali” OR “Mauritan*” OR “Mauriti*” OR “Micronesi*” OR “Mocambiqu*” OR “Moldov*” OR “Mongolia*” OR “Morocc*” OR “Mozambiqu*” OR “Myanmar*” OR “Namibia*” OR “Nepal*” OR “Nicaragua*” OR “Niger*” OR “North Korea*” OR “Northern Korea*” OR “Democratic People/s Republic of Korea” OR “Pakistan*” OR “Papua New Guinea*” OR “Philippine*” OR “Principe” OR “Rhodesia*” OR “Rwanda*” OR “Samoa*” OR “Sao Tome*” OR “Senegal*” OR “Sierra Leone*” OR “Solomon Islands*” OR “Somalia*” OR “South Africa*” OR “South Sudan*” OR “Southern Africa*” OR “Sri Lanka*” OR “Sub Saharan Africa*” OR “Subsaharan Africa*” OR “Sudan*” OR “Swaziland*” OR “Syria*” OR “Tajikist*” OR “Tanzan*” OR “Timor*” OR “Togo*” OR “Tonga*” OR “Tunis*” OR “Ugand*” OR “Ukrain*” OR “Uzbekistan*” OR “Vanuatu*” OR “Vietnam*” OR “West Africa*” OR “West Bank*” OR “Western Africa*” OR “Yemen*” OR “Zaire*” OR “Zambia*” OR “Zimbabw*”)

For peer review only

AND

TITLE-ABS-KEY("Stillbirth" OR "Perinatal Death" OR "Cesarean Section*" OR "Caesarean Section*" OR "Acidosis" OR "Premature Birth" OR "Neonatal Intensive Care" OR "Fetal Growth Retard*" OR "Newborn Respiratory Distress Syndrome*" OR "Gestational Age" OR "Birth Weight" OR "Asphyxia Neonatorum" OR "Apgar Score*" OR "Length of Stay" OR "Stillbirth" OR "Perinatal Death" OR "Cesarean Section" OR "Acidosis" OR "Premature Birth" OR "Intensive Care, Neonatal" OR "Fetal Growth Retardation" OR "Respiratory Distress Syndrome, Newborn" OR "Gestational Age" OR "Birth Weight" OR "Asphyxia Neonatorum" OR "Apgar Score" OR "Length of Stay" OR "Pregnancy" OR "Pregnancies" OR "Gestation" OR "Pregnant" OR "Pregnancy")

AND

TITLE-ABS-KEY("Umbilical Arteries" OR "Uterine Artery" OR "Middle Cerebral Artery" OR "Ductus Venosus" OR "Umbilical Veins" OR "Vena Cava, Inferior" OR "Umbilical Arter*" OR "Uterine Arter*" OR "Middle Cerebral Arter*" OR "Patent Ductus Venosus" OR "Umbilical Vein*" OR "Inferior Vena Cava" OR "Cerebroplacental Ratio" OR "CPR" OR "Fetal Descending Aorta" OR "FDA" OR "Ultrasonography, Doppler" OR "Doppler Ultrasound*"OR" Doppler Ultrasonography" OR "Uterine Artery Doppler")

For peer review only

Appendix S2. List of full-text articles excluded with reasons

a) Country income level: 3 studies

1. El Shourbagy, S., Elsakhawy, M. (2012). Prediction of fetal anemia by middle cerebral artery Doppler. *Middle East Fertility Society Journal, 17(4), 275-282.*
2. Haley, J., Tuffnell, D. J., Johnson, N. (1997). Randomized controlled trial of cardiotocography versus umbilical artery Doppler in the management of small for gestational age fetuses. *British Journal of Obstetrics and Gynaecology, 104(4), 431-435).*
3. Morales-Rosello, J., Dias, T., Khalil, A., Fornes-Ferrer, V., Ciammella, R., Gimenez-Roca, L., Perales-Marin, A., Thilaganathan, B. (2018). Birth-weight differences at term are explained by placental dysfunction and not by maternal ethnicity. *Ultrasound Obstet Gynecol, 52(4), 488-493.*

b) Design and quality: 9 studies

1. Abidoye, I. A., Ayoola, O. O., Idowu, B., Aderibigbe, A. S., Loto, O. M. (2017). Uterine artery Doppler velocimetry in hypertensive disorder of pregnancy in Nigeria. *J Ultrason, 17(71)) 253-258.*
2. Agarwal, R., Tiwari, A., Wadhwa, N., Radhakrishnan, G., Bhatt, S., Batra, P. (2017). Abnormal umbilical artery Doppler velocimetry and placental histopathological correlation in fetal growth restriction. *South African Journal of Obstetrics and Gynaecology, 23(1), 12-16.*
3. Ali, A., Ara, I., Sultana, R., Akram, F., Zaib, M. J. (2014). Comparison of perinatal outcome of growth restricted fetuses with normal and abnormal umbilical artery Doppler waveforms. *Journal of Ayub Medical College, Abottabad: JAMC, 26(3), 344-348.*
4. Kumar, S., Datta, S., Mittal, S., Roy, K. K. (2002). Doppler flow studies in middle cerebral and umbilical arteries in growth retarded and normal pregnancies. *JK Science, 4(0), 185-189*
5. Mufenda, J., Gebhardt, S., van Rooyen, R., Theron, G. (2015). Introducing a Mobile-Connected Umbilical Doppler Device (UmbiFlow) into a Primary Care Maternity Setting: Does This Reduce Unnecessary Referrals to Specialised Care? Results of a Pilot Study in Kraaifontein, South Africa. *PLoS One, 10(11) e0142743.*
6. Nguku, S. W., Wanyoike-Gichuhi, J., Aywak, A. A. (2006). Biophysical profile scores and resistance indices of the umbilical artery as seen in patients with pregnancy induced hypertension. *East African Medical Journal, 83(3), 96-101*
7. Nkosi, S., Makin, J., Hlongwane, T. M. A. G., & Pattinson, R. C. (2019). Screening and managing a low-risk pregnant population using continuous-wave Doppler ultrasound in a low-income population: A cohort analytical study. *SAMJ: South African Medical Journal*, *109*(5), 347-352.
8. Siddiqui, T. S., Asim, A., Ali, S., Tariq, A. (2014). Comparison of perinatal outcome in growth restricted fetuses retaining normal umbilical artery Doppler flow to those with diminished end-diastolic flow. *Journal of Ayub Medical College, Abbottabad: JAMC, 26(2), 221-224.*
9. Kachewar, S. G., Gandage, S. G., Pawar, H. J. (2012). An Indian study of novel non-invasive method of screening for foetal anaemia. *Journal of Clinical and Diagnostic Research, 6(4), 688-691.*

For peer review only

c) Outcomes: 11 studies

1. Adekanmi, A. J., Roberts, A., Akinmoladun, J. A., & Adeyinka, A. O. (2019). Uterine and umbilical artery doppler in women with pre-eclampsia and their pregnancy outcomes. *Nigerian Postgraduate Medical Journal*, *26*(2), 106.
2. El Behery, M. M., Siam, S., Seksaka, M. A., Mansou, S. M. (2013). Uterine artery Doppler and urinary hyperglycosylated HCG as predictors of threatened abortion outcome. *Middle East Fertility Society Journal, 19(1), 42-46*.
3. El-Mashad, A. I., Mohamed, M. A., Elahadi Farag, M. A., Ahmad, M. K., Ismail, Y. (2011). Role of uterine artery Doppler velocimetry indices and plasma adrenomedullin level in women with unexplained recurrent pregnancy loss. *Journal of Obstetrics and Gynaecology Research, 37(1), 51-57*.
4. Geerts, L., Van der Merwe, E., Theron, A., Rademan, K. (2016). Placental insufficiency among high-risk pregnancies with a normal umbilical artery resistance index after 32 weeks. *Int J Gynaecol Obstet, 135(1), 38-42*.
5. Kumar, B. S., Sarmila, K., Prasad, K. S. (2012). Prediction of preeclampsia by midtrimester uterine artery doppler velocimetry in high-risk and low-risk women. *Journal of Obstetrics and Gynecology of India, 62(3), 297-300*.
6. Maged. A. M., Elnassery, N., Fouad, M., Abdelhafiz, A., Al Mostafa, W. (2015). Third-trimester uterine artery Doppler measurement and maternal postpartum outcome among patients with severe pre-eclampsia. *International Journal of Gynecology and Obstetrics, 131(1), 49-53*.
7. Prajapati, S. R., Maitra, N. (2013). Prediction of pre-eclampsia by a combination of history, uterine artery doppler, and mean arterial pressure (A Prospective Study of 200 Cases). *Journal of Obstetrics and Gynecology of India, 63(1), 32-36*.
8. Sebastian, A., Raj, T. S., Yenuberi, H., Job, V., Varuhghese, S., & Regi, A. (2019). Angiogenic factors and uterine artery Doppler in predicting preeclampsia and associated adverse outcomes in a tertiary hospital in south India. *Pregnancy hypertension*, *16*, 26.
9. Shehata, N. A. A., Ali, H. A. A., Hassan, A., Katta, M. A., Ali, A. S. F. (2018). Doppler and biochemical assessment for the prediction of early pregnancy outcome in patients experiencing threatened spontaneous abortion. *Int J Gynaecol Obstet, 143(2), 150-155*.
10. Yusuf, M., Galadanci, H., Ismail, A., Aliyu, L. D., Danbatta, A. H. (2017). Uterine artery doppler velocimetry for the prediction of preeclampsia among high-risk pregnancies in low-resource setting: Our experience at aminu Kano teaching hospital, Kano, Nigeria. *Donald School Journal of Ultrasound in Obstetrics and Gynecology, 11(3), 197-202*
11. Puri, M. S., Deshpande, H., Kohli, S., Sharma, K., Singhania, S. (2013). A study of uterine artery colour doppler at 20-24 weeks gestation as a predictor of pregnancy induced hypertension and intra uterine growth restriction from industrial town in Western India. *Research Journal of Pharmaceutical, Biological and Chemical Sciences, 4(1), 698-705*.

For peer review only

Appendix S3. Pregnancy risk profiles in the selected studies

First Author	Risk Profile	Risk profile details in article
Abdallah et al., 2019	Low risk	Primigravida >=37 weeks admitted in labor to the delivery unit. Women with BMI >30 kg/m2, multiple pregnancy, fetal malpresentation, fetal demise, chorioamnionitis, meconium-stained liquor, associated medical disorder (hypertension, diabetes, autoimmune disease, etc.), perinatal complication (e.g. placental abruption), fetal malformation or abnormal fetal growth were excluded from the study.
Agbaje et al., 2018	High-risk	Sickle cell anemia.
Alanwar et al., 2018	High-risk	Pregnancies complicated with severe pre-eclampsia.
Allam et al., 2013	High-risk	Suspected IUGR, oligohydramnios, preeclampsia, or placental vascular dysfunction documented by abnormal umbilical artery pulsatility index by local reference ranges.
Anshul et al., 2010	High-risk	SGA foetuses, some mothers had hypertensive disorder, anemia, bad obstetric history
Bano et al., 2010	High risk	Clinical suspicion of IUGR
Dhand et al., 2011	High risk	SGA fetuses
Dorman et al., 2002	High-risk	Maternal falciparum malaria infection.
Ebrashy et al., 2005	High-risk	Pre-eclampsia women
Geerts et al., 2007	High-risk	Women with severe pre-eclampsia
Khanduri et al., 2013	High-risk	Clinical suspicion of IUGR
Kumari et al., 2019	High risk	Rhesus isoimmunized complicated pregnancies
Lakhkar et al., 2006	High risk	Preeclampsia and growth-restricted fetuses
Lakshmi et al., 2013	High-risk	IUGR, pregnancy induced hypertension, h/o previous intrauterine death
Malik et al., 2013	High-risk	IUGR; hypertensive disorder; pre-eclampsia
Masihi et al.2019	Low risk	Women that had uncomplicated pregnancies
Mullick et al., 1993	Low and high-risk	Women attending routine antenatal (any risk profile).
Nagar et al., 2015	High risk	History of preeclampsia or eclampsia in previous pregnancy pre-existing medical disorders like: Diabetes, Renal disease, Epilepsy, Autoimmune disease, Thrombophilia, and Hypertension, History of IUGR or still birth, history of abruptio placentae, preeclampsia or pregnancy-induced hypertension current, Nulliparity, Extremes of age (<20 years and >35 years).
Najam et al., 2016	Low and high-risk	Pregnancies undergoing routine antenatal (any risk profile).
Nouh et al., 2011	High-risk	Primigravida with ovulatory polycystic ovary syndrome (PCOS)

For peer review only - http://bmjopen.bmj.com/site/about/guidelines.xhtml

Pares et al., 2008	High-risk	Fetuses at risk for anemia because of maternal alloimmunization to red-cell antigens
Pattinson et al., 1991	High risk	SGA, preeclampsia and pregnancy wastage
Pattinson et al., 1993	Low and high-risk	Pregnancies undergoing routine antenatal (any risk profile).
Phupong et al., 2003	Low-risk	Healthy pregnant women
Rani et al., 2016	Low and high-risk	Women attending routine antenatal (any risk profile).
Rocca et al., 1995	High risk	Pre-eclampsia women
Verma et al., 2016	Low-risk	Women with uncomplicated pregnancies
Waa et al., 2010	Low and high-risk	Women undergoing routine antenatal (any risk profile).
Yelikar et al., 2013	High-risk	Preeclampsia and growth-restricted fetuses
Zarean et al., 2018	Low-risk	Women that had uncomplicated pregnancies

[a]FGR: fetal growth restriction; LBW: low birth weight; NICU: neonatal intensive care unit. High risk: pregnancies with any underlying condition that threatens the health or life of the mother or her foetus.
Any risk profile: unselected pregnancies (pregnancies undergoing routine antenatal). Low risk: Uncomplicated pregnancies or healthy pregnant women

For peer review only

Appendix S4. Risk of bias assessment results of the 30 studies included in the analysis

First Author: Abdallah et al., 2018 **ID:** 68614233

Potential Bias	Items to be considered for assessment of potential opportunity for bias	Yes	Partly	No	Unsure	NA*
Study population /sample selection	Inclusion and exclusion criteria are adequately described [including explicit diagnostic criteria, start/finish date of recruitment]	x				
	Baseline study sample [i.e., individuals entering the study and their key characteristics and sampling frame are adequately described]	x				
	Study sample represents population of interest on key characteristics, sufficient to limit potential bias to results	Low risk of bias				
Study attrition	Response rate (i.e., proportion of study sample completing the study and providing outcome data) is adequate				x	
	Participants lost to follow-up are adequately described for key characteristics				x	
	Statement as to the possible effect on the results from missing data				x	
	Loss to follow-up is not associated with key characteristics	Moderate risk of bias				
Prognostic factor measurement	Clear definition of the prognostic factors measured is provided (e.g imaging modality method, measurement, and timing described).	x				
	Specified instrument and personnel for measurement of predictive factors	x				
	Continuous variables are reported or appropriate (i.e. not data- dependent) cut-off points are used and specified a priori	x				
	Blinding: were estimators of risk factor status and of outcomes blinded?			x		
	The prognostic factor(s) of interest is (are) adequately measured in study participants to sufficiently limit potential bias	Low risk of bias				
Outcome measurement	Is the outcome(s) clearly defined?			x		
	The outcome measure and method used are adequately valid and reliable to limit misclassification bias	Moderate risk of bias				
Study confounding	Do the authors address potential confounders?	x				
	Important potential confounders are appropriately accounted for, limiting potential bias with respect to the prognostic factor of interest.	Low risk of bias				
Analysis and reporting	There is sufficient presentation of data to assess the adequacy of the analysis strategy and there is no selective reporting		x			
	The statistical analysis is appropriate for the study design, limiting potential for the presentation of invalid results	Moderate risk of bias				
NA*: not applicable. Note: The above table was adapted from: Hayden et al., 2013.						
**Overall opinion of study quality= Moderate risk of bias**						

First Author: Agbaje et al., 2018 **ID:** 6377433

Potential Bias	Items to be considered for assessment of potential opportunity for bias	Yes	Partly	No	Unsure	NA*
Study population /sample selection	Inclusion and exclusion criteria are adequately described [including explicit diagnostic criteria, start/finish date of recruitment]	x				
	Baseline study sample [i.e. individuals entering the study and their key characteristics and sampling frame are adequately described]		x			
	Study sample represents population of interest on key characteristics, sufficient to limit potential bias to results	Moderate risk of bias				
Study attrition	Response rate (i.e., proportion of study sample completing the study and providing outcome data) is adequate					x
	Participants lost to follow-up are adequately described for key characteristics					x
	Statement as to the possible effect on the results from missing data					x
	Loss to follow-up is not associated with key characteristics	Low risk of bias				
Prognostic factor measurement	Clear definition of the prognostic factors measured is provided (e.g. imaging modality method, measurement, and timing described).	x				
	Specified instrument and personnel for measurement of predictive factors	x				
	Continuous variables are reported or appropriate (i.e. not data- dependent) cut-off points are used and specified a priori	x				
	Blinding: were estimators of risk factor status and of outcomes blinded?		x			
	The prognostic factor(s) of interest is (are) adequately measured in study participants to sufficiently limit potential bias	Low risk of bias				
Outcome measurement	Is the outcome(s) clearly defined?		x			
	The outcome measure and method used are adequately valid and reliable to limit misclassification bias	Moderate risk of bias				
Study confounding	Do the authors address potential confounders?		x			
	Important potential confounders are appropriately accounted for, limiting potential bias with respect to the prognostic factor of interest.	Moderate risk of bias				
Analysis and reporting	There is sufficient presentation of data to assess the adequacy of the analysis strategy and there is no selective reporting		x			
	The statistical analysis is appropriate for the study design, limiting potential for the presentation of invalid results	Moderate risk of bias				

NA*: not applicable. Note: The above table was adapted from: Hayden et al., 2013.

Overall opinion of study quality= Moderate risk of bias

First Author: Alanwar et al., 2018 **ID:** 6377464

**Potential Bias**	**Items to be considered for assessment of potential opportunity for bias**	**Yes**	**Partly**	**No**	**Unsure**	**NA***
Study population /sample selection	Inclusion and exclusion criteria are adequately described [including explicit diagnostic criteria, start/finish date of recruitment]	x				
	Baseline study sample [i.e. individuals entering the study and their key characteristics and sampling frame are adequately described]	x				
	Study sample represents population of interest on key characteristics, sufficient to limit potential bias to results	Low risk of bias				
Study attrition	Response rate (i.e., proportion of study sample completing the study and providing outcome data) is adequate					x
	Participants lost to follow-up are adequately described for key characteristics					x
	Statement as to the possible effect on the results from missing data					x
	Loss to follow-up is not associated with key characteristics	Low risk of bias				
Prognostic factor measurement	Clear definition of the prognostic factors measured is provided (e.g. imaging modality method, measurement, and timing described).	x				
	Specified instrument and personnel for measurement of predictive factors	x				
	Continuous variables are reported or appropriate (i.e. not data- dependent) cut-off points are used and specified a priori	x				
	Blinding: were estimators of risk factor status and of outcomes blinded?				x	
	The prognostic factor(s) of interest is (are) adequately measured in study participants to sufficiently limit potential bias	Low risk of bias				
Outcome measurement	Is the outcome(s) clearly defined?	x				
	The outcome measure and method used are adequately valid and reliable to limit misclassification bias	Low risk of bias				
Study confounding	Do the authors address potential confounders?	x				
	Important potential confounders are appropriately accounted for, limiting potential bias with respect to the prognostic factor of interest.	Low risk of bias				
Analysis and reporting	There is sufficient presentation of data to assess the adequacy of the analysis strategy and there is no selective reporting	x				
	The statistical analysis is appropriate for the study design, limiting potential for the presentation of invalid results	Low risk of bias				

NA*: not applicable. Note: The above table was adapted from: Hayden et al., 2013.

Overall opinion of study quality= Low risk of bias

First Author: Allam et al., 2013 **ID:** 6377480

Potential Bias	Items to be considered for assessment of potential opportunity for bias	Yes	Partly	No	Unsure	NA*
Study population /sample selection	Inclusion and exclusion criteria are adequately described [including explicit diagnostic criteria, start/finish date of recruitment]		x			
	Baseline study sample [i.e. individuals entering the study and their key characteristics and sampling frame are adequately described]	x				
	Study sample represents population of interest on key characteristics, sufficient to limit potential bias to results	Low risk of bias				
Study attrition	Response rate (i.e., proportion of study sample completing the study and providing outcome data) is adequate	x				
	Participants lost to follow-up are adequately described for key characteristics				x	
	Statement as to the possible effect on the results from missing data					x
	Loss to follow-up is not associated with key characteristics	Low risk of bias				
Prognostic factor measurement	Clear definition of the prognostic factors measured is provided (e.g. imaging modality method, measurement, and timing described).	x				
	Specified instrument and personnel for measurement of predictive factors	x				
	Continuous variables are reported or appropriate (i.e. not data- dependent) cut-off points are used and specified a priori	x				
	Blinding: were estimators of risk factor status and of outcomes blinded?				x	
	The prognostic factor(s) of interest is (are) adequately measured in study participants to sufficiently limit potential bias	Low risk of bias				
Outcome measurement	Is the outcome(s) clearly defined?	x				
	The outcome measure and method used are adequately valid and reliable to limit misclassification bias	Low risk of bias				
Study confounding	Do the authors address potential confounders?		x			
	Important potential confounders are appropriately accounted for, limiting potential bias with respect to the prognostic factor of interest.	Moderate risk of bias				
Analysis and reporting	There is sufficient presentation of data to assess the adequacy of the analysis strategy and there is no selective reporting		x			
	The statistical analysis is appropriate for the study design, limiting potential for the presentation of invalid results	Moderate risk of bias				

NA*: not applicable. Note: The above table was adapted from: Hayden et al., 2013.

Overall opinion of study quality= Low risk of bias

First Author: Anshul et al., 2010 **ID:** 6377837

Potential Bias	Items to be considered for assessment of potential opportunity for bias	Yes	Partly	No	Unsure	NA*
Study population /sample selection	Inclusion and exclusion criteria are adequately described [including explicit diagnostic criteria, start/finish date of recruitment]	x				
	Baseline study sample [i.e. individuals entering the study and their key characteristics and sampling frame are adequately described]	x				
	Study sample represents population of interest on key characteristics, sufficient to limit potential bias to results	Low risk of bias				
Study attrition	Response rate (i.e., proportion of study sample completing the study and providing outcome data) is adequate				x	
	Participants lost to follow-up are adequately described for key characteristics				x	
	Statement as to the possible effect on the results from missing data				x	
	Loss to follow-up is not associated with key characteristics	High risk of bias				
Prognostic factor measurement	Clear definition of the prognostic factors measured is provided (e.g. imaging modality method, measurement, and timing described).		x			
	Specified instrument and personnel for measurement of predictive factors			x		
	Continuous variables are reported or appropriate (i.e. not data- dependent) cut-off points are used and specified a priori	x				
	Blinding: were estimators of risk factor status and of outcomes blinded?					x
	The prognostic factor(s) of interest is (are) adequately measured in study participants to sufficiently limit potential bias	Moderate risk of bias				
Outcome measurement	Is the outcome(s) clearly defined?			x		
	The outcome measure and method used are adequately valid and reliable to limit misclassification bias	High risk of bias				
Study confounding	Do the authors address potential confounders?	x				
	Important potential confounders are appropriately accounted for, limiting potential bias with respect to the prognostic factor of interest.	Low risk of bias				
Analysis and reporting	There is sufficient presentation of data to assess the adequacy of the analysis strategy and there is no selective reporting			x		
	The statistical analysis is appropriate for the study design, limiting potential for the presentation of invalid results	High risk of bias				

NA*: not applicable. Note: The above table was adapted from: Hayden et al., 2013.

Overall opinion of study quality= High risk of bias

For peer review only

First Author: Bano et al., 2010 **ID:** 74903018

Potential Bias	Items to be considered for assessment of potential opportunity for bias	Yes	Partly	No	Unsure	NA*
Study population /sample selection	Inclusion and exclusion criteria are adequately described [including explicit diagnostic criteria, start/finish date of recruitment]		x			
	Baseline study sample [i.e. individuals entering the study and their key characteristics and sampling frame are adequately described]			x		
	Study sample represents population of interest on key characteristics, sufficient to limit potential bias to results	Moderate risk of bias				
Study attrition	Response rate (i.e., proportion of study sample completing the study and providing outcome data) is adequate					x
	Participants lost to follow-up are adequately described for key characteristics					x
	Statement as to the possible effect on the results from missing data					x
	Loss to follow-up is not associated with key characteristics	Low risk of bias				
Prognostic factor measurement	Clear definition of the prognostic factors measured is provided (e.g. imaging modality method, measurement, and timing described).	x				
	Specified instrument and personnel for measurement of predictive factors			x		
	Continuous variables are reported or appropriate (i.e. not data- dependent) cut-off points are used and specified a priori	x				
	Blinding: were estimators of risk factor status and of outcomes blinded?			x		
	The prognostic factor(s) of interest is (are) adequately measured in study participants to sufficiently limit potential bias	Moderate risk of bias				
Outcome measurement	Is the outcome(s) clearly defined?			x		
	The outcome measure and method used are adequately valid and reliable to limit misclassification bias	High risk of bias				
Study confounding	Do the authors address potential confounders?			x		
	Important potential confounders are appropriately accounted for, limiting potential bias with respect to the prognostic factor of interest.	High risk of bias				
Analysis and reporting	There is sufficient presentation of data to assess the adequacy of the analysis strategy and there is no selective reporting		x			
	The statistical analysis is appropriate for the study design, limiting potential for the presentation of invalid results	Moderate risk of bias				
NA*: not applicable. Note: The above table was adapted from: Hayden et al., 2013.						
**Overall opinion of study quality= high risk of bias**						

First Author: Dhand et al., 2011 **ID:** 6379383

Potential Bias	Items to be considered for assessment of potential opportunity for bias	Yes	Partly	No	Unsure	NA*
Study population /sample selection	Inclusion and exclusion criteria are adequately described [including explicit diagnostic criteria, start/finish date of recruitment]	x				
	Baseline study sample [i.e. individuals entering the study and their key characteristics and sampling frame are adequately described]	x				
	Study sample represents population of interest on key characteristics, sufficient to limit potential bias to results	Low risk of bias				
Study attrition	Response rate (i.e., proportion of study sample completing the study and providing outcome data) is adequate				x	
	Participants lost to follow-up are adequately described for key characteristics				x	
	Statement as to the possible effect on the results from missing data				x	
	Loss to follow-up is not associated with key characteristics	Moderate risk of bias				
Prognostic factor measurement	Clear definition of the prognostic factors measured is provided (e.g. imaging modality method, measurement, and timing described).		x			
	Specified instrument and personnel for measurement of predictive factors		x			
	Continuous variables are reported or appropriate (i.e. not data- dependent) cut-off points are used and specified a priori		x			
	Blinding: were estimators of risk factor status and of outcomes blinded?				x	
	The prognostic factor(s) of interest is (are) adequately measured in study participants to sufficiently limit potential bias	High risk of bias				
Outcome measurement	Is the outcome(s) clearly defined?			x		
	The outcome measure and method used are adequately valid and reliable to limit misclassification bias	High risk of bias				
Study confounding	Do the authors address potential confounders?	x				
	Important potential confounders are appropriately accounted for, limiting potential bias with respect to the prognostic factor of interest.	Low risk of bias				
Analysis and reporting	There is sufficient presentation of data to assess the adequacy of the analysis strategy and there is no selective reporting			x		
	The statistical analysis is appropriate for the study design, limiting potential for the presentation of invalid results	High risk of bias				

NA*: not applicable. Note: The above table was adapted from: Hayden et al., 2013.

Overall opinion of study quality= High risk of bias

First Author: Dorman et al., 2002 **ID:** 6377862

**Potential Bias**	**Items to be considered for assessment of potential opportunity for bias**	**Yes**	**Partly**	**No**	**Unsure**	**NA***
Study population /sample selection	Inclusion and exclusion criteria are adequately described [including explicit diagnostic criteria, start/finish date of recruitment]	x				
	Baseline study sample [i.e. individuals entering the study and their key characteristics and sampling frame are adequately described]	x				
	Study sample represents population of interest on key characteristics, sufficient to limit potential bias to results	Low risk of bias				
Study attrition	Response rate (i.e., proportion of study sample completing the study and providing outcome data) is adequate	x				
	Participants lost to follow-up are adequately described for key characteristics	x				
	Statement as to the possible effect on the results from missing data	x				
	Loss to follow-up is not associated with key characteristics	Low risk of bias				
Prognostic factor measurement	Clear definition of the prognostic factors measured is provided (e.g. imaging modality method, measurement, and timing described).	x				
	Specified instrument and personnel for measurement of predictive factors	x				
	Continuous variables are reported or appropriate (i.e. not data- dependent) cut-off points are used and specified a priori	x				
	Blinding: were estimators of risk factor status and of outcomes blinded?	x				
	The prognostic factor(s) of interest is (are) adequately measured in study participants to sufficiently limit potential bias	Low risk of bias				
Outcome measurement	Is the outcome(s) clearly defined?	x				
	The outcome measure and method used are adequately valid and reliable to limit misclassification bias	Low risk of bias				
Study confounding	Do the authors address potential confounders?	x				
	Important potential confounders are appropriately accounted for, limiting potential bias with respect to the prognostic factor of interest.	Low risk of bias				
Analysis and reporting	There is sufficient presentation of data to assess the adequacy of the analysis strategy and there is no selective reporting	x				
	The statistical analysis is appropriate for the study design, limiting potential for the presentation of invalid results	Low risk of bias				
NA*: not applicable. Note: The above table was adapted from: Hayden et al., 2013.						
**Overall opinion of study quality= Low risk of bias**						

First Author: Ebrashy et al., 2005 **ID:** 6377887

**Potential Bias**	**Items to be considered for assessment of potential opportunity for bias**	**Yes**	**Partly**	**No**	**Unsure**	**NA***
Study population /sample selection	Inclusion and exclusion criteria are adequately described [including explicit diagnostic criteria, start/finish date of recruitment]	x				
	Baseline study sample [i.e. individuals entering the study and their key characteristics and sampling frame are adequately described]	x				
	Study sample represents population of interest on key characteristics, sufficient to limit potential bias to results	Low risk of bias				
Study attrition	Response rate (i.e., proportion of study sample completing the study and providing outcome data) is adequate	x				
	Participants lost to follow-up are adequately described for key characteristics	x				
	Statement as to the possible effect on the results from missing data					x
	Loss to follow-up is not associated with key characteristics	Low risk of bias				
Prognostic factor measurement	Clear definition of the prognostic factors measured is provided (e.g. imaging modality method, measurement, and timing described).	x				
	Specified instrument and personnel for measurement of predictive factors	x				
	Continuous variables are reported or appropriate (i.e. not data- dependent) cut-off points are used and specified a priori	x				
	Blinding: were estimators of risk factor status and of outcomes blinded?	x				
	The prognostic factor(s) of interest is (are) adequately measured in study participants to sufficiently limit potential bias	Low risk of bias				
Outcome measurement	Is the outcome(s) clearly defined?	x				
	The outcome measure and method used are adequately valid and reliable to limit misclassification bias	Low risk of bias				
Study confounding	Do the authors address potential confounders?	x				
	Important potential confounders are appropriately accounted for, limiting potential bias with respect to the prognostic factor of interest.	Low risk of bias				
Analysis and reporting	There is sufficient presentation of data to assess the adequacy of the analysis strategy and there is no selective reporting		x			
	The statistical analysis is appropriate for the study design, limiting potential for the presentation of invalid results	Moderate risk of bias				

NA*: not applicable. Note: The above table was adapted from: Hayden et al., 2013.

Overall opinion of study quality= Low risk of bias

First Author: Geerts et al., 2007 **ID:** 6378017

**Potential Bias**	**Items to be considered for assessment of potential opportunity for bias**	**Yes**	**Partly**	**No**	**Unsure**	**NA***
Study population /sample selection	Inclusion and exclusion criteria are adequately described [including explicit diagnostic criteria, start/finish date of recruitment]	x				
	Baseline study sample [i.e. individuals entering the study and their key characteristics and sampling frame are adequately described]	x				
	Study sample represents population of interest on key characteristics, sufficient to limit potential bias to results	Low risk of bias				
Study attrition	Response rate (i.e., proportion of study sample completing the study and providing outcome data) is adequate	x				
	Participants lost to follow-up are adequately described for key characteristics					x
	Statement as to the possible effect on the results from missing data					x
	Loss to follow-up is not associated with key characteristics	Low risk of bias				
Prognostic factor measurement	Clear definition of the prognostic factors measured is provided (e.g. imaging modality method, measurement, and timing described).		x			
	Specified instrument and personnel for measurement of predictive factors	x				
	Continuous variables are reported or appropriate (i.e. not data- dependent) cut-off points are used and specified a priori	x				
	Blinding: were estimators of risk factor status and of outcomes blinded?	x				
	The prognostic factor(s) of interest is (are) adequately measured in study participants to sufficiently limit potential bias	Low risk of bias				
Outcome measurement	Is the outcome(s) clearly defined?		x			
	The outcome measure and method used are adequately valid and reliable to limit misclassification bias	Moderate risk of bias				
Study confounding	Do the authors address potential confounders?		x			
	Important potential confounders are appropriately accounted for, limiting potential bias with respect to the prognostic factor of interest.	Moderate risk of bias				
Analysis and reporting	There is sufficient presentation of data to assess the adequacy of the analysis strategy and there is no selective reporting	x				
	The statistical analysis is appropriate for the study design, limiting potential for the presentation of invalid results	Low risk of bias				
NA*: not applicable. Note: The above table was adapted from: Hayden et al., 2013.						
**Overall opinion of study quality= Low risk of bias**						

First Author: Khanduri et al., 2013 **ID:** 6378321

**Potential Bias**	**Items to be considered for assessment of potential opportunity for bias**	**Yes**	**Partly**	**No**	**Unsure**	**NA***
Study population /sample selection	Inclusion and exclusion criteria are adequately described [including explicit diagnostic criteria, start/finish date of recruitment]	x				
	Baseline study sample [i.e. individuals entering the study and their key characteristics and sampling frame are adequately described]	x				
	Study sample represents population of interest on key characteristics, sufficient to limit potential bias to results	Low risk of bias				
Study attrition	Response rate (i.e., proportion of study sample completing the study and providing outcome data) is adequate	x				
	Participants lost to follow-up are adequately described for key characteristics	x				
	Statement as to the possible effect on the results from missing data					x
	Loss to follow-up is not associated with key characteristics	Low risk of bias				
Prognostic factor measurement	Clear definition of the prognostic factors measured is provided (e.g. imaging modality method, measurement, and timing described).	x				
	Specified instrument and personnel for measurement of predictive factors	x				
	Continuous variables are reported or appropriate (i.e. not data- dependent) cut-off points are used and specified a priori	x				
	Blinding: were estimators of risk factor status and of outcomes blinded?		x			
	The prognostic factor(s) of interest is (are) adequately measured in study participants to sufficiently limit potential bias	Low risk of bas				
Outcome measurement	Is the outcome(s) clearly defined?		x			
	The outcome measure and method used are adequately valid and reliable to limit misclassification bias	Moderate risk of bas				
Study confounding	Do the authors address potential confounders?	x				
	Important potential confounders are appropriately accounted for, limiting potential bias with respect to the prognostic factor of interest.	Low risk of bias				
Analysis and reporting	There is sufficient presentation of data to assess the adequacy of the analysis strategy and there is no selective reporting		x			
	The statistical analysis is appropriate for the study design, limiting potential for the presentation of invalid results	Moderate risk of bias				

NA*: not applicable. Note: The above table was adapted from: Hayden et al., 2013.

Overall opinion of study quality= Low risk of bias

First Author: Kumari et al., 2019 **ID:** 68614385

Potential Bias	Items to be considered for assessment of potential opportunity for bias	Yes	Partly	No	Unsure	NA*
Study population /sample selection	Inclusion and exclusion criteria are adequately described [including explicit diagnostic criteria, start/finish date of recruitment]		x			
	Baseline study sample [i.e. individuals entering the study and their key characteristics and sampling frame are adequately described]		x			
	Study sample represents population of interest on key characteristics, sufficient to limit potential bias to results	Moderate risk of bias				
Study attrition	Response rate (i.e., proportion of study sample completing the study and providing outcome data) is adequate				x	
	Participants lost to follow-up are adequately described for key characteristics				x	
	Statement as to the possible effect on the results from missing data				x	
	Loss to follow-up is not associated with key characteristics	Moderate risk of bias				
Prognostic factor measurement	Clear definition of the prognostic factors measured is provided (e.g. imaging modality method, measurement, and timing described).	x				
	Specified instrument and personnel for measurement of predictive factors	x				
	Continuous variables are reported or appropriate (i.e. not data- dependent) cut-off points are used and specified a priori	x				
	Blinding: were estimators of risk factor status and of outcomes blinded?			x		
	The prognostic factor(s) of interest is (are) adequately measured in study participants to sufficiently limit potential bias	Low risk of bias				
Outcome measurement	Is the outcome(s) clearly defined?		x			
	The outcome measure and method used are adequately valid and reliable to limit misclassification bias	Moderate risk of bias				
Study confounding	Do the authors address potential confounders?	x				
	Important potential confounders are appropriately accounted for, limiting potential bias with respect to the prognostic factor of interest.	Low risk of bias				
Analysis and reporting	There is sufficient presentation of data to assess the adequacy of the analysis strategy and there is no selective reporting	x				
	The statistical analysis is appropriate for the study design, limiting potential for the presentation of invalid results	Low risk of bias				

NA*: not applicable. Note: The above table was adapted from: Hayden et al., 2013.

Overall opinion of study quality= Moderate risk of bias

For peer review only - http://bmjopen.bmj.com/site/about/guidelines.xhtml

First Author: Lakhkar et al., 2006 **ID:** 74903014

**Potential Bias**	**Items to be considered for assessment of potential opportunity for bias**	**Yes**	**Partly**	**No**	**Unsure**	**NA***
Study population /sample selection	Inclusion and exclusion criteria are adequately described [including explicit diagnostic criteria, start/finish date of recruitment]		x			
	Baseline study sample [i.e. individuals entering the study and their key characteristics and sampling frame are adequately described]	x				
	Study sample represents population of interest on key characteristics, sufficient to limit potential bias to results	Moderate risk of bias				
Study attrition	Response rate (i.e., proportion of study sample completing the study and providing outcome data) is adequate					x
	Participants lost to follow-up are adequately described for key characteristics					x
	Statement as to the possible effect on the results from missing data					x
	Loss to follow-up is not associated with key characteristics	Low risk of bias				
Prognostic factor measurement	Clear definition of the prognostic factors measured is provided (e.g. imaging modality method, measurement, and timing described).	x				
	Specified instrument and personnel for measurement of predictive factors		x			
	Continuous variables are reported or appropriate (i.e. not data- dependent) cut-off points are used and specified a priori	x				
	Blinding: were estimators of risk factor status and of outcomes blinded?			x		
	The prognostic factor(s) of interest is (are) adequately measured in study participants to sufficiently limit potential bias	Moderate risk of bias				
Outcome measurement	Is the outcome(s) clearly defined?	x				
	The outcome measure and method used are adequately valid and reliable to limit misclassification bias	Low risk of bias				
Study confounding	Do the authors address potential confounders?	x				
	Important potential confounders are appropriately accounted for, limiting potential bias with respect to the prognostic factor of interest.	Low risk of bias				
Analysis and reporting	There is sufficient presentation of data to assess the adequacy of the analysis strategy and there is no selective reporting		x			
	The statistical analysis is appropriate for the study design, limiting potential for the presentation of invalid results	Moderate risk of bias				

NA*: not applicable. Note: The above table was adapted from: Hayden et al., 2013.

Overall opinion of study quality= Moderate risk of bias

For peer review only

First Author: Lakshmi et al., 2013 **ID:** 6378401

**Potential Bias**	**Items to be considered for assessment of potential opportunity for bias**	**Yes**	**Partly**	**No**	**Unsure**	**NA***
Study population /sample selection	Inclusion and exclusion criteria are adequately described [including explicit diagnostic criteria, start/finish date of recruitment]	x				
	Baseline study sample [i.e. individuals entering the study and their key characteristics and sampling frame are adequately described]	x				
	Study sample represents population of interest on key characteristics, sufficient to limit potential bias to results	Low risk of bias				
Study attrition	Response rate (i.e., proportion of study sample completing the study and providing outcome data) is adequate	x				
	Participants lost to follow-up are adequately described for key characteristics	x				
	Statement as to the possible effect on the results from missing data			x		
	Loss to follow-up is not associated with key characteristics	Low risk of bias				
Prognostic factor measurement	Clear definition of the prognostic factors measured is provided (e.g. imaging modality method, measurement, and timing described).	x				
	Specified instrument and personnel for measurement of predictive factors	x				
	Continuous variables are reported or appropriate (i.e. not data- dependent) cut-off points are used and specified a priori	x				
	Blinding: were estimators of risk factor status and of outcomes blinded?	x				
	The prognostic factor(s) of interest is (are) adequately measured in study participants to sufficiently limit potential bias	Low risk of bias				
Outcome measurement	Is the outcome(s) clearly defined?	x				
	The outcome measure and method used are adequately valid and reliable to limit misclassification bias	Low risk of bias				
Study confounding	Do the authors address potential confounders?	x				
	Important potential confounders are appropriately accounted for, limiting potential bias with respect to the prognostic factor of interest.	Low risk of bias				
Analysis and reporting	There is sufficient presentation of data to assess the adequacy of the analysis strategy and there is no selective reporting		x			
	The statistical analysis is appropriate for the study design, limiting potential for the presentation of invalid results	Moderate risk of bias				

NA*: not applicable. Note: The above table was adapted from: Hayden et al., 2013.

Overall opinion of study quality= Low risk of bias

First Author: Malik et al., 2013 **ID:** 6378519

**Potential Bias**	**Items to be considered for assessment of potential opportunity for bias**	**Yes**	**Partly**	**No**	**Unsure**	**NA***
Study population /sample selection	Inclusion and exclusion criteria are adequately described [including explicit diagnostic criteria, start/finish date of recruitment]	x				
	Baseline study sample [i.e. individuals entering the study and their key characteristics and sampling frame are adequately described]	x				
	Study sample represents population of interest on key characteristics, sufficient to limit potential bias to results	Low risk of bias				
Study attrition	Response rate (i.e., proportion of study sample completing the study and providing outcome data) is adequate	x				
	Participants lost to follow-up are adequately described for key characteristics			x		
	Statement as to the possible effect on the results from missing data				x	
	Loss to follow-up is not associated with key characteristics	High risk of bias				
Prognostic factor measurement	Clear definition of the prognostic factors measured is provided (e.g. imaging modality method, measurement, and timing described).			x		
	Specified instrument and personnel for measurement of predictive factors		x			
	Continuous variables are reported or appropriate (i.e. not data- dependent) cut-off points are used and specified a priori				x	
	Blinding: were estimators of risk factor status and of outcomes blinded?				x	
	The prognostic factor(s) of interest is (are) adequately measured in study participants to sufficiently limit potential bias	High risk of bias				
Outcome measurement	Is the outcome(s) clearly defined?		x			
	The outcome measure and method used are adequately valid and reliable to limit misclassification bias	Moderate risk of bias				
Study confounding	Do the authors address potential confounders?		x			
	Important potential confounders are appropriately accounted for, limiting potential bias with respect to the prognostic factor of interest.	Moderate risk of bias				
Analysis and reporting	There is sufficient presentation of data to assess the adequacy of the analysis strategy and there is no selective reporting		x			
	The statistical analysis is appropriate for the study design, limiting potential for the presentation of invalid results	Moderate risk of bias				

NA*: not applicable. Note: The above table was adapted from: Hayden et al., 2013.

Overall opinion of study quality= High risk of bias

For peer review only

First Author: Masihi et al., 2019 **ID:** 68614415

Potential Bias	Items to be considered for assessment of potential opportunity for bias	Yes	Partly	No	Unsure	NA*
Study population /sample selection	Inclusion and exclusion criteria are adequately described [including explicit diagnostic criteria, start/finish date of recruitment]	x				
	Baseline study sample [i.e. individuals entering the study and their key characteristics and sampling frame are adequately described]	x				
	Study sample represents population of interest on key characteristics, sufficient to limit potential bias to results	Low risk of bias				
Study attrition	Response rate (i.e., proportion of study sample completing the study and providing outcome data) is adequate			x		
	Participants lost to follow-up are adequately described for key characteristics			x		
	Statement as to the possible effect on the results from missing data			x		
	Loss to follow-up is not associated with key characteristics	Moderate risk of bias				
Prognostic factor measurement	Clear definition of the prognostic factors measured is provided (e.g. imaging modality method, measurement, and timing described).	x				
	Specified instrument and personnel for measurement of predictive factors			x		
	Continuous variables are reported or appropriate (i.e. not data- dependent) cut-off points are used and specified a priori	x				
	Blinding: were estimators of risk factor status and of outcomes blinded?			x		
	The prognostic factor(s) of interest is (are) adequately measured in study participants to sufficiently limit potential bias	Moderate risk of bias				
Outcome measurement	Is the outcome(s) clearly defined?	x				
	The outcome measure and method used are adequately valid and reliable to limit misclassification bias	Low risk of bias				
Study confounding	Do the authors address potential confounders?	x				
	Important potential confounders are appropriately accounted for, limiting potential bias with respect to the prognostic factor of interest.	Low risk of bias				
Analysis and reporting	There is sufficient presentation of data to assess the adequacy of the analysis strategy and there is no selective reporting	x				
	The statistical analysis is appropriate for the study design, limiting potential for the presentation of invalid results	Low risk of bias				
NA*: not applicable. Note: The above table was adapted from: Hayden et al., 2013.						
**Overall opinion of study quality= Low risk of bias**						

First Author: Mullick et al., 1993 **ID:** 6378675

**Potential Bias**	**Items to be considered for assessment of potential opportunity for bias**	**Yes**	**Partly**	**No**	**Unsure**	**NA***
Study population /sample selection	Inclusion and exclusion criteria are adequately described [including explicit diagnostic criteria, start/finish date of recruitment]	x				
	Baseline study sample [i.e. individuals entering the study and their key characteristics and sampling frame are adequately described]		x			
	Study sample represents population of interest on key characteristics, sufficient to limit potential bias to results	Low risk of bias				
Study attrition	Response rate (i.e., proportion of study sample completing the study and providing outcome data) is adequate	x				
	Participants lost to follow-up are adequately described for key characteristics	x				
	Statement as to the possible effect on the results from missing data					x
	Loss to follow-up is not associated with key characteristics	Low risk of bias				
Prognostic factor measurement	Clear definition of the prognostic factors measured is provided (e.g. imaging modality method, measurement, and timing described).	x				
	Specified instrument and personnel for measurement of predictive factors		x			
	Continuous variables are reported or appropriate (i.e. not data- dependent) cut-off points are used and specified a priori		x			
	Blinding: were estimators of risk factor status and of outcomes blinded?				x	
	The prognostic factor(s) of interest is (are) adequately measured in study participants to sufficiently limit potential bias	Moderate risk of bias				
Outcome measurement	Is the outcome(s) clearly defined?		x			
	The outcome measure and method used are adequately valid and reliable to limit misclassification bias	Moderate risk of bias				
Study confounding	Do the authors address potential confounders?		x			
	Important potential confounders are appropriately accounted for, limiting potential bias with respect to the prognostic factor of interest.	Moderate risk of bias				
Analysis and reporting	There is sufficient presentation of data to assess the adequacy of the analysis strategy and there is no selective reporting		x			
	The statistical analysis is appropriate for the study design, limiting potential for the presentation of invalid results	Moderate risk of bias				

NA*: not applicable. Note: The above table was adapted from: Hayden et al., 2013.

Overall opinion of study quality= Moderate risk of bias

First Author: Nagar et al., 2015 **ID:** 6378692

**Potential Bias**	**Items to be considered for assessment of potential opportunity for bias**	**Yes**	**Partly**	**No**	**Unsure**	**NA***
Study population /sample selection	Inclusion and exclusion criteria are adequately described [including explicit diagnostic criteria, start/finish date of recruitment]	x				
	Baseline study sample [i.e. individuals entering the study and their key characteristics and sampling frame are adequately described]		x			
	Study sample represents population of interest on key characteristics, sufficient to limit potential bias to results	Low risk of bias				
Study attrition	Response rate (i.e., proportion of study sample completing the study and providing outcome data) is adequate	x				
	Participants lost to follow-up are adequately described for key characteristics				x	
	Statement as to the possible effect on the results from missing data					x
	Loss to follow-up is not associated with key characteristics	Low risk of bias				
Prognostic factor measurement	Clear definition of the prognostic factors measured is provided (e.g. imaging modality method, measurement, and timing described).	x				
	Specified instrument and personnel for measurement of predictive factors			x		
	Continuous variables are reported or appropriate (i.e. not data- dependent) cut-off points are used and specified a priori	x				
	Blinding: were estimators of risk factor status and of outcomes blinded?			x		
	The prognostic factor(s) of interest is (are) adequately measured in study participants to sufficiently limit potential bias	Moderate risk of bias				
Outcome measurement	Is the outcome(s) clearly defined?		x			
	The outcome measure and method used are adequately valid and reliable to limit misclassification bias	Moderate risk of bias				
Study confounding	Do the authors address potential confounders?	x				
	Important potential confounders are appropriately accounted for, limiting potential bias with respect to the prognostic factor of interest.	Low risk of bias				
Analysis and reporting	There is sufficient presentation of data to assess the adequacy of the analysis strategy and there is no selective reporting		x			
	The statistical analysis is appropriate for the study design, limiting potential for the presentation of invalid results	Moderate risk of bias				

NA*: not applicable. Note: The above table was adapted from: Hayden et al., 2013.

Overall opinion of study quality= Moderate risk of bias

First Author: Najam et al., 2016 **ID:** 6378705

**Potential Bias**	**Items to be considered for assessment of potential opportunity for bias**	**Yes**	**Partly**	**No**	**Unsure**	**NA***
Study population /sample selection	Inclusion and exclusion criteria are adequately described [including explicit diagnostic criteria, start/finish date of recruitment]			x		
	Baseline study sample [i.e. individuals entering the study and their key characteristics and sampling frame are adequately described]			x		
	Study sample represents population of interest on key characteristics, sufficient to limit potential bias to results	High risk of bias				
Study attrition	Response rate (i.e., proportion of study sample completing the study and providing outcome data) is adequate		x			
	Participants lost to follow-up are adequately described for key characteristics		x			
	Statement as to the possible effect on the results from missing data			x		
	Loss to follow-up is not associated with key characteristics	High risk of bias				
Prognostic factor measurement	Clear definition of the prognostic factors measured is provided (e.g. imaging modality method, measurement, and timing described).	x				
	Specified instrument and personnel for measurement of predictive factors			x		
	Continuous variables are reported or appropriate (i.e. not data- dependent) cut-off points are used and specified a priori	x				
	Blinding: were estimators of risk factor status and of outcomes blinded?			x		
	The prognostic factor(s) of interest is (are) adequately measured in study participants to sufficiently limit potential bias	Moderate risk of bias				
Outcome measurement	Is the outcome(s) clearly defined?			x		
	The outcome measure and method used are adequately valid and reliable to limit misclassification bias	High risk of bias				
Study confounding	Do the authors address potential confounders?				x	
	Important potential confounders are appropriately accounted for, limiting potential bias with respect to the prognostic factor of interest.	High risk of bias				
Analysis and reporting	There is sufficient presentation of data to assess the adequacy of the analysis strategy and there is no selective reporting			x		
	The statistical analysis is appropriate for the study design, limiting potential for the presentation of invalid results	High risk of bias				

NA*: not applicable. Note: The above table was adapted from: Hayden et al., 2013.

Overall opinion of study quality= High risk of bias

First Author: Nouh et al., 2011 **ID:** 6378752

Potential Bias	Items to be considered for assessment of potential opportunity for bias	Yes	Partly	No	Unsure	NA*
Study population /sample selection	Inclusion and exclusion criteria are adequately described [including explicit diagnostic criteria, start/finish date of recruitment]	x				
	Baseline study sample [i.e. individuals entering the study and their key characteristics and sampling frame are adequately described]	x				
	Study sample represents population of interest on key characteristics, sufficient to limit potential bias to results	Low risk of bias				
Study attrition	Response rate (i.e., proportion of study sample completing the study and providing outcome data) is adequate	x				
	Participants lost to follow-up are adequately described for key characteristics					x
	Statement as to the possible effect on the results from missing data				x	
	Loss to follow-up is not associated with key characteristics	Low risk of bias				
Prognostic factor measurement	Clear definition of the prognostic factors measured is provided (e.g. imaging modality method, measurement, and timing described).	x				
	Specified instrument and personnel for measurement of predictive factors	x				
	Continuous variables are reported or appropriate (i.e. not data- dependent) cut-off points are used and specified a priori	x				
	Blinding: were estimators of risk factor status and of outcomes blinded?				x	
	The prognostic factor(s) of interest is (are) adequately measured in study participants to sufficiently limit potential bias	Low risk of bias				
Outcome measurement	Is the outcome(s) clearly defined?		x			
	The outcome measure and method used are adequately valid and reliable to limit misclassification bias	Low risk of bias				
Study confounding	Do the authors address potential confounders?	x				
	Important potential confounders are appropriately accounted for, limiting potential bias with respect to the prognostic factor of interest.	Low risk of bias				
Analysis and reporting	There is sufficient presentation of data to assess the adequacy of the analysis strategy and there is no selective reporting		x			
	The statistical analysis is appropriate for the study design, limiting potential for the presentation of invalid results	Moderate risk of bias				

NA*: not applicable. Note: The above table was adapted from: Hayden et al., 2013.

Overall opinion of study quality= Low risk of bias

First Author: Pares et al., 2008 **ID:** 6378809

**Potential Bias**	**Items to be considered for assessment of potential opportunity for bias**	**Yes**	**Partly**	**No**	**Unsure**	**NA***
Study population /sample selection	Inclusion and exclusion criteria are adequately described [including explicit diagnostic criteria, start/finish date of recruitment]	x				
	Baseline study sample [i.e. individuals entering the study and their key characteristics and sampling frame are adequately described]	x				
	Study sample represents population of interest on key characteristics, sufficient to limit potential bias to results	Low risk of bias				
Study attrition	Response rate (i.e., proportion of study sample completing the study and providing outcome data) is adequate	x				
	Participants lost to follow-up are adequately described for key characteristics					x
	Statement as to the possible effect on the results from missing data					x
	Loss to follow-up is not associated with key characteristics	Low risk of bias				
Prognostic factor measurement	Clear definition of the prognostic factors measured is provided (e.g. imaging modality method, measurement, and timing described).	x				
	Specified instrument and personnel for measurement of predictive factors	x				
	Continuous variables are reported or appropriate (i.e. not data- dependent) cut-off points are used and specified a priori	x				
	Blinding: were estimators of risk factor status and of outcomes blinded?				x	
	The prognostic factor(s) of interest is (are) adequately measured in study participants to sufficiently limit potential bias	Moderate risk of bias				
Outcome measurement	Is the outcome(s) clearly defined?	x				
	The outcome measure and method used are adequately valid and reliable to limit misclassification bias	Low risk of bias				
Study confounding	Do the authors address potential confounders?	x				
	Important potential confounders are appropriately accounted for, limiting potential bias with respect to the prognostic factor of interest.	Low risk of bias				
Analysis and reporting	There is sufficient presentation of data to assess the adequacy of the analysis strategy and there is no selective reporting	x				
	The statistical analysis is appropriate for the study design, limiting potential for the presentation of invalid results	Low risk of bias				

NA*: not applicable. Note: The above table was adapted from: Hayden et al., 2013.

Overall opinion of study quality= Low risk of bias

For peer review only

First Author: Pattinson et al., 1991 **ID:** 74903015

Potential Bias	Items to be considered for assessment of potential opportunity for bias	Yes	Partly	No	Unsure	NA*
Study population /sample selection	Inclusion and exclusion criteria are adequately described [including explicit diagnostic criteria, start/finish date of recruitment]	x				
	Baseline study sample [i.e. individuals entering the study and their key characteristics and sampling frame are adequately described]	x				
	Study sample represents population of interest on key characteristics, sufficient to limit potential bias to results	Low risk of bias				
Study attrition	Response rate (i.e., proportion of study sample completing the study and providing outcome data) is adequate					x
	Participants lost to follow-up are adequately described for key characteristics					x
	Statement as to the possible effect on the results from missing data					x
	Loss to follow-up is not associated with key characteristics	Low risk of bias				
Prognostic factor measurement	Clear definition of the prognostic factors measured is provided (e.g. imaging modality method, measurement, and timing described).	x				
	Specified instrument and personnel for measurement of predictive factors		x			
	Continuous variables are reported or appropriate (i.e. not data- dependent) cut-off points are used and specified a priori	x				
	Blinding: were estimators of risk factor status and of outcomes blinded?	x				
	The prognostic factor(s) of interest is (are) adequately measured in study participants to sufficiently limit potential bias	Low risk of bias				
Outcome measurement	Is the outcome(s) clearly defined?		x			
	The outcome measure and method used are adequately valid and reliable to limit misclassification bias	Moderate risk of bias				
Study confounding	Do the authors address potential confounders?	x				
	Important potential confounders are appropriately accounted for, limiting potential bias with respect to the prognostic factor of interest.	Low risk of bias				
Analysis and reporting	There is sufficient presentation of data to assess the adequacy of the analysis strategy and there is no selective reporting		x			
	The statistical analysis is appropriate for the study design, limiting potential for the presentation of invalid results	Moderate risk of bias				

NA*: not applicable. Note: The above table was adapted from: Hayden et al., 2013.

Overall opinion of study quality= Low risk of bias

First Author: Pattinson et al., 1993 **ID:** 6378815

**Potential Bias**	**Items to be considered for assessment of potential opportunity for bias**	**Yes**	**Partly**	**No**	**Unsure**	**NA***
Study population /sample selection	Inclusion and exclusion criteria are adequately described [including explicit diagnostic criteria, start/finish date of recruitment]	x				
	Baseline study sample [i.e. individuals entering the study and their key characteristics and sampling frame are adequately described]	x				
	Study sample represents population of interest on key characteristics, sufficient to limit potential bias to results	Low risk of bias				
Study attrition	Response rate (i.e., proportion of study sample completing the study and providing outcome data) is adequate	x				
	Participants lost to follow-up are adequately described for key characteristics	x				
	Statement as to the possible effect on the results from missing data				x	
	Loss to follow-up is not associated with key characteristics	Low risk of bias				
Prognostic factor measurement	Clear definition of the prognostic factors measured is provided (e.g. imaging modality method, measurement, and timing described).	x				
	Specified instrument and personnel for measurement of predictive factors		x			
	Continuous variables are reported or appropriate (i.e. not data- dependent) cut-off points are used and specified a priori	x				
	Blinding: were estimators of risk factor status and of outcomes blinded?				x	
	The prognostic factor(s) of interest is (are) adequately measured in study participants to sufficiently limit potential bias	Moderate risk of bias				
Outcome measurement	Is the outcome(s) clearly defined?	x				
	The outcome measure and method used are adequately valid and reliable to limit misclassification bias	Low risk of bias				
Study confounding	Do the authors address potential confounders?	x				
	Important potential confounders are appropriately accounted for, limiting potential bias with respect to the prognostic factor of interest.	Low risk of bias				
Analysis and reporting	There is sufficient presentation of data to assess the adequacy of the analysis strategy and there is no selective reporting	x				
	The statistical analysis is appropriate for the study design, limiting potential for the presentation of invalid results	Low risk of bias				

NA*: not applicable. Note: The above table was adapted from: Hayden et al., 2013.

Overall opinion of study quality= Low risk of bias

For peer review only

First Author: Phupong et al., 2003 **ID:** 6378830

Potential Bias	Items to be considered for assessment of potential opportunity for bias	Yes	Partly	No	Unsure	NA*
Study population /sample selection	Inclusion and exclusion criteria are adequately described [including explicit diagnostic criteria, start/finish date of recruitment]	x				
	Baseline study sample [i.e. individuals entering the study and their key characteristics and sampling frame are adequately described]	x				
	Study sample represents population of interest on key characteristics, sufficient to limit potential bias to results	Low risk of bias				
Study attrition	Response rate (i.e., proportion of study sample completing the study and providing outcome data) is adequate	x				
	Participants lost to follow-up are adequately described for key characteristics	x				
	Statement as to the possible effect on the results from missing data					x
	Loss to follow-up is not associated with key characteristics	Low risk of bias				
Prognostic factor measurement	Clear definition of the prognostic factors measured is provided (e.g. imaging modality method, measurement, and timing described).	x				
	Specified instrument and personnel for measurement of predictive factors	x				
	Continuous variables are reported or appropriate (i.e. not data- dependent) cut-off points are used and specified a priori	x				
	Blinding: were estimators of risk factor status and of outcomes blinded?	x				
	The prognostic factor(s) of interest is (are) adequately measured in study participants to sufficiently limit potential bias	Low risk of bias				
Outcome measurement	Is the outcome(s) clearly defined?	x				
	The outcome measure and method used are adequately valid and reliable to limit misclassification bias	Low risk of bias				
Study confounding	Do the authors address potential confounders?	x				
	Important potential confounders are appropriately accounted for, limiting potential bias with respect to the prognostic factor of interest.	Low risk of bias				
Analysis and reporting	There is sufficient presentation of data to assess the adequacy of the analysis strategy and there is no selective reporting	x				
	The statistical analysis is appropriate for the study design, limiting potential for the presentation of invalid results	Low risk of bias				

NA*: not applicable. Note: The above table was adapted from: Hayden et al., 2013.

Overall opinion of study quality= Low risk of bias

First Author: Rani et al., 2016 **ID:** 74903020

Potential Bias	Items to be considered for assessment of potential opportunity for bias	Yes	Partly	No	Unsure	NA*
Study population /sample selection	Inclusion and exclusion criteria are adequately described [including explicit diagnostic criteria, start/finish date of recruitment]	x				
	Baseline study sample [i.e. individuals entering the study and their key characteristics and sampling frame are adequately described]		x			
	Study sample represents population of interest on key characteristics, sufficient to limit potential bias to results	Moderate risk of bias				
Study attrition	Response rate (i.e., proportion of study sample completing the study and providing outcome data) is adequate					x
	Participants lost to follow-up are adequately described for key characteristics					x
	Statement as to the possible effect on the results from missing data					x
	Loss to follow-up is not associated with key characteristics	Low risk of bias				
Prognostic factor measurement	Clear definition of the prognostic factors measured is provided (e.g. imaging modality method, measurement, and timing described).	x				
	Specified instrument and personnel for measurement of predictive factors		x			
	Continuous variables are reported or appropriate (i.e. not data- dependent) cut-off points are used and specified a priori		x			
	Blinding: were estimators of risk factor status and of outcomes blinded?			x		
	The prognostic factor(s) of interest is (are) adequately measured in study participants to sufficiently limit potential bias	Moderate risk of bias				
Outcome measurement	Is the outcome(s) clearly defined?	x				
	The outcome measure and method used are adequately valid and reliable to limit misclassification bias	Low risk of bias				
Study confounding	Do the authors address potential confounders?		x			
	Important potential confounders are appropriately accounted for, limiting potential bias with respect to the prognostic factor of interest.	Moderate risk of bias				
Analysis and reporting	There is sufficient presentation of data to assess the adequacy of the analysis strategy and there is no selective reporting		x			
	The statistical analysis is appropriate for the study design, limiting potential for the presentation of invalid results	Moderate risk of bias				
NA*: not applicable. Note: The above table was adapted from: Hayden et al., 2013.						
**Overall opinion of study quality= Moderate risk of bias**						

First Author: Rocca et al., 1995 **ID:** 74903016

Potential Bias	Items to be considered for assessment of potential opportunity for bias	Yes	Partly	No	Unsure	NA*
Study population /sample selection	Inclusion and exclusion criteria are adequately described [including explicit diagnostic criteria, start/finish date of recruitment]	x				
	Baseline study sample [i.e. individuals entering the study and their key characteristics and sampling frame are adequately described]		x			
	Study sample represents population of interest on key characteristics, sufficient to limit potential bias to results	Moderate risk of bias				
Study attrition	Response rate (i.e., proportion of study sample completing the study and providing outcome data) is adequate					x
	Participants lost to follow-up are adequately described for key characteristics					x
	Statement as to the possible effect on the results from missing data					x
	Loss to follow-up is not associated with key characteristics	Low risk of bias				
Prognostic factor measurement	Clear definition of the prognostic factors measured is provided (e.g. imaging modality method, measurement, and timing described).	x				
	Specified instrument and personnel for measurement of predictive factors		x			
	Continuous variables are reported or appropriate (i.e. not data- dependent) cut-off points are used and specified a priori	x				
	Blinding: were estimators of risk factor status and of outcomes blinded?			x		
	The prognostic factor(s) of interest is (are) adequately measured in study participants to sufficiently limit potential bias	Moderate risk of bias				
Outcome measurement	Is the outcome(s) clearly defined?		x			
	The outcome measure and method used are adequately valid and reliable to limit misclassification bias	Moderate risk of bias				
Study confounding	Do the authors address potential confounders?		x			
	Important potential confounders are appropriately accounted for, limiting potential bias with respect to the prognostic factor of interest.	Moderate risk of bias				
Analysis and reporting	There is sufficient presentation of data to assess the adequacy of the analysis strategy and there is no selective reporting		x			
	The statistical analysis is appropriate for the study design, limiting potential for the presentation of invalid results	Moderate risk of bias				

NA*: not applicable. Note: The above table was adapted from: Hayden et al., 2013.

Overall opinion of study quality= Moderate risk of bias

First Author: Verma et al., 2016 **ID:** 6379243

Potential Bias	Items to be considered for assessment of potential opportunity for bias	Yes	Partly	No	Unsure	NA*
Study population /sample selection	Inclusion and exclusion criteria are adequately described [including explicit diagnostic criteria, start/finish date of recruitment]	x				
	Baseline study sample [i.e. individuals entering the study and their key characteristics and sampling frame are adequately described]	x				
	Study sample represents population of interest on key characteristics, sufficient to limit potential bias to results	Low risk of bias				
Study attrition	Response rate (i.e., proportion of study sample completing the study and providing outcome data) is adequate	x				
	Participants lost to follow-up are adequately described for key characteristics					x
	Statement as to the possible effect on the results from missing data				x	
	Loss to follow-up is not associated with key characteristics	Low risk of bias				
Prognostic factor measurement	Clear definition of the prognostic factors measured is provided (e.g. imaging modality method, measurement, and timing described).	x				
	Specified instrument and personnel for measurement of predictive factors	x				
	Continuous variables are reported or appropriate (i.e. not data- dependent) cut-off points are used and specified a priori	x				
	Blinding: were estimators of risk factor status and of outcomes blinded?				x	
	The prognostic factor(s) of interest is (are) adequately measured in study participants to sufficiently limit potential bias	Low risk of bias				
Outcome measurement	Is the outcome(s) clearly defined?	x				
	The outcome measure and method used are adequately valid and reliable to limit misclassification bias	Low risk of bias				
Study confounding	Do the authors address potential confounders?	x				
	Important potential confounders are appropriately accounted for, limiting potential bias with respect to the prognostic factor of interest.	Low risk of bias				
Analysis and reporting	There is sufficient presentation of data to assess the adequacy of the analysis strategy and there is no selective reporting	x				
	The statistical analysis is appropriate for the study design, limiting potential for the presentation of invalid results	Low risk of bias				

NA*: not applicable. Note: The above table was adapted from: Hayden et al., 2013.

Overall opinion of study quality= Low risk of bias

First Author: Waa et al., 2010 **ID:** 6379255

Potential Bias	Items to be considered for assessment of potential opportunity for bias	Yes	Partly	No	Unsure	NA*
Study population /sample selection	Inclusion and exclusion criteria are adequately described [including explicit diagnostic criteria, start/finish date of recruitment]	x				
	Baseline study sample [i.e. individuals entering the study and their key characteristics and sampling frame are adequately described]	x				
	Study sample represents population of interest on key characteristics, sufficient to limit potential bias to results	Low risk of bias				
Study attrition	Response rate (i.e., proportion of study sample completing the study and providing outcome data) is adequate	x				
	Participants lost to follow-up are adequately described for key characteristics	x				
	Statement as to the possible effect on the results from missing data	x				
	Loss to follow-up is not associated with key characteristics	Low risk of bias				
Prognostic factor measurement	Clear definition of the prognostic factors measured is provided (e.g. imaging modality method, measurement, and timing described).	x				
	Specified instrument and personnel for measurement of predictive factors	x				
	Continuous variables are reported or appropriate (i.e. not data- dependent) cut-off points are used and specified a priori	x				
	Blinding: were estimators of risk factor status and of outcomes blinded?			x		
	The prognostic factor(s) of interest is (are) adequately measured in study participants to sufficiently limit potential bias	Low risk of bias				
Outcome measurement	Is the outcome(s) clearly defined?		x			
	The outcome measure and method used are adequately valid and reliable to limit misclassification bias	Moderate risk of bias				
Study confounding	Do the authors address potential confounders?		x			
	Important potential confounders are appropriately accounted for, limiting potential bias with respect to the prognostic factor of interest.	Moderate risk of bias				
Analysis and reporting	There is sufficient presentation of data to assess the adequacy of the analysis strategy and there is no selective reporting		x			
	The statistical analysis is appropriate for the study design, limiting potential for the presentation of invalid results	Moderate risk of bias				
NA*: not applicable. Note: The above table was adapted from: Hayden et al., 2013.						
**Overall opinion of study quality= Moderate risk of bias**						

First Author: Yelikar et al., 2013 **ID:** 6379339

Potential Bias	Items to be considered for assessment of potential opportunity for bias	Yes	Partly	No	Unsure	NA*
Study population /sample selection	Inclusion and exclusion criteria are adequately described [including explicit diagnostic criteria, start/finish date of recruitment]	x				
	Baseline study sample [i.e. individuals entering the study and their key characteristics and sampling frame are adequately described]	x				
	Study sample represents population of interest on key characteristics, sufficient to limit potential bias to results	Low risk of bias				
Study attrition	Response rate (i.e., proportion of study sample completing the study and providing outcome data) is adequate		x			
	Participants lost to follow-up are adequately described for key characteristics				x	
	Statement as to the possible effect on the results from missing data				x	
	Loss to follow-up is not associated with key characteristics	Moderate risk of bias				
Prognostic factor measurement	Clear definition of the prognostic factors measured is provided (e.g. imaging modality method, measurement, and timing described).	x				
	Specified instrument and personnel for measurement of predictive factors		x			
	Continuous variables are reported or appropriate (i.e. not data- dependent) cut-off points are used and specified a priori	x				
	Blinding: were estimators of risk factor status and of outcomes blinded?			x		
	The prognostic factor(s) of interest is (are) adequately measured in study participants to sufficiently limit potential bias	Moderate risk of bias				
Outcome measurement	Is the outcome(s) clearly defined?		x			
	The outcome measure and method used are adequately valid and reliable to limit misclassification bias	Moderate risk of bias				
Study confounding	Do the authors address potential confounders?	x				
	Important potential confounders are appropriately accounted for, limiting potential bias with respect to the prognostic factor of interest.	Low risk of bias				
Analysis and reporting	There is sufficient presentation of data to assess the adequacy of the analysis strategy and there is no selective reporting		x			
	The statistical analysis is appropriate for the study design, limiting potential for the presentation of invalid results	Moderate risk of bias				

NA*: not applicable. Note: The above table was adapted from: Hayden et al., 2013.

Overall opinion of study quality= Moderate risk of bias

First Author: Zarean et al., 2018 **ID:** 6379369

**Potential Bias**	**Items to be considered for assessment of potential opportunity for bias**	**Yes**	**Partly**	**No**	**Unsure**	**NA***
Study population /sample selection	Inclusion and exclusion criteria are adequately described [including explicit diagnostic criteria, start/finish date of recruitment]	x				
	Baseline study sample [i.e. individuals entering the study and their key characteristics and sampling frame are adequately described]		x			
	Study sample represents population of interest on key characteristics, sufficient to limit potential bias to results	Moderate risk of bias				
Study attrition	Response rate (i.e., proportion of study sample completing the study and providing outcome data) is adequate	x				
	Participants lost to follow-up are adequately described for key characteristics					x
	Statement as to the possible effect on the results from missing data					x
	Loss to follow-up is not associated with key characteristics	Low risk of bias				
Prognostic factor measurement	Clear definition of the prognostic factors measured is provided (e.g. imaging modality method, measurement, and timing described).	x				
	Specified instrument and personnel for measurement of predictive factors	x				
	Continuous variables are reported or appropriate (i.e. not data- dependent) cut-off points are used and specified a priori	x				
	Blinding: were estimators of risk factor status and of outcomes blinded?				x	
	The prognostic factor(s) of interest is (are) adequately measured in study participants to sufficiently limit potential bias	Low risk of bias				
Outcome measurement	Is the outcome(s) clearly defined?		x			
	The outcome measure and method used are adequately valid and reliable to limit misclassification bias	Moderate risk of bias				
Study confounding	Do the authors address potential confounders?	x				
	Important potential confounders are appropriately accounted for, limiting potential bias with respect to the prognostic factor of interest.	Low risk of bias				
Analysis and reporting	There is sufficient presentation of data to assess the adequacy of the analysis strategy and there is no selective reporting	x				
	The statistical analysis is appropriate for the study design, limiting potential for the presentation of invalid results	Low risk of bias				
NA*: not applicable. Note: The above table was adapted from: Hayden et al., 2013.						
**Overall opinion of study quality= Low risk of bias**						

Table S1. Statistical measures of prognostic performance of Doppler ultrasound reported in the selected studies

Prognostic determinant	Outcome	Studies	Sn	Sp	PPV	NPV	AUROC	Diagnostic accuracy	OR [95% CI]	RR [95% CI]	Correlation	Normal Doppler n (%)	Abnormal Doppler n (%)
**UA flow impedance**	FGR	Agbaje et al., 2018	67.00	53.00			0.63						
		Mullick et al., 1993	85.00	89.00	88.50								
		Najam et al., 2016	48.15	80.67	53.06	77.40							
		Rocca et al., 1995	92.30	91.90	77.40	97.60		92.0					
		Khanduri et al., 2013	73.80	75.90	87.70	55.40		75.00					
		Bano et al., 2010	46.70	93.30	87.50	63.60		70.00					
		Nagar et al., 2015	42.86	94.62	37.50	95.65							
	NICU Admission	Anshul et al., 2010										13 (24.07)	36 (78.2)
		Najam et al., 2016	50.00	80.30	48.90	80.95							
	Fetal Distress	Anshul et al., 2010										18 (33)	35 (76)
		Rocca et al., 1995										2 (2.5)	12 (39)
		Najam et al., 2016	66.67	78.04	74.89	89.72							
		Yelikar et al., 2013	42.10	65.90	12.10	91.10							
	Stillbirth	Anshul et al., 2010										0 (0)	4 (9.5)
		Najam et al., 2016										0 (0)	5 (8.2)
	Perinatal death	Rocca et al., 1995										0 (0)	2 (6.5)
		Anshul et al., 2010										0 (0)	9 (60)
	LBW	Anshul et al., 2010										15 (27.0)	35 (77.8)
	Apgar Score	Rocca et al., 1995	80.00	82.40	41.00	96.00		83.00					
		Anshul et al., 2010										2 (3.7)	14 (82.35)
		Najam et al., 2016										3 (60.0)	6 (85.71)
		Agbaje et al., 2018									0.378		
	Fetal Anemia	Kumari et al., 2019									0.21		
	HIE	Najam et al., 2016										1 (1.29)	8 (16.31)
	MAS	Najam et al., 2016										1 (1.29)	16 (32.65)
	CAPO	Bano et al., 2010	79.20	92.40	79.20	92.20		88.90					
		Lakhkar et al 2006	50.00	59.00	66.60	41.90							

For peer review only

		Rani et al., 2016	17.80	95.80	80.70	50.50	0.57						
		Geerts et al., 2007	75.00			95.00			0.6 (0.1, 4.1)				
		Malik et al., 2013	64.40	80.00	96.60	20.00							
		Pattinson et al., 1993	12.50	91.80	22.70	84.50							
		Ebrashy et al., 2005	53.30	36.40	81.10	30.80							
		Waa et al., 2010	8.00	100.00	0.00	26.00							
**UA AREDF**	Perinatal death	Lakshmi et al., 2013							9.8 (2.1, 46.4)				
		Najam et al., 2016										2 (2.59)	4 (33.33)
	RDS	Lakshmi et al., 2013							2.4 (1.1, 5.0)				
	CAPO	Pattinson et al., 1991	75.00	90.00	69.00								
		Lakshmi et al., 2013							8.4 (2.3, 30.5)				
**MCA flow impedance**	FGR	Najam et al., 2016	59.25	88.89	72.72	81.35							
		Bano et al., 2010	8.90	100.0	100.0	52.30		54.40					
		Khanduri et al., 2013	26.20	92.60	89.20	35.00		46.10					
	Fetal Anemia	Pares et al., 2008	100.00	65.00	90.90	100.0		92.20					
		Kumari et al., 2019	68.00	57.00	83.00	33.00	0.70				-0.43		
	NICU Admission	Najam et al., 2016	64.58	88.69	70.45	85.71							
	Neonatal Acidosis	Allam et al., 2013	87.50	64.00	74.00	82.00	0.82						
	Fetal Distress	Najam et al., 2016	72.73	78.05	54.55	91.53							
	Stillbirth	Najam et al., 2016										0 (0)	2 (4.5)
	Apgar Score	Najam et al., 2016										1 (1.29)	17 (38.6)
	HIE	Najam et al., 2016										1 (1.29)	10 (22.72)
	MAS	Najam et al., 2016										1 (1.29)	20 (45.5)
	CAPO	Bano et al., 2010	16.70	100.0	100.0	76.70		77.80					
		Lakhkar et al 2006	41.60	90.90	88.20	48.70							
		Rani et al., 2016	18.60	90.30	68.70	49.40	0.58						
		Dhand et al., 2011	71.00	92.00	94.00	65.00							
		Malik et al., 2013	7.70	90.00	87.50	9.80							
		Ebrashy et al., 2005	41.00	63.60	80.00	23.30							
		Waa et al., 2010	23.0	68.00	76.00	33.00							

For peer review only

**CPR**	FGR	Najam et al., 2016	85.10	89.72	80.70	92.30							
		Bano et al., 2010						72.20					
	NICU Admission	Najam et al., 2016	75.00	82.92	63.15	89.47							
		Alanwar et al., 2018	62.50	71.42	29.40	90.90							
	Foetal Distress	Najam et al., 2016	90.91	78.04	52.63	96.97							
		Masihi et al.2019	80.95	50.00	17.50	95.20							
	Stillbirth	Najam et al., 2016										0 (0)	4 (7.14)
	Apgar Score	Najam et al., 2016										1 (1.29)	19 (33.33)
		Alanwar et al., 2018	50.0	88.10	44.40	90.20							
	Neonatal Acidosis	Ebrashy et al., 2005	64.10	72.70	89.30	36.40				1.4 (1.2, 1.7)			
		Alanwar et al., 2018	43.75	69.05	21.21	86.57							
	HIE	Najam et al., 2016										1 (1.29)	12 (21.05)
	MAS	Najam et al., 2016	96.15			99.20						1 (1.29)	25 (43.85)
	CAPO	Bano et al., 2010	83.30	100.0	100.00	94.30		95.60					
		Lakhkar et al 2006	47.20	86.30	85.00	50.00							
		Rani et al., 2016	7.60	98.00	81.80	48.30	0.60						
		Malik et al., 2013	68.80	100.00	100.0	26.30							
		Geerts et al., 2007			57.0				1.1 (0.1, 14.6)				
**UtA flow impedance**	FGR	Verma et al., 2016	45.0	84.10	28.10	91.70							
		Phupong et al., 2003	67.0	82.90	6.90	99.20				9.1 (1.7, 48.5)			
		Nagar et al., 2015	25.0	94.56	28.57	93.55							
	Perinatal Death	Dorman et al., 2002								2.37 (1.3, 4.3)			
	LBW	Verma et al., 2016	45.40	84.60	31.30	90.90							
		Dorman et al., 2002								2.52 (1.5, 4.2)			
	Preterm Birth	Verma et al., 2016	57.10	63.20	18.50	91.00							
		Dorman et al., 2002								1.53 (0.9, 2.4)			

For peer review only

	CAPO	Verma et al., 2016	48.20	95.40	84.40	78.20							
		Nouh et al., 2011	84.60	96.30	91.70	92.90							
		Malik et al., 2013	37.70	70.00	91.80	11.00							
		Zarean et al., 2018	37.50	73.30	48.40	63.70	0.55						
**FDA flow impedance**	Fetal anemia	Pares et al., 2008	95.70	100.0	100.0	86.90		96.70					
		Kumari et al., 2019	87.00	57.00			0.80				-0.54		
	CAPO	Lakhkar et al 2006	44.40	59.00	64.00	56.50							
**FDA & MCA**	Fetal anemia	Pares et al., 2008	98.40	100.0	100.0	91.70		98.60					
		Kumari et al., 2019	86.00	67.00	86.00	67.00							
**DV flow impedance**	Neonatal Acidosis	Allam et al., 2013	100.0	57.00	72.0	100.0	0.88	80.00					
	CAPO	Geerts et al., 2007		92.0	33.0				0.3 (0.03, 4.6)				

[a]UA: umbilical artery; MCA: middle cerebral artery; CPR: cerebroplacental ratio; UtA: uterine artery; FDA: fetal descending aorta; DV: ductus venosus; RI: resistive index; PI: pulsatility index; S/D ratio: systolic diastolic ratio; PSV: peak systolic velocity; MV: mean velocity; AREDF: absent and/or reversed end diastolic flow; FGR: fetal growth restriction; LBW: low birth weight; HIE: hypoxic ischemic encephalopathy; MAS: meconium aspiration syndrome; RDS: respiratory distress syndrome; NICU: neonatal intensive care unit; CAPO: composite adverse perinatal outcomes; Sn: sensitivity; Sp: specificity; PPV: positive predictive value; NPV: negative predictive value; OR: odds ratio; RR: relative risk; and n (%): frequency (percentage).

For peer review only

Table S2. Definitions of adverse perinatal outcomes reported in the selected studies

First Author	Outcomes	Definition (detailed description in the article)
Abdallah et al., 2019	LBW	Not defined
	NICU admission	Not defined
	Stillbirth	Not defined
	Perinatal mortality	Not defined
	Low APGAR score (1min & 5min)	Not defined
Agbaje et al., 2018	FGR	Abnormal birth weight: defined as estimated foetal weight below the 10th percentile for gestational age and abdominal circumference below the 10th percentile for gestational age.
	Low APGAR score at 5 minutes	APGAR score less than 6
Alanwar et al., 2018	Acidosis	Neonatal academia of pH < 7.2
	NICU admission	New-born was admitted to the neo- natal intensive care unit
	Low APGAR score at 5 minutes	APGAR score < 7 at 5 min
Allam et al., 2013	Neonatal acidosis	Cord blood pH <7.25
Anshul et al., 2010	Stillbirth	Not defined
	Neonatal death	Not defined
	NICU admission	Admission required
	Foetal distress	Delivered by emergency caesarean section for suspected foetal distress
	LBW	Not defined
	Low APGAR score at birth.	APGAR score <7 at birth
Bano et al., 2010	Perinatal death	Not defined
	Foetal distress	Caesarean section for foetal distress (FD not defined)
	NICU admission	Not defined
	Low APGAR score at 5min	APGAR score <7 at 5 min
	FGR	Birth weight less than 10th percentile for gestational age

For peer review only

	Composite adverse perinatal outcome	Not defined
Dhand et al., 2011	Composite adverse perinatal outcome	Abnormal foetal outcome (details not provided)
Dorman et al., 2002	Perinatal death	Not defined
	Preterm delivery	Delivery < 37 weeks
	LBW	Birth weight <2.5kg
Ebrashy et al., 2005	Acidosis	Neonatal acidaemia of pH<7.2 were present
	Composite adverse neonatal outcome	Neonatal morbidity (neonatal academia pH<7.2, 5-minute APGAR score <6, and/or admission to NICU)
Geerts et al., 2007	Composite adverse perinatal outcome	Poor outcome (perinatal demise or clinical/ultrasound signs of neurological compromise in the infant at the time of discharge from the tertiary institution)
Khanduri et al., 2013	FGR	Ponderal index was calculated as birth weight (in gm) per length (in cm^3). Ponderal index of <10 indicates growth restriction.
Kumari et al., 2019	Foetal anaemia	Haematocrit of the umbilical cord blood was used as the reference test to diagnose foetal anaemia (defined as haemoglobin <0.65 times the median for gestational age).
Lakhkar et al., 2006	Composite adverse perinatal outcome	Adverse perinatal outcome (Major and Minor). Major adverse outcomes were perinatal deaths including intrauterine and early neonatal deaths. Major complications like hypoxic ischemic encephalopathy, intraventricular haemorrhage, periventricular leukomalacia, pulmonary haemorrhage and necrotizing enterocolitis. Minor outcomes include-caesarean delivery for foetal distress, APGAR score below 7 at 5 minutes, admission to NICU (neonatal intensive care unit) for treatment.
Lakshmi et al., 2013	Neonatal death	Not defined
	Respiratory distress syndrome	Not defined
	Composite adverse perinatal outcome	Composite outcome of death or major neuro-morbidity at 12-18 months of corrected age, defined as presence of cerebral palsy or visual or hearing impairment.
Malik et al., 2013	Composite adverse perinatal outcome	Abnormal foetal outcome (IUGR, IUFD and perinatal mortality)
Masihi et al.2019	Intrapartum foetal distress	Emergency caesarean section for foetal distress
Mullick et al., 1993	FGR	Not defined
Nagar et al., 2015	FGR	Not defined
Najam et al., 2016	FGR	Not defined

	NICU admission	Not defined
	Foetal distress	Not defined
	Stillbirth	Not defined
	Neonatal death	Not defined
	Low APGAR score	Not defined
	Hypoxic ischemic encephalopathy	Not defined
	Meconium aspiration syndrome	Not defined.
Nouh et al., 2011	Composite adverse perinatal outcome	The presence of one or more of the following; miscarriage, gestational DM, PIH, PE, antepartum haemorrhage, intrauterine growth retardation, instrumental, caesarean delivery and preterm labour.
Pares et al., 2008	Foetal anaemia	Anaemia was considered moderate to severe when foetal haemoglobin concentrations were < or =0.64 multiples of the median for gestational age.
Pattinson et al., 1991	Composite adverse perinatal outcome	Poor foetal outcome (details not provided).
Pattinson et al., 1993	Composite adverse perinatal outcome	Complications of pregnancy, namely intra-uterine growth retardation and proteinuric hypertension.
Phupong et al., 2003	FGR	Birth weight less than 10 percentile for gestational age.
Rani et al., 2016	Composite adverse perinatal outcome	Adverse perinatal outcome was defined as any of these: small for gestational age, still birth, APGAR score <5 at 5 minutes, need of bag and mask ventilation for >10 minutes or hypoxic ischemic encephalopathy, admission to neonatal intensive care unit (NICU), and caesarean section due to non-reassuring foetal heart rate.
Rocca et al., 1995	IUGR	Not defined.
	Low APGAR score 5mins	APGAR score <7 at 5 minutes.
	Perinatal death	Not defined.
	Foetal distress	Emergency operative delivery for foetal distress.
Verma et al., 2016	FGR	Not defined.
	LBW	Birth weight <2500 gm.
	Preterm delivery	Spontaneous delivery <37 weeks.

For peer review only

	Composite adverse perinatal outcome	At least one adverse outcome (preeclampsia, FGR, low birth weight, spontaneous preterm delivery, oligohydramnios, foetal loss).
Waa et al., 2010	Composite adverse perinatal outcome	Poor outcome was defined by foetal mortality or appearance, pulse rate, grimace, activity, respiration (APGAR) score less than eight at five minutes or weight less than 10th percentile for gestation 20 or head circumference and length below 10th percentile for gestation.
Yelikar et al., 2013	Intrapartum foetal distress	Delivered by emergency caesarean section for suspected foetal distress.
Zarean et al., 2018	Composite adverse perinatal outcome	Adverse perinatal outcome, including preterm labour, intrauterine foetal death, PE, low 5-min APGAR score (<7), low umbilical arterial cord blood pH, admitted to Intensive Care Unit in the first 3 days of birth, low birth weight, infant with low weight, death of new-borns, caesarean section for respiratory distress, and meconial amniotic fluid.

[a]FGR: fetal growth restriction; FGR: intrauterine growth restriction; LBW: low birth weight; NICU: neonatal intensive care unit.

For peer review only

Reporting checklist for systematic review and meta-analysis.

Based on the PRISMA guidelines.

Instructions to authors

Complete this checklist by entering the page numbers from your manuscript where readers will find each of the items listed below.

Your article may not currently address all the items on the checklist. Please modify your text to include the missing information. If you are certain that an item does not apply, please write "n/a" and provide a short explanation.

Upload your completed checklist as an extra file when you submit to a journal.

In your methods section, say that you used the PRISMAreporting guidelines, and cite them as:

Moher D, Liberati A, Tetzlaff J, Altman DG, The PRISMA Group. Preferred Reporting Items for Systematic Reviews and Meta-Analyses: The PRISMA Statement

For peer review only

		Reporting Item	Page Number
**Title**			
	#1	Identify the report as a systematic review, meta-analysis, or both.	1
**Abstract**			
Structured summary	#2	Provide a structured summary including, as applicable: background; objectives; data sources; study eligibility criteria, participants, and interventions; study appraisal and synthesis methods; results; limitations; conclusions and implications of key findings; systematic review registration number	2
**Introduction**			
Rationale	#3	Describe the rationale for the review in the context of what is already known.	3
Objectives	#4	Provide an explicit statement of questions being addressed with reference to participants, interventions, comparisons, outcomes, and study design	2

		(PICOS).	
**Methods**			
Protocol and registration	#5	Indicate if a review protocol exists, if and where it can be accessed (e.g., Web address) and, if available, provide registration information including the registration number.	4
Eligibility criteria	#6	Specify study characteristics (e.g., PICOS, length of follow-up) and report characteristics (e.g., years considered, language, publication status) used as criteria for eligibility, giving rational	4
Information sources	#7	Describe all information sources in the search (e.g., databases with dates of coverage, contact with study authors to identify additional studies) and date last searched.	4
Search	#8	Present full electronic search strategy for at least one database, including any limits used, such that it could be repeated.	4
Study selection	#9	State the process for selecting studies (i.e., for screening, for determining eligibility, for inclusion in the systematic review, and, if applicable, for inclusion in the meta-analysis).	5
Data collection process	#10	Describe the method of data extraction from reports (e.g., piloted forms, independently by two reviewers) and any processes for obtaining and confirming data from investigators.	5
Data items	#11	List and define all variables for which data were sought (e.g., PICOS, funding sources), and any assumptions and simplifications made.	4
Risk of bias in individual studies	#12	Describe methods used for assessing risk of bias in individual studies (including specification of whether this was done at the study or outcome level, or both), and how this information is to be used in any data synthesis.	5
Summary measures	#13	State the principal summary measures (e.g., risk ratio, difference in means).	5
Planned methods of analyis	#14	Describe the methods of handling data and combining results of studies, if done, including measures of consistency (e.g., I2) for each meta-analysis.	5
Risk of bias across studies	#15	Specify any assessment of risk of bias that may affect the cumulative evidence (e.g., publication bias, selective reporting within studies).	5
Additional	#16	Describe methods of additional analyses (e.g., sensitivity or subgroup	na

For peer review only

analyses		analyses, meta-regression), if done, indicating which were pre-specified.	
**Results**			
Study selection	#17	Give numbers of studies screened, assessed for eligibility, and included in the review, with reasons for exclusions at each stage, ideally with a flow diagram.	6
Study characteristics	#18	For each study, present characteristics for which data were extracted (e.g., study size, PICOS, follow-up period) and provide the citation.	6
Risk of bias within studies	#19	Present data on risk of bias of each study and, if available, any outcome-level assessment (see Item 12).	5
Results of individual studies	#20	For all outcomes considered (benefits and harms), present, for each study: (a) simple summary data for each intervention group and (b) effect estimates and confidence intervals, ideally with a forest plot.	6-8
Synthesis of results	#21	Present the main results of the review. If meta-analyses are done, include for each, confidence intervals and measures of consistency.	6-8
Risk of bias across studies	#22	Present results of any assessment of risk of bias across studies (see Item 15).	6
Additional analysis	#23	Give results of additional analyses, if done (e.g., sensitivity or subgroup analyses, meta-regression [see Item 16]).	na
**Discussion**			
Summary of Evidence	#24	Summarize the main findings, including the strength of evidence for each main outcome; consider their relevance to key groups (e.g., health care providers, users, and policy makers	8
Limitations	#25	Discuss limitations at study and outcome level (e.g., risk of bias), and at review level (e.g., incomplete retrieval of identified research, reporting bias).	8
Conclusions	#26	Provide a general interpretation of the results in the context of other evidence, and implications for future research.	10
**Funding**			
Funding	#27	Describe sources of funding or other support (e.g., supply of data) for the systematic review; role of funders for the systematic review.	11

For peer review only

The PRISMA checklist is distributed under the terms of the Creative Commons Attribution License CC-BY.
This checklist was completed on 02. February 2021 using https://www.goodreports.org/, a tool made by the EQUATOR Network in collaboration with Penelope.ai

For peer review only

BMJ Open

Prognostic accuracy of antenatal Doppler ultrasound for adverse perinatal outcomes in low- and middle-income countries: a systematic review

Journal:	*BMJ Open*
Manuscript ID	bmjopen-2021-049799.R1
Article Type:	Original research
Date Submitted by the Author:	29-Sep-2021
Complete List of Authors:	ALI, SAM; Utrecht University, Julius Global Health, Julius Center for Health Sciences and Primary Care, University Medical Center Utrecht; Ernest Cook Ultrasound Research and Education Institute<br>Heuving, Simelina; Utrecht University, Julius Global Health, Julius Center for Health Sciences and Primary Care, University Medical Center Utrecht<br>Kawooya, Michael; Ernest Cook Ultrasound Research and Education Institute<br>Byamugisha , Josaphat; Makerere University, Department of Obstetrics and Gynecology<br>Grobbee, Diederick; Utrecht University, Julius Global Health, Julius Center for Health Sciences and Primary Care, University Medical Center Utrecht<br>Papageorghiou, Aris; University of Oxford, Nuffield Department of Women's & Reproductive Health<br>Klipstein-Grobusch, Kerstin; Utrecht University, Julius Global Health, Julius Center for Health Sciences and Primary Care, University Medical Center Utrecht; University of the Witwatersrand, Division of Epidemiology and Biostatistics, School of Public Health, Faculty of Health Sciences<br>Rijken, Marcus; Utrecht University, Julius Global Health, Julius Center for Health Sciences and Primary Care, University Medical Center Utrecht; Universitair Medisch Centrum Utrecht, Department of Obstetrics and Gynecology
**Primary Subject Heading**:	Obstetrics and gynaecology
Secondary Subject Heading:	Radiology and imaging, Reproductive medicine, Diagnostics
Keywords:	Ultrasound < RADIOLOGY & IMAGING, Prenatal diagnosis < OBSTETRICS, Ultrasonography < OBSTETRICS, Fetal medicine < OBSTETRICS

SCHOLARONE™
Manuscripts

I, the Submitting Author has the right to grant and does grant on behalf of all authors of the Work (as defined in the below author licence), an exclusive licence and/or a non-exclusive licence for contributions from authors who are: i) UK Crown employees; ii) where BMJ has agreed a CC-BY licence shall apply, and/or iii) in accordance with the terms applicable for US Federal Government officers or employees acting as part of their official duties; on a worldwide, perpetual, irrevocable, royalty-free basis to BMJ Publishing Group Ltd ("BMJ") its licensees and where the relevant Journal is co-owned by BMJ to the co-owners of the Journal, to publish the Work in this journal and any other BMJ products and to exploit all rights, as set out in our licence.

The Submitting Author accepts and understands that any supply made under these terms is made by BMJ to the Submitting Author unless you are acting as an employee on behalf of your employer or a postgraduate student of an affiliated institution which is paying any applicable article publishing charge ("APC") for Open Access articles. Where the Submitting Author wishes to make the Work available on an Open Access basis (and intends to pay the relevant APC), the terms of reuse of such Open Access shall be governed by a Creative Commons licence – details of these licences and which Creative Commons licence will apply to this Work are set out in our licence referred to above.

Other than as permitted in any relevant BMJ Author's Self Archiving Policies, I confirm this Work has not been accepted for publication elsewhere, is not being considered for publication elsewhere and does not duplicate material already published. I confirm all authors consent to publication of this Work and authorise the granting of this licence.

For peer review only

Prognostic accuracy of antenatal Doppler ultrasound for adverse perinatal outcomes in low- and middle-income countries: a systematic review

Sam Ali[1,2*], Simelina Heuving[1], Michael G. Kawooya[2], Josaphat Byamugisha[3], Diederick E. Grobbee[1], Aris T. Papageorghiou[4], Kerstin Klipstein-Grobusch[1,5], Marcus J. Rijken[1,6]

[1]Julius Global Health, Julius Center for Health Sciences and Primary Care, University Medical Center Utrecht, Utrecht University, Utrecht, The Netherlands.

[2]Ernest Cook Ultrasound Research and Education Institute (ECUREI), Mengo Hospital, Kampala, Uganda.

[3]Department of Obstetrics and Gynecology, Makerere University College of Health Sciences, Kampala, Uganda.

[4]Nuffield Department of Women's and Reproductive Health, John Radcliffe Hospital, University of Oxford, Oxford, United Kingdom.

[5]Division of Epidemiology and Biostatistics, School of Public Health, Faculty of Health Sciences, University of the Witwatersrand, Johannesburg, South Africa.

[6]Department of Obstetrics and Gynecology, University Medical Center Utrecht, Utrecht University, Utrecht, Netherlands.

Corresponding author:

Sam Ali

For peer review only

Department of research, Ernest Cook Ultrasound Research and Education Institute (ECUREI), Mengo Hospital. Sir Albert Cook Road. P.O. Box 7161, Kampala, Uganda.

Email: S.Ali-2@umcutrecht.nl or alisambecker@gmail.com

Word count: 2834

ABSTRACT

Objectives This systematic review examined available literature on the prognostic accuracy of Doppler ultrasound for adverse perinatal outcomes in LMIC.

Design We searched PubMed, Embase, Cochrane Library and Scopus from inception to April 2020.

Setting Observational or interventional studies from low- and middle-income countries

Participants Singleton pregnancies of any risk profile.

Interventions Umbilical artery (UA), middle cerebral artery (MCA), cerebroplacental ratio (CPR), uterine artery (UtA), fetal descending aorta (FDA), ductus venosus, umbilical vein, and inferior vena cava.

Primary and secondary outcome measures. Perinatal death, stillbirth, neonatal death, expedited delivery for fetal distress, meconium-stained amniotic fluid, low birth weight, fetal growth restriction (FGR), admission to neonatal intensive care unit, neonatal acidosis, Apgar scores, preterm birth, fetal anemia, respiratory distress syndrome, length of hospital stay, birth asphyxia and composite adverse perinatal outcomes.

For peer review only

Results We identified 2825 records, and 30 (including 4977 women) from Africa (40.0%, n= 12), Asia (56.7%, n= 17) and South America (3.3%, n= 01) were included. Many individual studies reported associations and promising predictive values of UA Doppler for various adverse perinatal outcomes mostly in high-risk pregnancies, and moderate to high predictive values of MCA, CPR and UtA Dopplers for composite adverse perinatal outcomes. A few studies suggested that the MCA and FDA may be potent predictors of fetal anemia. No randomized clinical trial was found. Most studies were of sub-optimal quality, poorly powered and characterized by wide variations in outcome classifications, the timing for the Doppler tests and study populations.

Conclusion Local evidence to guide how antenatal Doppler ultrasound should be used in LMIC is lacking. Well-designed studies, preferably randomized clinical trials, are required. Standardization of practice and classification of perinatal outcomes across countries, following the international standards, is imperative.

Keywords Pregnancy, ultrasound, prenatal diagnosis, prenatal care, developing countries, and systematic review.

Strengths and limitations of this study

- This systematic review used the most optimal database combinations and snowballing technique with no time restrictions to identify the records.
- We comprehensively examined available literature on the prognostic accuracy of Doppler ultrasound for adverse pregnancy outcomes in low and middle-income countries.

For peer review only

- Although only English language articles were included, it is unlikely that high impact papers were not identified.
- Pooling and interpreting the data for wider clinical application was not possible due to the large heterogeneity across studies.

INTRODUCTION

Stillbirths remain a major global challenge,[1] with nearly three million cases reported annually.[2] The vast majority of the cases (98%) are contributed by low- and middle-income countries (LMIC).[3] These deaths have profound effects on the families and communities involved, and strategies for reduction are of high societal importance. The risk of adverse perinatal outcomes is higher in compromised fetuses than in normally growing babies, and could be distinguishable using antenatal Doppler ultrasound.[4,5] Prenatal diagnosis of fetuses at risk provides a window for close monitoring and/or expedited delivery of well-developed babies with the prospect of improving survival and long-term wellbeing.[4]

The predictive performance of Doppler ultrasound for adverse perinatal outcomes has been demonstrated in primary studies, systematic reviews and meta-analysis from high-income countries (HIC), guiding the development of HIC practice guidelines.[6] The use of HIC guidelines for clinical guidance in LMIC without local validation may be inappropriate given the differences in the prevalence of adverse pregnancy outcomes in the two settings. For instance, the stillbirth rates per 1000 total births (95% confidence interval) is 3.4 (3.4-3.5) in HIC, 25.5 (22.5-29.1) in Southern

For peer review only - http://bmjopen.bmj.com/site/about/guidelines.xhtml

Asia and 28.7 (25.1-34.2) in sub-Saharan Africa.[2] Since the prevalence and severity of a disease influences the diagnostic or prognostic test performance, context-specific guidance is necessary.[7] However, there are still knowledge gaps about the predictive ability of antenatal Doppler for adverse pregnancy outcomes in LMIC.

This systematic review examined existing literature on the prognostic accuracy of Doppler ultrasound for adverse perinatal outcomes in LMIC. The implications for clinical utility of the available local evidence to guide practice in LMIC are highlighted.

MATERIAL AND METHODS

Protocol and registration

This systematic review protocol was registered in the PROSPERO database: CRD42019128546, and reported following the Preferred Reporting Items for a Systematic Review and Meta-analysis of Diagnostic Test Accuracy Studies: The PRISMA-DTA Statement.[8]

Eligibility criteria

We included observational (cohort or case-control) studies and randomized clinical trials (RCTs) from LMIC (as per the World Bank country classifications in the year 2020) reporting the prognostic value of Doppler ultrasound for adverse perinatal outcomes in singleton pregnancies of any risk profile. Doppler measurements of interest included umbilical artery (UA), middle cerebral artery (MCA), cerebroplacental ratio (CPR), uterine artery (UtA), fetal descending aorta (FDA), ductus venosus (DV), umbilical vein (UV) and inferior vena cava (IVC). Adverse perinatal outcomes (as defined in the included studies) were perinatal death, stillbirth, neonatal death,

For peer review only

expedited delivery for fetal distress, meconium stained amniotic fluid, low birth weight, fetal growth restriction (FGR), admission to neonatal intensive care unit (NICU), neonatal acidosis, Apgar scores, preterm birth, fetal anemia, respiratory distress syndrome (RDS), length of hospital stay, birth asphyxia, and composite adverse perinatal outcomes (CAPO). Conference proceedings/posters that did not appear as full-text papers, case reports and review articles without original data were excluded.

For peer review only

Information sources and search

We conducted a comprehensive literature search in PubMed (Medline), Embase, Cochrane Library and Scopus for articles published from inception to April 07, 2020. The search strategies (online supplementary appendix S1) were developed with the support of a librarian at University Medical Center Utrecht. When applicable, pre-defined search (Title/Abstract) and MeSH/Emtree terms were used. No limits were applied to the searches.

Study selection

The records retrieved from the databases were exported to Endnote to eliminate duplicates and then transferred to Rayyan for review and selection. Two reviewers (SA and SH) independently assessed all studies for inclusion based on title and abstract. Studies reporting any Doppler parameter and adverse pregnancy outcome of interest in the title or abstract were further retrieved in full text and assessed by the same two reviewers against full eligibility criteria. Disagreements were resolved by discussion or, if required, we consulted the third review author (MJR).

Data extraction

For peer review only - http://bmjopen.bmj.com/site/about/guidelines.xhtml

Using a pre-piloted data extraction sheet, two reviewers (SA and SH) independently extracted data on authors, study title, year of publication, aims of the study, study period, the number of women recruited, gestational age at Doppler ultrasound exam, method of pregnancy dating, pregnancy risk profile, blood vessels studied, pregnancy outcomes (as defined in the primary study), and key results. If any relevant information was missing, the corresponding authors were contacted once by e-mail.

Risk of bias assessment

Two raters (SA and SH) independently evaluated the risk of bias for each study using the quality in prognostic studies (QUIPS) tool.[9] The risk of bias domains included study population, attrition, prognostic factor measurement, outcome measurement, confounding and statistical analysis. All the domains were separately judged by two raters as having a low, moderate or high risk of bias. Any disagreement during this process was resolved by contacting the third rater (MJR).

Prognostic test accuracy measures

Doppler test prognostic performance measures, as reported in the selected studies, are presented in table S1. These included diagnostic test accuracy measures such as sensitivity, specificity, positive predictive values (PPV) and negative predictive values (NPV); measures of association; proportions; and correlations.

Data synthesis and analysis

The results were narratively summarized. The large heterogeneity in the study populations, timing for Doppler tests, outcome definitions and prognostic performance

For peer review only

measures in the included studies did not allow for a meta-analysis. If a study reported multiple Doppler indices, the most commonly used (pulsatility index) was selected.

Patient and public involvement

No patient was involved. The public was also not involved in the design, conduct and dissemination of this research.

RESULTS

Study selection

The 2825 records we identified through electronic searches were reduced to 2210 after the removal of duplicates, and 2162 were further excluded based on title and abstract screening, retaining 48 records. After full-text assessment for eligibility, 23 studies were excluded with reasons, and 25 remained (online supplementary appendix S2). Five additional records were identified through snowballing (Figure 1). Thirty studies, involving a total count of 4977 women and a median (interquartile range) sample size of 100 (30 to 181) were included in the analysis (table 1).

Study characteristics

The selected studies were from Africa (40.0%, n = 12), Asia 17 (56.7%, n = 17) and South America (3.3%, n = 01). Twenty studies (67%) recruited high-risk pregnancies, six (16.7%) both high and low-risk populations, while five (16.7%) studied the low-risk group (online supplementary appendix S3). Thirteen (43.3%) studies did not specify a method of pregnancy dating, 13 (43.3%) assessed gestational age using last menstrual period (LMP) combined with ultrasound, three (10.0%) used ultrasound alone, and one

For peer review only

(3.3%) study used LMP. No RCTs was identified, and no study provided data on the UV and IVC Dopplers (table 1). The reasons for undertaking the Doppler research varied by individual studies and included the prediction of the risk of FGR, fetal anemia, neonatal acidosis, among others (online supplementary appendix S3).

Methodological quality of included studies

The results of the QUIPS assessment are provided in Figure 2 and online supplementary appendix S4. Overall, the risk of bias was low in 15 (50%), moderate in 10 (33.3%), and high in five (16.7%) studies. In the study population domain, the risk of bias was low in 73.3%, moderate in 23.3%, and high in 3.3% of the studies. Selective reporting remarkably resulted in a moderate to high risk of bias for analysis and reporting in 20 (66.7%) studies. We found a moderate to high risk of bias for outcome measurement in 17 (56.7%) studies, mostly due to inconsistencies in outcome classifications (online supplementary table S2).

Prognostic accuracy of antenatal Doppler ultrasound for adverse perinatal outcomes

Twenty studies evaluated the umbilical artery,[10–29] and seven reported its predictive values for FGR. The positive predictive values for FGR reported in the individual studies were between 77.40 and 88.5,[11,16,21,24] while the area under the receiver operating characteristic (AU ROC) curve was 0.63,[17] mostly in high-risk pregnancies. The NPV ranged from 55.4 - 95.65.[11,16,21,24] FGR was defined as birth weight or abdominal circumference below the 10th percentile in two studies,[11,17] ponderal index less than 10 in one study,[21] and was not defined in the remaining studies.[16,24,26] Increased flow impedance in the UA had positive predictive values for

For peer review only - http://bmjopen.bmj.com/site/about/guidelines.xhtml

composite adverse outcomes between 66.60 and 96.6 in high-risk pregnancies.[11,13,19,23] All studies provided individual components of the CAPO except only one.[11] Absent or reversed end-diastolic flow (AREDF) in the UA was associated with poor pregnancy outcomes (perinatal death, odds ratio (OR) 9.8, 95% confidence interval (CI) 2.1 to 46.4; CAPO: OR 2.4, 95% CI 1.1 to 5.0; and RDS: OR 8.4, 95% CI 2.3 to 30.5).[14,22,26]

The MCA was reported in 12 studies.[11,12,13,15,19,21,23,26,28,30,31,32] The positive predictive values for fetal anemia in Rhesus (Rh) isoimmunized pregnancies requiring transfusion were between 83.0 - 90.9 and the AU ROC curve was 0.7.[12,32] Fetal anemia was consistently defined as hemoglobin (Hb)=< 0.64 g/dl in the two studies, though they recruited low numbers of women.[12,32] MCA Doppler had a sensitivity of 87.5%, PPV of 74.0% and AU ROC curve of 0.82 for neonatal acidosis.[30] The positive predictive values for CAPO ranged from 80.0-100% in high-risk pregnancies,[11,13,19,23,31] but two studies did not provide details of the individual components of the CAPO.[11,31]

Nine studies reported the prognostic value of CPR.[11,13,15,19,20,23,26,33,34] CPR showed promising predictive value for adverse perinatal outcomes in unselected pregnancies in the third trimester. One study reported sensitivity 85.10, specificity 89.72, PPV 80.70 and NPV 92.30 for FGR.[26] Two studies found sensitivity between 80.90 and 90.91%, and specificity between 50.0 and 78.04% for emergency caesarean section for fetal distress though the tests had poor positive predictive values.[26,34] Abnormal CPR had positive predictive values for CAPO between 81.80 and 100% in high-risk pregnancies.[11,13,15,23]

For peer review only

For peer review only - http://bmjopen.bmj.com/site/about/guidelines.xhtml

Eight studies reported the prognostic value of UtA Doppler,[14,23,25,35–39] and two showed positive predictive values of over 91.8% for CAPO in high-risk pregnancies.[23,36] The remaining studies had poor predictive values for adverse perinatal outcomes.

Three studies evaluated the prognostic accuracy of FDA Doppler.[12,13,32] The FDA sensitivity for fetal anemia in Rh isoimmunized pregnancies ranged from 87.0% to 95.7% when used in isolation.[12,32] The sensitivity varied between 86.0% and 98.4% and positive predictive values ranged from 86.0- 100% when combined with the MCA.[12,32]

The DV was sampled in two studies undertaken in high-risk pregnancies.[20,30] Abnormal DV had a sensitivity of 100, PPV of 72.0 and AU ROC curve of 0.88 for the prediction of neonatal acidosis, though this study included only 30 women between 36-41 weeks of gestation.[30] The second study found a borderline significance and positive predictive value of 92.0% for the prediction of composite adverse perinatal outcomes at 24-34 weeks of gestation.[20]

DISCUSSION

Summary of findings

Many individual studies showed that abnormal UA Doppler was associated with poor perinatal outcomes, mostly in high-risk pregnancies, and that abnormal UA, MCA, CPR and UtA Dopplers had moderate to high predictive values for composite adverse perinatal outcomes. A few studies suggested that abnormal MCA Doppler had high individual predictive value for fetal anemia, but performed better when combined with the FDA. However, the majority of the available evidence was of sub-optimal quality,

For peer review only

For peer review only - http://bmjopen.bmj.com/site/about/guidelines.xhtml

based on a few poorly powered studies and had no RCTs. Further, wide variations in the populations studied, definitions of adverse perinatal outcomes and prognostic accuracy measures across studies was present. Thus, pooling and interpreting the evidence for wider clinical application was not possible.

Implications for practice

Evidence from HIC suggests that adding Doppler studies into clinical diagnostic or prognostic rules improves pregnancy risk assessment,[6] and are increasingly becoming integrated into their pregnancy management guidelines.[4,6] The use of guidance based entirely on HIC data in daily practice in LMIC could be inappropriate considering the differences in the adverse pregnancy outcome rates in the two settings. The stillbirth rates in LMIC is approximately 10 times that of HIC,[2] a large variation likely to influence the predictive performance of diagnostic or prognostic tests.[7] Thus, a proper understanding of existing literature from LMIC is important. This paper reports the findings of a systematic review of primary evidence on the prognostic value of antenatal Doppler ultrasound for adverse perinatal outcomes in LMIC.

Abnormal blood flow patterns in the UA had moderate to high predictive values for FGR and was associated with poor outcomes in high-risk pregnancies. Similarly, a recent Cochrane review of RCTs from HIC suggests that using UA Doppler in high-risk pregnancies could reduce perinatal deaths by 30% (risk ratio 0.71, 95% CI 0.52 to 0.98), and lead to fewer obstetric interventions.[40] Despite some similarities with our findings, the definitions of adverse outcomes, including FGR were inconsistent (or not even defined in many studies included in this review) with recommended international

For peer review only - http://bmjopen.bmj.com/site/about/guidelines.xhtml

standards,[4,41] and with no clear distinction between early and late FGR. Scanty data from this review indicate that abnormal CPR, UA, MCA and UtA Doppler could be predictive of CAPO. However, in a previous systematic review from HIC, CPR had low predictive accuracy (pooled sensitivity: 57%, specificity: 77%, and summary positive likelihood ratio (LR): 2.5, and negative LR: 0.60) for CAPO in pregnancies with suspected FGR antenatally.[42] In another review, CPR was significantly better than UA and MCA Doppler in predicting CAPO ($P < 0.001$) and emergency delivery for fetal distress in singleton pregnancies of all risk profiles,[43] but the primary studies reviewed had numerous methodological limitations.[43] Further, first-trimester UtA Doppler had very low sensitivity 25.8% (95% CI 15.5 to 39.7) for CAPO in a systematic review of 18 studies (involving 55974 women).[44] More data from HIC indicate that MCA-PSV reliably predicts fetal anemia in un-transfused fetuses.[45] The area under the hierarchical summary ROC curve for moderate-severe anemia in untransfused fetuses was 87%, pooled sensitivity 86% (95% CI 75 to 93%) and specificity 71% (95% CI 49 to 87%).[45] Similarly, in our study, MCA alone or when combined with FDA had high predictive values for fetal anemia in Rh isoimmunized pregnancies, but this was based on only three studies. Overall, this review found that high-quality studies on the predictive accuracy of Doppler ultrasound for adverse perinatal outcomes in LMIC were scarce. The large heterogeneity across studies precluded a meta-analysis and between-study comparisons.

Implications for research

For peer review only

Future studies need to specify the methods and timing for pregnancy dating. Accurate dating is crucial for timing the Doppler tests and interventions to expedite delivery in compromised fetuses. The interpretation and comparison of Doppler studies could be improved by using standard outcome definitions and completeness in reporting.[46] Most primary studies in this review studied the predictive ability of a single variable (Doppler test) for the outcome(s) of interest, without considering existing characteristics of clinical importance to estimate pregnancy risk. The predictive accuracies of new determinants need to be assessed individually and by multivariable analysis to facilitate the clinical applicability of the findings. The clinical applicability of Doppler ultrasound also depends on the clinical judgement of the Doppler measurements and the feasibilities of local healthcare systems to interpret and respond to the results of the Doppler scan. Along the same line, our recently concluded prospective cohort study in a rural sub-Saharan African setting will soon highlight the prognostic value of Doppler ultrasound in the late third trimester and the feasibilities of integrating such advanced technologies into routine antenatal care in LMIC.

Strengths and limitations

A strength of this systematic review is that it was conducted according to a registered protocol, using the most optimal database combinations and snowballing with no time restrictions. However, it is possible that some studies performed in low-resource settings, may not have been indexed in the searched databases. Although we only included English language articles, it is unlikely that high impact papers were not identified. Further, this review primarily aimed to thoroughly examine the current

For peer review only

evidence on the predictive value of Doppler ultrasound for adverse perinatal outcomes in LMIC using a meta-analysis. However, due to the inherent limitations in the included studies such as large heterogeneity in the study populations, inconsistencies in the definition of pregnancy outcomes, differences in the gestational age at the Doppler study and prognostic accuracy measures reported, we were only able to present our findings narratively. A future updated systematic review and meta-analysis of high-quality evidence is recommended.

CONCLUSION

This review demonstrated that a scientific basis to provide evidence for how antenatal Doppler should be used in LMIC is lacking. Well-designed studies, preferably randomized clinical trials, testing application models of antenatal Doppler while respecting the local conditions are needed. Moreover, local practice and classification of perinatal outcomes need to be standardized, utilizing approaches consistent with international consensus.

Acknowledgements

We thank the University Medical Center Utrecht librarians Paulien Wiersma and Felix. We thank the team from Makerere University College of Health Sciences and ECUREI that supported us to undertake this review. We thank Fosca Tumushabe for the English language review.

Author contributions

For peer review only

SA, SH, KKG, and MJR drafted the protocol and conducted the review. MGK, JB, DEG, and ATP critically reviewed the work for important intellectual content. All the authors approved the final manuscript.

Funding

This research was supported by the UMC Utrecht Global Health Support Program (Ref: FM/ADB/D-18-015006), University Medical Center Utrecht, Utrecht, The Netherlands, and Grand Challenges Canada (Grant Number: R-ST-POC-1808-17038).

For peer review only

Competing interests

None

Data sharing statement

No additional data are available.

Ethics statements

Patient consent for publication

Not required.

Ethics approval

Given this is a systematic review, ethics approval is not required.

REFERENCES

1. de Bernis L, Kinney M V, Stones W, ten Hoope-Bender P, Vivio D, Leisher SH, et al. Stillbirths: ending preventable deaths by 2030. Lancet. 2016;387(10019):703–16.
2. Lawn JE, Blencowe H, Waiswa P, Amouzou A, Mathers C, Hogan D, et al. Stillbirths: Rates, risk factors, and acceleration towards 2030. Lancet. 2016;387(10018):587–603.

3. Reinebrant HE, Leisher SH, Coory M, Henry S, Wojcieszek AM, Gardener G, et al. Making stillbirths visible: a systematic review of globally reported causes of stillbirth. BJOG An Int J Obstet Gynaecol. 2018 Jan 1;125(2):212–24.

4. Lees CC, Stampalija T, Baschat A, da Silva Costa F, Ferrazzi E, Figueras F, et al. ISUOG Practice Guidelines: diagnosis and management of small-for-gestational-age fetus and fetal growth restriction. Ultrasound Obstet Gynecol. 2020;56(2):298–312.

5. Figueras F, Caradeux J, Crispi F, Eixarch E, Peguero A, Gratacos E. Diagnosis and surveillance of late-onset fetal growth restriction. Am J Obstet Gynecol. 2018 Feb 1;218(2):S790-S802.e1.

6. McCowan LM, Figueras F, Anderson NH. Evidence-based national guidelines for the management of suspected fetal growth restriction: comparison, consensus, and controversy. Am J Obstet Gynecol. 2018 Feb 1;218(2):S855–68.

7. Grobbee DE, Hoes AW. Clinical Epidemiology: Principles, Methods, and Applications for Clinical Research. Jones & Bartlett Publishers; 2014. 472 p.

8. McInnes MDF, Moher D, Thombs BD, McGrath TA, Bossuyt PM, and the P-DTAG. Preferred Reporting Items for a Systematic Review and Meta-analysis of Diagnostic Test Accuracy Studies: The PRISMA-DTA Statement. JAMA. 2018;319(4):388–96.

9. Hayden JA, van der Windt DA, Cartwright JL, Côté P, Bombardier C. Assessing Bias in Studies of Prognostic Factors. Ann Intern Med. 2013;158(4):280–6.

10. Abdallah A, Eldorf A, Sallam S, Ahmed S, Shawky M, Nawara M, et al. Nuchal cord: impact of umbilical artery Doppler indices on intrapartum and neonatal outcomes: a prospective cohort study. J Matern Neonatal Med. 2019;32(20):3367–78.

11. Bano S, Chaudhary V, Pande S, Mehta V, Sharma A. Color doppler evaluation of cerebral-umbilical pulsatility ratio and its usefulness in the diagnosis of intrauterine growth retardation and prediction of adverse perinatal outcome. Indian J Radiol Imaging. 2010;20(1):20–5.

12. Kumari S, Deka D, Dadhwal V, Perumal V. Correlation of fetal blood vessel Doppler measurements with fetal anemia among Rhesus isoimmunized pregnancies after two intrauterine transfusions. Int J Gynecol Obstet. 2019;146(2):218–22.

For peer review only

13. Lakhkar BN, Rajagopal K V, Gourisankar PT. Doppler prediction of adverse perinatal outcome in PIH and IUGR. Indian J Radiol Imaging. 2006;16(1):109.

14. Pattinson RC, Brink AL, De Wet PE, Odendaal HJ. Early detection of poor fetal prognosis by serial Doppler velocimetry in high-risk pregnancies. South African Med J. 1991/11/02. 1991;80(9):428–31.

15. Rani S, Huria A, Kaur R. Prediction of perinatal outcome in preeclampsia using middle cerebral artery and umbilical artery pulsatility and resistance indices. Hypertens Pregnancy. 2016;35(2):210–6.

16. Rocca MM, Said MS, Khamis MY, Ghanem IA, Karkour TA. The Value of Doppler Study of the Umbilical Artery in Predicting Perinatal Outcome in Pre-Eclamptic Patients. J Obstet Gynaecol (Lahore). 1995;21(5):427–31.

17. Agbaje OA, Adeyomoye AAO, Omidiji OAT, Oboke OS, Afolabi BB. Evaluation of Umbilical Artery Doppler Indices in Pregnant Women With Sickle Cell Anemia Disease at a Nigerian Tertiary Hospital. J Diagnostic Med Sonogr. 2018;34(6):466–78.

18. Anshul D, Neelu S, Suneeta G. Significance of umbilical artery Doppler velocimetry in the perinatal outcome of the growth restricted fetuses. J Obstet Gynecol India. 2010;60(1):38–43.

19. Ebrashy A, Azmy O, Ibrahim M, Waly M, Edris A. Middle cerebral/umbilical artery resistance index ratio as sensitive parameter for fetal well-being and neonatal outcome in patients with preeclampsia: case-control study. Croat Med J. 2005;46(5).

20. Geerts L, Odendaal HJ. Severe early onset pre-eclampsia: prognostic value of ultrasound and Doppler assessment. J Perinatol. 2007;27(6):335–42.

21. Khanduri S, Parashari UC, Bashir S, Bhadury S, Bansal A. Comparison of diagnostic efficacy of umbilical artery and middle cerebral artery waveform with color Doppler study for detection of intrauterine growth restriction. J Obstet Gynaecol India. 2013/03/12. 2013;63(4):249–55.

22. Lakshmi CVS, Pramod G, Geeta K, Subramaniam S, Rao MB, Kallapur SG, et al. Outcome of very low birth weight infants with abnormal antenatal doppler flow patterns: A prospective cohort study. Indian Pediatr. 2013;50(9):847–52.

23. Malik R, Saxena A. Role of Colour Doppler Indices in the Diagnosis of Intrauterine Growth Retardation in High-Risk Pregnancies. J Obstet Gynecol

For peer review only

India. 2013;63(1):37–44.
24. Mullick S, Kushtagi P, Swain UK, Rao K, Muralidhar BG. Doppler waveform patterns of the umbilical artery — screening test for high risk pregnancies. Int J Gynecol Obstet. 1993 Feb 1;40(2):115–8.
25. Nagar T, Sharma D, Choudhary M, Khoiwal S, Nagar RP, Pandita A. The Role of Uterine and Umbilical Arterial Doppler in High-risk Pregnancy: A Prospective Observational Study from India. Clin Med Insights Reprod Heal. 2015;9:CMRH.S24048.
26. Najam R, Gupta S, Shalini. Predictive Value of Cerebroplacental Ratio in Detection of Perinatal Outcome in High-Risk Pregnancies. J Obstet Gynecol India. 2016;66(4):244–7.
27. Pattinson RC, Norman K, Odendaal HJ. The use of Doppler velocimetry of the umbilical artery before 24 weeks' gestation to screen for high-risk pregnancies. South African Med J. 1993;83(10):734–6.
28. Waa S, Vinayak S. Comparision of doppler studies in obstetrics with foetal outcome. East Afr Med J. 2010;87(12):502–8.
29. Yelikar KA, Prabhu A, Thakre GG. Role of Fetal Doppler and Non-Stress Test in Preeclampsia and Intrauterine Growth Restriction. J Obstet Gynecol India. 2013;63(3):168–72.
30. Allam IS, Abuelghar W, Fathy H, Enin LA, Sayed M. Prediction of neonatal acidosis by ductus venosus Doppler pattern in high risk pregnancies. Middle East Fertil Soc J. 2013;18(1):47–52.
31. Dhand H, Kansal HK, Dave A. Middle cerebral artery Doppler indices better predictor for fetal outcome in IUGR. J Obstet Gynecol India. 2011;61(2):166–71.
32. Pares D, Chinen PA, Camano L, Moron AF, Torloni MR. Prediction of fetal anemia by Doppler of the middle cerebral artery and descending thoracic aorta. Arch Gynecol Obstet. 2008;278(1):27–31.
33. Alanwar A, El Nour AA, El Mandooh M, Abdelazim IA, Abbas L, Abbas AM, et al. Prognostic accuracy of cerebroplacental ratio for adverse perinatal outcomes in pregnancies complicated with severe pre-eclampsia; a prospective cohort study. Pregnancy Hypertens. 2018;14:86–9.
34. Masihi S, Nikbakht R, Barati M, Momen Gharibvand M, Jadidi A. Association Between Fetal Middle Cerebral Artery and Umbilical Artery Doppler Ratio with

For peer review only

Fetal Distress in 38–40 Weeks of Gestation. J Obstet Gynecol India. 2019;69(6):509–13.

35. Dorman EK, Shulman CE, Kingdom J, Bulmer JN, Mwendwa J, Peshu N, et al. Impaired uteroplacental blood flow in pregnancies complicated by falciparum malaria. Ultrasound Obstet Gynecol. 2002;19(2):165–70.

36. Nouh AA, Shalaby SM. The predictive value of uterine blood flow in detecting the risk of adverse pregnancy outcome in patients with polycystic ovary syndrome. Middle East Fertil Soc J. 2011;16(4):284–90.

37. Phupong V, Dejthevaporn T, Tanawattanacharoen S, Manotaya S, Tannirandorn Y, Charoenvidhya D. Predicting the risk of preeclampsia and small for gestational age infants by uterine artery Doppler in low-risk women. Arch Gynecol Obstet. 2003;268(3):158–61.

38. Verma D, Gupta S. Prediction of adverse pregnancy outcomes using uterine artery Doppler imaging at 22-24 weeks of pregnancy: A North Indian experience. Turkish J Obstet Gynecol. 2016/06/15. 2016;13(2):80–4.

39. Zarean E, Shabaninia S. The Assessment of Association between Uterine Artery Pulsatility Index at 30-34 Week's Gestation and Adverse Perinatal Outcome. Adv Biomed Res. 2018;7:111.

40. Alfirevic Z, Stampalija T, Dowswell T. Fetal and umbilical Doppler ultrasound in high-risk pregnancies. Cochrane Database Syst Rev. 2017/06/15. 2017;2017(6):CD007529.

41. Gordijn SJ, Beune IM, Thilaganathan B, Papageorghiou A, Baschat AA, Baker PN, et al. Consensus definition of fetal growth restriction: a Delphi procedure. Ultrasound Obstet Gynecol. 2016;48(3):333–9.

42. Conde-Agudelo A, Villar J, Kennedy SH, Papageorghiou AT. Predictive accuracy of cerebroplacental ratio for adverse perinatal and neurodevelopmental outcomes in suspected fetal growth restriction: systematic review and meta-analysis. Ultrasound Obstet Gynecol. 2018;52(4):430–41.

43. Vollgraff Heidweiller-Schreurs CA, De Boer MA, Heymans MW, Schoonmade LJ, Bossuyt PMM, Mol BWJ, et al. Prognostic accuracy of cerebroplacental ratio and middle cerebral artery Doppler for adverse perinatal outcome: systematic review and meta-analysis. Ultrasound Obstet Gynecol. 2018;51(3):313–22.

44. Velauthar L, Plana MN, Kalidindi M, Zamora J, Thilaganathan B, Illanes SE, et

For peer review only

al. First-trimester uterine artery Doppler and adverse pregnancy outcome: a meta-analysis involving 55 974 women. Ultrasound Obstet Gynecol. 2014 May 1;43(5):500–7.
45. Martinez-Portilla RJ, Lopez-Felix J, Hawkins-Villareal A, Villafan-Bernal JR, Paz y Miño F, Figueras F, et al. Performance of fetal middle cerebral artery peak systolic velocity for prediction of anemia in untransfused and transfused fetuses: systematic review and meta-analysis. Ultrasound Obstet Gynecol. 2019;54(6):722–31.
46. Duffy JMN, Rolph R, Gale C, Hirsch M, Khan KS, Ziebland S, et al. Core outcome sets in women's and newborn health: a systematic review. BJOG An Int J Obstet Gynaecol. 2017 Sep 1;124(10):1481–9.

LEGENDS

Online supplementary data legends

Appendix S1. Search strings for the databases used to retrieve articles.

Appendix S2. List of full-text articles excluded with reasons.

Appendix S3. The aims of the selected studies and risk profiles of the women recruited

Appendix S4. Risk of bias assessment results of the 30 studies included in the analysis.

Table S1. Statistical measures of prognostic performance of Doppler ultrasound reported in the selected studies.

Table S2. Definitions of adverse perinatal outcomes reported in the selected studies

Figures legends

Figure 1. PRIMA flow diagram

For peer review only - http://bmjopen.bmj.com/site/about/guidelines.xhtml

Figure 2. Risk of bias assessment results of the 30 included studies

Figure 2 key

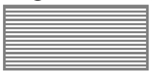

Low-risk of bias

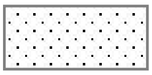

Moderate-risk of bias

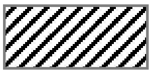

High-risk of bias

Table legends

Table 1 Summary of studies included in the systematic review of current evidence on the prognostic value of Doppler ultrasound for predicting adverse pregnancy outcomes in LMIC.

For peer review only

Table 1

Author, Year	Country	Study Period	Women	Weeks	Study Design	Vessels	Abnormal Doppler Thresholds
Abdallah, 2019.[10]	Egypt	2015-2017	92	>= 37	Cohort	UA	UA (RI, PI and S/D ratio) > 95th centile
Agbaje, 2018.[17]	Nigeria	2014-2015	120	26	Cohort	UA	S/D ratio > 95th percentile, RI > 95th percentile, and AREDF.
Alanwar, 2018.[33]	Egypt	2017	100	30 - 40	Cohort	CPR	CPR PI < 1 or CPR PI < 5th percentile.
Allam, 2013.[30]	Egypt	2007- 2010	30	36 - 41	Cohort	MCA, DV	MCA S/D ratio <4.37, DV RI > 0.29, or Decrease in a-, v- and d- waves, or reversed flow in both a- and v-waves.
Anshul, 2010.[18]	India	2005-2007	100	>= 28	Cohort	UA	S/D ratio >= 3 or AREDF.
Bano, 2010.[11]	India	Not stated	90	30 - 41	Cohort	UA, MCA, CPR	MCA < 2SD; UA > 2SD or CPR PI < 1.08
Dhand, 2011.[31]	India	2005- 2006	121	28 - 41	Cohort	MCA	Not specified
Dorman, 2002.[35]	Kenya	1996- 1997	854	24 - 31	Cohort	UtA	Early diastolic notch or mean/ipsilateral UtA RI >= 0.58
Ebrashy, 2005.[19]	Egypt	2002- 2003	80	>= 28	Case-control	UA, MCA, CPR	UA RI > 0.72, MCA RI < 0.69, CPR RI < 1.0
Geerts, 2007.[20]	South Africa	Not stated	113	24 - 34	Cohort	UA, CPR, DV	UA PI >95th centile; UA/MCA >1; DV PI > 95th centile.
Khanduri, 2013.[21]	India	2009- 2011	60	23 - 37	Cohort	UA, MCA	UA PI > 1.42 or UA RI > 0.72, MCA PI <1.5, MCA RI < 0.59

For peer review only

Kumari, 2019.[12]	India	2015-2016	30		Cohort	UA, MCA, FDA	MCA PSV > 1.50 MoM, FDA PSV delta > 70.50. Not specified for UA
Lakhkar, 2006.[13]	India	2001-2002	58	> 30	Cohort	UA, MCA, CPR, FDA	S/D ratio, RI or PI of UA > 2SD; MCA < 5th centile; FDA > 2SD; CPR PI or S/D ratio < 1.0
Lakshmi, 2013.[22]	India	2007- 2008	238	< 35	Cohort	UA	Absent and/or reversed end-diastolic flow (AREDF)
Malik, 2013.[23]	India	2010- 2011	100	31 - 41	Cohort	UA, MCA, CPR, UtA	Not specified
Masihi, 2019.[34]	Iran	2016- 2017	181	38 - 40	Cohort	CPR	CPR PI <1.94
Mullick, 1993.[24]	India	Not stated	73	22 - 26, 30 - 32, > 37	Cohort	UA	S/D ratio >= 4 (26 weeks), 3.5 (30-32 weeks) and 3 (37-40 weeks)
Nagar, 2015.[25]	India	2009 - 2011	500	26 - 30	Cohort	UA, UtA	UA (S/D ratio or RI) > 95th centile or AREDF. UtA S/D ratio > 95th centile
Najam, 2016.[26]	India	Not stated	150	28 - 40	Cohort	UA, MCA, CPR	UA S/D ratio > 2SD, or AREDF, MCA SD ratio < 5th percentile, MCA/UA SD ratio of < 1.0
Nouh, 2011.[36]	Egypt	2009-2011	80	8 - 12, 26	Case-control	UtA	UtA PI> 95th percentile, and/or Unilateral or bilateral notch
Pares, 2008.[32]	Brasil	1997- 2005	46	20 - 34	Cohort	MCA, FDA	FDA-MV >= 2SD MCA-PSV >= 1.5 MoM

For peer review only

Pattinson, 1991.[14]	South Africa	1987-1989	53	16 - 28	Cohort	UA, UtA	UA RI > 95th centile<br>UtA RI > 0.58
Pattinson, 1993.[27]	South Africa	1990	496	16 - 24	Cohort	UA	UA RI > 95th centile
Phupong, 2003.[37]	Thailand	2000- 2001	322	22 - 28	Cohort	UtA	Unilateral or bilateral early diastolic notch
Rani, 2016.[15]	India	2012-2014	223	30 - 36	Cohort	UA, MCA, CPR	UA PI > 1.03, UA RI >0.695; MCA PI < 1.2, MCA RI < 0.75; CPR PI < 1.08 or CPR RI < 1.05.
Rocca, 1995.[16]	Egypt	Not stated	113	>= 28	Cohort	UA	UA S/D ratio >= 3
Verma, 2016.[38]	India	Not stated	165	22 - 24	Cohort	UtA	Bilateral diastolic notches or mean UtA PI > 1.45 (UtA PI > 95th centile).
Waa, 2010.[28]	Kenya	2007	100	>= 28	Cohort	MCA, UA	MCA RI < 0.71, and UA > 0.71.
Yelikar, 2013.[29]	India	Not stated	189	> 32	Cohort	UA	UA S/D ratio > 90th centile or AREDF
Zarean, 2018.[39]	Iran	2015- 2016	100	30 - 34	Cohort	UtA	UtA PI > 95th centile

[a]LMP: last menstrual period; UA: umbilical artery; MCA: middle cerebral artery; CPR: cerebroplacental ratio; UtA: uterine artery; FDA: fetal descending aorta; DV: ductus venosus; RI: resistive index; PI: pulsatility index; S/D ratio: systolic diastolic ratio; PSV: peak systolic velocity; MV: mean velocity; AREDF: absent and/or reversed end diastolic flow.

For peer review only

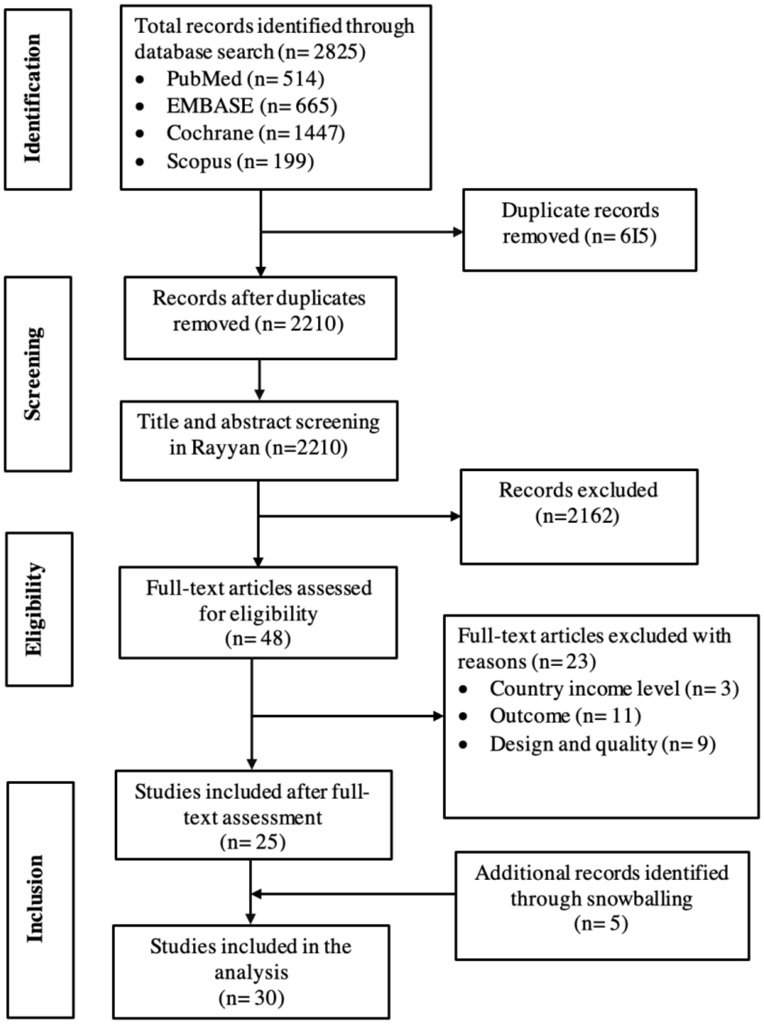

PRISMA flow diagram

273x283mm (144 x 144 DPI)

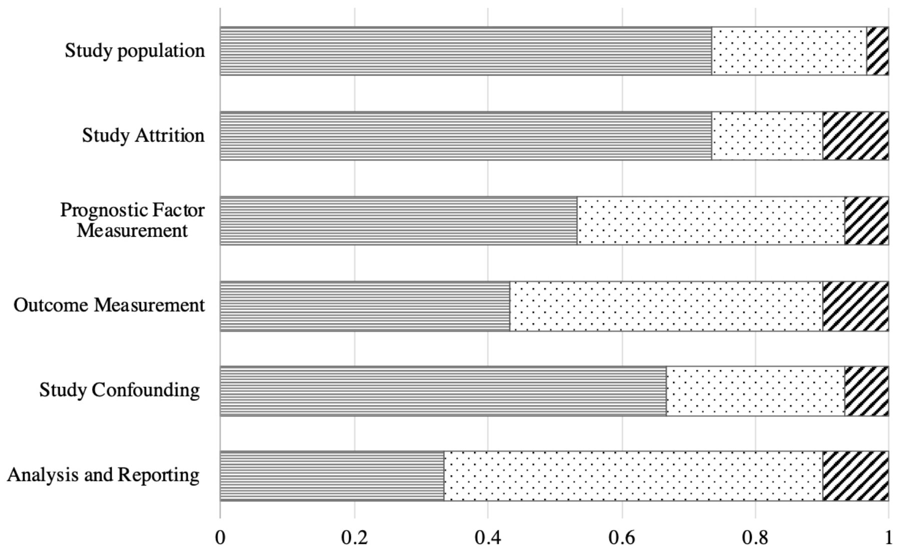

Risk of bias assessment results of the 30 included studies

482x350mm (72 x 72 DPI)

Appendix S1. Search strings for the databases used to retrieve articles

EMBASE

(‘developing countr*’:ti,ab,kw OR ‘developing nation*’:ti,ab,kw OR ‘developing population*’:ti,ab,kw OR ‘developing econom*’:ti,ab,kw OR ‘undeveloped countr*’:ti,ab,kw OR ‘undeveloped nation*’:ti,ab,kw OR ‘undeveloped economy’:ti,ab,kw OR ‘undeveloped economies’:ti,ab,kw OR ‘least developed countr*’:ti,ab,kw OR ‘least developed nation*’:ti,ab,kw OR ‘least developed economy’:ti,ab,kw OR ‘least developed economies’:ti,ab,kw OR ‘less-developed countr*’:ti,ab,kw OR ‘less-developed nation*’:ti,ab,kw OR ‘less-developed population’:ti,ab,kw OR ‘less-developed populations’:ti,ab,kw OR ‘less-developed econom*’:ti,ab,kw OR ‘lesser developed countr*’:ti,ab,kw OR ‘lesser developed nation*’:ti,ab,kw OR ‘lesser developed population’:ti,ab,kw OR ‘lesser developed populations’:ti,ab,kw OR ‘lesser developed economy’:ti,ab,kw OR ‘lesser developed economies’:ti,ab,kw OR ‘under-developed countr*’:ti,ab,kw OR ‘under-developed nation*’:ti,ab,kw OR ‘underdeveloped countr*’:ti,ab,kw OR ‘underdeveloped nation*’:ti,ab,kw OR ‘underdeveloped population*’:ti,ab,kw OR ‘underdeveloped econom*’:ti,ab,kw OR ‘low income countr*’:ti,ab,kw OR ‘middle income countr*’:ti,ab,kw OR ‘low income nation*’:ti,ab,kw OR ‘middle income nation*’:ti,ab,kw OR ‘low income population*’:ti,ab,kw OR ‘middle income population*’:ti,ab,kw OR ‘low income econom*’:ti,ab,kw OR ‘middle income econom*’:ti,ab,kw OR ‘lower income countr*’:ti,ab,kw OR ‘lower income nation*’:ti,ab,kw OR ‘lower income population*’:ti,ab,kw OR ‘lower income economy’:ti,ab,kw OR ‘lower income economies’:ti,ab,kw OR ‘resource limited’:ti,ab,kw OR ‘low resource countr*’:ti,ab,kw OR ‘lower resource countr*’:ti,ab,kw OR ‘low resource nation*’:ti,ab,kw OR ‘low resource population*’:ti,ab,kw OR ‘low resource economy’:ti,ab,kw OR ‘low resource economies’:ti,ab,kw OR ‘underserved countr*’:ti,ab,kw OR ‘underserved nation*’:ti,ab,kw OR ‘underserved population*’:ti,ab,kw OR ‘underserved economy’:ti,ab,kw OR ‘underserved economies’:ti,ab,kw OR ‘under-served country’:ti,ab,kw OR ‘under-served countries’:ti,ab,kw OR ‘under-served nation’:ti,ab,kw OR ‘under-served nations’:ti,ab,kw OR ‘under-served population’:ti,ab,kw OR ‘under-served populations’:ti,ab,kw OR ‘underserved economy’:ti,ab,kw OR ‘underserved economies’:ti,ab,kw OR ‘derived countr*’:ti,ab,kw OR ‘deprived nation’:ti,ab,kw OR ‘deprived nations’:ti,ab,kw OR ‘derived population*’:ti,ab,kw OR ‘deprived economy’:ti,ab,kw OR ‘deprived economies’:ti,ab,kw OR ‘poor countr*’:ti,ab,kw OR ‘poor nation*’:ti,ab,kw OR ‘poor population*’:ti,ab,kw OR ‘poor econom*’:ti,ab,kw OR ‘poorer countr*’:ti,ab,kw OR ‘poorer nation*’:ti,ab,kw OR ‘poorer population*’:ti,ab,kw OR ‘poorer econom*’:ti,ab,kw OR ‘lmic’:ti,ab,kw OR ‘lmics’:ti,ab,kw OR ‘lami’:ti,ab,kw OR ‘transitional countr*’:ti,ab,kw OR ‘transitional nation’:ti,ab,kw OR ‘transitional nations’:ti,ab,kw OR ‘transitional econom*’:ti,ab,kw OR ‘transition countr*’:ti,ab,kw OR ‘transition nation*’:ti,ab,kw OR ‘transition econom*’:ti,ab,kw OR low ‘resource setting*’:ti,ab,kw OR ‘lower resource setting*’:ti,ab,kw OR ‘middle resource setting*’:ti,ab,kw OR ‘Third World*’:ti,ab,kw OR ‘south east asia*’:ti,ab,kw OR ‘middle east*’:ti,ab,kw OR ‘Afghan*’:ti,ab,kw OR ‘Angola*’:ti,ab,kw OR ‘Angolese*’:ti,ab,kw OR ‘Angolian*’:ti,ab,kw OR ‘Armenia*’:ti,ab,kw OR ‘Bangladesh*’:ti,ab,kw OR ‘Benin*’:ti,ab,kw OR ‘Bhutan*’:ti,ab,kw OR ‘Birma*’:ti,ab,kw OR ‘Burma*’:ti,ab,kw OR ‘Birmese*’:ti,ab,kw OR ‘Burmese*’:ti,ab,kw OR ‘Boliv*’:ti,ab,kw OR ‘Botswan*’:ti,ab,kw OR ‘burkina Faso*’:ti,ab,kw OR ‘Burundi*’:ti,ab,kw OR ‘Cabo Verde*’:ti,ab,kw OR ‘Cambod*’:ti,ab,kw OR ‘Cameroon*’:ti,ab,kw OR ‘Cape Verd*’:ti,ab,kw OR ‘Central Africa*’:ti,ab,kw OR ‘Chad’:ti,ab,kw OR ‘Comoro*’:ti,ab,kw OR ‘Congo*’:ti,ab,kw OR ‘Cote d/Ivoire*’:ti,ab,kw OR ‘Djibouti*’:ti,ab,kw OR ‘East Africa*’:ti,ab,kw OR ‘Eastern Africa*’:ti,ab,kw OR ‘Egypt*’:ti,ab,kw OR ‘El Salvador*’:ti,ab,kw OR ‘Equatorial Guinea*’:ti,ab,kw OR ‘Eritre*’:ti,ab,kw OR ‘Ethiopia*’:ti,ab,kw OR ‘Gabon*’:ti,ab,kw OR ‘Gambia*’:ti,ab,kw OR ‘Gaza*’:ti,ab,kw OR ‘Georgia Republic’/exp OR

For peer review only

'Ghan*':ti,ab,kw OR 'Guatemal*':ti,ab,kw OR 'Guinea':ti,ab,kw OR 'Haiti*':ti,ab,kw OR 'Hondur*':ti,ab,kw OR 'India*':ti,ab,kw OR 'Indones*':ti,ab,kw OR 'Ivory Coast*':ti,ab,kw OR 'Kenya*':ti,ab,kw OR 'Kiribati*':ti,ab,kw OR 'Kosovo*':ti,ab,kw OR 'Kyrgyz*':ti,ab,kw OR 'Lao PDR*':ti,ab,kw OR 'Laos*':ti,ab,kw OR 'Lesotho*':ti,ab,kw OR 'Liberia*':ti,ab,kw OR 'Madagascar*':ti,ab,kw OR 'Malaw*':ti,ab,kw OR 'Mali':ti,ab,kw OR 'Mauritan*':ti,ab,kw OR 'Mauriti*':ti,ab,kw OR 'Micronesi*':ti,ab,kw OR 'Mocambiqu*':ti,ab,kw OR 'Moldov*':ti,ab,kw OR 'Mongolia*':ti,ab,kw OR 'Morocc*':ti,ab,kw OR 'Mozambiqu*':ti,ab,kw OR 'Myanmar*':ti,ab,kw OR 'Namibia*':ti,ab,kw OR 'Nepal*':ti,ab,kw OR 'Nicaragua*':ti,ab,kw OR 'Niger*':ti,ab,kw OR 'North Korea*':ti,ab,kw OR 'Northern Korea*':ti,ab,kw OR 'Democratic People/s Republic of Korea':ti,ab,kw OR 'Pakistan*':ti,ab,kw OR 'Papua New Guinea*':ti,ab,kw OR 'Philippine*':ti,ab,kw OR 'Principe':ti,ab,kw OR 'Rhodesia*':ti,ab,kw OR 'Rwanda*':ti,ab,kw OR 'Samoa*':ti,ab,kw OR 'Sao Tome*':ti,ab,kw OR 'Senegal*':ti,ab,kw OR 'Sierra Leone*':ti,ab,kw OR 'Solomon Islands*':ti,ab,kw OR 'Somalia*':ti,ab,kw OR 'South Africa*':ti,ab,kw OR 'South Sudan*':ti,ab,kw OR 'Southern Africa*':ti,ab,kw OR 'Sri Lanka*':ti,ab,kw OR 'Sub Saharan Africa*':ti,ab,kw OR 'Subsaharan Africa*':ti,ab,kw OR 'Sudan*':ti,ab,kw OR 'Swaziland*':ti,ab,kw OR 'Syria*':ti,ab,kw OR 'Tajikist*':ti,ab,kw OR 'Tanzan*':ti,ab,kw OR 'Timor*':ti,ab,kw OR 'Togo*':ti,ab,kw OR 'Tonga*':ti,ab,kw OR 'Tunis*':ti,ab,kw OR 'Ugand*':ti,ab,kw OR 'Ukrain*':ti,ab,kw OR 'Uzbekistan*':ti,ab,kw OR 'Vanuatu*':ti,ab,kw OR 'Vietnam*':ti,ab,kw OR 'West Africa*':ti,ab,kw OR 'West Bank*':ti,ab,kw OR 'Western Africa*':ti,ab,kw OR 'Yemen*':ti,ab,kw OR 'Zaire*':ti,ab,kw OR 'Zambia*':ti,ab,kw OR 'Zimbabw*':ti,ab,kw)

AND

('Umbilical Arter*'/exp OR 'Uterine Artery'/exp OR 'Middle Cerebral Artery'/exp OR 'Ductus Venosus'/exp OR 'Umbilical Vein*'/exp OR 'Inferior Cava Vein'/exp OR 'Umbilical Arter*':ti,ab,kw OR 'Uterine Arter*':ti,ab,kw OR 'Middle Cerebral Arter*':ti,ab,kw OR 'Patent Ductus Venosus':ti,ab,kw OR 'Umbilical Vein*':ti,ab,kw OR 'Inferior Vena Cava':ti,ab,kw OR 'Cerebroplacental Ratio':ti,ab,kw OR 'CPR':ti,ab,kw OR 'Fetal Descending Aorta':ti,ab,kw OR 'FDA':ti,ab,kw OR 'Doppler Ultrasonography'/exp OR 'Doppler Ultrasound*':ti,ab,kw OR 'Doppler Ultrasonography':ti,ab,kw OR 'Uterine Artery Doppler':ti,ab,kw)

AND

('Stillbirth':ti,ab,kw OR 'Perinatal Death':ti,ab,kw OR 'Cesarean Section*':ti,ab,kw OR 'Caesarean Section*':ti,ab,kw OR 'Acidosis':ti,ab,kw OR 'Premature Birth':ti,ab,kw OR 'Neonatal Intensive Care':ti,ab,kw OR 'Fetal Growth Retard*':ti,ab,kw OR 'Newborn Respiratory Distress Syndrome*':ti,ab,kw OR 'Gestational Age':ti,ab,kw OR 'Birth Weight':ti,ab,kw OR 'Asphyxia Neonatorum':ti,ab,kw OR 'Apgar Score*':ti,ab,kw OR 'Length of Stay':ti,ab,kw OR 'Stillbirth'/exp OR 'Perinatal Death'/exp OR 'Perinatal Mortality'/exp OR 'Cesarean Section'/exp OR 'Acidosis'/exp OR 'Prematurity'/exp OR 'Newborn Intensive Care'/exp OR 'Intrauterine Growth Retardation'/exp OR 'Neonatal Respiratory Distress Syndrome'/exp OR 'Gestational Age'/exp OR 'Birth Weight'/exp OR 'Newborn Hypoxia'/exp OR 'Apgar Score'/exp OR 'Length of Stay'/exp OR 'Pregnancy':ti,ab,kw OR 'Pregnancies':ti,ab,kw OR 'Gestation':ti,ab,kw OR 'Pregnant':ti,ab,kw OR 'Pregnancy'/exp)

For peer review only

PUBMED (MEDLINE)

("Developing Countries"[Mesh] OR developing countr*[tiab] OR developing nation*[tiab] OR developing population*[tiab] OR developing econom*[tiab] OR undeveloped countr*[tiab] OR undeveloped nation*[tiab] OR "undeveloped economy"[tiab] OR "undeveloped economies"[tiab] OR least developed countr*[tiab] OR least developed nation*[tiab] OR "least developed economy"[tiab] OR "least developed economies"[tiab] OR less-developed countr*[tiab] OR less-developed nation*[tiab] OR "less-developed population"[tiab] OR "less-developed populations"[tiab] OR less-developed econom*[tiab] OR lesser developed countr*[tiab] OR lesser developed nation*[tiab] OR "lesser developed population"[tiab] OR "lesser developed populations"[tiab] OR "lesser developed economy"[tiab] OR "lesser developed economies"[tiab] OR under-developed countr*[tiab] OR under-developed nation*[tiab] OR underdeveloped countr*[tiab] OR underdeveloped nation*[tiab] OR underdeveloped population*[tiab] OR underdeveloped econom*[tiab] OR low income countr*[tiab] OR middle income countr*[tiab] OR low income nation*[tiab] OR middle income nation*[tiab] OR low income population*[tiab] OR middle income population*[tiab] OR low income econom*[tiab] OR middle income econom*[tiab] OR lower income countr*[tiab] OR lower income nation*[tiab] OR lower income population*[tiab] OR "lower income economy"[tiab] OR "lower income economies"[tiab] OR resource limited[tiab] OR low resource countr*[tiab] OR lower resource countr*[tiab] OR low resource nation*[tiab] OR low resource population*[tiab] OR "low resource economy"[tiab] OR "low resource economies"[tiab] OR underserved countr*[tiab] OR underserved nation*[tiab] OR underserved population*[tiab] OR "underserved economy"[tiab] OR "underserved economies"[tiab] OR "under-served country"[tiab] OR "under-served countries"[tiab] OR "under-served nation"[tiab] OR "under-served nations"[tiab] OR "under-served population"[tiab] OR "under-served populations"[tiab] OR "underserved economy"[tiab] OR "underserved economies"[tiab] OR derived countr*[tiab] OR "deprived nation"[tiab] OR "deprived nations"[tiab] OR derived population*[tiab] OR "deprived economy"[tiab] OR "deprived economies"[tiab] OR poor countr*[tiab] OR poor nation*[tiab] OR poor population*[tiab] OR poor econom*[tiab] OR poorer countr*[tiab] OR poorer nation*[tiab] OR poorer population*[tiab] OR poorer econom*[tiab] OR lmic[tiab] OR lmics[tiab] OR lami[tiab] OR transitional countr*[tiab] OR "transitional nation"[tiab] OR "transitional nations"[tiab] OR transitional econom*[tiab] OR transition countr*[tiab] OR transition nation*[tiab] OR transition econom*[tiab] OR low resource setting*[tiab] OR lower resource setting*[tiab] OR middle resource setting*[tiab] OR Third World*[tiab] OR south east asia*[tw] OR middle east*[tw] OR Afghan*[tw] OR Angola*[tw] OR Angolese*[tw] OR Angolian*[tw] OR Armenia*[tw] OR Bangladesh*[tw] OR Benin*[tw] OR Bhutan*[tw] OR Birma*[tw] OR Burma*[tw] OR Birmese*[tw] OR Burmese*[tw] OR Boliv*[tw] OR Botswan*[tw] OR burkina Faso*[tw] OR Burundi*[tw] OR Cabo Verde*[tw] OR Cambod*[tw] OR Cameroon*[tw] OR Cape Verd*[tw] OR Central Africa*[tw] OR Chad[tw] OR Comoro*[tw] OR Congo*[tw] OR Cote d'Ivoire*[tw] OR Djibouti*[tw] OR East Africa*[tw] OR Eastern Africa*[tw] OR Egypt*[tw] OR El Salvador*[tw] OR Equatorial Guinea*[tw] OR Eritre*[tw] OR Ethiopia*[tw] OR Gabon*[tw] OR Gambia*[tw] OR Gaza*[tw] OR "Georgia Republic"[Mesh] OR Ghan*[tw] OR Guatemal*[tw] OR Guinea[tw] OR Haiti*[tw] OR Hondur*[tw] OR India*[tw] OR Indones*[tw] OR Ivory Coast*[tw] OR Kenya*[tw] OR Kiribati*[tw] OR Kosovo*[tw] OR Kyrgyz*[tw] OR Lao PDR*[tw] OR Laos*[tw] OR Lesotho*[tw] OR Liberia*[tw] OR Madagascar*[tw] OR Malaw*[tw] OR Mali[tw] OR Mauritan*[tw] OR Mauriti*[tw] OR Micronesi*[tw] OR Mocambiqu*[tw] OR Moldov*[tw] OR Mongolia*[tw] OR Morocc*[tw] OR Mozambiqu*[tw] OR Myanmar*[tw] OR Namibia*[tw] OR Nepal*[tw] OR Nicaragua*[tw] OR Niger*[tw] OR North Korea*[tw] OR Northern Korea*[tw] OR "Democratic People s Republic of Korea"[tiab] OR "Democratic People's Republic of Korea"[Mesh] OR Pakistan*[tw] OR Papua New Guinea*[tw] OR Philippine*[tw] OR Principe[tw] OR Rhodesia*[tw] OR Rwanda*[tw] OR Samoa*[tw] OR Sao Tome*[tw] OR Senegal*[tw] OR Sierra Leone*[tw] OR Solomon Islands*[tw]

For peer review only

OR Somalia*[tw] OR South Africa*[tw] OR South Sudan*[tw] OR Southern Africa*[tw] OR Sri Lanka*[tw] OR Sub Saharan Africa*[tw] OR Subsaharan Africa*[tw] OR Sudan*[tw] OR Swaziland*[tw] OR Syria*[tw] OR Tajikist*[tw] OR Tanzan*[tw] OR Timor*[tw] OR Togo*[tw] OR Tonga*[tw] OR Tunis*[tw] OR Ugand*[tw] OR Ukrain*[tw] OR Uzbekistan*[tw] OR Vanuatu*[tw] OR Vietnam*[tw] OR West Africa*[tw] OR West Bank*[tw] OR Western Africa*[tw] OR Yemen*[tw] OR Zaire*[tw] OR Zambia*[tw] OR Zimbabw*[tw])

AND

("Umbilical Arteries"[Mesh] OR "Uterine Artery"[Mesh] OR "Middle Cerebral Artery"[Mesh] OR "Ductus Venosus" [Supplementary Concept] OR "Umbilical Veins"[Mesh] OR "Vena Cava, Inferior"[Mesh] OR Umbilical Arter*[tiab] OR Uterine Arter*[tiab] OR Middle Cerebral Arter*[tiab] OR Patent Ductus Venosus[tiab] OR Umbilical Vein*[tiab] OR Inferior Vena Cava[tiab] OR Cerebroplacental Ratio[tiab] OR CPR[tiab] OR Fetal Descending Aorta[tiab] OR FDA[tiab] OR "Ultrasonography, Doppler"[Mesh] OR Doppler Ultrasound*[Title/Abstract] OR Doppler Ultrasonography[Title/Abstract] OR Uterine Artery Doppler[Title/Abstract])

AND

("Stillbirth"[tiab] OR "Perinatal Death"[tiab] OR "Cesarean Section*"[tiab] OR "Caesarean Section*"[tiab] OR Acidosis[tiab] OR Premature Birth[tiab] OR Neonatal Intensive Care"[tiab] OR Fetal Growth Retard*[tiab] OR Newborn Respiratory Distress Syndrome*[tiab] OR Gestational Age[tiab] OR Birth Weight[tiab] OR Asphyxia Neonatorum[tiab] OR Apgar Score*[tiab] OR Length of Stay"[tiab] OR "Stillbirth"[Mesh] OR "Perinatal Death"[Mesh] OR "Cesarean Section"[Mesh] OR "Acidosis"[Mesh] OR "Premature Birth"[Mesh] OR "Intensive Care, Neonatal"[Mesh] OR "Fetal Growth Retardation"[Mesh] OR "Respiratory Distress Syndrome, Newborn"[Mesh] OR "Gestational Age"[Mesh] OR "Birth Weight"[Mesh] OR "Asphyxia Neonatorum"[Mesh] OR "Apgar Score"[Mesh] OR "Length of Stay"[Mesh] OR Pregnancy[Title/Abstract] OR Pregnancies[Title/Abstract] OR Gestation[Title/Abstract] OR Pregnant[Title/Abstract] OR "Pregnancy"[Mesh])

COCHRANE

'developing countr*' OR 'developing nation*' OR 'developing population*' OR 'developing econom*' OR 'undeveloped countr*' OR 'undeveloped nation*' OR 'undeveloped economy' OR 'undeveloped economies' OR 'least developed countr*' OR 'least developed nation*' OR 'least developed economy' OR 'least developed economies' OR 'less-developed countr*' OR 'less-developed nation*' OR 'less-developed population' OR 'less-developed populations' OR 'less-developed econom*' OR 'lesser developed countr*' OR 'lesser developed nation*' OR 'lesser developed population' OR 'lesser developed populations' OR 'lesser developed economy' OR 'lesser developed economies' OR 'under-developed countr*' OR 'under-developed nation*' OR 'underdeveloped countr*'OR 'underdeveloped nation*' OR 'underdeveloped population*' OR 'underdeveloped econom*' OR 'low income countr*' OR 'middle income countr*' OR 'low income nation*' OR 'middle income nation*' OR 'low income population*' OR 'middle income population*' OR 'low income econom*' OR 'middle income econom*' OR 'lower income countr*' OR 'lower income nation*' OR 'lower income population*' OR 'lower income economy' OR 'lower income economies' OR 'resource limited' OR 'low resource countr*' OR 'lower resource countr*' OR 'low resource nation*' OR 'low resource population*' OR 'low resource economy' OR 'low resource economies' OR 'underserved countr*' OR 'underserved nation*' OR 'underserved

For peer review only

population*' OR 'underserved economy' OR 'underserved economies' OR 'under-served country' OR 'under-served countries' OR 'under-served nation' OR 'under-served nations' OR 'under-served population' OR 'under-served populations' OR 'underserved economy' OR 'underserved economies' OR 'derived countr*' OR 'deprived nation' OR 'deprived nations' OR 'derived population*' OR 'deprived economy' OR 'deprived economies' OR 'poor countr*' OR 'poor nation*' OR 'poor population*' OR 'poor econom*' OR 'poorer countr*' OR 'poorer nation*' OR 'poorer population*' OR 'poorer econom*' OR 'lmic' OR 'lmics' OR 'lami' OR 'transitional countr*' OR 'transitional nation' OR 'transitional nations' OR 'transitional econom*' OR 'transition countr*' OR 'transition nation*' OR 'transition econom*' OR low 'resource setting*' OR 'lower resource setting*' OR 'middle resource setting*' OR 'Third World*' OR 'south east asia*' OR 'middle east*' OR 'Afghan*' OR 'Angola*' OR 'Angolese*' OR 'Angolian*' OR 'Armenia*' OR 'Bangladesh*' OR 'Benin*' OR 'Bhutan*' OR 'Birma*' OR 'Burma*' OR 'Birmese*' OR 'Burmese*' OR 'Boliv*' OR 'Botswan*' OR 'burkina Faso*' OR 'Burundi*' OR 'Cabo Verde*' OR 'Cambod*' OR 'Cameroon*' OR 'Cape Verd*' OR 'Central Africa*' OR 'Chad' OR 'Comoro*' OR 'Congo*' OR 'Cote d'Ivoire*' OR 'Djibouti*' OR 'East Africa*' OR 'Eastern Africa*' OR 'Egypt*' OR 'El Salvador*' OR 'Equatorial Guinea*' OR 'Eritre*' OR 'Ethiopia*' OR 'Gabon*' OR 'Gambia*' OR 'Gaza*' OR 'Georgia Republic' OR 'Ghan*' OR 'Guatemal*' OR 'Guinea' OR 'Haiti*' OR 'Hondur*' OR 'India*' OR 'Indones*' OR 'Ivory Coast*' OR 'Kenya*' OR 'Kiribati*' OR 'Kosovo*' OR 'Kyrgyz*' OR 'Lao PDR*' OR 'Laos*' OR 'Lesotho*' OR 'Liberia*' OR 'Madagascar*' OR 'Malaw*' OR 'Mali' OR 'Mauritan*' OR 'Mauriti*' OR 'Micronesi*' OR 'Mocambiqu*' OR 'Moldov*' OR 'Mongolia*' OR 'Morocc*' OR 'Mozambiqu*' OR 'Myanmar*' OR 'Namibia*' OR 'Nepal*' OR 'Nicaragua*' OR 'Niger*' OR 'North Korea*' OR 'Northern Korea*' OR 'Democratic People's Republic of Korea' OR 'Pakistan*' OR 'Papua New Guinea*' OR 'Philippine*' OR 'Principe' OR 'Rhodesia*' OR 'Rwanda*' OR 'Samoa*' OR 'Sao Tome*' OR 'Senegal*' OR 'Sierra Leone*' OR 'Solomon Islands*' OR 'Somalia*' OR 'South Africa*' OR 'South Sudan*' OR 'Southern Africa*' OR 'Sri Lanka*' OR 'Sub Saharan Africa*' OR 'Subsaharan Africa*' OR 'Sudan*' OR 'Swaziland*' OR 'Syria*' OR 'Tajikist*' OR 'Tanzan*' OR 'Timor*' OR 'Togo*' OR 'Tonga*' OR 'Tunis*' OR 'Ugand*' OR 'Ukrain*' OR 'Uzbekistan*' OR 'Vanuatu*' OR 'Vietnam*' OR 'West Africa*' OR 'West Bank*' OR 'Western Africa*' OR 'Yemen*' OR 'Zaire*' OR 'Zambia*' OR 'Zimbabw*'

AND
'Umbilical Arter*' OR 'Uterine Artery' OR 'Middle Cerebral Artery' OR 'Ductus Venosus' OR 'Umbilical Vein*' OR 'Inferior Cava Vein' OR 'Uterine Arter*' OR 'Middle Cerebral Arter*' OR 'Patent Ductus Venosus' OR 'Inferior Vena Cava' OR 'Cerebroplacental Ratio' OR 'CPR' OR 'Fetal Descending Aorta' OR 'FDA' OR 'Doppler Ultrasonography' OR 'Doppler Ultrasound*' OR 'Doppler Ultrasonography' OR 'Uterine Artery Doppler'

AND

'Stillbirth' OR 'Perinatal Death' OR 'Cesarean Section*' OR 'Caesarean Section*' OR 'Acidosis' OR 'Premature Birth' OR 'Neonatal Intensive Care' OR 'Fetal Growth Retard*' OR 'Newborn Respiratory Distress Syndrome*' OR 'Gestational Age' OR 'Birth Weight' OR 'Asphyxia Neonatorum' OR 'Apgar Score*' OR 'Perinatal Mortality' OR 'Cesarean Section' OR 'Prematurity' OR 'Newborn Intensive Care' OR 'Intrauterine Growth Retardation' OR 'Neonatal Respiratory Distress Syndrome' OR 'Gestational Age' OR 'Birth Weight' OR 'Newborn Hypoxia' OR 'Length of Stay' OR 'Pregnancy' OR 'Pregnancies' OR 'Gestation' OR 'Pregnant'

For peer review only

SCOPUS

TITLE-ABS-KEY("developing countr*" OR "developing nation*" OR "developing population*" OR "developing econom*" OR "undeveloped countr*" OR "undeveloped nation*" OR "undeveloped economy" OR "undeveloped economies" OR "least developed countr*" OR "least developed nation*" OR "least developed economy" OR "least developed economies" OR "less-developed countr*" OR "less-developed nation*" OR "less-developed population" OR "less-developed populations" OR "less-developed econom*" OR "lesser developed countr*" OR "lesser developed nation*" OR "lesser developed population" OR "lesser developed populations" OR "lesser developed economy" OR "lesser developed economies" OR "under-developed countr*" OR "under-developed nation*" OR "underdeveloped countr*" OR "underdeveloped nation*" OR "underdeveloped population*" OR "underdeveloped econom*" OR "low income countr*" OR "middle income countr*" OR "low income nation*" OR "middle income nation*" OR "low income population*" OR "middle income population*" OR "low income econom*" OR "middle income econom*" OR "lower income countr*" OR "lower income nation*" OR "lower income population*" OR "lower income economy" OR "lower income economies" OR "resource limited" OR "low resource countr*" OR "lower resource countr*" OR "low resource nation*" OR "low resource population*" OR "low resource economy" OR "low resource economies" OR "underserved countr*" OR "underserved nation*" OR "underserved population*" OR "underserved economy" OR "underserved economies" OR "under-served country" OR "under-served countries" OR "under-served nation" OR "under-served nations" OR "under-served population" OR "under-served populations" OR "underserved economy" OR "underserved economies" OR "derived countr*" OR "deprived nation" OR "deprived nations" OR "derived population*" OR "deprived economy" OR "deprived economies" OR "poor countr*" OR "poor nation*" OR "poor population*" OR "poor econom*" OR "poorer countr*" OR "poorer nation*" OR "poorer population*" OR "poorer econom*" OR "lmic" OR "lmics" OR "lami" OR "transitional countr*" OR "transitional nation" OR "transitional nations" OR "transitional econom*" OR "transition countr*" OR "transition nation*" OR "transition econom*" OR low "resource setting*" OR "lower resource setting*" OR "middle resource setting*" OR "Third World*" OR "south east asia*" OR "middle east*" OR "Afghan*" OR "Angola*" OR "Angolese*" OR "Angolian*" OR "Armenia*" OR "Bangladesh*" OR "Benin*" OR "Bhutan*" OR "Birma*" OR "Burma*" OR "Birmese*" OR "Burmese*" OR "Boliv*" OR "Botswan*" OR "burkina Faso*" OR "Burundi*" OR "Cabo Verde*" OR "Cambod*" OR "Cameroon*" OR "Cape Verd*" OR "Central Africa*" OR "Chad" OR "Comoro*" OR "Congo*" OR "Cote d/Ivoire*" OR "Djibouti*" OR "East Africa*" OR "Eastern Africa*" OR "Egypt*" OR "El Salvador*" OR "Equatorial Guinea*" OR "Eritre*" OR "Ethiopia*" OR "Gabon*" OR "Gambia*" OR "Gaza*" OR "Georgia Republic" OR "Ghan*" OR "Guatemal*" OR "Guinea" OR "Haiti*" OR "Hondur*" OR "India*" OR "Indones*" OR "Ivory Coast*" OR "Kenya*" OR "Kiribati*" OR "Kosovo*" OR "Kyrgyz*" OR "Lao PDR*" OR "Laos*" OR "Lesotho*" OR "Liberia*" OR "Madagascar*" OR "Malaw*" OR "Mali" OR "Mauritan*" OR "Mauriti*" OR "Micronesi*" OR "Mocambiqu*" OR "Moldov*" OR "Mongolia*" OR "Morocc*" OR "Mozambiqu*" OR "Myanmar*" OR "Namibia*" OR "Nepal*" OR "Nicaragua*" OR "Niger*" OR "North Korea*" OR "Northern Korea*" OR "Democratic People/s Republic of Korea" OR "Pakistan*" OR "Papua New Guinea*" OR "Philippine*" OR "Principe" OR "Rhodesia*" OR "Rwanda*" OR "Samoa*" OR "Sao Tome*" OR "Senegal*" OR "Sierra Leone*" OR "Solomon Islands*" OR "Somalia*" OR "South Africa*" OR "South Sudan*" OR "Southern Africa*" OR "Sri Lanka*" OR "Sub Saharan Africa*" OR "Subsaharan Africa*" OR "Sudan*" OR "Swaziland*" OR "Syria*" OR "Tajikist*" OR "Tanzan*" OR "Timor*" OR "Togo*" OR "Tonga*" OR "Tunis*" OR "Ugand*" OR "Ukrain*" OR "Uzbekistan*" OR "Vanuatu*" OR "Vietnam*" OR "West Africa*" OR "West Bank*" OR "Western Africa*" OR "Yemen*" OR "Zaire*" OR "Zambia*" OR "Zimbabw*")

AND

TITLE-ABS-KEY("Stillbirth" OR "Perinatal Death" OR "Cesarean Section*" OR "Caesarean Section*" OR “Acidosis” OR “Premature Birth” OR “Neonatal Intensive Care" OR “Fetal Growth Retard*” OR “Newborn Respiratory Distress Syndrome*” OR “Gestational Age” OR “Birth Weight” OR “Asphyxia Neonatorum” OR “Apgar Score*” OR “Length of Stay" OR "Stillbirth" OR "Perinatal Death" OR "Cesarean Section" OR "Acidosis" OR "Premature Birth" OR "Intensive Care, Neonatal" OR "Fetal Growth Retardation" OR "Respiratory Distress Syndrome, Newborn" OR "Gestational Age" OR "Birth Weight" OR "Asphyxia Neonatorum" OR "Apgar Score" OR "Length of Stay" OR “Pregnancy” OR “Pregnancies” OR “Gestation” OR “Pregnant” OR "Pregnancy")

AND

TITLE-ABS-KEY("Umbilical Arteries" OR "Uterine Artery" OR "Middle Cerebral Artery" OR "Ductus Venosus" OR "Umbilical Veins" OR "Vena Cava, Inferior" OR “Umbilical Arter*” OR “Uterine Arter*” OR “Middle Cerebral Arter*” OR “Patent Ductus Venosus” OR “Umbilical Vein*” OR “Inferior Vena Cava” OR “Cerebroplacental Ratio” OR “CPR” OR “Fetal Descending Aorta” OR “FDA” OR "Ultrasonography, Doppler" OR “Doppler Ultrasound*”OR” Doppler Ultrasonography” OR “Uterine Artery Doppler”)

For peer review only

Appendix S2. List of full-text articles excluded with reasons

a) Country income level: 3 studies

1. El Shourbagy, S., Elsakhawy, M. (2012). Prediction of fetal anemia by middle cerebral artery Doppler. *Middle East Fertility Society Journal, 17(4), 275-282.*
2. Haley, J., Tuffnell, D. J., Johnson, N. (1997). Randomized controlled trial of cardiotocography versus umbilical artery Doppler in the management of small for gestational age fetuses. *British Journal of Obstetrics and Gynaecology, 104(4), 431-435).*
3. Morales-Rosello, J., Dias, T., Khalil, A., Fornes-Ferrer, V., Ciammella, R., Gimenez-Roca, L., Perales-Marin, A., Thilaganathan, B. (2018). Birth-weight differences at term are explained by placental dysfunction and not by maternal ethnicity. *Ultrasound Obstet Gynecol, 52(4), 488-493.*

b) Design and quality: 9 studies

1. Abidoye, I. A., Ayoola, O. O., Idowu, B., Aderibigbe, A. S., Loto, O. M. (2017). Uterine artery Doppler velocimetry in hypertensive disorder of pregnancy in Nigeria. *J Ultrason, 17(71)) 253-258.*
2. Agarwal, R., Tiwari, A., Wadhwa, N., Radhakrishnan, G., Bhatt, S., Batra, P. (2017). Abnormal umbilical artery Doppler velocimetry and placental histopathological correlation in fetal growth restriction. *South African Journal of Obstetrics and Gynaecology, 23(1), 12-16.*
3. Ali, A., Ara, I., Sultana, R., Akram, F., Zaib, M. J. (2014). Comparison of perinatal outcome of growth restricted fetuses with normal and abnormal umbilical artery Doppler waveforms. *Journal of Ayub Medical College, Abottabad: JAMC, 26(3), 344-348.*
4. Kumar, S., Datta, S., Mittal, S., Roy, K. K. (2002). Doppler flow studies in middle cerebral and umbilical arteries in growth retarded and normal pregnancies. *JK Science, 4(0), 185-189*
5. Mufenda, J., Gebhardt, S., van Rooyen, R., Theron, G. (2015). Introducing a Mobile-Connected Umbilical Doppler Device (UmbiFlow) into a Primary Care Maternity Setting: Does This Reduce Unnecessary Referrals to Specialised Care? Results of a Pilot Study in Kraaifontein, South Africa. *PLoS One, 10(11) e0142743.*
6. Nguku, S. W., Wanyoike-Gichuhi, J., Aywak, A. A. (2006). Biophysical profile scores and resistance indices of the umbilical artery as seen in patients with pregnancy induced hypertension. *East African Medical Journal, 83(3), 96-101*
7. Nkosi, S., Makin, J., Hlongwane, T. M. A. G., & Pattinson, R. C. (2019). Screening and managing a low-risk pregnant population using continuous-wave Doppler ultrasound in a low-income population: A cohort analytical study. *SAMJ: South African Medical Journal*, *109*(5), 347-352.
8. Siddiqui, T. S., Asim, A., Ali, S., Tariq, A. (2014). Comparison of perinatal outcome in growth restricted fetuses retaining normal umbilical artery Doppler flow to those with diminished end-diastolic flow. *Journal of Ayub Medical College, Abbottabad: JAMC, 26(2), 221-224.*
9. Kachewar, S. G., Gandage, S. G., Pawar, H. J. (2012). An Indian study of novel non-invasive method of screening for foetal anaemia. *Journal of Clinical and Diagnostic Research, 6(4), 688-691.*

For peer review only

c) Outcomes: 11 studies

1. Adekanmi, A. J., Roberts, A., Akinmoladun, J. A., & Adeyinka, A. O. (2019). Uterine and umbilical artery doppler in women with pre-eclampsia and their pregnancy outcomes. *Nigerian Postgraduate Medical Journal*, *26*(2), 106.
2. El Behery, M. M., Siam, S., Seksaka, M. A., Mansou, S. M. (2013). Uterine artery Doppler and urinary hyperglycosylated HCG as predictors of threatened abortion outcome. *Middle East Fertility Society Journal, 19(1), 42-46*.
3. El-Mashad, A. I., Mohamed, M. A., Elahadi Farag, M. A., Ahmad, M. K., Ismail, Y. (2011). Role of uterine artery Doppler velocimetry indices and plasma adrenomedullin level in women with unexplained recurrent pregnancy loss. *Journal of Obstetrics and Gynaecology Research, 37(1), 51-57*.
4. Geerts, L., Van der Merwe, E., Theron, A., Rademan, K. (2016). Placental insufficiency among high-risk pregnancies with a normal umbilical artery resistance index after 32 weeks. *Int J Gynaecol Obstet, 135(1), 38-42*.
5. Kumar, B. S., Sarmila, K., Prasad, K. S. (2012). Prediction of preeclampsia by midtrimester uterine artery doppler velocimetry in high-risk and low-risk women. *Journal of Obstetrics and Gynecology of India, 62(3), 297-300*.
6. Maged. A. M., Elnassery, N., Fouad, M., Abdelhafiz, A., Al Mostafa, W. (2015). Third-trimester uterine artery Doppler measurement and maternal postpartum outcome among patients with severe pre-eclampsia. *International Journal of Gynecology and Obstetrics, 131(1), 49-53*.
7. Prajapati, S. R., Maitra, N. (2013). Prediction of pre-eclampsia by a combination of history, uterine artery doppler, and mean arterial pressure (A Prospective Study of 200 Cases). *Journal of Obstetrics and Gynecology of India, 63(1), 32-36*.
8. Sebastian, A., Raj, T. S., Yenuberi, H., Job, V., Varuhghese, S., & Regi, A. (2019). Angiogenic factors and uterine artery Doppler in predicting preeclampsia and associated adverse outcomes in a tertiary hospital in south India. *Pregnancy hypertension*, *16*, 26.
9. Shehata, N. A. A., Ali, H. A. A., Hassan, A., Katta, M. A., Ali, A. S. F. (2018). Doppler and biochemical assessment for the prediction of early pregnancy outcome in patients experiencing threatened spontaneous abortion. *Int J Gynaecol Obstet, 143(2), 150-155*.
10. Yusuf, M., Galadanci, H., Ismail, A., Aliyu, L. D., Danbatta, A. H. (2017). Uterine artery doppler velocimetry for the prediction of preeclampsia among high-risk pregnancies in low-resource setting: Our experience at aminu Kano teaching hospital, Kano, Nigeria. *Donald School Journal of Ultrasound in Obstetrics and Gynecology, 11(3), 197-202*
11. Puri, M. S., Deshpande, H., Kohli, S., Sharma, K., Singhania, S. (2013). A study of uterine artery colour doppler at 20-24 weeks gestation as a predictor of pregnancy induced hypertension and intra uterine growth restriction from industrial town in Western India. *Research Journal of Pharmaceutical, Biological and Chemical Sciences, 4(1), 698-705*.

For peer review only

Appendix S3. The aims of the selected studies and risk profiles of the women recruited

First Author	Aim of study	Dating method	Risk Profile	Participant risk profile details in the article
Abdallah et al., 2019	To study the value of umbilical artery Doppler indices in predicting the risk of intrapartum and neonatal outcomes in pregnancies with and without nuchal cord.	LMP or first trimester ultrasound	Low risk	Primigravida >=37 weeks admitted in labor to the delivery unit. Women with BMI >30 kg/m2, multiple pregnancy, fetal malpresentation, fetal demise, chorioamnionitis, meconium-stained liquor, associated medical disorder (hypertension, diabetes, autoimmune disease, etc.), perinatal complication (e.g. placental abruption), fetal malformation or abnormal fetal growth were excluded from the study.
Agbaje et al., 2018	To assess umbilical artery Doppler findings in women with sickle cell anemia in the local environment at the onset of the third trimester and compare with obstetric outcomes.	LMP and/or early dating sonograms	High-risk	Sickle cell anemia.
Alanwar et al., 2018	To assess the efficacy of fetal middle cerebral artery/umbilical artery pulsatility index ratio (cerebroplacental ratio CPR) in predicting the occurrence of adverse perinatal outcomes in pregnancies complicated with severe pre-eclampsia.	Not specified	High-risk	Pregnancies complicated with severe pre-eclampsia.
Allam et al., 2013	To investigate, in high-risk pregnancies, the prediction of neonatal acidosis using DV, MCA and UA Doppler studies and subsequently to determine the best parameters and cutoff values.	Not specified	High-risk	Suspected IUGR, oligohydramnios, preeclampsia, or placental vascular dysfunction documented by abnormal umbilical artery pulsatility index by local reference ranges.
Anshul et al., 2010	To evaluate the role of umbilical artery Doppler in growth-restricted fetuses.	LMP and first trimester dating scan	High-risk	SGA foetuses, some mothers had hypertensive disorder, anemia, bad obstetric history
Bano et al., 2010	To evaluate the usefulness of the pulsatility index (PI) of the umbilical artery (UA) and that of the middle cerebral artery (MCA), as well as the ratio of the MCA PI to the UA PI (C/U ratio), in the diagnosis of small-for-gestational-age (SGA) fetuses and the prediction of adverse perinatal outcome.	Not specified	High risk	Clinical suspicion of FGR

For peer review only

Dhand et al., 2011	To compare the role of the middle cerebral artery and umbilical artery Doppler pulsatility indices in predicting the fetal outcome in intrauterine growth restriction.	LMP and fetal biometry <22weeks	High risk	SGA fetuses
Dorman et al., 2002	To determine whether impaired uteroplacental blood flow might account for the low infant birth weight associated with maternal falciparum malaria infection.	LMP and fetal biometry	High-risk	Maternal falciparum malaria infection.
Ebrashy et al., 2005	To evaluate the accuracy of middle cerebral/umbilical artery resistance index (C/U RI) ratio in predicting acidemia and low Apgar score at 5 minutes after birth in the infants of women with preeclampsia.	Fetal biometry (BPD, AC and FL)	High-risk	Pre-eclampsia women
Geerts et al., 2007	To assess the prognostic value of ultrasound findings and fetoplacental Doppler indices in severe preterm preeclampsia in identifying fetuses at high risk of death, major morbidity or long-term compromise.	LMP and fetal biometry	High-risk	Women with severe pre-eclampsia
Khanduri et al., 2013	To measure the pulsatility index (PI) and resistive index (RI) of the middle cerebral artery (MCA) and umbilical artery (UA) in predicting fetal growth restriction.	LMP and first or second trimester ultrasound	High-risk	Clinical suspicion of FGR
Kumari et al., 2019	To assess the correlation between fetal blood vessel Doppler measurements and fetal anemia among Rhesus isoimmunized pregnancies after two intrauterine transfusions as a potential guide to therapy.	Not specified	High risk	Rhesus isoimmunized complicated pregnancies
Lakhkar et al., 2006	To determine and compare the diagnostic performance of Doppler sonography of fetal middle cerebral artery (MCA), descending abdominal aorta (DAA), umbilical artery (UA), umbilical vein (UV) and inferior vena cava (IVC) for prediction of adverse perinatal outcome in suspected intrauterine growth retardation (IUGR) and pre-eclampsia (PET).	LMP, clinical gestational age, 1st or 2nd trimester biometry	High risk	Preeclampsia and suspicion of growth-restricted fetuses

For peer review only

Lakshmi et al., 2013	To determine outcomes of preterm infants with history of absent/reversed end-diastolic umbilical artery Doppler flow (AREDF) vs. infants with forward end-diastolic flow (FEDF).	LMP or first trimester ultrasound	High-risk	FGR, pregnancy induced hypertension, h/o previous intrauterine death
Malik et al., 2013	To determine the role of ultrasonography in screening high-risk mothers for detection of IUGR, to find out the impact of fetal parameters on the extent of IUGR, correlation between the sonographic pattern of IUGR and the birth weight, and to find out the sensitivities of various fetal parameters and their evaluation against each other and against the birth weight.	LMP	High-risk	FGR; hypertensive disorder; pre-eclampsia
Masihi et al.2019	To determine the relationship between the fetal middle cerebral artery and the umbilical artery ratio on color Doppler sonography with fetal distress at 38-40 weeks of gestation.	First trimester ultrasound	Low risk	Women that had uncomplicated pregnancies
Mullick et al., 1993	To explore whether measurement of umbilical artery blood velocity waveform between 22 and 26 weeks might predict pregnancies destined to become complicated by pregnancy could induce hypertension (PIH) and/or fetal growth restriction (IUGR).	Not specified	Low and high-risk	Women attending routine antenatal (any risk profile).
Nagar et al., 2015	To evaluate the predictive values of Uterine and Umbilical artery Doppler indices in high-risk pregnancies.	LMP and ultrasound before 21 weeks	High risk	History of preeclampsia or eclampsia in previous pregnancy pre-existing medical disorders like: Diabetes, Renal disease, Epilepsy, Autoimmune disease, Thrombophilia, and Hypertension, History of IUGR or still birth, history of abruptio placentae, preeclampsia or pregnancy-induced hypertension current, Nulliparity, Extremes of age (<20 years and >35 years).

For peer review only

Najam et al., 2016	To assess the predictive value of the cerebroplacental ratio in the detection of perinatal outcome in high-risk pregnancies in comparison to its components.	Not specified	Low and high-risk	Pregnancies undergoing routine antenatal (any risk profile).
Nouh et al., 2011	To assess the value of uterine artery Doppler screening during pregnancy in predicting adverse pregnancy outcomes in women with polycystic ovary syndrome (PCOS).	LMP and first trimester ultrasound	High-risk	Primigravida with ovulatory polycystic ovary syndrome (PCOS)
Pares et al., 2008	To evaluate the accuracy of middle cerebral artery peak systolic velocity (MCA-PSV) associated with descending thoracic aorta mean velocity (DTA-MV) in the prediction of fetal anemia.	Sonographic exam at <= 20 weeks	High-risk	Fetuses at risk for anemia because of maternal alloimmunization to red-cell antigens
Pattinson et al., 1991	To investigate whether abnormalities in Doppler waveform can predict the outcome of pregnancy accurately before other clinical signs develop	LMP and biometry: 16-20 weeks	High risk	SGA, preeclampsia and pregnancy wastage
Pattinson et al., 1993	To describe the prevalence and natural history of absent end-diastolic velocities (AEDV) in the umbilical artery of the fetus between 16 and 24 weeks gestation, and to evaluate its role as a screening test for identifying high-risk pregnancies.	Not specified	Low and high-risk	Pregnancies undergoing routine antenatal (any risk profile).
Phupong et al., 2003	To assess the value of uterine artery notching as a screening test for preeclampsia and fetal growth restriction in a low-risk population of healthy pregnant women.	LMP and first trimester ultrasound	Low-risk	Healthy pregnant women
Rani et al., 2016	To assess the accuracy of the middle cerebral artery (MCA) and umbilical artery (UmA), pulsatility index (PI) and resistance index (RI) in predicting perinatal outcome in pregnancies complicated by preeclampsia with or without intrauterine growth restriction (IUGR).	Not specified	Low and high-risk	Women attending routine antenatal (any risk profile).

For peer review only

Rocca et al., 1995	To test the value of routine Doppler study of the umbilical artery to predict the perinatal outcome in pre-eclamptic patients.	Not specified	High risk	Pre-eclampsia women
Verma et al., 2016	To assess the predictive value of uterine artery Doppler imaging at 22-24 weeks of gestation for adverse pregnancy outcomes.	Not specified	Low-risk	Women with uncomplicated pregnancies
Waa et al., 2010	To assess the value of umbilical and middle cerebral artery doppler ultrasound values in predicting foetal outcome in high and low-risk pregnancies.	Not specified	Low and high-risk	Women undergoing routine antenatal (any risk profile).
Yelikar et al., 2013	To study the efficacy of fetal Doppler and Non-Stress Test (NST) in predicting fetal compromise in preeclampsia and growth-restricted fetuses.	Not specified	High-risk	Preeclampsia and growth-restricted fetuses
Zarean et al., 2018	To assess the diagnostic value of UtA-PI in the prediction of the adverse perinatal outcome at 30–34 week's gestation.	Not specified	Low-risk	Women that had uncomplicated pregnancies

[a]FGR: fetal growth restriction; LBW: low birth weight; NICU: neonatal intensive care unit. High risk: pregnancies with any underlying condition that threatens the health or life of the mother or her foetus.
Any risk profile: unselected pregnancies (pregnancies undergoing routine antenatal). Low risk: Uncomplicated pregnancies or healthy pregnant women

For peer review only

Appendix S4. Risk of bias assessment results of the 30 studies included in the analysis

First Author: Abdallah et al., 2018 **ID:** 68614233

Potential Bias	Items to be considered for assessment of potential opportunity for bias	Yes	Partly	No	Unsure	NA*
Study population /sample selection	Inclusion and exclusion criteria are adequately described [including explicit diagnostic criteria, start/finish date of recruitment]	x				
	Baseline study sample [i.e., individuals entering the study and their key characteristics and sampling frame are adequately described]	x				
	Study sample represents population of interest on key characteristics, sufficient to limit potential bias to results	Low risk of bias				
Study attrition	Response rate (i.e., proportion of study sample completing the study and providing outcome data) is adequate				x	
	Participants lost to follow-up are adequately described for key characteristics				x	
	Statement as to the possible effect on the results from missing data				x	
	Loss to follow-up is not associated with key characteristics	Moderate risk of bias				
Prognostic factor measurement	Clear definition of the prognostic factors measured is provided (e.g imaging modality method, measurement, and timing described).	x				
	Specified instrument and personnel for measurement of predictive factors	x				
	Continuous variables are reported or appropriate (i.e. not data- dependent) cut-off points are used and specified a priori	x				
	Blinding: were estimators of risk factor status and of outcomes blinded?			x		
	The prognostic factor(s) of interest is (are) adequately measured in study participants to sufficiently limit potential bias	Low risk of bias				
Outcome measurement	Is the outcome(s) clearly defined?			x		
	The outcome measure and method used are adequately valid and reliable to limit misclassification bias	Moderate risk of bias				
Study confounding	Do the authors address potential confounders?	x				
	Important potential confounders are appropriately accounted for, limiting potential bias with respect to the prognostic factor of interest.	Low risk of bias				
Analysis and reporting	There is sufficient presentation of data to assess the adequacy of the analysis strategy and there is no selective reporting		x			
	The statistical analysis is appropriate for the study design, limiting potential for the presentation of invalid results	Moderate risk of bias				

NA*: not applicable. Note: The above table was adapted from: Hayden et al., 2013.

Overall opinion of study quality= Moderate risk of bias

For peer review only

First Author: Agbaje et al., 2018 **ID:** 6377433

Potential Bias	Items to be considered for assessment of potential opportunity for bias	Yes	Partly	No	Unsure	NA*
Study population /sample selection	Inclusion and exclusion criteria are adequately described [including explicit diagnostic criteria, start/finish date of recruitment]	x				
	Baseline study sample [i.e. individuals entering the study and their key characteristics and sampling frame are adequately described]		x			
	Study sample represents population of interest on key characteristics, sufficient to limit potential bias to results	Moderate risk of bias				
Study attrition	Response rate (i.e., proportion of study sample completing the study and providing outcome data) is adequate					x
	Participants lost to follow-up are adequately described for key characteristics					x
	Statement as to the possible effect on the results from missing data					x
	Loss to follow-up is not associated with key characteristics	Low risk of bias				
Prognostic factor measurement	Clear definition of the prognostic factors measured is provided (e.g. imaging modality method, measurement, and timing described).	x				
	Specified instrument and personnel for measurement of predictive factors	x				
	Continuous variables are reported or appropriate (i.e. not data- dependent) cut-off points are used and specified a priori	x				
	Blinding: were estimators of risk factor status and of outcomes blinded?		x			
	The prognostic factor(s) of interest is (are) adequately measured in study participants to sufficiently limit potential bias	Low risk of bias				
Outcome measurement	Is the outcome(s) clearly defined?		x			
	The outcome measure and method used are adequately valid and reliable to limit misclassification bias	Moderate risk of bias				
Study confounding	Do the authors address potential confounders?		x			
	Important potential confounders are appropriately accounted for, limiting potential bias with respect to the prognostic factor of interest.	Moderate risk of bias				
Analysis and reporting	There is sufficient presentation of data to assess the adequacy of the analysis strategy and there is no selective reporting		x			
	The statistical analysis is appropriate for the study design, limiting potential for the presentation of invalid results	Moderate risk of bias				

NA*: not applicable. Note: The above table was adapted from: Hayden et al., 2013.

Overall opinion of study quality= Moderate risk of bias

First Author: Alanwar et al., 2018 **ID:** 6377464

Potential Bias	Items to be considered for assessment of potential opportunity for bias	Yes	Partly	No	Unsure	NA*
Study population /sample selection	Inclusion and exclusion criteria are adequately described [including explicit diagnostic criteria, start/finish date of recruitment]	x				
	Baseline study sample [i.e. individuals entering the study and their key characteristics and sampling frame are adequately described]	x				
	Study sample represents population of interest on key characteristics, sufficient to limit potential bias to results	Low risk of bias				
Study attrition	Response rate (i.e., proportion of study sample completing the study and providing outcome data) is adequate					x
	Participants lost to follow-up are adequately described for key characteristics					x
	Statement as to the possible effect on the results from missing data					x
	Loss to follow-up is not associated with key characteristics	Low risk of bias				
Prognostic factor measurement	Clear definition of the prognostic factors measured is provided (e.g. imaging modality method, measurement, and timing described).	x				
	Specified instrument and personnel for measurement of predictive factors	x				
	Continuous variables are reported or appropriate (i.e. not data- dependent) cut-off points are used and specified a priori	x				
	Blinding: were estimators of risk factor status and of outcomes blinded?				x	
	The prognostic factor(s) of interest is (are) adequately measured in study participants to sufficiently limit potential bias	Low risk of bias				
Outcome measurement	Is the outcome(s) clearly defined?	x				
	The outcome measure and method used are adequately valid and reliable to limit misclassification bias	Low risk of bias				
Study confounding	Do the authors address potential confounders?	x				
	Important potential confounders are appropriately accounted for, limiting potential bias with respect to the prognostic factor of interest.	Low risk of bias				
Analysis and reporting	There is sufficient presentation of data to assess the adequacy of the analysis strategy and there is no selective reporting	x				
	The statistical analysis is appropriate for the study design, limiting potential for the presentation of invalid results	Low risk of bias				

NA*: not applicable. Note: The above table was adapted from: Hayden et al., 2013.

Overall opinion of study quality= Low risk of bias

First Author: Allam et al., 2013 **ID:** 6377480

**Potential Bias**	**Items to be considered for assessment of potential opportunity for bias**	**Yes**	**Partly**	**No**	**Unsure**	**NA***
Study population /sample selection	Inclusion and exclusion criteria are adequately described [including explicit diagnostic criteria, start/finish date of recruitment]		x			
	Baseline study sample [i.e. individuals entering the study and their key characteristics and sampling frame are adequately described]	x				
	Study sample represents population of interest on key characteristics, sufficient to limit potential bias to results	Low risk of bias				
Study attrition	Response rate (i.e., proportion of study sample completing the study and providing outcome data) is adequate	x				
	Participants lost to follow-up are adequately described for key characteristics				x	
	Statement as to the possible effect on the results from missing data					x
	Loss to follow-up is not associated with key characteristics	Low risk of bias				
Prognostic factor measurement	Clear definition of the prognostic factors measured is provided (e.g. imaging modality method, measurement, and timing described).	x				
	Specified instrument and personnel for measurement of predictive factors	x				
	Continuous variables are reported or appropriate (i.e. not data- dependent) cut-off points are used and specified a priori	x				
	Blinding: were estimators of risk factor status and of outcomes blinded?				x	
	The prognostic factor(s) of interest is (are) adequately measured in study participants to sufficiently limit potential bias	Low risk of bias				
Outcome measurement	Is the outcome(s) clearly defined?	x				
	The outcome measure and method used are adequately valid and reliable to limit misclassification bias	Low risk of bias				
Study confounding	Do the authors address potential confounders?		x			
	Important potential confounders are appropriately accounted for, limiting potential bias with respect to the prognostic factor of interest.	Moderate risk of bias				
Analysis and reporting	There is sufficient presentation of data to assess the adequacy of the analysis strategy and there is no selective reporting		x			
	The statistical analysis is appropriate for the study design, limiting potential for the presentation of invalid results	Moderate risk of bias				
NA*: not applicable. Note: The above table was adapted from: Hayden et al., 2013.						
**Overall opinion of study quality= Low risk of bias**						

For peer review only

First Author: Anshul et al., 2010 **ID:** 6377837

Potential Bias	Items to be considered for assessment of potential opportunity for bias	Yes	Partly	No	Unsure	NA*
Study population /sample selection	Inclusion and exclusion criteria are adequately described [including explicit diagnostic criteria, start/finish date of recruitment]	x				
	Baseline study sample [i.e. individuals entering the study and their key characteristics and sampling frame are adequately described]	x				
	Study sample represents population of interest on key characteristics, sufficient to limit potential bias to results	Low risk of bias				
Study attrition	Response rate (i.e., proportion of study sample completing the study and providing outcome data) is adequate				x	
	Participants lost to follow-up are adequately described for key characteristics				x	
	Statement as to the possible effect on the results from missing data				x	
	Loss to follow-up is not associated with key characteristics	High risk of bias				
Prognostic factor measurement	Clear definition of the prognostic factors measured is provided (e.g. imaging modality method, measurement, and timing described).		x			
	Specified instrument and personnel for measurement of predictive factors			x		
	Continuous variables are reported or appropriate (i.e. not data- dependent) cut-off points are used and specified a priori	x				
	Blinding: were estimators of risk factor status and of outcomes blinded?					x
	The prognostic factor(s) of interest is (are) adequately measured in study participants to sufficiently limit potential bias	Moderate risk of bias				
Outcome measurement	Is the outcome(s) clearly defined?			x		
	The outcome measure and method used are adequately valid and reliable to limit misclassification bias	High risk of bias				
Study confounding	Do the authors address potential confounders?	x				
	Important potential confounders are appropriately accounted for, limiting potential bias with respect to the prognostic factor of interest.	Low risk of bias				
Analysis and reporting	There is sufficient presentation of data to assess the adequacy of the analysis strategy and there is no selective reporting			x		
	The statistical analysis is appropriate for the study design, limiting potential for the presentation of invalid results	High risk of bias				
NA*: not applicable. Note: The above table was adapted from: Hayden et al., 2013.						
**Overall opinion of study quality= High risk of bias**						

For peer review only

First Author: Bano et al., 2010 **ID:** 74903018

**Potential Bias**	**Items to be considered for assessment of potential opportunity for bias**	**Yes**	**Partly**	**No**	**Unsure**	**NA***
Study population /sample selection	Inclusion and exclusion criteria are adequately described [including explicit diagnostic criteria, start/finish date of recruitment]		x			
	Baseline study sample [i.e. individuals entering the study and their key characteristics and sampling frame are adequately described]			x		
	Study sample represents population of interest on key characteristics, sufficient to limit potential bias to results	Moderate risk of bias				
Study attrition	Response rate (i.e., proportion of study sample completing the study and providing outcome data) is adequate					x
	Participants lost to follow-up are adequately described for key characteristics					x
	Statement as to the possible effect on the results from missing data					x
	Loss to follow-up is not associated with key characteristics	Low risk of bias				
Prognostic factor measurement	Clear definition of the prognostic factors measured is provided (e.g. imaging modality method, measurement, and timing described).	x				
	Specified instrument and personnel for measurement of predictive factors			x		
	Continuous variables are reported or appropriate (i.e. not data- dependent) cut-off points are used and specified a priori	x				
	Blinding: were estimators of risk factor status and of outcomes blinded?			x		
	The prognostic factor(s) of interest is (are) adequately measured in study participants to sufficiently limit potential bias	Moderate risk of bias				
Outcome measurement	Is the outcome(s) clearly defined?			x		
	The outcome measure and method used are adequately valid and reliable to limit misclassification bias	High risk of bias				
Study confounding	Do the authors address potential confounders?			x		
	Important potential confounders are appropriately accounted for, limiting potential bias with respect to the prognostic factor of interest.	High risk of bias				
Analysis and reporting	There is sufficient presentation of data to assess the adequacy of the analysis strategy and there is no selective reporting		x			
	The statistical analysis is appropriate for the study design, limiting potential for the presentation of invalid results	Moderate risk of bias				

NA*: not applicable. Note: The above table was adapted from: Hayden et al., 2013.

Overall opinion of study quality= high risk of bias

First Author: Dhand et al., 2011 **ID:** 6379383

Potential Bias	Items to be considered for assessment of potential opportunity for bias	Yes	Partly	No	Unsure	NA*
Study population /sample selection	Inclusion and exclusion criteria are adequately described [including explicit diagnostic criteria, start/finish date of recruitment]	x				
	Baseline study sample [i.e. individuals entering the study and their key characteristics and sampling frame are adequately described]	x				
	Study sample represents population of interest on key characteristics, sufficient to limit potential bias to results	Low risk of bias				
Study attrition	Response rate (i.e., proportion of study sample completing the study and providing outcome data) is adequate				x	
	Participants lost to follow-up are adequately described for key characteristics				x	
	Statement as to the possible effect on the results from missing data				x	
	Loss to follow-up is not associated with key characteristics	Moderate risk of bias				
Prognostic factor measurement	Clear definition of the prognostic factors measured is provided (e.g. imaging modality method, measurement, and timing described).		x			
	Specified instrument and personnel for measurement of predictive factors		x			
	Continuous variables are reported or appropriate (i.e. not data- dependent) cut-off points are used and specified a priori		x			
	Blinding: were estimators of risk factor status and of outcomes blinded?				x	
	The prognostic factor(s) of interest is (are) adequately measured in study participants to sufficiently limit potential bias	High risk of bias				
Outcome measurement	Is the outcome(s) clearly defined?			x		
	The outcome measure and method used are adequately valid and reliable to limit misclassification bias	High risk of bias				
Study confounding	Do the authors address potential confounders?	x				
	Important potential confounders are appropriately accounted for, limiting potential bias with respect to the prognostic factor of interest.	Low risk of bias				
Analysis and reporting	There is sufficient presentation of data to assess the adequacy of the analysis strategy and there is no selective reporting			x		
	The statistical analysis is appropriate for the study design, limiting potential for the presentation of invalid results	High risk of bias				

NA*: not applicable. Note: The above table was adapted from: Hayden et al., 2013.

Overall opinion of study quality= High risk of bias

First Author: Dorman et al., 2002 **ID:** 6377862

Potential Bias	Items to be considered for assessment of potential opportunity for bias	Yes	Partly	No	Unsure	NA*
Study population /sample selection	Inclusion and exclusion criteria are adequately described [including explicit diagnostic criteria, start/finish date of recruitment]	x				
	Baseline study sample [i.e. individuals entering the study and their key characteristics and sampling frame are adequately described]	x				
	Study sample represents population of interest on key characteristics, sufficient to limit potential bias to results	Low risk of bias				
Study attrition	Response rate (i.e., proportion of study sample completing the study and providing outcome data) is adequate	x				
	Participants lost to follow-up are adequately described for key characteristics	x				
	Statement as to the possible effect on the results from missing data	x				
	Loss to follow-up is not associated with key characteristics	Low risk of bias				
Prognostic factor measurement	Clear definition of the prognostic factors measured is provided (e.g. imaging modality method, measurement, and timing described).	x				
	Specified instrument and personnel for measurement of predictive factors	x				
	Continuous variables are reported or appropriate (i.e. not data- dependent) cut-off points are used and specified a priori	x				
	Blinding: were estimators of risk factor status and of outcomes blinded?	x				
	The prognostic factor(s) of interest is (are) adequately measured in study participants to sufficiently limit potential bias	Low risk of bias				
Outcome measurement	Is the outcome(s) clearly defined?	x				
	The outcome measure and method used are adequately valid and reliable to limit misclassification bias	Low risk of bias				
Study confounding	Do the authors address potential confounders?	x				
	Important potential confounders are appropriately accounted for, limiting potential bias with respect to the prognostic factor of interest.	Low risk of bias				
Analysis and reporting	There is sufficient presentation of data to assess the adequacy of the analysis strategy and there is no selective reporting	x				
	The statistical analysis is appropriate for the study design, limiting potential for the presentation of invalid results	Low risk of bias				

NA*: not applicable. Note: The above table was adapted from: Hayden et al., 2013.

Overall opinion of study quality= Low risk of bias

First Author: Ebrashy et al., 2005 **ID:** 6377887

Potential Bias	Items to be considered for assessment of potential opportunity for bias	Yes	Partly	No	Unsure	NA*
Study population /sample selection	Inclusion and exclusion criteria are adequately described [including explicit diagnostic criteria, start/finish date of recruitment]	x				
	Baseline study sample [i.e. individuals entering the study and their key characteristics and sampling frame are adequately described]	x				
	Study sample represents population of interest on key characteristics, sufficient to limit potential bias to results	Low risk of bias				
Study attrition	Response rate (i.e., proportion of study sample completing the study and providing outcome data) is adequate	x				
	Participants lost to follow-up are adequately described for key characteristics	x				
	Statement as to the possible effect on the results from missing data					x
	Loss to follow-up is not associated with key characteristics	Low risk of bias				
Prognostic factor measurement	Clear definition of the prognostic factors measured is provided (e.g. imaging modality method, measurement, and timing described).	x				
	Specified instrument and personnel for measurement of predictive factors	x				
	Continuous variables are reported or appropriate (i.e. not data- dependent) cut-off points are used and specified a priori	x				
	Blinding: were estimators of risk factor status and of outcomes blinded?	x				
	The prognostic factor(s) of interest is (are) adequately measured in study participants to sufficiently limit potential bias	Low risk of bias				
Outcome measurement	Is the outcome(s) clearly defined?	x				
	The outcome measure and method used are adequately valid and reliable to limit misclassification bias	Low risk of bias				
Study confounding	Do the authors address potential confounders?	x				
	Important potential confounders are appropriately accounted for, limiting potential bias with respect to the prognostic factor of interest.	Low risk of bias				
Analysis and reporting	There is sufficient presentation of data to assess the adequacy of the analysis strategy and there is no selective reporting		x			
	The statistical analysis is appropriate for the study design, limiting potential for the presentation of invalid results	Moderate risk of bias				

NA*: not applicable. Note: The above table was adapted from: Hayden et al., 2013.

Overall opinion of study quality= Low risk of bias

First Author: Geerts et al., 2007 **ID:** 6378017

**Potential Bias**	**Items to be considered for assessment of potential opportunity for bias**	**Yes**	**Partly**	**No**	**Unsure**	**NA***
Study population /sample selection	Inclusion and exclusion criteria are adequately described [including explicit diagnostic criteria, start/finish date of recruitment]	x				
	Baseline study sample [i.e. individuals entering the study and their key characteristics and sampling frame are adequately described]	x				
	Study sample represents population of interest on key characteristics, sufficient to limit potential bias to results	Low risk of bias				
Study attrition	Response rate (i.e., proportion of study sample completing the study and providing outcome data) is adequate	x				
	Participants lost to follow-up are adequately described for key characteristics					x
	Statement as to the possible effect on the results from missing data					x
	Loss to follow-up is not associated with key characteristics	Low risk of bias				
Prognostic factor measurement	Clear definition of the prognostic factors measured is provided (e.g. imaging modality method, measurement, and timing described).		x			
	Specified instrument and personnel for measurement of predictive factors	x				
	Continuous variables are reported or appropriate (i.e. not data- dependent) cut-off points are used and specified a priori	x				
	Blinding: were estimators of risk factor status and of outcomes blinded?	x				
	The prognostic factor(s) of interest is (are) adequately measured in study participants to sufficiently limit potential bias	Low risk of bias				
Outcome measurement	Is the outcome(s) clearly defined?		x			
	The outcome measure and method used are adequately valid and reliable to limit misclassification bias	Moderate risk of bias				
Study confounding	Do the authors address potential confounders?		x			
	Important potential confounders are appropriately accounted for, limiting potential bias with respect to the prognostic factor of interest.	Moderate risk of bias				
Analysis and reporting	There is sufficient presentation of data to assess the adequacy of the analysis strategy and there is no selective reporting	x				
	The statistical analysis is appropriate for the study design, limiting potential for the presentation of invalid results	Low risk of bias				
NA*: not applicable. Note: The above table was adapted from: Hayden et al., 2013.						
**Overall opinion of study quality= Low risk of bias**						

First Author: Khanduri et al., 2013 **ID:** 6378321

Potential Bias	Items to be considered for assessment of potential opportunity for bias	Yes	Partly	No	Unsure	NA*
Study population /sample selection	Inclusion and exclusion criteria are adequately described [including explicit diagnostic criteria, start/finish date of recruitment]	x				
	Baseline study sample [i.e. individuals entering the study and their key characteristics and sampling frame are adequately described]	x				
	Study sample represents population of interest on key characteristics, sufficient to limit potential bias to results	Low risk of bias				
Study attrition	Response rate (i.e., proportion of study sample completing the study and providing outcome data) is adequate	x				
	Participants lost to follow-up are adequately described for key characteristics	x				
	Statement as to the possible effect on the results from missing data					x
	Loss to follow-up is not associated with key characteristics	Low risk of bias				
Prognostic factor measurement	Clear definition of the prognostic factors measured is provided (e.g. imaging modality method, measurement, and timing described).	x				
	Specified instrument and personnel for measurement of predictive factors	x				
	Continuous variables are reported or appropriate (i.e. not data- dependent) cut-off points are used and specified a priori	x				
	Blinding: were estimators of risk factor status and of outcomes blinded?		x			
	The prognostic factor(s) of interest is (are) adequately measured in study participants to sufficiently limit potential bias	Low risk of bas				
Outcome measurement	Is the outcome(s) clearly defined?		x			
	The outcome measure and method used are adequately valid and reliable to limit misclassification bias	Moderate risk of bas				
Study confounding	Do the authors address potential confounders?	x				
	Important potential confounders are appropriately accounted for, limiting potential bias with respect to the prognostic factor of interest.	Low risk of bias				
Analysis and reporting	There is sufficient presentation of data to assess the adequacy of the analysis strategy and there is no selective reporting		x			
	The statistical analysis is appropriate for the study design, limiting potential for the presentation of invalid results	Moderate risk of bias				

NA*: not applicable. Note: The above table was adapted from: Hayden et al., 2013.

Overall opinion of study quality= Low risk of bias

First Author: Kumari et al., 2019 **ID:** 68614385

**Potential Bias**	**Items to be considered for assessment of potential opportunity for bias**	**Yes**	**Partly**	**No**	**Unsure**	**NA***
Study population /sample selection	Inclusion and exclusion criteria are adequately described [including explicit diagnostic criteria, start/finish date of recruitment]		x			
	Baseline study sample [i.e. individuals entering the study and their key characteristics and sampling frame are adequately described]		x			
	Study sample represents population of interest on key characteristics, sufficient to limit potential bias to results	Moderate risk of bias				
Study attrition	Response rate (i.e., proportion of study sample completing the study and providing outcome data) is adequate				x	
	Participants lost to follow-up are adequately described for key characteristics				x	
	Statement as to the possible effect on the results from missing data				x	
	Loss to follow-up is not associated with key characteristics	Moderate risk of bias				
Prognostic factor measurement	Clear definition of the prognostic factors measured is provided (e.g. imaging modality method, measurement, and timing described).	x				
	Specified instrument and personnel for measurement of predictive factors	x				
	Continuous variables are reported or appropriate (i.e. not data- dependent) cut-off points are used and specified a priori	x				
	Blinding: were estimators of risk factor status and of outcomes blinded?			x		
	The prognostic factor(s) of interest is (are) adequately measured in study participants to sufficiently limit potential bias	Low risk of bias				
Outcome measurement	Is the outcome(s) clearly defined?		x			
	The outcome measure and method used are adequately valid and reliable to limit misclassification bias	Moderate risk of bias				
Study confounding	Do the authors address potential confounders?	x				
	Important potential confounders are appropriately accounted for, limiting potential bias with respect to the prognostic factor of interest.	Low risk of bias				
Analysis and reporting	There is sufficient presentation of data to assess the adequacy of the analysis strategy and there is no selective reporting	x				
	The statistical analysis is appropriate for the study design, limiting potential for the presentation of invalid results	Low risk of bias				

NA*: not applicable. Note: The above table was adapted from: Hayden et al., 2013.

Overall opinion of study quality= Moderate risk of bias

First Author: Lakhkar et al., 2006 **ID:** 74903014

**Potential Bias**	**Items to be considered for assessment of potential opportunity for bias**	**Yes**	**Partly**	**No**	**Unsure**	**NA***
Study population /sample selection	Inclusion and exclusion criteria are adequately described [including explicit diagnostic criteria, start/finish date of recruitment]		x			
	Baseline study sample [i.e. individuals entering the study and their key characteristics and sampling frame are adequately described]	x				
	Study sample represents population of interest on key characteristics, sufficient to limit potential bias to results	Moderate risk of bias				
Study attrition	Response rate (i.e., proportion of study sample completing the study and providing outcome data) is adequate					x
	Participants lost to follow-up are adequately described for key characteristics					x
	Statement as to the possible effect on the results from missing data					x
	Loss to follow-up is not associated with key characteristics	Low risk of bias				
Prognostic factor measurement	Clear definition of the prognostic factors measured is provided (e.g. imaging modality method, measurement, and timing described).	x				
	Specified instrument and personnel for measurement of predictive factors		x			
	Continuous variables are reported or appropriate (i.e. not data- dependent) cut-off points are used and specified a priori	x				
	Blinding: were estimators of risk factor status and of outcomes blinded?			x		
	The prognostic factor(s) of interest is (are) adequately measured in study participants to sufficiently limit potential bias	Moderate risk of bias				
Outcome measurement	Is the outcome(s) clearly defined?	x				
	The outcome measure and method used are adequately valid and reliable to limit misclassification bias	Low risk of bias				
Study confounding	Do the authors address potential confounders?	x				
	Important potential confounders are appropriately accounted for, limiting potential bias with respect to the prognostic factor of interest.	Low risk of bias				
Analysis and reporting	There is sufficient presentation of data to assess the adequacy of the analysis strategy and there is no selective reporting		x			
	The statistical analysis is appropriate for the study design, limiting potential for the presentation of invalid results	Moderate risk of bias				
NA*: not applicable. Note: The above table was adapted from: Hayden et al., 2013.						
**Overall opinion of study quality= Moderate risk of bias**						

For peer review only

First Author: Lakshmi et al., 2013 **ID:** 6378401

Potential Bias	Items to be considered for assessment of potential opportunity for bias	Yes	Partly	No	Unsure	NA*
Study population /sample selection	Inclusion and exclusion criteria are adequately described [including explicit diagnostic criteria, start/finish date of recruitment]	x				
	Baseline study sample [i.e. individuals entering the study and their key characteristics and sampling frame are adequately described]	x				
	Study sample represents population of interest on key characteristics, sufficient to limit potential bias to results	Low risk of bias				
Study attrition	Response rate (i.e., proportion of study sample completing the study and providing outcome data) is adequate	x				
	Participants lost to follow-up are adequately described for key characteristics	x				
	Statement as to the possible effect on the results from missing data			x		
	Loss to follow-up is not associated with key characteristics	Low risk of bias				
Prognostic factor measurement	Clear definition of the prognostic factors measured is provided (e.g. imaging modality method, measurement, and timing described).	x				
	Specified instrument and personnel for measurement of predictive factors	x				
	Continuous variables are reported or appropriate (i.e. not data- dependent) cut-off points are used and specified a priori	x				
	Blinding: were estimators of risk factor status and of outcomes blinded?	x				
	The prognostic factor(s) of interest is (are) adequately measured in study participants to sufficiently limit potential bias	Low risk of bias				
Outcome measurement	Is the outcome(s) clearly defined?	x				
	The outcome measure and method used are adequately valid and reliable to limit misclassification bias	Low risk of bias				
Study confounding	Do the authors address potential confounders?	x				
	Important potential confounders are appropriately accounted for, limiting potential bias with respect to the prognostic factor of interest.	Low risk of bias				
Analysis and reporting	There is sufficient presentation of data to assess the adequacy of the analysis strategy and there is no selective reporting		x			
	The statistical analysis is appropriate for the study design, limiting potential for the presentation of invalid results	Moderate risk of bias				

NA*: not applicable. Note: The above table was adapted from: Hayden et al., 2013.

Overall opinion of study quality= Low risk of bias

First Author: Malik et al., 2013 **ID:** 6378519

Potential Bias	Items to be considered for assessment of potential opportunity for bias	Yes	Partly	No	Unsure	NA*
Study population /sample selection	Inclusion and exclusion criteria are adequately described [including explicit diagnostic criteria, start/finish date of recruitment]	x				
	Baseline study sample [i.e. individuals entering the study and their key characteristics and sampling frame are adequately described]	x				
	Study sample represents population of interest on key characteristics, sufficient to limit potential bias to results	Low risk of bias				
Study attrition	Response rate (i.e., proportion of study sample completing the study and providing outcome data) is adequate	x				
	Participants lost to follow-up are adequately described for key characteristics			x		
	Statement as to the possible effect on the results from missing data				x	
	Loss to follow-up is not associated with key characteristics	High risk of bias				
Prognostic factor measurement	Clear definition of the prognostic factors measured is provided (e.g. imaging modality method, measurement, and timing described).			x		
	Specified instrument and personnel for measurement of predictive factors		x			
	Continuous variables are reported or appropriate (i.e. not data- dependent) cut-off points are used and specified a priori				x	
	Blinding: were estimators of risk factor status and of outcomes blinded?				x	
	The prognostic factor(s) of interest is (are) adequately measured in study participants to sufficiently limit potential bias	High risk of bias				
Outcome measurement	Is the outcome(s) clearly defined?		x			
	The outcome measure and method used are adequately valid and reliable to limit misclassification bias	Moderate risk of bias				
Study confounding	Do the authors address potential confounders?		x			
	Important potential confounders are appropriately accounted for, limiting potential bias with respect to the prognostic factor of interest.	Moderate risk of bias				
Analysis and reporting	There is sufficient presentation of data to assess the adequacy of the analysis strategy and there is no selective reporting		x			
	The statistical analysis is appropriate for the study design, limiting potential for the presentation of invalid results	Moderate risk of bias				
NA*: not applicable. Note: The above table was adapted from: Hayden et al., 2013.						
**Overall opinion of study quality= High risk of bias**						

For peer review only

First Author: Masihi et al., 2019 **ID:** 68614415

Potential Bias	Items to be considered for assessment of potential opportunity for bias	Yes	Partly	No	Unsure	NA*
Study population /sample selection	Inclusion and exclusion criteria are adequately described [including explicit diagnostic criteria, start/finish date of recruitment]	x				
	Baseline study sample [i.e. individuals entering the study and their key characteristics and sampling frame are adequately described]	x				
	Study sample represents population of interest on key characteristics, sufficient to limit potential bias to results	Low risk of bias				
Study attrition	Response rate (i.e., proportion of study sample completing the study and providing outcome data) is adequate			x		
	Participants lost to follow-up are adequately described for key characteristics			x		
	Statement as to the possible effect on the results from missing data			x		
	Loss to follow-up is not associated with key characteristics	Moderate risk of bias				
Prognostic factor measurement	Clear definition of the prognostic factors measured is provided (e.g. imaging modality method, measurement, and timing described).	x				
	Specified instrument and personnel for measurement of predictive factors			x		
	Continuous variables are reported or appropriate (i.e. not data- dependent) cut-off points are used and specified a priori	x				
	Blinding: were estimators of risk factor status and of outcomes blinded?			x		
	The prognostic factor(s) of interest is (are) adequately measured in study participants to sufficiently limit potential bias	Moderate risk of bias				
Outcome measurement	Is the outcome(s) clearly defined?	x				
	The outcome measure and method used are adequately valid and reliable to limit misclassification bias	Low risk of bias				
Study confounding	Do the authors address potential confounders?	x				
	Important potential confounders are appropriately accounted for, limiting potential bias with respect to the prognostic factor of interest.	Low risk of bias				
Analysis and reporting	There is sufficient presentation of data to assess the adequacy of the analysis strategy and there is no selective reporting	x				
	The statistical analysis is appropriate for the study design, limiting potential for the presentation of invalid results	Low risk of bias				

NA*: not applicable. Note: The above table was adapted from: Hayden et al., 2013.

Overall opinion of study quality= Low risk of bias

For peer review only

First Author: Mullick et al., 1993 **ID:** 6378675

**Potential Bias**	**Items to be considered for assessment of potential opportunity for bias**	**Yes**	**Partly**	**No**	**Unsure**	**NA***
Study population /sample selection	Inclusion and exclusion criteria are adequately described [including explicit diagnostic criteria, start/finish date of recruitment]	x				
	Baseline study sample [i.e. individuals entering the study and their key characteristics and sampling frame are adequately described]		x			
	Study sample represents population of interest on key characteristics, sufficient to limit potential bias to results	Low risk of bias				
Study attrition	Response rate (i.e., proportion of study sample completing the study and providing outcome data) is adequate	x				
	Participants lost to follow-up are adequately described for key characteristics	x				
	Statement as to the possible effect on the results from missing data					x
	Loss to follow-up is not associated with key characteristics	Low risk of bias				
Prognostic factor measurement	Clear definition of the prognostic factors measured is provided (e.g. imaging modality method, measurement, and timing described).	x				
	Specified instrument and personnel for measurement of predictive factors		x			
	Continuous variables are reported or appropriate (i.e. not data- dependent) cut-off points are used and specified a priori		x			
	Blinding: were estimators of risk factor status and of outcomes blinded?				x	
	The prognostic factor(s) of interest is (are) adequately measured in study participants to sufficiently limit potential bias	Moderate risk of bias				
Outcome measurement	Is the outcome(s) clearly defined?		x			
	The outcome measure and method used are adequately valid and reliable to limit misclassification bias	Moderate risk of bias				
Study confounding	Do the authors address potential confounders?		x			
	Important potential confounders are appropriately accounted for, limiting potential bias with respect to the prognostic factor of interest.	Moderate risk of bias				
Analysis and reporting	There is sufficient presentation of data to assess the adequacy of the analysis strategy and there is no selective reporting		x			
	The statistical analysis is appropriate for the study design, limiting potential for the presentation of invalid results	Moderate risk of bias				

NA*: not applicable. Note: The above table was adapted from: Hayden et al., 2013.

Overall opinion of study quality= Moderate risk of bias

For peer review only

First Author: Nagar et al., 2015 **ID:** 6378692

Potential Bias	Items to be considered for assessment of potential opportunity for bias	Yes	Partly	No	Unsure	NA*
Study population /sample selection	Inclusion and exclusion criteria are adequately described [including explicit diagnostic criteria, start/finish date of recruitment]	x				
	Baseline study sample [i.e. individuals entering the study and their key characteristics and sampling frame are adequately described]		x			
	Study sample represents population of interest on key characteristics, sufficient to limit potential bias to results	Low risk of bias				
Study attrition	Response rate (i.e., proportion of study sample completing the study and providing outcome data) is adequate	x				
	Participants lost to follow-up are adequately described for key characteristics				x	
	Statement as to the possible effect on the results from missing data					x
	Loss to follow-up is not associated with key characteristics	Low risk of bias				
Prognostic factor measurement	Clear definition of the prognostic factors measured is provided (e.g. imaging modality method, measurement, and timing described).	x				
	Specified instrument and personnel for measurement of predictive factors			x		
	Continuous variables are reported or appropriate (i.e. not data- dependent) cut-off points are used and specified a priori	x				
	Blinding: were estimators of risk factor status and of outcomes blinded?			x		
	The prognostic factor(s) of interest is (are) adequately measured in study participants to sufficiently limit potential bias	Moderate risk of bias				
Outcome measurement	Is the outcome(s) clearly defined?		x			
	The outcome measure and method used are adequately valid and reliable to limit misclassification bias	Moderate risk of bias				
Study confounding	Do the authors address potential confounders?	x				
	Important potential confounders are appropriately accounted for, limiting potential bias with respect to the prognostic factor of interest.	Low risk of bias				
Analysis and reporting	There is sufficient presentation of data to assess the adequacy of the analysis strategy and there is no selective reporting		x			
	The statistical analysis is appropriate for the study design, limiting potential for the presentation of invalid results	Moderate risk of bias				

NA*: not applicable. Note: The above table was adapted from: Hayden et al., 2013.

Overall opinion of study quality= Moderate risk of bias

First Author: Najam et al., 2016 **ID:** 6378705

**Potential Bias**	**Items to be considered for assessment of potential opportunity for bias**	**Yes**	**Partly**	**No**	**Unsure**	**NA***
Study population /sample selection	Inclusion and exclusion criteria are adequately described [including explicit diagnostic criteria, start/finish date of recruitment]			x		
	Baseline study sample [i.e. individuals entering the study and their key characteristics and sampling frame are adequately described]			x		
	Study sample represents population of interest on key characteristics, sufficient to limit potential bias to results	High risk of bias				
Study attrition	Response rate (i.e., proportion of study sample completing the study and providing outcome data) is adequate		x			
	Participants lost to follow-up are adequately described for key characteristics		x			
	Statement as to the possible effect on the results from missing data			x		
	Loss to follow-up is not associated with key characteristics	High risk of bias				
Prognostic factor measurement	Clear definition of the prognostic factors measured is provided (e.g. imaging modality method, measurement, and timing described).	x				
	Specified instrument and personnel for measurement of predictive factors			x		
	Continuous variables are reported or appropriate (i.e. not data- dependent) cut-off points are used and specified a priori	x				
	Blinding: were estimators of risk factor status and of outcomes blinded?			x		
	The prognostic factor(s) of interest is (are) adequately measured in study participants to sufficiently limit potential bias	Moderate risk of bias				
Outcome measurement	Is the outcome(s) clearly defined?			x		
	The outcome measure and method used are adequately valid and reliable to limit misclassification bias	High risk of bias				
Study confounding	Do the authors address potential confounders?				x	
	Important potential confounders are appropriately accounted for, limiting potential bias with respect to the prognostic factor of interest.	High risk of bias				
Analysis and reporting	There is sufficient presentation of data to assess the adequacy of the analysis strategy and there is no selective reporting			x		
	The statistical analysis is appropriate for the study design, limiting potential for the presentation of invalid results	High risk of bias				

NA*: not applicable. Note: The above table was adapted from: Hayden et al., 2013.

Overall opinion of study quality= High risk of bias

First Author: Nouh et al., 2011 **ID:** 6378752

**Potential Bias**	**Items to be considered for assessment of potential opportunity for bias**	**Yes**	**Partly**	**No**	**Unsure**	**NA***
Study population /sample selection	Inclusion and exclusion criteria are adequately described [including explicit diagnostic criteria, start/finish date of recruitment]	x				
	Baseline study sample [i.e. individuals entering the study and their key characteristics and sampling frame are adequately described]	x				
	Study sample represents population of interest on key characteristics, sufficient to limit potential bias to results	Low risk of bias				
Study attrition	Response rate (i.e., proportion of study sample completing the study and providing outcome data) is adequate	x				
	Participants lost to follow-up are adequately described for key characteristics					x
	Statement as to the possible effect on the results from missing data				x	
	Loss to follow-up is not associated with key characteristics	Low risk of bias				
Prognostic factor measurement	Clear definition of the prognostic factors measured is provided (e.g. imaging modality method, measurement, and timing described).	x				
	Specified instrument and personnel for measurement of predictive factors	x				
	Continuous variables are reported or appropriate (i.e. not data- dependent) cut-off points are used and specified a priori	x				
	Blinding: were estimators of risk factor status and of outcomes blinded?				x	
	The prognostic factor(s) of interest is (are) adequately measured in study participants to sufficiently limit potential bias	Low risk of bias				
Outcome measurement	Is the outcome(s) clearly defined?		x			
	The outcome measure and method used are adequately valid and reliable to limit misclassification bias	Low risk of bias				
Study confounding	Do the authors address potential confounders?	x				
	Important potential confounders are appropriately accounted for, limiting potential bias with respect to the prognostic factor of interest.	Low risk of bias				
Analysis and reporting	There is sufficient presentation of data to assess the adequacy of the analysis strategy and there is no selective reporting		x			
	The statistical analysis is appropriate for the study design, limiting potential for the presentation of invalid results	Moderate risk of bias				
NA*: not applicable. Note: The above table was adapted from: Hayden et al., 2013.						
**Overall opinion of study quality= Low risk of bias**						

First Author: Pares et al., 2008 **ID:** 6378809

Potential Bias	Items to be considered for assessment of potential opportunity for bias	Yes	Partly	No	Unsure	NA*
Study population /sample selection	Inclusion and exclusion criteria are adequately described [including explicit diagnostic criteria, start/finish date of recruitment]	x				
	Baseline study sample [i.e. individuals entering the study and their key characteristics and sampling frame are adequately described]	x				
	Study sample represents population of interest on key characteristics, sufficient to limit potential bias to results	Low risk of bias				
Study attrition	Response rate (i.e., proportion of study sample completing the study and providing outcome data) is adequate	x				
	Participants lost to follow-up are adequately described for key characteristics					x
	Statement as to the possible effect on the results from missing data					x
	Loss to follow-up is not associated with key characteristics	Low risk of bias				
Prognostic factor measurement	Clear definition of the prognostic factors measured is provided (e.g. imaging modality method, measurement, and timing described).	x				
	Specified instrument and personnel for measurement of predictive factors	x				
	Continuous variables are reported or appropriate (i.e. not data- dependent) cut-off points are used and specified a priori	x				
	Blinding: were estimators of risk factor status and of outcomes blinded?				x	
	The prognostic factor(s) of interest is (are) adequately measured in study participants to sufficiently limit potential bias	Moderate risk of bias				
Outcome measurement	Is the outcome(s) clearly defined?	x				
	The outcome measure and method used are adequately valid and reliable to limit misclassification bias	Low risk of bias				
Study confounding	Do the authors address potential confounders?	x				
	Important potential confounders are appropriately accounted for, limiting potential bias with respect to the prognostic factor of interest.	Low risk of bias				
Analysis and reporting	There is sufficient presentation of data to assess the adequacy of the analysis strategy and there is no selective reporting	x				
	The statistical analysis is appropriate for the study design, limiting potential for the presentation of invalid results	Low risk of bias				

NA*: not applicable. Note: The above table was adapted from: Hayden et al., 2013.

Overall opinion of study quality= Low risk of bias

First Author: Pattinson et al., 1991 **ID:** 74903015

Potential Bias	Items to be considered for assessment of potential opportunity for bias	Yes	Partly	No	Unsure	NA*
Study population /sample selection	Inclusion and exclusion criteria are adequately described [including explicit diagnostic criteria, start/finish date of recruitment]	x				
	Baseline study sample [i.e. individuals entering the study and their key characteristics and sampling frame are adequately described]	x				
	Study sample represents population of interest on key characteristics, sufficient to limit potential bias to results	Low risk of bias				
Study attrition	Response rate (i.e., proportion of study sample completing the study and providing outcome data) is adequate					x
	Participants lost to follow-up are adequately described for key characteristics					x
	Statement as to the possible effect on the results from missing data					x
	Loss to follow-up is not associated with key characteristics	Low risk of bias				
Prognostic factor measurement	Clear definition of the prognostic factors measured is provided (e.g. imaging modality method, measurement, and timing described).	x				
	Specified instrument and personnel for measurement of predictive factors		x			
	Continuous variables are reported or appropriate (i.e. not data- dependent) cut-off points are used and specified a priori	x				
	Blinding: were estimators of risk factor status and of outcomes blinded?	x				
	The prognostic factor(s) of interest is (are) adequately measured in study participants to sufficiently limit potential bias	Low risk of bias				
Outcome measurement	Is the outcome(s) clearly defined?		x			
	The outcome measure and method used are adequately valid and reliable to limit misclassification bias	Moderate risk of bias				
Study confounding	Do the authors address potential confounders?	x				
	Important potential confounders are appropriately accounted for, limiting potential bias with respect to the prognostic factor of interest.	Low risk of bias				
Analysis and reporting	There is sufficient presentation of data to assess the adequacy of the analysis strategy and there is no selective reporting		x			
	The statistical analysis is appropriate for the study design, limiting potential for the presentation of invalid results	Moderate risk of bias				

NA*: not applicable. Note: The above table was adapted from: Hayden et al., 2013.

Overall opinion of study quality= Low risk of bias

For peer review only

First Author: Pattinson et al., 1993 **ID:** 6378815

Potential Bias	Items to be considered for assessment of potential opportunity for bias	Yes	Partly	No	Unsure	NA*
Study population /sample selection	Inclusion and exclusion criteria are adequately described [including explicit diagnostic criteria, start/finish date of recruitment]	x				
	Baseline study sample [i.e. individuals entering the study and their key characteristics and sampling frame are adequately described]	x				
	Study sample represents population of interest on key characteristics, sufficient to limit potential bias to results	Low risk of bias				
Study attrition	Response rate (i.e., proportion of study sample completing the study and providing outcome data) is adequate	x				
	Participants lost to follow-up are adequately described for key characteristics	x				
	Statement as to the possible effect on the results from missing data				x	
	Loss to follow-up is not associated with key characteristics	Low risk of bias				
Prognostic factor measurement	Clear definition of the prognostic factors measured is provided (e.g. imaging modality method, measurement, and timing described).	x				
	Specified instrument and personnel for measurement of predictive factors		x			
	Continuous variables are reported or appropriate (i.e. not data- dependent) cut-off points are used and specified a priori	x				
	Blinding: were estimators of risk factor status and of outcomes blinded?				x	
	The prognostic factor(s) of interest is (are) adequately measured in study participants to sufficiently limit potential bias	Moderate risk of bias				
Outcome measurement	Is the outcome(s) clearly defined?	x				
	The outcome measure and method used are adequately valid and reliable to limit misclassification bias	Low risk of bias				
Study confounding	Do the authors address potential confounders?	x				
	Important potential confounders are appropriately accounted for, limiting potential bias with respect to the prognostic factor of interest.	Low risk of bias				
Analysis and reporting	There is sufficient presentation of data to assess the adequacy of the analysis strategy and there is no selective reporting	x				
	The statistical analysis is appropriate for the study design, limiting potential for the presentation of invalid results	Low risk of bias				
NA*: not applicable. Note: The above table was adapted from: Hayden et al., 2013.						
**Overall opinion of study quality= Low risk of bias**						

First Author: Phupong et al., 2003 **ID:** 6378830

**Potential Bias**	**Items to be considered for assessment of potential opportunity for bias**	**Yes**	**Partly**	**No**	**Unsure**	**NA***
Study population /sample selection	Inclusion and exclusion criteria are adequately described [including explicit diagnostic criteria, start/finish date of recruitment]	x				
	Baseline study sample [i.e. individuals entering the study and their key characteristics and sampling frame are adequately described]	x				
	Study sample represents population of interest on key characteristics, sufficient to limit potential bias to results	Low risk of bias				
Study attrition	Response rate (i.e., proportion of study sample completing the study and providing outcome data) is adequate	x				
	Participants lost to follow-up are adequately described for key characteristics	x				
	Statement as to the possible effect on the results from missing data					x
	Loss to follow-up is not associated with key characteristics	Low risk of bias				
Prognostic factor measurement	Clear definition of the prognostic factors measured is provided (e.g. imaging modality method, measurement, and timing described).	x				
	Specified instrument and personnel for measurement of predictive factors	x				
	Continuous variables are reported or appropriate (i.e. not data- dependent) cut-off points are used and specified a priori	x				
	Blinding: were estimators of risk factor status and of outcomes blinded?	x				
	The prognostic factor(s) of interest is (are) adequately measured in study participants to sufficiently limit potential bias	Low risk of bias				
Outcome measurement	Is the outcome(s) clearly defined?	x				
	The outcome measure and method used are adequately valid and reliable to limit misclassification bias	Low risk of bias				
Study confounding	Do the authors address potential confounders?	x				
	Important potential confounders are appropriately accounted for, limiting potential bias with respect to the prognostic factor of interest.	Low risk of bias				
Analysis and reporting	There is sufficient presentation of data to assess the adequacy of the analysis strategy and there is no selective reporting	x				
	The statistical analysis is appropriate for the study design, limiting potential for the presentation of invalid results	Low risk of bias				
NA*: not applicable. Note: The above table was adapted from: Hayden et al., 2013.						
**Overall opinion of study quality= Low risk of bias**						

First Author: Rani et al., 2016 **ID:** 74903020

Potential Bias	Items to be considered for assessment of potential opportunity for bias	Yes	Partly	No	Unsure	NA*
Study population /sample selection	Inclusion and exclusion criteria are adequately described [including explicit diagnostic criteria, start/finish date of recruitment]	x				
	Baseline study sample [i.e. individuals entering the study and their key characteristics and sampling frame are adequately described]		x			
	Study sample represents population of interest on key characteristics, sufficient to limit potential bias to results	Moderate risk of bias				
Study attrition	Response rate (i.e., proportion of study sample completing the study and providing outcome data) is adequate					x
	Participants lost to follow-up are adequately described for key characteristics					x
	Statement as to the possible effect on the results from missing data					x
	Loss to follow-up is not associated with key characteristics	Low risk of bias				
Prognostic factor measurement	Clear definition of the prognostic factors measured is provided (e.g. imaging modality method, measurement, and timing described).	x				
	Specified instrument and personnel for measurement of predictive factors		x			
	Continuous variables are reported or appropriate (i.e. not data- dependent) cut-off points are used and specified a priori		x			
	Blinding: were estimators of risk factor status and of outcomes blinded?			x		
	The prognostic factor(s) of interest is (are) adequately measured in study participants to sufficiently limit potential bias	Moderate risk of bias				
Outcome measurement	Is the outcome(s) clearly defined?	x				
	The outcome measure and method used are adequately valid and reliable to limit misclassification bias	Low risk of bias				
Study confounding	Do the authors address potential confounders?		x			
	Important potential confounders are appropriately accounted for, limiting potential bias with respect to the prognostic factor of interest.	Moderate risk of bias				
Analysis and reporting	There is sufficient presentation of data to assess the adequacy of the analysis strategy and there is no selective reporting		x			
	The statistical analysis is appropriate for the study design, limiting potential for the presentation of invalid results	Moderate risk of bias				

NA*: not applicable. Note: The above table was adapted from: Hayden et al., 2013.

Overall opinion of study quality= Moderate risk of bias

First Author: Rocca et al., 1995 **ID:** 74903016

Potential Bias	Items to be considered for assessment of potential opportunity for bias	Yes	Partly	No	Unsure	NA*
Study population /sample selection	Inclusion and exclusion criteria are adequately described [including explicit diagnostic criteria, start/finish date of recruitment]	x				
	Baseline study sample [i.e. individuals entering the study and their key characteristics and sampling frame are adequately described]		x			
	Study sample represents population of interest on key characteristics, sufficient to limit potential bias to results	Moderate risk of bias				
Study attrition	Response rate (i.e., proportion of study sample completing the study and providing outcome data) is adequate					x
	Participants lost to follow-up are adequately described for key characteristics					x
	Statement as to the possible effect on the results from missing data					x
	Loss to follow-up is not associated with key characteristics	Low risk of bias				
Prognostic factor measurement	Clear definition of the prognostic factors measured is provided (e.g. imaging modality method, measurement, and timing described).	x				
	Specified instrument and personnel for measurement of predictive factors		x			
	Continuous variables are reported or appropriate (i.e. not data- dependent) cut-off points are used and specified a priori	x				
	Blinding: were estimators of risk factor status and of outcomes blinded?			x		
	The prognostic factor(s) of interest is (are) adequately measured in study participants to sufficiently limit potential bias	Moderate risk of bias				
Outcome measurement	Is the outcome(s) clearly defined?		x			
	The outcome measure and method used are adequately valid and reliable to limit misclassification bias	Moderate risk of bias				
Study confounding	Do the authors address potential confounders?		x			
	Important potential confounders are appropriately accounted for, limiting potential bias with respect to the prognostic factor of interest.	Moderate risk of bias				
Analysis and reporting	There is sufficient presentation of data to assess the adequacy of the analysis strategy and there is no selective reporting		x			
	The statistical analysis is appropriate for the study design, limiting potential for the presentation of invalid results	Moderate risk of bias				

NA*: not applicable. Note: The above table was adapted from: Hayden et al., 2013.

Overall opinion of study quality= Moderate risk of bias

First Author: Verma et al., 2016 **ID:** 6379243

Potential Bias	Items to be considered for assessment of potential opportunity for bias	Yes	Partly	No	Unsure	NA*
Study population /sample selection	Inclusion and exclusion criteria are adequately described [including explicit diagnostic criteria, start/finish date of recruitment]	x				
	Baseline study sample [i.e. individuals entering the study and their key characteristics and sampling frame are adequately described]	x				
	Study sample represents population of interest on key characteristics, sufficient to limit potential bias to results	Low risk of bias				
Study attrition	Response rate (i.e., proportion of study sample completing the study and providing outcome data) is adequate	x				
	Participants lost to follow-up are adequately described for key characteristics					x
	Statement as to the possible effect on the results from missing data				x	
	Loss to follow-up is not associated with key characteristics	Low risk of bias				
Prognostic factor measurement	Clear definition of the prognostic factors measured is provided (e.g. imaging modality method, measurement, and timing described).	x				
	Specified instrument and personnel for measurement of predictive factors	x				
	Continuous variables are reported or appropriate (i.e. not data- dependent) cut-off points are used and specified a priori	x				
	Blinding: were estimators of risk factor status and of outcomes blinded?				x	
	The prognostic factor(s) of interest is (are) adequately measured in study participants to sufficiently limit potential bias	Low risk of bias				
Outcome measurement	Is the outcome(s) clearly defined?	x				
	The outcome measure and method used are adequately valid and reliable to limit misclassification bias	Low risk of bias				
Study confounding	Do the authors address potential confounders?	x				
	Important potential confounders are appropriately accounted for, limiting potential bias with respect to the prognostic factor of interest.	Low risk of bias				
Analysis and reporting	There is sufficient presentation of data to assess the adequacy of the analysis strategy and there is no selective reporting	x				
	The statistical analysis is appropriate for the study design, limiting potential for the presentation of invalid results	Low risk of bias				
NA*: not applicable. Note: The above table was adapted from: Hayden et al., 2013.						
**Overall opinion of study quality= Low risk of bias**						

For peer review only

First Author: Waa et al., 2010 **ID:** 6379255

**Potential Bias**	**Items to be considered for assessment of potential opportunity for bias**	**Yes**	**Partly**	**No**	**Unsure**	**NA***
Study population /sample selection	Inclusion and exclusion criteria are adequately described [including explicit diagnostic criteria, start/finish date of recruitment]	x				
	Baseline study sample [i.e. individuals entering the study and their key characteristics and sampling frame are adequately described]	x				
	Study sample represents population of interest on key characteristics, sufficient to limit potential bias to results	Low risk of bias				
Study attrition	Response rate (i.e., proportion of study sample completing the study and providing outcome data) is adequate	x				
	Participants lost to follow-up are adequately described for key characteristics	x				
	Statement as to the possible effect on the results from missing data	x				
	Loss to follow-up is not associated with key characteristics	Low risk of bias				
Prognostic factor measurement	Clear definition of the prognostic factors measured is provided (e.g. imaging modality method, measurement, and timing described).	x				
	Specified instrument and personnel for measurement of predictive factors	x				
	Continuous variables are reported or appropriate (i.e. not data- dependent) cut-off points are used and specified a priori	x				
	Blinding: were estimators of risk factor status and of outcomes blinded?			x		
	The prognostic factor(s) of interest is (are) adequately measured in study participants to sufficiently limit potential bias	Low risk of bias				
Outcome measurement	Is the outcome(s) clearly defined?		x			
	The outcome measure and method used are adequately valid and reliable to limit misclassification bias	Moderate risk of bias				
Study confounding	Do the authors address potential confounders?		x			
	Important potential confounders are appropriately accounted for, limiting potential bias with respect to the prognostic factor of interest.	Moderate risk of bias				
Analysis and reporting	There is sufficient presentation of data to assess the adequacy of the analysis strategy and there is no selective reporting		x			
	The statistical analysis is appropriate for the study design, limiting potential for the presentation of invalid results	Moderate risk of bias				
NA*: not applicable. Note: The above table was adapted from: Hayden et al., 2013.						
**Overall opinion of study quality= Moderate risk of bias**						

For peer review only

First Author: Yelikar et al., 2013 **ID:** 6379339

Potential Bias	Items to be considered for assessment of potential opportunity for bias	Yes	Partly	No	Unsure	NA*
Study population /sample selection	Inclusion and exclusion criteria are adequately described [including explicit diagnostic criteria, start/finish date of recruitment]	x				
	Baseline study sample [i.e. individuals entering the study and their key characteristics and sampling frame are adequately described]	x				
	Study sample represents population of interest on key characteristics, sufficient to limit potential bias to results	Low risk of bias				
Study attrition	Response rate (i.e., proportion of study sample completing the study and providing outcome data) is adequate		x			
	Participants lost to follow-up are adequately described for key characteristics				x	
	Statement as to the possible effect on the results from missing data				x	
	Loss to follow-up is not associated with key characteristics	Moderate risk of bias				
Prognostic factor measurement	Clear definition of the prognostic factors measured is provided (e.g. imaging modality method, measurement, and timing described).	x				
	Specified instrument and personnel for measurement of predictive factors		x			
	Continuous variables are reported or appropriate (i.e. not data- dependent) cut-off points are used and specified a priori	x				
	Blinding: were estimators of risk factor status and of outcomes blinded?			x		
	The prognostic factor(s) of interest is (are) adequately measured in study participants to sufficiently limit potential bias	Moderate risk of bias				
Outcome measurement	Is the outcome(s) clearly defined?		x			
	The outcome measure and method used are adequately valid and reliable to limit misclassification bias	Moderate risk of bias				
Study confounding	Do the authors address potential confounders?	x				
	Important potential confounders are appropriately accounted for, limiting potential bias with respect to the prognostic factor of interest.	Low risk of bias				
Analysis and reporting	There is sufficient presentation of data to assess the adequacy of the analysis strategy and there is no selective reporting		x			
	The statistical analysis is appropriate for the study design, limiting potential for the presentation of invalid results	Moderate risk of bias				
NA*: not applicable. Note: The above table was adapted from: Hayden et al., 2013.						
**Overall opinion of study quality= Moderate risk of bias**						

First Author: Zarean et al., 2018 **ID:** 6379369

**Potential Bias**	**Items to be considered for assessment of potential opportunity for bias**	**Yes**	**Partly**	**No**	**Unsure**	**NA***
Study population /sample selection	Inclusion and exclusion criteria are adequately described [including explicit diagnostic criteria, start/finish date of recruitment]	x				
	Baseline study sample [i.e. individuals entering the study and their key characteristics and sampling frame are adequately described]		x			
	Study sample represents population of interest on key characteristics, sufficient to limit potential bias to results	Moderate risk of bias				
Study attrition	Response rate (i.e., proportion of study sample completing the study and providing outcome data) is adequate	x				
	Participants lost to follow-up are adequately described for key characteristics					x
	Statement as to the possible effect on the results from missing data					x
	Loss to follow-up is not associated with key characteristics	Low risk of bias				
Prognostic factor measurement	Clear definition of the prognostic factors measured is provided (e.g. imaging modality method, measurement, and timing described).	x				
	Specified instrument and personnel for measurement of predictive factors	x				
	Continuous variables are reported or appropriate (i.e. not data- dependent) cut-off points are used and specified a priori	x				
	Blinding: were estimators of risk factor status and of outcomes blinded?				x	
	The prognostic factor(s) of interest is (are) adequately measured in study participants to sufficiently limit potential bias	Low risk of bias				
Outcome measurement	Is the outcome(s) clearly defined?		x			
	The outcome measure and method used are adequately valid and reliable to limit misclassification bias	Moderate risk of bias				
Study confounding	Do the authors address potential confounders?	x				
	Important potential confounders are appropriately accounted for, limiting potential bias with respect to the prognostic factor of interest.	Low risk of bias				
Analysis and reporting	There is sufficient presentation of data to assess the adequacy of the analysis strategy and there is no selective reporting	x				
	The statistical analysis is appropriate for the study design, limiting potential for the presentation of invalid results	Low risk of bias				
NA*: not applicable. Note: The above table was adapted from: Hayden et al., 2013.						
**Overall opinion of study quality= Low risk of bias**						

Table S1. Statistical measures of prognostic performance of Doppler ultrasound reported in the selected studies

**Prognostic determinant**	**Outcome**	**Studies**	**Sn**	**Sp**	**PPV**	**NPV**	**AUROC**	**Diagnostic accuracy**	**OR [95% CI]**	**RR [95% CI]**	**Correlation**	**Normal Doppler n (%)**	**Abnormal Doppler n (%)**
**UA flow impedance**	FGR	Agbaje et al., 2018	67.00	53.00			0.63						
		Mullick et al., 1993	85.00	89.00	88.50								
		Najam et al., 2016	48.15	80.67	53.06	77.40							
		Rocca et al., 1995	92.30	91.90	77.40	97.60		92.0					
		Khanduri et al., 2013	73.80	75.90	87.70	55.40		75.00					
		Bano et al., 2010	46.70	93.30	87.50	63.60		70.00					
		Nagar et al., 2015	42.86	94.62	37.50	95.65							
	NICU Admission	Anshul et al., 2010										13 (24.07)	36 (78.2)
		Najam et al., 2016	50.00	80.30	48.90	80.95							
	Fetal Distress	Anshul et al., 2010										18 (33)	35 (76)
		Rocca et al., 1995										2 (2.5)	12 (39)
		Najam et al., 2016	66.67	78.04	74.89	89.72							
		Yelikar et al., 2013	42.10	65.90	12.10	91.10							
	Stillbirth	Anshul et al., 2010										0 (0)	4 (9.5)
		Najam et al., 2016										0 (0)	5 (8.2)
	Perinatal death	Rocca et al., 1995										0 (0)	2 (6.5)
		Anshul et al., 2010										0 (0)	9 (60)
	LBW	Anshul et al., 2010										15 (27.0)	35 (77.8)
	Apgar Score	Rocca et al., 1995	80.00	82.40	41.00	96.00		83.00					
		Anshul et al., 2010										2 (3.7)	14 (82.35)
		Najam et al., 2016										3 (60.0)	6 (85.71)
		Agbaje et al., 2018									0.378		
	Fetal Anemia	Kumari et al., 2019									0.21		
	HIE	Najam et al., 2016										1 (1.29)	8 (16.31)
	MAS	Najam et al., 2016										1 (1.29)	16 (32.65)
	CAPO	Bano et al., 2010	79.20	92.40	79.20	92.20		88.90					
		Lakhkar et al 2006	50.00	59.00	66.60	41.90							

For peer review only

		Rani et al., 2016	17.80	95.80	80.70	50.50	0.57						
		Geerts et al., 2007	75.00			95.00			0.6 (0.1, 4.1)				
		Malik et al., 2013	64.40	80.00	96.60	20.00							
		Pattinson et al., 1993	12.50	91.80	22.70	84.50							
		Ebrashy et al., 2005	53.30	36.40	81.10	30.80							
		Waa et al., 2010	8.00	100.00	0.00	26.00							
**UA AREDF**	Perinatal death	Lakshmi et al., 2013							9.8 (2.1, 46.4)				
		Najam et al., 2016										2 (2.59)	4 (33.33)
	RDS	Lakshmi et al., 2013							2.4 (1.1, 5.0)				
	CAPO	Pattinson et al., 1991	75.00	90.00	69.00								
		Lakshmi et al., 2013							8.4 (2.3, 30.5)				
**MCA flow impedance**	FGR	Najam et al., 2016	59.25	88.89	72.72	81.35							
		Bano et al., 2010	8.90	100.0	100.0	52.30		54.40					
		Khanduri et al., 2013	26.20	92.60	89.20	35.00		46.10					
	Fetal Anemia	Pares et al., 2008	100.00	65.00	90.90	100.0		92.20					
		Kumari et al., 2019	68.00	57.00	83.00	33.00	0.70				-0.43		
	NICU Admission	Najam et al., 2016	64.58	88.69	70.45	85.71							
	Neonatal Acidosis	Allam et al., 2013	87.50	64.00	74.00	82.00	0.82						
	Fetal Distress	Najam et al., 2016	72.73	78.05	54.55	91.53							
	Stillbirth	Najam et al., 2016										0 (0)	2 (4.5)
	Apgar Score	Najam et al., 2016										1 (1.29)	17 (38.6)
	HIE	Najam et al., 2016										1 (1.29)	10 (22.72)
	MAS	Najam et al., 2016										1 (1.29)	20 (45.5)
	CAPO	Bano et al., 2010	16.70	100.0	100.0	76.70		77.80					
		Lakhkar et al 2006	41.60	90.90	88.20	48.70							
		Rani et al., 2016	18.60	90.30	68.70	49.40	0.58						
		Dhand et al., 2011	71.00	92.00	94.00	65.00							
		Malik et al., 2013	7.70	90.00	87.50	9.80							
		Ebrashy et al., 2005	41.00	63.60	80.00	23.30							
		Waa et al., 2010	23.0	68.00	76.00	33.00							

For peer review only

**CPR**	FGR	Najam et al., 2016	85.10	89.72	80.70	92.30							
		Bano et al., 2010						72.20					
	NICU Admission	Najam et al., 2016	75.00	82.92	63.15	89.47							
		Alanwar et al., 2018	62.50	71.42	29.40	90.90							
	Foetal Distress	Najam et al., 2016	90.91	78.04	52.63	96.97							
		Masihi et al.2019	80.95	50.00	17.50	95.20							
	Stillbirth	Najam et al., 2016										0 (0)	4 (7.14)
	Apgar Score	Najam et al., 2016										1 (1.29)	19 (33.33)
		Alanwar et al., 2018	50.0	88.10	44.40	90.20							
	Neonatal Acidosis	Ebrashy et al., 2005	64.10	72.70	89.30	36.40				1.4 (1.2, 1.7)			
		Alanwar et al., 2018	43.75	69.05	21.21	86.57							
	HIE	Najam et al., 2016										1 (1.29)	12 (21.05)
	MAS	Najam et al., 2016	96.15			99.20						1 (1.29)	25 (43.85)
	CAPO	Bano et al., 2010	83.30	100.0	100.00	94.30		95.60					
		Lakhkar et al 2006	47.20	86.30	85.00	50.00							
		Rani et al., 2016	7.60	98.00	81.80	48.30	0.60						
		Malik et al., 2013	68.80	100.00	100.0	26.30							
		Geerts et al., 2007			57.0				1.1 (0.1, 14.6)				
**UtA flow impedance**	FGR	Verma et al., 2016	45.0	84.10	28.10	91.70							
		Phupong et al., 2003	67.0	82.90	6.90	99.20				9.1 (1.7, 48.5)			
		Nagar et al., 2015	25.0	94.56	28.57	93.55							
	Perinatal Death	Dorman et al., 2002								2.37 (1.3, 4.3)			
	LBW	Verma et al., 2016	45.40	84.60	31.30	90.90							
		Dorman et al., 2002								2.52 (1.5, 4.2)			
	Preterm Birth	Verma et al., 2016	57.10	63.20	18.50	91.00							
		Dorman et al., 2002								1.53 (0.9, 2.4)			

For peer review only

	CAPO	Verma et al., 2016	48.20	95.40	84.40	78.20							
		Nouh et al., 2011	84.60	96.30	91.70	92.90							
		Malik et al., 2013	37.70	70.00	91.80	11.00							
		Zarean et al., 2018	37.50	73.30	48.40	63.70	0.55						
**FDA flow impedance**	Fetal anemia	Pares et al., 2008	95.70	100.0	100.0	86.90		96.70					
		Kumari et al., 2019	87.00	57.00			0.80				-0.54		
	CAPO	Lakhkar et al 2006	44.40	59.00	64.00	56.50							
**FDA & MCA**	Fetal anemia	Pares et al., 2008	98.40	100.0	100.0	91.70		98.60					
		Kumari et al., 2019	86.00	67.00	86.00	67.00							
**DV flow impedance**	Neonatal Acidosis	Allam et al., 2013	100.0	57.00	72.0	100.0	0.88	80.00					
	CAPO	Geerts et al., 2007		92.0	33.0				0.3 (0.03, 4.6)				

[a]UA: umbilical artery; MCA: middle cerebral artery; CPR: cerebroplacental ratio; UtA: uterine artery; FDA: fetal descending aorta; DV: ductus venosus; RI: resistive index; PI: pulsatility index; S/D ratio: systolic diastolic ratio; PSV: peak systolic velocity; MV: mean velocity; AREDF: absent and/or reversed end diastolic flow; FGR: fetal growth restriction; LBW: low birth weight; HIE: hypoxic ischemic encephalopathy; MAS: meconium aspiration syndrome; RDS: respiratory distress syndrome; NICU: neonatal intensive care unit; CAPO: composite adverse perinatal outcomes; Sn: sensitivity; Sp: specificity; PPV: positive predictive value; NPV: negative predictive value; OR: odds ratio; RR: relative risk; and n (%): frequency (percentage).

For peer review only

Table S2. Definitions of adverse perinatal outcomes reported in the selected studies

First Author	Outcomes	Definition (detailed description in the article)
Abdallah et al., 2019	LBW	Not defined
	NICU admission	Not defined
	Stillbirth	Not defined
	Perinatal mortality	Not defined
	Low APGAR score (1min & 5min)	Not defined
Agbaje et al., 2018	FGR	Abnormal birth weight: defined as estimated foetal weight below the 10th percentile for gestational age and abdominal circumference below the 10th percentile for gestational age.
	Low APGAR score at 5 minutes	APGAR score less than 6
Alanwar et al., 2018	Acidosis	Neonatal academia of pH < 7.2
	NICU admission	New-born was admitted to the neo- natal intensive care unit
	Low APGAR score at 5 minutes	APGAR score < 7 at 5 min
Allam et al., 2013	Neonatal acidosis	Cord blood pH <7.25
Anshul et al., 2010	Stillbirth	Not defined
	Neonatal death	Not defined
	NICU admission	Admission required
	Foetal distress	Delivered by emergency caesarean section for suspected foetal distress
	LBW	Not defined
	Low APGAR score at birth.	APGAR score <7 at birth
Bano et al., 2010	Perinatal death	Not defined
	Foetal distress	Caesarean section for foetal distress (FD not defined)
	NICU admission	Not defined
	Low APGAR score at 5min	APGAR score <7 at 5 min
	FGR	Birth weight less than 10th percentile for gestational age

For peer review only

	Composite adverse perinatal outcome	Not defined
Dhand et al., 2011	Composite adverse perinatal outcome	Abnormal foetal outcome (details not provided)
Dorman et al., 2002	Perinatal death	Not defined
	Preterm delivery	Delivery < 37 weeks
	LBW	Birth weight <2.5kg
Ebrashy et al., 2005	Acidosis	Neonatal acidaemia of pH<7.2 were present
	Composite adverse neonatal outcome	Neonatal morbidity (neonatal academia pH<7.2, 5-minute APGAR score <6, and/or admission to NICU)
Geerts et al., 2007	Composite adverse perinatal outcome	Poor outcome (perinatal demise or clinical/ultrasound signs of neurological compromise in the infant at the time of discharge from the tertiary institution)
Khanduri et al., 2013	FGR	Ponderal index was calculated as birth weight (in gm) per length (in cm^3). Ponderal index of <10 indicates growth restriction.
Kumari et al., 2019	Foetal anaemia	Haematocrit of the umbilical cord blood was used as the reference test to diagnose foetal anaemia (defined as haemoglobin <0.65 times the median for gestational age).
Lakhkar et al., 2006	Composite adverse perinatal outcome	Adverse perinatal outcome (Major and Minor). Major adverse outcomes were perinatal deaths including intrauterine and early neonatal deaths. Major complications like hypoxic ischemic encephalopathy, intraventricular haemorrhage, periventricular leukomalacia, pulmonary haemorrhage and necrotizing enterocolitis. Minor outcomes include-caesarean delivery for foetal distress, APGAR score below 7 at 5 minutes, admission to NICU (neonatal intensive care unit) for treatment.
Lakshmi et al., 2013	Neonatal death	Not defined
	Respiratory distress syndrome	Not defined
	Composite adverse perinatal outcome	Composite outcome of death or major neuro-morbidity at 12-18 months of corrected age, defined as presence of cerebral palsy or visual or hearing impairment.
Malik et al., 2013	Composite adverse perinatal outcome	Abnormal foetal outcome (IUGR, IUFD and perinatal mortality)
Masihi et al.2019	Intrapartum foetal distress	Emergency caesarean section for foetal distress
Mullick et al., 1993	FGR	Not defined
Nagar et al., 2015	FGR	Not defined
Najam et al., 2016	FGR	Not defined

For peer review only

	NICU admission	Not defined
	Foetal distress	Not defined
	Stillbirth	Not defined
	Neonatal death	Not defined
	Low APGAR score	Not defined
	Hypoxic ischemic encephalopathy	Not defined
	Meconium aspiration syndrome	Not defined.
Nouh et al., 2011	Composite adverse perinatal outcome	The presence of one or more of the following; miscarriage, gestational DM, PIH, PE, antepartum haemorrhage, intrauterine growth retardation, instrumental, caesarean delivery and preterm labour.
Pares et al., 2008	Foetal anaemia	Anaemia was considered moderate to severe when foetal haemoglobin concentrations were < or =0.64 multiples of the median for gestational age.
Pattinson et al., 1991	Composite adverse perinatal outcome	Poor foetal outcome (details not provided).
Pattinson et al., 1993	Composite adverse perinatal outcome	Complications of pregnancy, namely intra-uterine growth retardation and proteinuric hypertension.
Phupong et al., 2003	FGR	Birth weight less than 10 percentile for gestational age.
Rani et al., 2016	Composite adverse perinatal outcome	Adverse perinatal outcome was defined as any of these: small for gestational age, still birth, APGAR score <5 at 5 minutes, need of bag and mask ventilation for >10 minutes or hypoxic ischemic encephalopathy, admission to neonatal intensive care unit (NICU), and caesarean section due to non-reassuring foetal heart rate.
Rocca et al., 1995	IUGR	Not defined.
	Low APGAR score 5mins	APGAR score <7 at 5 minutes.
	Perinatal death	Not defined.
	Foetal distress	Emergency operative delivery for foetal distress.
Verma et al., 2016	FGR	Not defined.
	LBW	Birth weight <2500 gm.
	Preterm delivery	Spontaneous delivery <37 weeks.

For peer review only

For peer review only - http://bmjopen.bmj.com/site/about/guidelines.xhtml

	Composite adverse perinatal outcome	At least one adverse outcome (preeclampsia, FGR, low birth weight, spontaneous preterm delivery, oligohydramnios, foetal loss).
Waa et al., 2010	Composite adverse perinatal outcome	Poor outcome was defined by foetal mortality or appearance, pulse rate, grimace, activity, respiration (APGAR) score less than eight at five minutes or weight less than 10th percentile for gestation 20 or head circumference and length below 10th percentile for gestation.
Yelikar et al., 2013	Intrapartum foetal distress	Delivered by emergency caesarean section for suspected foetal distress.
Zarean et al., 2018	Composite adverse perinatal outcome	Adverse perinatal outcome, including preterm labour, intrauterine foetal death, PE, low 5-min APGAR score (<7), low umbilical arterial cord blood pH, admitted to Intensive Care Unit in the first 3 days of birth, low birth weight, infant with low weight, death of new-borns, caesarean section for respiratory distress, and meconial amniotic fluid.

[a]FGR: fetal growth restriction; FGR: intrauterine growth restriction; LBW: low birth weight; NICU: neonatal intensive care unit.

For peer review only

Reporting checklist for systematic review and meta-analysis.

Based on the PRISMA guidelines.

Instructions to authors

Complete this checklist by entering the page numbers from your manuscript where readers will find each of the items listed below.

Your article may not currently address all the items on the checklist. Please modify your text to include the missing information. If you are certain that an item does not apply, please write "n/a" and provide a short explanation.

Upload your completed checklist as an extra file when you submit to a journal.

In your methods section, say that you used the PRISMAreporting guidelines, and cite them as:

Moher D, Liberati A, Tetzlaff J, Altman DG, The PRISMA Group. Preferred Reporting Items for Systematic Reviews and Meta-Analyses: The PRISMA Statement

		Reporting Item	Page Number
**Title**			
	#1	Identify the report as a systematic review, meta-analysis, or both.	1
**Abstract**			
Structured summary	#2	Provide a structured summary including, as applicable: background; objectives; data sources; study eligibility criteria, participants, and interventions; study appraisal and synthesis methods; results; limitations; conclusions and implications of key findings; systematic review registration number	2
**Introduction**			
Rationale	#3	Describe the rationale for the review in the context of what is already known.	3

For peer review only

Objectives	#4	Provide an explicit statement of questions being addressed with reference to participants, interventions, comparisons, outcomes, and study design (PICOS).	3
**Methods**			
Protocol and registration	#5	Indicate if a review protocol exists, if and where it can be accessed (e.g., Web address) and, if available, provide registration information including the registration number.	4
Eligibility criteria	#6	Specify study characteristics (e.g., PICOS, length of follow-up) and report characteristics (e.g., years considered, language, publication status) used as criteria for eligibility, giving rational	4
Information sources	#7	Describe all information sources in the search (e.g., databases with dates of coverage, contact with study authors to identify additional studies) and date last searched.	4
Search	#8	Present full electronic search strategy for at least one database, including any limits used, such that it could be repeated.	4
Study selection	#9	State the process for selecting studies (i.e., for screening, for determining eligibility, for inclusion in the systematic review, and, if applicable, for inclusion in the meta-analysis).	4-5
Data collection process	#10	Describe the method of data extraction from reports (e.g., piloted forms, independently by two reviewers) and any processes for obtaining and confirming data from investigators.	5
Data items	#11	List and define all variables for which data were sought (e.g., PICOS, funding sources), and any assumptions and simplifications made.	4
Risk of bias in individual studies	#12	Describe methods used for assessing risk of bias in individual studies (including specification of whether this was done at the study or outcome level, or both), and how this information is to be used in any data synthesis.	5
Summary measures	#13	State the principal summary measures (e.g., risk ratio, difference in means).	5
Planned methods of analyis	#14	Describe the methods of handling data and combining results of studies, if done, including measures of consistency (e.g., I2) for each meta-analysis.	5

For peer review only

Risk of bias across studies	#15	Specify any assessment of risk of bias that may affect the cumulative evidence (e.g., publication bias, selective reporting within studies).	5
Additional analyses	#16	Describe methods of additional analyses (e.g., sensitivity or subgroup analyses, meta-regression), if done, indicating which were pre-specified.	na
**Results**			
Study selection	#17	Give numbers of studies screened, assessed for eligibility, and included in the review, with reasons for exclusions at each stage, ideally with a flow diagram.	6
Study characteristics	#18	For each study, present characteristics for which data were extracted (e.g., study size, PICOS, follow-up period) and provide the citation.	6
Risk of bias within studies	#19	Present data on risk of bias of each study and, if available, any outcome-level assessment (see Item 12).	5
Results of individual studies	#20	For all outcomes considered (benefits and harms), present, for each study: (a) simple summary data for each intervention group and (b) effect estimates and confidence intervals, ideally with a forest plot.	7-9
Synthesis of results	#21	Present the main results of the review. If meta-analyses are done, include for each, confidence intervals and measures of consistency.	7-9
Risk of bias across studies	#22	Present results of any assessment of risk of bias across studies (see Item 15).	6
Additional analysis	#23	Give results of additional analyses, if done (e.g., sensitivity or subgroup analyses, meta-regression [see Item 16]).	na
**Discussion**			
Summary of Evidence	#24	Summarize the main findings, including the strength of evidence for each main outcome; consider their relevance to key groups (e.g., health care providers, users, and policy makers	8
Limitations	#25	Discuss limitations at study and outcome level (e.g., risk of bias), and at review level (e.g., incomplete retrieval of identified research, reporting bias).	10
Conclusions	#26	Provide a general interpretation of the results in the context of other evidence, and implications for future research.	11

For peer review only

Funding

Funding	#27	Describe sources of funding or other support (e.g., supply of data) for the systematic review; role of funders for the systematic review.	11

The PRISMA checklist is distributed under the terms of the Creative Commons Attribution License CC-BY. This checklist was completed on 02. February 2021 using https://www.goodreports.org/, a tool made by the EQUATOR Network in collaboration with Penelope.ai

For peer review only
